# Halogen Complexes of Anionic N‐Heterocyclic Carbenes

**DOI:** 10.1002/chem.202004418

**Published:** 2020-12-21

**Authors:** Jenni Frosch, Marvin Koneczny, Thomas Bannenberg, Matthias Tamm

**Affiliations:** ^1^ Institut für Anorganische und Analytische Chemie Technische Universität Braunschweig Hagenring 30 38106 Braunschweig Germany

**Keywords:** halogen bonding, hypervalency, iodine, London dispersion, N-heterocyclic carbenes

## Abstract

The lithium complexes [(WCA‐NHC)Li(toluene)] of anionic N‐heterocyclic carbenes with a weakly coordinating anionic borate moiety (WCA‐NHC) reacted with iodine, bromine, or CCl_4_ to afford the zwitterionic 2‐halogenoimidazolium borates (WCA‐NHC)X (X=I, Br, Cl; WCA=B(C_6_F_5_)_3_, B{3,5‐C_6_H_3_(CF_3_)_2_}_3_; NHC=IDipp=1,3‐bis(2,6‐diisopropylphenyl)imidazolin‐2‐ylidene, or NHC=IMes=1,3‐bis(2,4,6‐trimethylphenyl)imidazolin‐2‐ylidene). The iodine derivative (WCA‐IDipp)I (WCA=B(C_6_F_5_)_3_) formed several complexes of the type (WCA‐IDipp)I**⋅**L (L=C_6_H_5_Cl, C_6_H_5_Me, CH_3_CN, THF, ONMe_3_), revealing its ability to act as an efficient halogen bond donor, which was also exploited for the preparation of hypervalent bis(carbene)iodine(I) complexes of the type [(WCA‐IDipp)I(NHC)] and [PPh_4_][(WCA‐IDipp)I(WCA‐NHC)] (NHC=IDipp, IMes). The corresponding bromine complex [PPh_4_][(WCA‐IDipp)_2_Br] was isolated as a rare example of a hypervalent (10‐Br‐2) system. DFT calculations reveal that London dispersion contributes significantly to the stability of the bis(carbene)halogen(I) complexes, and the bonding was further analyzed by quantum theory of atoms in molecules (QTAIM) analysis.

## Graphical abstract

**Halogen complexes** with N‐heterocyclic carbenes with a weakly coordinating anionic borate moiety, (WCA‐NHC)X (X=I, Br,Cl), were prepared and their ability to serve as halogen bond donors was investigated. Their reaction with carbenes afforded bis(carbene)iodine(I) and ‐bromine(I) complexes with an unusual hypervalent 10‐X‐2 bonding situation.

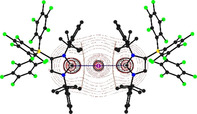

## Introduction

**Scheme 1:**
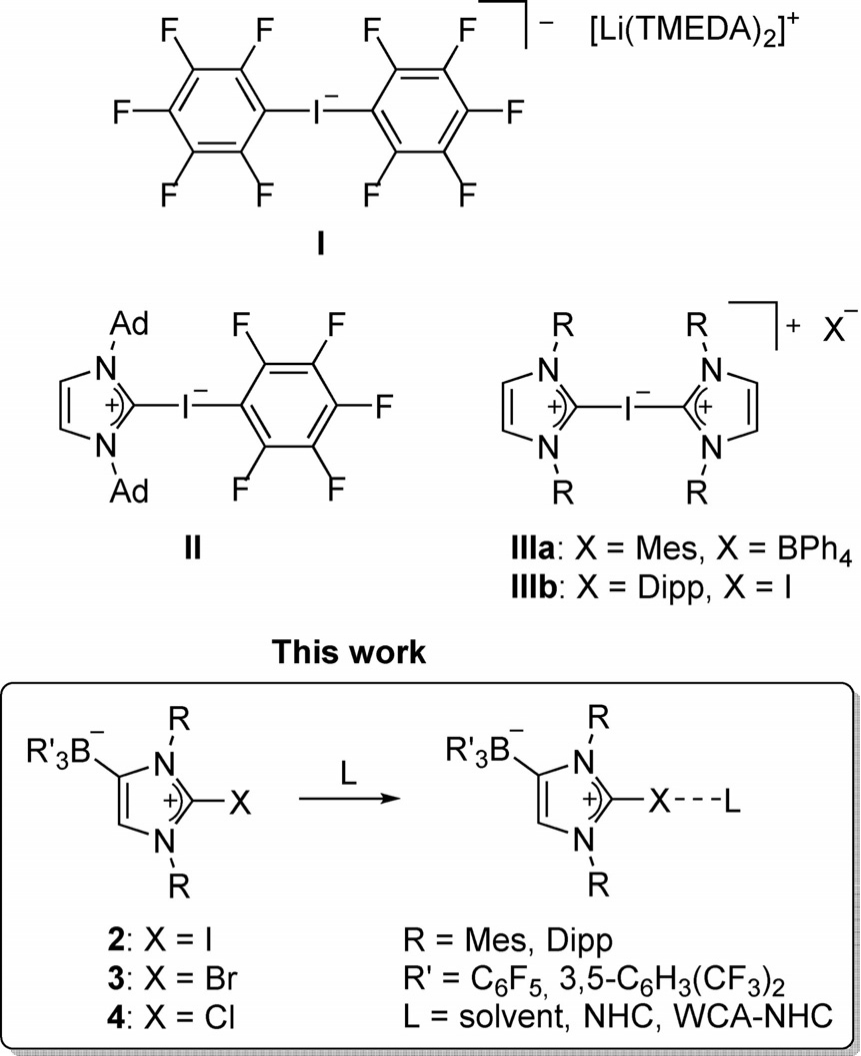
Examples of hypervalent iodine(I) species (10‐I‐2).

Studies of hypervalent iodine species have focused largely on iodine(III) (λ^3^‐iodane, “iodinane”) and iodine(V) (λ^5^‐iodane, “periodinane”) species.^[1]^ In contrast, examples of stable hypervalent iodine(I) species (10‐I‐2)^[2]^ are rare with exceptions such as trihalide anions [IX_2_]^−^ (X=I, Cl, F)^[3]^ and bis(pyridine)iodine(I) cations [I(Py)_2_]^+^.[^4^, ^5^] The first isolation and structural characterization of a hypervalent organoiodine(I) compound was reported by Farnham et al. in 1986, who prepared the bis(pentafluorophenyl)iodate(I) **I** by reaction of iodopentafluorobenzene (C_6_F_5_I) with (pentafluorophenyl)lithium in the presence of tetramethylethylenediamine (TMEDA).^[6]^ The geometrical factors of this compound—a nearly linear C‐I‐C arrangement (175.2(2)°) with long carbon–iodine bond lengths (2.331(5) Å, 2.403(6) Å)—are consistent with a hypervalent 10‐I‐2 system in which the C_6_F_5_ groups serve as apical ligands in a three‐center, four‐electron (3c4e) bonding scheme (Scheme 1).[^7^, ^8^, ^9^] The related species **II** was obtained by Arduengo III, et al. shortly after the report of a stable crystalline carbene, 1,3‐di‐1‐adamantylimidazolin‐2‐ylidene (IAd).^[10]^ Its reaction with C_6_F_5_I furnished the carbene complex **II** with a large C‐I‐C angle of 178.9(2)° and considerably different carbon–iodine bond lengths of C_*ipso*_−I=2.159(3) Å and C_carbene_−I=2.754(3) Å.^[11]^ The availability of other nucleophilic carbenes such as 1,3‐bis(2,4,6‐trimethylphenyl)imidazolin‐2‐ylidene (IMes)^[12]^ gave access to the bis(carbene) adduct [(IMes)_2_I][BPh_4_] (**III a**) with a linear and more symmetric C‐I‐C arrangement (177.5(2)°, 2.286(4) Å, 2.363(4) Å). More recently, the corresponding bis(carbene) complex [(IDipp)_2_I]I (**III b**) was obtained from the reaction of 1,3‐bis(2,6‐diisopropylphenyl)imidazolin‐2‐ylidene (IDipp) with 0.5 equivalents of I_2_, and its structural characterization afforded similar geometrical features for the C‐I‐C moiety (178.8(3)°, 2.302(7) Å, 2.363(7) Å).^[27]^ Isocyanide adducts of iodofluorobenzenes also reveal linear C‐I‐C arrangements in the solid state;^[13]^ however, the much longer C_isocyanide_−I distances, for example, 3.134(6) Å in [(C_6_F_5_)I(CNMes)] (CNMes=2,4,6‐trimethylphenyl isocyanide) indicate a significantly weaker interaction, which was described within the concept of noncovalent charge‐transfer or halogen bonding, respectively.[^14^, ^15^, ^16^]

Halogen bonding is also an important issue in azolium‐based compounds such as 2‐iodo‐ and 2‐bromoimidazolium salts, which serve as important entities in organic synthesis and catalysis,[^17^, ^18^] anion recognition,[^19^, ^20^] and supramolecular chemistry.^[21]^ In these systems, the electrophilicity of the polarized halogen atom is exploited, which formally acts as a halogen bond (XB) donor towards nucleophilic substrates or anions. Such interactions are usually explained by the presence of a region of positive electrostatic potential, the so‐called σ‐hole,^[22]^ on the outermost portion of the halogen's surface, centered on the C−X axis, which also accounts for the directional (linear) nature of the XB interaction. Numerous 2‐iodo‐, 2‐bromo‐, and 2‐chloroimidazolium salts are known, many of which have been prepared directly from N‐heterocyclic carbenes (NHC) by reaction with elemental halogens or conventional halogenation reagents.^[23]^ In the solid state, these species may reveal weak, noncovalent halogen–halogen interactions with linear C‐X‐X arrangements, as, for instance, found in the iodine and bromine systems [(IMes)I]I,[^24^, ^25^] [(IMes)Br]Br,^[26]^ [(IDipp)X]X (X=I, Br).^[27]^ The halide counterion in these imidazolium salts can be replaced by less and more weakly coordinating counterions, which facilitates the possible coordination to a Lewis basic substrate and the ability of the cationic halogen donor to promote organocatalytic reactions.[^25^, ^28^]

Based on our original interest in the chemistry of frustrated N‐heterocyclic carbene–borane Lewis pairs,^[29]^ our group has developed anionic N‐heterocyclic carbenes,^[30]^ which bear a weakly coordinating anionic (WCA) fluoroborate moiety, for example, B(C_6_F_5_)_3_ or B(*m*‐XyF_6_)_3_ (*m*‐XyF_6_=3,5‐C_6_H_3_(CF_3_)_2_), in the 4‐position of the imidazole heterocycle. These WCA‐NHC ligands have been used extensively in transition‐metal chemistry and homogeneous catalysis, providing, for instance, neutral or coordinatively unsaturated derivatives of related cationic NHC complexes for applications in nonpolar solvents.[^31^, ^32^, ^33^] Moreover, these WCA‐NHC ligands were introduced into the chemistry of the p‐block elements, particularly the pnictogens and chalcogens.[^34^, ^35^] In a continuation of this work, we wish to present herein the preparation of halogen complexes, as the reaction of the anionic WCA‐NHC ligands (as their lithium salts) with halogens or halogenation reagents will afford the neutral 2‐halogenoimidazolium borate zwitterions **2**–**4**, which will allow to study their interaction with additional ligands (L) in the absence of competing counterions (Scheme 1).

## Results and Discussion

### Synthesis and characterization of 2‐halogenoimidazolium borates

**Scheme 2:**
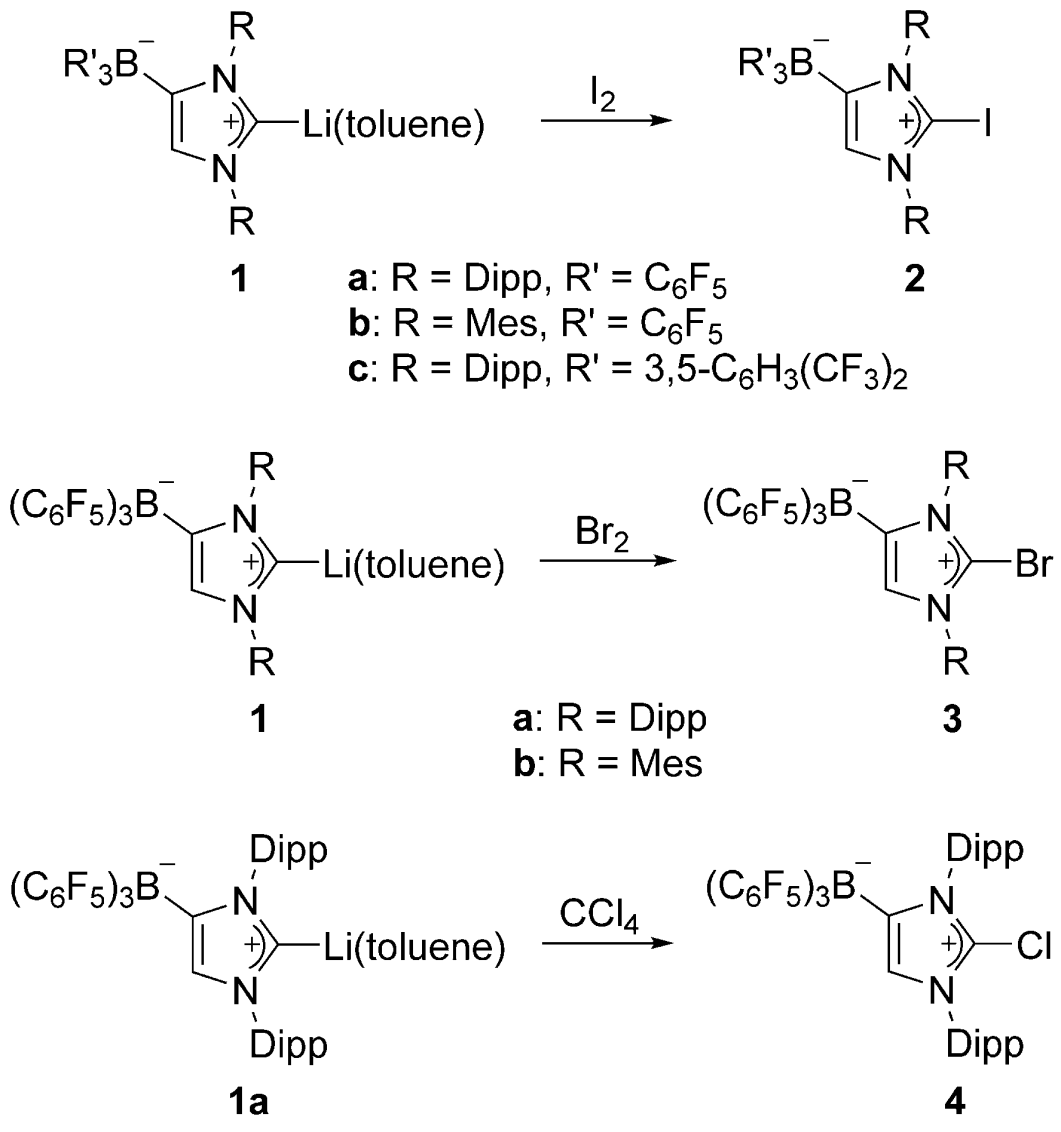
Synthesis of 2‐iodo‐, 2‐bromo‐, and 2‐chloroimidazolium borates. Dipp=2,6‐diisopropylphenyl, Mes=2,4,6‐trimethylphenyl.

The lithium salts **1 a**–**1 c** were isolated as toluene solvates according to published procedures[^32^, ^33^] and their suspensions in chlorobenzene were treated with a solution of iodine in the same solvent at room temperature (Scheme 2). Iodine consumption could be followed by the instantaneous discoloration of the reaction solution. The 2‐iodoimidazolium borates **2 a**–**2 c** were isolated in moderate to high yields (64–86 %) by filtration and evaporation. Characterization by ^1^H, ^13^C{^1^H}, ^11^B{^1^H}, and ^19^F{^1^H} NMR spectroscopy was performed in [D_8_]THF solution. The ^1^H NMR spectra of **2 a**–**2 c** exhibit characteristic singlets for the backbone CH hydrogen atoms at 7.33 ppm (**2 a**) 7.69 ppm (**2 b**), and 7.72 ppm (**2 c**), which are shifted to significantly lower field values compared with the lithiocarbene precursors **1 a**–**1 c**, namely from 6.11 ppm (**1 a**), 6.73 ppm (**1 b**), and 6.30 ppm (**1 c**). In addition, two sets of signals are observed for the Dipp or Mes substituents owing to the presence of the borate moiety. In the ^13^C{^1^H} NMR spectra, the signals for the carbene carbon atoms are found at 105.5 ppm (**2 a**), 102.7 ppm (**2 b**), and 106.2 ppm (**2 c**), which is in good agreement with the chemical shift reported for [(IMes)I]BPh_4_ (107.1 ppm in CD_3_CN).^[24]^ The ^11^B{^1^H} NMR signals are found as sharp singlets at −15.0 ppm (**2 a**) and −16.0 ppm (**2 b**) for the B(C_6_F_5_)_3_ and at −8.0 ppm (**2 c**) for the B(*m*‐XyF_6_)_3_ moieties. The ^19^F{^1^H} NMR spectra of **2 a** and **2 b** show three signals for the fluorine atoms in the *ortho*‐, *meta*‐, and *para*‐positions at approximately −130, −162, and −166 ppm, whereas one signal is found at −62.3 ppm for the CF_3_ groups in **2 c**.

**Figure 1:**
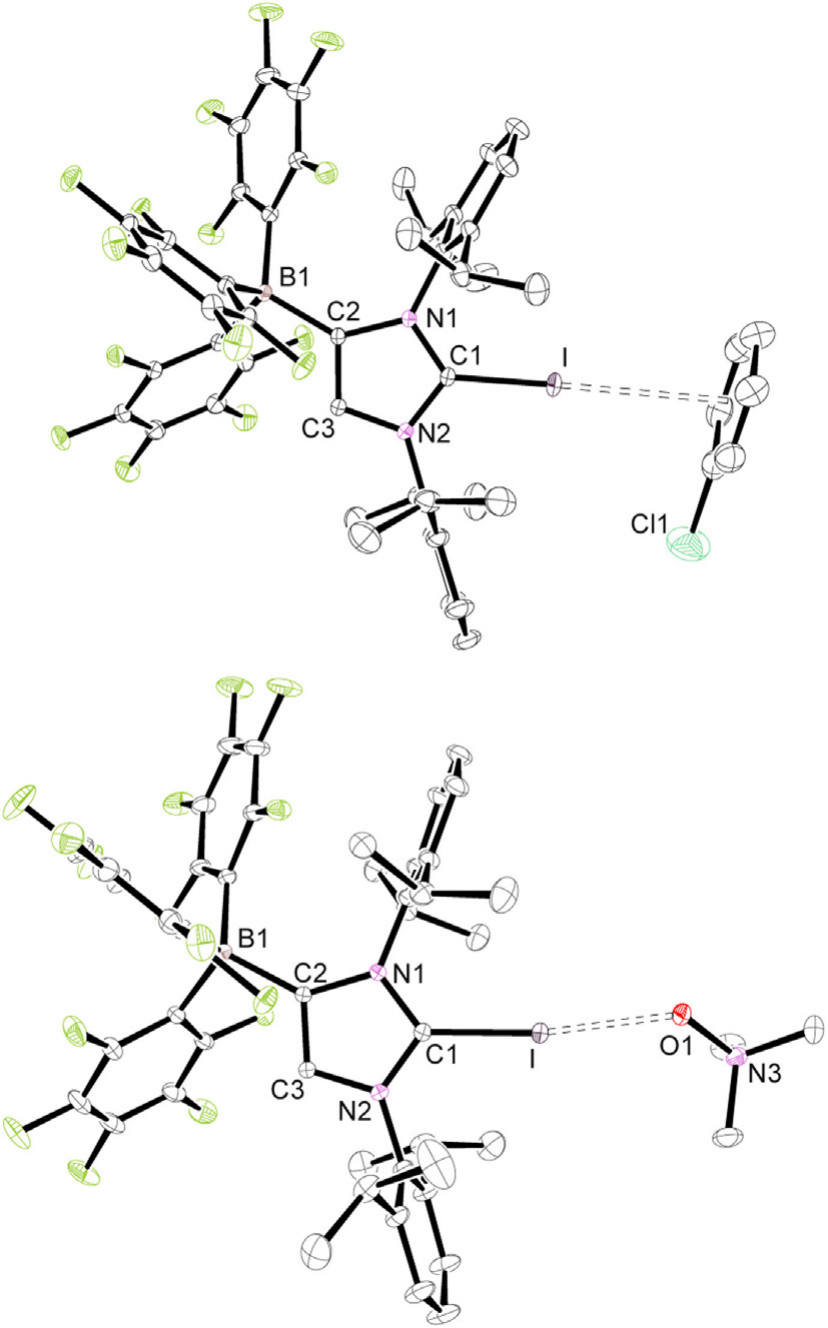
ORTEP diagram of **2 a⋅**C_6_H_5_Cl and **2 a⋅**ONMe_3_ with thermal displacement parameters drawn at the 50 % probability level; hydrogen atoms are omitted for clarity. Pertinent structural data of all compounds **2** are assembled in Table 1.

**Figure 2:**
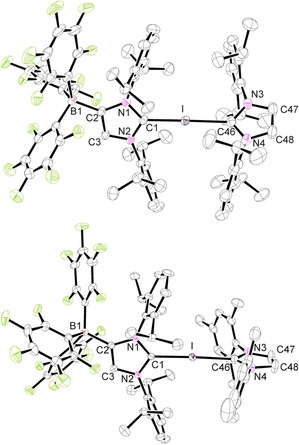
ORTEP diagrams of **5 a⋅**C_6_H_5_Cl and **5 b⋅**C_6_H_5_Cl**⋅** *n*‐hexane with thermal displacement parameters drawn at the 50 % probability level; hydrogen atoms and solvent molecules are omitted for clarity. **5 a** must be regarded with care, owing to the usage of SQUEZZE. Pertinent structural data of all compounds **5** are assembled in Table 2.

Crystallization of **2 a** by layering chlorobenzene, toluene, acetonitrile/chlorobenzene, or THF solutions with *n*‐hexane afforded single crystals of the solvates **2 a⋅**C_6_H_5_Cl, **2 a⋅**C_6_H_5_Me, **2 a⋅**2 CH_3_CN**⋅**C_6_H_5_Cl, and **2 a⋅**THF, respectively. The molecular structure of **2 a⋅**C_6_H_5_Cl is shown in Figure 1, whereas presentations of the other solvates can be found in the Supporting Information (Figures S2, S3, and S5). The crystal structures reveal in all cases a charge‐transfer interaction with a solvent molecule;^[36]^ in the chlorobenzene and toluene complexes, several C_arene_−I distances can be found, which clearly fall below the sum of the van der Waals (vdW) radii (3.68 Å).^[37]^ The bonding of the arene rings is asymmetric with the C_arene_−I distances ranging from 3.4639(17)–4.0083(19) Å in **2 a⋅**C_6_H_5_Cl and 3.367(2)–3.971(2) Å in **2 a⋅**C_6_H_5_Me. The iodine–centroid distances are 3.4814(9) and 3.41715(4) Å and generate C‐I‐centroid angles of 171.01(3)° and 170.28(6)°. Similar structural features were, for instance, found for the C−I⋅⋅⋅π interactions in arene complexes of 1,4‐diiodotetrafluorobenzene^[38]^ and benzene complexes of trifluoroiodomethane (CF_3_I).^[39]^ In the mixed solvate **2 a⋅**2 CH_3_CN**⋅**C_6_H_5_Cl, one acetonitrile ligand interacts with the iodine atom, affording an iodine–nitrogen contact of 2.9156(17) Å, far below the sum of the vdW radii (3.53 Å), and a C‐I‐N angle of 171.71(6)°. Likewise, the THF ligand in **2 a⋅**THF generates an iodine–oxygen contact of 2.743(2) Å, much shorter than the sum of the vdW radii (3.50 Å), and a C‐I‐O angle of 172.45(7)°. Surprisingly, crystallographic evidence for CH_3_CN or THF binding to iodine is rare,^[40]^ and the structural characterization of a THF complex of *N*‐iodosaccharin provides the only closely related example with an even shorter I−O distance of 2.512(2) Å and an N‐I‐O angle of 178.51(9)°.^[41]^ We have also isolated the trimethylamine *N*‐oxide complex **2 a⋅**ONMe_3_ during the attempt to oxidize **2 a** to an iodosyl (IO) derivative. However, **2 a** resisted oxidation, and the molecular structure of **2 a⋅**ONMe_3_ could be established by X‐ray diffraction analysis (Figure 2). Compared with **2 a⋅**THF, a similar C‐I‐O angle of 171.68(3)° is found, whereas the I−O distance of 2.5343(9) Å is significantly shorter. This stronger interaction is also reflected by a slight elongation of the carbon–iodine bond, which increases from 2.0568(8), 2.0601(13), 2.0637(12), and 2.065(2) Å in the chlorobenzene, toluene, acetonitrile, and THF adducts to 2.1090(8) Å in **2 a⋅**ONMe_3_ (Table 1).

**Table 1:** Selected bond lengths [Å] and angles [°] of 2‐halogenoimidazolium borates and calculated reaction enthalpies (Δ*H*_298K_) for adduct formation with **2 a**.

Compound	X	C1−X [Å]	X⋅⋅⋅Y [Å]^[b]^	C1−N1 [Å]	C1−N2 [Å]	N1‐C1‐N2 [°]	C‐I⋅⋅⋅Y [°]^[c]^	Δ*H*_298K_ [kcal mol^−1^]^[c]^
**2 a⋅**C_6_H_5_Cl	I	2.0568(8)	3.4814(9)	1.3491(10)	1.3351(11)	108.50(7)	171.01(3)	−7.5
**2 a⋅**C_6_H_5_Me	I	2.0601(13)	3.41715(4)	1.3477(16)	1.3347(17)	108.37(11)	170.28(6)	−9.3
**2 a⋅**CH_3_CN^[a]^	I	2.0637(12)	2.9156(17)	1.3532(15)	1.4180(15)	108.16(10)	171.71(6)	−5.7
**2 a⋅**THF	I	2.065(2)	2.743(2)	1.351(3)	1.335(3)	108.4(2)	172.45(7)	−10.8
**2 a⋅**ONMe_3_	I	2.1090(8)	2.5343(9)	1.3544(10)	1.3373(10)	107.32(7)	171.68(3)	−17.5
**2 b⋅**C_6_H_5_Me	I	2.0536(13)	–	1.3458(16)	1.3340(16)	108.51(11)	–	–
**2 c**	I	2.0544(16)	–	1.347(2)	1.337(2)	108.43(14)	–	–
**3 a**	Br	1.845(2)	–	1.344(3)	1.323(3)	110.22(17)	–	–
**3 b**	Br	1.8396(15)	–	1.3388(19)	1.3317(19)	109.47(13)	–	–
**4**	Cl	1.6825(13)	–	1.3406(16)	1.3230(16)	110.24(11)	–	–

[a] In the solvate **2 a⋅**2 CH_3_CN**⋅**C_6_H_5_Cl. [b] All listed contacts are below the sum of the van der Waals radii; for **2 a⋅**C_6_H_5_Cl and **2 a⋅**C_6_H_5_Me, Y represents the centroid of the benzene ring. [c] At the B97‐D/6–311G(d,p) level of theory with a quasi‐relativistic basis set “Stuttgart‐Koeln RLC ECP” (46MWB) for iodine.

The molecular structures of the 2‐iodoimidazolium borates **2 b** and **2 c** were also determined by X‐ray diffraction analysis; **2 b** crystallized from a toluene/*n*‐hexane solution as the toluene solvate **2 b⋅**C_6_H_5_Me, whereas **2 c** crystallized without the inclusion of solvent molecules from the THF/*n*‐hexane solution. In contrast to **2 a⋅**C_6_H_5_Me, the toluene solvate molecule in **2 b⋅**C_6_H_5_Me does not interact with the iodine atom, which instead displays short intermolecular C_arene_⋅⋅⋅I contacts of 3.1742(13), 3.4860(15), and 3.5277(1) Å to the 2,4,6‐trimethylphenyl (Mes) group of another molecule of **2 b**, resulting in a head‐to‐head linkage of two molecules in the crystal structure. Likewise, **2 c** forms dimeric units through two intermolecular C_arene_⋅⋅⋅I contacts of 3.338(2) and 3.390(2) Å involving one of the 2,6‐diisopropylphenyl (Dipp) substituents. Packing diagrams can be found in the Supporting Information (Figures S7 and S9). The covalent carbon–iodine bond lengths are 2.0536(13) Å in **2 b⋅**C_6_H_5_Me and 2.0544(16) Å in **2 c** and identical with 2.0568(8) Å established for **2 a⋅**C_6_H_5_Cl (Table 1).

The 2‐bromoimidazolium borates **3 a** and **3 b** were prepared in a similar fashion by reaction of **1 a** and **1 b** with elemental bromine in chlorobenzene solution, which afforded both compounds as colorless solids in high yield (**3 a**: 74 %, **3 b**: 78 %). For the preparation of the corresponding 2‐chloroimidazolium borate **4**, CCl_4_ was used as the chlorination reagent, and its reaction with **1 a** in chlorobenzene solution furnished **4** as a colorless solid in moderate yield (47 %, Scheme 2). It should be noted that the reaction of IDipp and IMes with CCl_4_, however, gives stable 4,5‐dichloroimidazolin‐2‐ylidenes.[^42^, ^43^] All complexes **3** and **4** were characterized by ^1^H, ^11^B{^1^H}, ^13^C{^1^H}, and ^19^F{^1^H} NMR spectroscopy in [D_8_]THF solution (see the Supporting Information for the presentation of all spectra). The NMR spectra are like those recorded for the iodine derivatives **2 a** and **2 b**, except for the ^13^C NMR signals of the carbene carbon atoms, which are found at lower field. Single crystals of **3 a**, **3 b**, and **4⋅**THF were subjected to X‐ray diffraction analysis; the molecular structures are presented in the Supporting Information (Figures S10, S12, and S14), whereas pertinent structural data are assembled in Table 1. The bromine atom in **3 a** shows only conventional Br⋅⋅⋅F and Br⋅⋅⋅H van der Waals contacts in the solid state, **3 b** exhibits intermolecular C_arene_⋅⋅⋅Br contacts of 3.3925(17) and 3.4470(17) Å, which are just below the sum of the van der Waals radii (3.53 Å).^[37]^ The chlorine atom in **4⋅**THF does not interact with the THF solvate molecule, but shows a Cl⋅⋅⋅Cl contact of 3.2665(8) Å, which is slightly shorter than twice the van der Waals radius (3.50 Å).^[37]^ The C−Br bond lengths of 1.845(2) Å (**3 a**) and 1.8396(15) Å (**3 b**) as well as the C−Cl bond length of 1.6825(13) Å in **4⋅**THF fall in the expected ranges and are in good agreement with the values reported for other 2‐bromo‐ and 2‐chloroimidazolium salts.[^44^, ^45^]

### Synthesis and characterization of bis(carbene)halogen(I) complexes

**Scheme 3:**
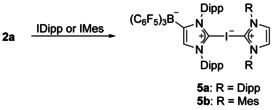
Synthesis of neutral bis(carbene)iodine(I) complexes. Dipp=2,6‐diisopropylphenyl, Mes=2,4,6‐trimethylphenyl.

The strong tendency of the 2‐iodoimidazolium borate **2 a** to act as a halogen bond donor by formation of the adducts **2 a⋅**L (L=C_6_H_5_Cl, C_6_H_5_Me, CH_3_CN, THF, ONMe_3_) prompted us to react **2 a⋅**C_6_H_5_Cl with the neutral carbenes IDipp and IMes to generate hypervalent bis(carbene)iodine(I) complexes (Scheme 3). Layering a chlorobenzene solution containing a 1:1 mixture of **2 a⋅**C_6_H_5_Cl and the respective NHC ligand with *n*‐hexane gave suitable single crystals of **2 a⋅**IDipp (**5 a**) and **2 a⋅**IMes (**5 b**). The structural parameters of **5 a** have to be handled with care owing to the usage of SQUEZZE.^[46]^ The molecular structures are shown in Figure 2, and pertinent structural data are listed in Table 2. The central C1‐I‐C46 unit is in both cases almost linear (**5 a**: 177.02(7)°, **5 b**: 177.44(8)°) with slightly different carbon–iodine bond lengths of 2.260(2)/2.441(2) Å in **5 a** and 2.288(2)/2.361(2) Å in **5 b**. The shorter C−I bonds are formed with the WCA‐NHC ligand, implying that this ligand is a slightly stronger donor in comparison with the neutral NHC. However, they are significantly longer than the C−I bond length of 2.0568(8) Å in **2 a⋅**C_6_H_5_Cl, and this elongation can be attributed to the strong and partly covalent interaction with the neutral NHC ligands, in accordance with a hypervalent 10‐I‐2 bonding situation. The N‐C‐N angles lie between the values of the **2 a** adducts (Table 1) and the neutral carbenes IDipp and IMes (101.4°).[^12^, ^43^] Overall, the structural parameters agree perfectly with those determined for the cationic congeners [(IMes)_2_I][BPh_4_] (**III a**) and [(IDipp)_2_I]I (**III b**).[^24^, ^27^] In the ^13^C NMR spectra ([D_8_]THF solution), two signals at 170.6/156.5 ppm (**5 a**) and 162.9/150.8 ppm (**5 b**) can be assigned to the carbene carbon atoms of the NHC and WCA‐NHC ligands, respectively, revealing a weaker donor and therefore more pronounced carbene character for the neutral NHC ligand as also observed in the solid state (Table 2).

**Table 2:** Selected bond lengths [Å] and angles [°] of homoleptic and heteroleptic bis(carbene)iodine(I) and ‐bromine(I) complexes.

Parameter	**5 a⋅**C_6_H_5_Cl	**5 b⋅**C_6_H_5_Cl**⋅** *n*‐hexane	**7 a⋅**2 THF	**7 b**	**8⋅**C_6_H_5_Cl
X	I	I	I	I	Br
C1−X	2.260(2)	2.288(2)	2.3373(11)	2.4055(17)/2.4089(17)	1.9010(18)
C46−X	2.441(2)	2.361(2)	2.4018(12)	2.2542(17)/2.2575(17)	2.822(2)
C1−N1	1.364(3)	1.355(3)	1.3637(14)	1.356(2)/1.355(2)	1.351(2)
C1−N2	1.336(3)	1.339(3)	1.3415(15)	1.343(2)/1.339(2)	1.341(2)
C46−N3	1.350(3)	1.383(3)	1.3609(16)	1.347(2)/1.348(2)	1.375(2)
C46−N4	1.358(3)	1.346(3)	1.3448(15)	1.338(2)/1.342(2)	1.355(2)
N1‐C1‐N2	106.15(18)	106.07(17)	105.25(10)	105.57(15)/105.22(15)	107.48(15)
N3‐C46‐N4	104.1(2)	105.2(2)	104.85(10)	106.38(14)/106.01(14)	102.50(16)
C1‐X‐C46	177.02(7)	177.44(8)	178.48(4)	178.71(6)/179.32(6)	177.40(6)
Interplanar angle	49.70(9)	92.78(10)	42.58(5)	53.13(7)/48.58(7)	35.65(8)

**Scheme 4:**
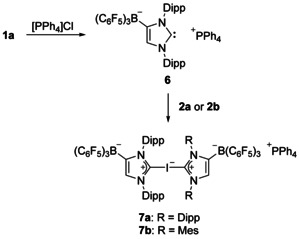
Synthesis of anionic bis(carbene)iodine(I) complexes. Dipp=2,6‐diisopropylphenyl, Mes=2,4,6‐trimethylphenyl.

Attempts to combine **2 a** with the sterically more demanding NHC 1,4‐di‐*tert*‐butylimidazolin‐2‐ylidene (I*t*Bu) failed, and instead of **2 a⋅**I*t*Bu, crystals of the imidazolium salt [I*t*BuH][(WCA‐IDipp)_2_I]**⋅**5 THF were obtained. In the anion, iodine is symmetrically flanked by two WCA‐IDipp ligands with C‐I‐C=178.67(7)° and C−I=2.365(2)/2.378(2) Å (see the Supporting Information, Figure S26). The formation of this compound indicates a high stability of the bis(carbene)iodine(I) anion in analogy with the bis(pentafluorophenyl)iodate(I) **I** (Scheme 1). Therefore, we aimed at the targeted synthesis of this anion in the following. In principle, the combination of **1 a** and **2 a** would lead to a corresponding lithium salt; however, to avoid interaction with the lithium ion, the phosphonium salt [PPh_4_][WCA‐IDipp] (**6**) was prepared by reacting the lithiocarbene **1 a** with tetraphenylphosphonium chloride in chlorobenzene (Scheme 4). Compound **6** was isolated as a pale‐orange solid in satisfactory yield (70 %) after filtration through Celite^®^ and washing with toluene and *n*‐hexane. Naturally, the NMR spectroscopic data of **6** in [D_8_]THF largely correspond to those of the lithiocarbene precursor **1 a**; the ^13^C NMR resonance of the carbene carbon atom is found at 218.8 ppm, which is in the range of **1 a** (217.4 ppm).^[33]^ In addition, the expected signals for the [PPh_4_]^+^ counterion are observed, for example, a singlet at 23.8 ppm in the ^31^P{^1^H} NMR spectrum.

**Figure 3:**
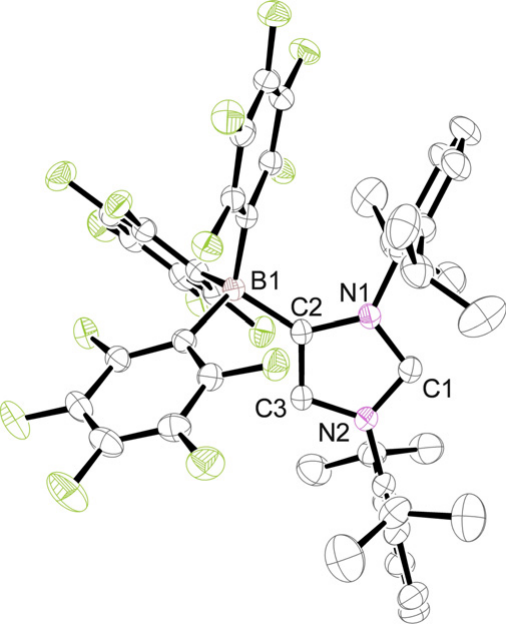
ORTEP diagram of **6** with thermal displacement parameters drawn at the 50 % probability level; the PPh_4_^+^ counterion and the hydrogen atoms are omitted for clarity. Selected bond lengths [Å] and angles [°]: C1−N1 1.3739(18), C1−N2 1.3586(19), N1−C2 1.4164(17), C2−C3 1.3512(19), C3−N2 1.3853(17), C2−B1 1.6416(19); N1‐C1‐N2 101.93(11), C1‐N1‐C2 113.89(11), N1‐C2‐C3 102.97(11), C2‐C3‐N2 109.05(12), C3‐N2‐C1 112.15(11), N1‐C2‐B1 133.29(11), C3‐C2‐B1 123.56(12).

Single crystals of **6** suitable for X‐ray diffraction analysis were obtained by layering a concentrated THF solution with *n*‐hexane, and the molecular structure of the anion is shown in Figure 3. The carbene carbon atom does not show any significant intermolecular contacts and only displays a weak C⋅⋅⋅H contact with one phenyl group of the phosphonium ion. The structural parameters are very similar to those reported for IDipp^[43]^ and also for the lithiocarbene **1 a**,^[34]^ and the small N1‐C1‐N2 angle of 101.93(11)° is in line with the presence of a “free” carbene. A related example is the potassium salt of an amido‐functionalized anionic NHC, in which the carbene and the K^+^ ion are separated by complexation with 18‐crown‐6.^[47]^

The phosphonium salt **6** was treated with **2 a⋅**C_6_H_5_Cl or **2 b** in chlorobenzene solution, furnishing the anionic bis(carbene)iodine(I) complexes **7 a** and **7 b** in moderate yield by crystallization from chlorobenzene or THF/*n*‐hexane solution (Scheme 4). Both compounds were fully characterized by ^1^H, ^11^B{^1^H}, ^13^C{^1^H}, ^19^F{^1^H}, and ^31^P{^1^H} NMR spectroscopy in [D_8_]THF solution (see the Supporting Information for the presentation of all spectra). The symmetric complex **7 a** exhibits only one set of signals for the two equivalent carbene ligands, for example, one singlet for the backbone CH hydrogen atoms at 6.25 ppm, which is intermediate between the chemical shifts of **2 a⋅**C_6_H_5_Cl (7.33 ppm) and **6** (6.11 ppm). In contrast, two singlets at 6.67 and 6.22 ppm are observed for **7 b**. Consequently, one ^13^C NMR resonance is found at 156.2 ppm for **7 a**, whereas two resonances at 164.3 and 150.6 ppm can be assigned to the carbene carbon atoms in **7 b**. The ^31^P{^1^H} NMR spectra of both compounds exhibit one signal at 23.9 ppm for the PPh_4_^+^ ion.

**Figure 4:**
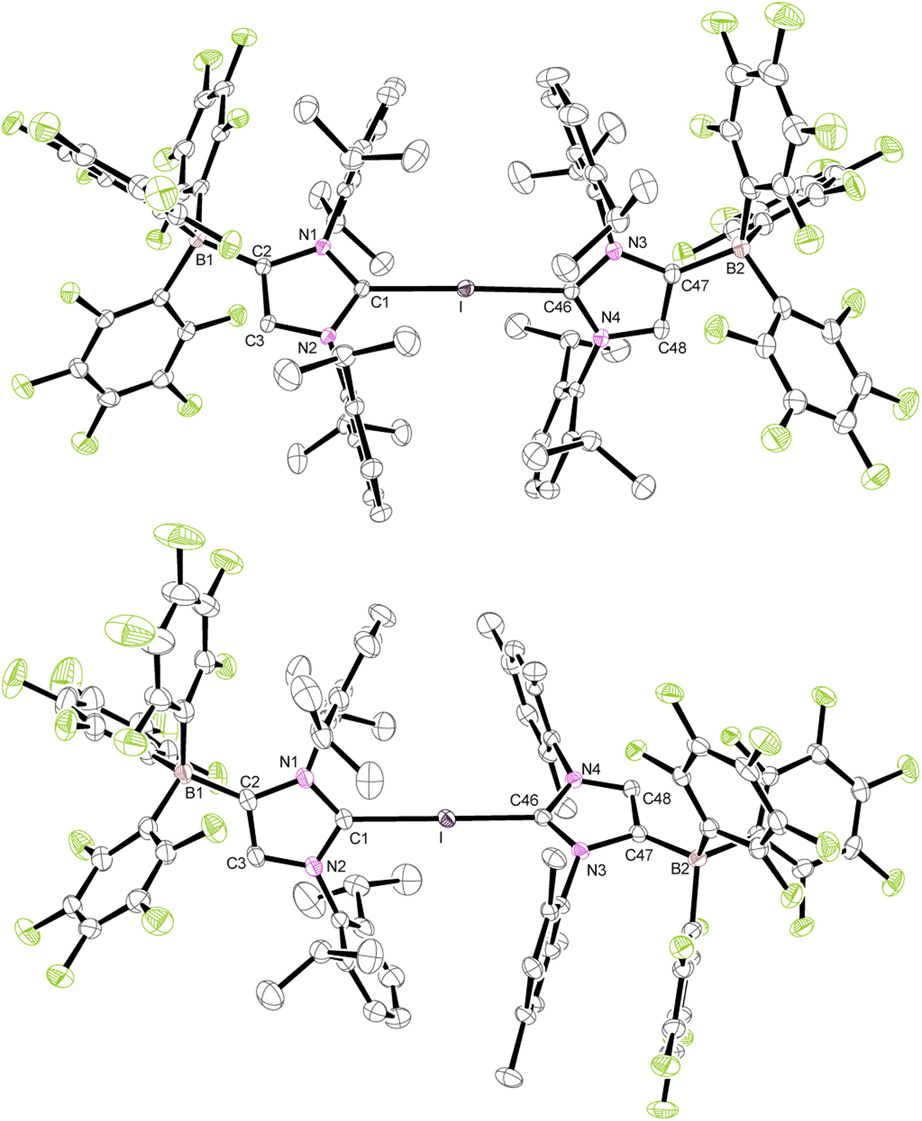
ORTEP diagrams of **7 a⋅**2 THF and **7 b** with thermal displacement parameters drawn at the 50 % probability level; the PPh_4_^+^ counterions, the hydrogen atoms, THF molecules, and a second molecule in the asymmetric unit of **7 b** are omitted for clarity. Pertinent structural data of all compounds **7** are assembled in Table 2.

The crystal structures of **7 a⋅**2 THF and **7 b** were determined by X‐ray diffraction analysis, and Figure 4 shows the ORTEP diagrams of the bis(carbene)iodine(I) anions. Important bond lengths and angles are summarized in Table 2. Compound **7 b** crystallizes with two independent ion pairs in the asymmetric unit with similar structural parameters. Both anions show the expected linear C‐I‐C arrangement with angles of 178.48(4)° (**7 a**) and 178.71(6)°/179.32(6)° (**7 b**) with only little asymmetry of the C−I bond lengths, viz. 2.3373(11)/2.4018(12) Å in **7 a** and 2.4055(17)/2.2542(17) Å (molecule 1) and 2.4089(17)/2.2575(17) Å (molecule 2) in **7 b**. There are no significant differences in comparison with the structural features found for the neutral systems **5 a** and **5 b**, except that the WCA‐IMes ligand in **7 b** forms a shorter C−I bond than the WCA‐IDipp ligand, whereas this ligand has the shorter bond in **5 b**.

**Figure 5:**
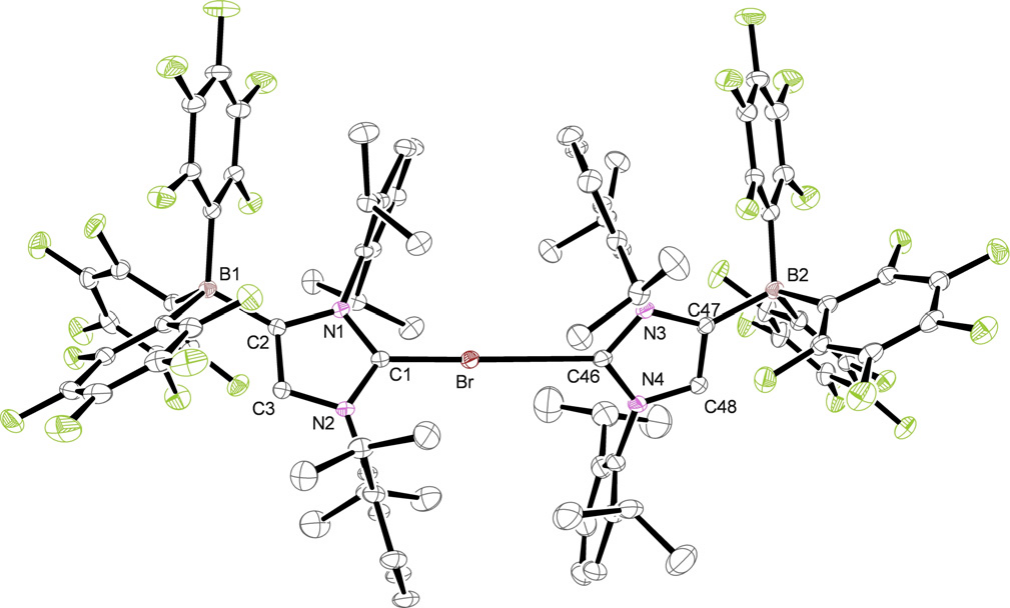
ORTEP diagram of **8⋅**C_6_H_5_Cl with thermal displacement parameters drawn at the 50 % probability level; the PPh_4_^+^ counterion, the hydrogen atoms, and the chlorobenzene molecule are omitted for clarity. Pertinent structural data of all compounds **8⋅**C_6_F_5_Cl are assembled in Table 2.

Attempts to isolate similar bis(carbene)bromine(I) and bis(carbene)chlorine(I) complexes proved difficult, and, for instance, mixing the 2‐bromo‐ and 2‐chloroimidazolium borates **3 a** und **4** with IDipp or IMes led to decomposition and formation of imidazolium salts. However, layering a chlorobenzene solution of **3 a** and **6** with *n*‐hexane furnished colorless single crystals of [PPh_4_][(WCA‐IDipp)_2_Br]**⋅**C_6_H_5_Cl (**8**) suitable for X‐ray diffraction analysis. The molecular structure of the anion in **8** is shown in Figure 5, revealing a linear C‐Br‐C unit with an angle of 177.40(6)°, which unlike the C‐I‐C units in **5** and **7** displays considerably different C−Br bond lengths of 1.9010(18) and 2.822(2) Å. The shorter bond (C1−Br) is noticeably longer compared with 1.845(2) Å in **3 a**, whereas the longer one is still significantly shorter than the sum of the van der Waals radii (3.53 Å).^[37]^ This asymmetry is also expressed in the clearly different N‐C‐N angles of 107.48(15)° and 102.50(16)°, revealing more imidazolium or more carbene character, respectively. Accordingly, the bonding in **8** is better conceived as halogen bonding between **3 a** and the anionic carbene **6**, but this interaction is certainly stronger compared with the Br⋅⋅⋅Br interaction in 2‐bromoimidazolium bromides, which consistently feature shorter C−Br bonds.[^26^, ^27^, ^44^, ^48^] With the exception of bis(pyridine)bromine(I) cations,^[49]^ we are unaware of other bromine systems with a linear L‐Br‐L arrangement. NMR spectroscopic characterization of **8** was hampered by its gradual decomposition in solution. Nevertheless, the NMR spectra in [D_8_]THF reveal the signals for two equivalent WCA‐IDipp ligands, whereas broadening indicates slow bromine exchange between the two carbene ligands on the NMR timescale. Unfortunately, the corresponding chlorine complex could not be isolated by the combination of **4** and **6**, which can be ascribed to its lower thermodynamic stability, resulting in stronger dissociation and faster decomposition in solution. In particular, the zwitterionic imidazolium borates (WCA‐IDipp)H (**9 a**) and (WCA‐IMes)H (**9 b**) crystallized repeatedly as side products and were characterized by X‐ray diffraction analysis (see the Supporting information, Figures S23 and S24).

## Computational Studies

The isolation and crystallization of the solvate complexes **2 a⋅**L (L=C_6_H_5_Cl, C_6_H_5_Me, CH_3_CN, THF) and of the trimethylamine *N*‐oxide complex **2 a⋅**ONMe_3_ prove the ability of the 2‐iodoimidazolium borate **2 a** to serve as a halogen bond donor. To assess the stability of these complexes, their structures were optimized by applying the density functional theory (DFT) method B97‐D.^[50]^ The calculated structural parameters agree with those found experimentally in the solid state by X‐ray diffraction analysis, although the calculated gas‐phase geometries consistently afford slightly longer bond lengths (see the Supporting Information). The resulting association enthalpies Δ*H*_298K_ for the formation of complexes **2 a⋅**L (L=C_6_H_5_Cl, C_6_H_5_Me, CH_3_CN, THF, ONMe_3_) from **2 a** and L are also included in Table 1. For the solvate complexes, the enthalpies range from −5.7 kcal mol^−1^ for L=CH_3_CN to −10.8 kcal mol^−1^ for L=THF, which is typical, but at the stronger end for noncovalent halogen bonding.[^15^, ^16^, ^51^, ^52^] A significantly stronger interaction is calculated for the trimethylamine *N*‐oxide adduct with Δ*H*_298K_=−17.5 kcal mol^−1^, which is on the same order of magnitude as that calculated for *N*‐iodosaccharin pyridine *N*‐oxide complexes.^[53]^ Complexation of **2 a** with the carbenes IDipp and IMes turned out to be more exothermic (Table 3), revealing a significantly higher stability of **2 a⋅**IDipp (**5 a**, −34.5 kcal mol^−1^) and **2 a⋅**IMes (**5 b**, −33.2 kcal mol^−1^). In comparison, isocyanide adducts such as [(C_6_F_5_)I(CNMes)] are significantly more labile with dissociation energies of approximately 7 kcal mol^−1^,^[13]^ which can be ascribed to the higher σ‐donor ability of NHC ligands.

**Table 3:** Calculated Δ*H*_298K_ reaction enthalpies [kcal mol^−1^] for two different DFT methods B97‐D and B3LYP, the latter with and without D3 dispersion correction.^[a,b]^

Compound	Bond length [Å]^[c]^		Δ*H*_298K_ [kcal mol^−1^]			
			B97‐D	B3LYP‐D3	B3LYP	D3
(WCA‐IDipp)I(IDipp) (**5 a**)	2.254	2.552	−34.5	−33.7	−10.0	−23.7
(WCA‐IDipp)I(IMes) (**5 b**)	2.267	2.505	−33.2	−32.3	−12.8	−19.5
[(WCA‐IDipp)_2_I]^−^ (in **7 a**)	2.395	2.385	−50.0	−47.3	−18.9	−28.4
[(WCA‐IDipp)I(IMes‐WCA)]^−^ (in **7 b**)	2.426	2.338	−51.2	−49.9	−28.5	−21.4
[(IDipp)_2_I]^+^ (in **III b**)	2.382	2.382	−47.7	−45.6	−23.2	−22.4
(WCA‐IDipp)Br(IDipp)	1.986	2.624	−27.0	−26.9	−4.1	−22.8
[(WCA‐IDipp)_2_Br]^−^ (in **8**)	2.252	2.260	−41.3	−37.6	−9.5	−28.1
[(IDipp)_2_Br]^+^	2.241	2.241	−37.2	−35.3	−13.6	−21.7
(WCA‐IDipp)Cl(IDipp)	1.821	2.736	−21.1	−22.2	−1.2	−21.0
[(WCA‐IDipp)_2_Cl]^−^	1.766	2.942	−30.6	−29.8	−6.3	−23.5
[(IDipp)_2_Cl]^+^	2.168	2.168	−26.1	−28.3	−6.8	−21.5

[a] Calculated for the reaction of the corresponding 2‐halogenoimidazolium species with the respective NHC. [b] A triple‐ξ basis set 6–311G(d,p) was employed for all main group elements except for the halogen atoms iodine, bromine, and chlorine, for which a quasi‐relativistic basis set “Stuttgart‐Koeln RLC ECP” (46MWB) was applied. [c] Calculated (B97‐D) carbon–halogen bond lengths.

The corresponding anionic dicarbene complexes **7** can be assigned even higher stabilities of −50.0 kcal mol^−1^ (**7 a**) and −51.2 kcal mol^−1^ (**7 b**), which were calculated with respect to **2 a** and the respective WCA‐NHC ligand. Likewise, the formation of the cationic complex [(IDipp)_2_I]^+^ (as in **III a**) from [(IDipp)I]^+^ and IDipp was calculated to be equally exothermic (−47.7 kcal mol^−1^). The same trend was derived for the analogous neutral, anionic, and cationic bis(carbene)bromine(I) and ‐chlorine(I) complexes, with the overall stability decreasing together with the polarizability in the order I>Br>Cl (Table 3). A similar trend has been derived for the energies of heterolytic dissociation of bis(pyridine)halogen(I) cations; it was also found that solvent effects were significant as expected for charged species, with the stability decreasing on moving from the gas to the solution phase.^[54]^ Therefore, our gas‐phase calculations should be considered as the upper limit for the true stabilities, and despite the favorable thermodynamics calculated also for the formation of the corresponding bromine and chlorine species, only the complex [(WCA‐IDipp)_2_Br]^−^ has been obtained as the phosphonium salt **8** so far. We suspect that competing side reactions, especially with the free carbenes in solution, prevent the clean formation of the desired products and afford side products such as the imidazolium borates **9**.

It has been shown that London dispersion decisively contributes to the thermodynamic stability of main‐group element NHC adducts,^[55]^ which has also been demonstrated for pnictogen complexes of anionic N‐heterocyclic carbenes.^[34]^ As similar factors might contribute to the stability of the halogen complexes reported here, the association enthalpies (Δ*H*_298K_) were calculated again for the bis(carbene)halogen(I) complexes employing the B3LYP‐D3^[56]^ and B3LYP^[57]^ levels of theory with and without dispersion correction (Table 3). The corrected DFT results (B3LYP‐D3) are in excellent agreement with those previously discussed for the B97‐D method (see above), whereas significantly lower stabilities were obtained with the uncorrected DFT method B3LYP. The dispersion stabilization (D3) ranges from −21 to −28 kcal mol^−1^, with the highest contribution to the overall stability found for the homoleptic [(WCA‐IDipp)X(WCA‐NHC)]^−^ (X=I, Br, Cl) systems. These results indicate that the WCA–borate moieties clearly enhance the dispersion forces upon adduct formation and therefore enable the isolation of the analogous bromine complex [PPh_4_][(WCA‐IDipp)_2_Br] (**8**). Nevertheless, the calculations suggest that even bis(carbene)chlorine(I) complexes should become isolable if undesired side reactions could be avoided by optimization of the reaction conditions.

**Figure 6:**
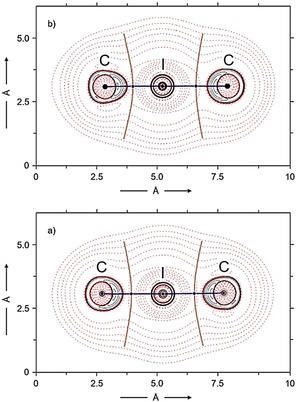
Contour maps of the Laplacian of the electron density, ∇^2^ *ρ*(*r*), along the C‐I‐C axis in [(WCA‐IDipp)I(IDipp)] (**5 a**, top) and [(WCA‐IDipp)_2_I]^−^ (as in **7 a**, bottom). Red dashed lines and solid black lines indicate positive (electronic charge depletion) and negative (electronic charge concentration) ∇^2^ *ρ*(**r**) values, respectively. Solid blue points mark (3, −1) bond critical points (bcp) along the bond path (solid blue line). The solid brown lines separate the atomic basins.

It should be noted that the calculations generally afforded structures with almost identical or very similar carbon−iodine bond lengths for the homoleptic [(WCA‐IDipp)_2_I]^−^ and [(IDipp)_2_I]^+^ systems. Therefore, these complexes should be described as static complexes with a symmetric three‐center, four‐electron (3c4e) bond, in agreement with the NMR spectroscopic results and despite the slight differences found in the crystal structures (cf. Tables 2 and 3). As expected, slightly different carbon−iodine bond lengths were calculated for the heteroleptic congeners [(WCA‐IDipp)I(WCA‐IMes)]^−^, [(WCA‐IDipp)I(IDipp)], and [(WCA‐IDipp)I(IMes)] as also found by X‐ray diffraction analysis, suggesting a similar, but more polarized 3c4e bonding situation.[^7^, ^58^] Accordingly, the bonding situation in two examples, asymmetric [(WCA‐IDipp)I(IDipp)] (**5 a**) and symmetric [(WCA‐IDipp)_2_I]^−^ (as in **7 a**), was further examined by quantum theory of atoms in molecules (QTAIM) analysis,^[59]^ which has previously been used to study the nature of halogen bonding.[^13^, ^51^, ^60^, ^61^] Figure 6 shows the contour map of the Laplacian of the electron density, ∇^2^ *ρ*(*r*), along the C‐I‐C axis in both bis(carbene)iodine(I) complexes, and topological values derived from the QTAIM analysis are assembled in Table 4. For both systems, the two expected (3, −1) bond critical points (bcp) are found along the C−I−C bond paths, which present typical properties of closed‐shell interactions: The value of electron density, *ρ*(*r*_bcp_), is small, and the Laplacian of the electron density, ∇^2^ *ρ*(*r*_bcp_), is positive.[^51^, ^62^] The asymmetric complex [(WCA‐IDipp)I(IDipp)] (**5 a**) features bond critical points with markedly different *ρ*(*r*_bcp_) values of 0.091 and 0.052 *e* *a*_0_^−3^ (*e*=elementary charge, *a*_0_=Bohr radius) in agreement with shorter and stronger binding to the WCA‐IDipp ligand, whereas identical and intermediate *ρ*(*r*_bcp_) values of 0.070 *e* *a*_0_^−3^ together with positive ∇^2^ *ρ*(*r*_bcp_) values (0.096 *e* *a*_0_^−5^) were established for symmetric [(WCA‐IDipp)_2_I]^−^ (as in **7 a**). It is also illustrative to compare these topological values with those associated with the I−C_WCA‐IDipp_ bcp in **2 a**, namely *ρ*(*r*_bcp_)=0.122 *e* *a*_0_^−3^ and ∇^2^ *ρ*(*r*_bcp_)=0.055 *e* *a*_0_^−5^, which would suggest a stronger, but still a closed‐shell carbon–iodine interaction.

**Table 4:** Topological indicators, electron density *ρ*(*r*_bcp_) and Laplacian of the electron density ∇^2^ *ρ*(*r*_bcp_), at the carbon–iodine (3, −1) bond critical points.^[a]^

Compound	Bond	*ρ*(*r*_bcp_) [*e* *a*_0_^−3^]	∇^2^ *ρ*(*r*_bcp_) [*e* *a*_0_^−5^]
(WCA‐IDipp)I (**2 a**)	I−C_WCA‐IDipp_	0.122	0.055
(WCA‐IDipp)I(IDipp) (**5 a**)	I−C_WCA‐IDipp_	0.091	0.092
	I−C_IDipp_	0.052	0.095
[(WCA‐IDipp)_2_I]^−^ (as in **7 a**)	I−C_WCA‐IDipp_	0.070	0.096
	I−C_WCA‐IDipp_	0.070	0.096

[a] Based on the calculated B97‐D geometries, see Table 3; atomic units: *e*=elementary charge, *a*_0_=Bohr radius.

It should be noted that the results of the above topological analysis must be treated with caution^[63]^ and that the naive interpretation as a noncovalent closed‐shell interaction is probably not valid in view of the similar topological values reported for the triiodide anion (I_3_^−^), namely *ρ*(*r*_bcp_)=0.064 *e* *a*_0_^−3^ and ∇^2^ *ρ*(*r*_bcp_)=0.0305 *e* *a*_0_^−5^ for both I−I bond critical points.^[61]^ This anion is a classical textbook example for describing the bonding in a hypervalent (10‐I‐2) system through a 3c4e interaction. Thus, the bonding in the bis(carbene)iodine(I) complexes is probably best conceived as a Class II (L_2_Z) 3c4e interaction,^[8]^ in which the two carbene ligands (L) each provide a pair of electrons and the iodine atom (Z) provides none. This is also in line with the description derived for the bonding in bis(pyridine)iodine(I) systems.[^4^, ^7^] Alternatively, the formation of bis(carbene)iodine(I) complexes can be described by covalent interaction between the σ*‐orbital of the 2‐iodoimidazolium species with the carbene lone pair, and a molecular orbital (MO) diagram of **7 a** in line with this n→σ* bonding model can be found in the Supporting Information (Figure S27).^[9]^ Natural bond orbital (NBO) analysis affords Wiberg bond indices (WBI) of 0.54 for both carbon–iodine bonds in symmetric **7 a**, revealing the expected decrease of the bond order from 1.04 in **2 a**, albeit in agreement with covalent 3c4e bonding.

**Figure 7:**
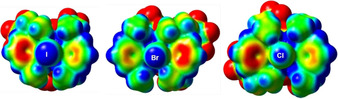
Calculated electrostatic potential (ESP) surface of **2 a** (X=I), **3** (X=Br), and **4** (X=Cl) based on the B97‐D geometries; the blue discs on the surface in the forefront represent the σ‐hole (see Figure S28 in the Supporting Information for further details).

The transition from hypervalency to secondary, noncovalent bonding certainly takes place for the significantly less stable complexes **2 a⋅**L (L=C_6_H_5_Cl, C_6_H_5_Me, CH_3_CN, THF, ONMe_3_),^[9]^ in which the 2‐iodoimidazolium borate **2 a** acts as a halogen bond donor. As noncovalent halogen bonding is often described with the so‐called σ‐hole model,[^15^, ^18^, ^20^, ^22^] we also calculated the electrostatic potential (ESP) surface of **2 a** (Figure 7). Examination of the ESP reveals an area of significant positive potential (in blue) associated with the iodine atom that is available for the directed, linear interaction with the nucleophiles L (C_6_H_5_Cl, C_6_H_5_Me, CH_3_CN, THF, ONMe_3_, IDipp, IMes, WCA‐IDipp, WCA‐IMes) studied in this contribution. For comparison, the ESP of the bromine and chlorine congeners **3** and **4** were also calculated (Figure 7), which qualitatively show less pronounced areas of positive potential, that is, “smaller σ‐holes”, which is in line with these systems acting as weaker halogen bond donors towards nucleophiles such as N‐heterocyclic carbenes, affording lower stabilities for the corresponding bis(carbene)bromine(I) and ‐chlorine(I) complexes (Table 3).

## Conclusion

Zwitterionic 2‐halogenoimidazolium borates of the type (WCA‐NHC)X (X=I, Br, Cl; WCA=weakly coordinating anionic fluoroborate moiety) were obtained from the reactions of the lithium salts [(WCA‐NHC)Li(toluene)] (**1**) with elemental iodine, bromine, or CCl_4_, respectively. Crystallization of the 2‐iodoimidazolium borate (WCA‐IDipp)I (**2 a**, WCA=B(C_6_F_5_)_3_) from different solvents or in the presence of nucleophiles affords several adducts of the type **2 a⋅**L (L=C_6_H_5_Cl, C_6_H_5_Me, CH_3_CN, THF, ONMe_3_), which demonstrates the ability of **2 a** to act as an efficient halogen bond donor, and the calculated association enthalpies suggest weak noncovalent charge‐transfer interactions. In contrast, significantly higher stabilities were calculated for bis(carbene)iodine(I) complexes, and accordingly, the neutral and anionic complexes [(WCA‐IDipp)I(NHC)] (**5**) and [PPh_4_][(WCA‐IDipp)I(WCA‐NHC)] (**7**) could be isolated (NHC=IDipp, IMes), which display short carbon–iodine bond lengths in the solid state in agreement with the presence of more covalent three‐center, four‐electron (3c4e) bonding. The calculations also reveal that London dispersion contributes significantly to the stability of these complexes, which could be exploited for the isolation and structural characterization of the bis(carbene)bromine(I) complex [PPh_4_][(WCA‐IDipp)_2_Br] (**8**) as a rare example of a hypervalent 10‐Br‐2 system.

The experimental and theoretical results also reveal that the anionic carbenes WCA‐NHC act as stronger donors than their neutral NHC congeners, and with the phosphonium salt [PPh_4_][WCA‐IDipp], we have introduced a “free” anionic N‐heterocyclic carbene, which can be employed as a useful synthon in transition metal and main group element chemistry. In addition, we feel that the neutral 2‐iodoimidazolium borates **2**, and potentially also the corresponding bromine derivatives such as **3**, represent useful additions to the family of strong halogen bond donors such as ubiquitous fluoroiodobenzenes, and their application in organic synthesis and catalysis will be further studied by our group.

## Experimental Section

All operations with air‐ and moisture‐sensitive compounds were performed in a glovebox under a dry argon atmosphere (MBraun 200B) or on a vacuum line using Schlenk techniques. All solvents were distilled from Na/benzophenone or CaH_2_, degassed prior to use and stored over molecular sieves (4 Å). The ^1^H, ^13^C{^1^H}, ^11^B{^1^H},^19^F{^1^H}, and ^31^P{^1^H} NMR spectra were recorded with Bruker DPX 200 (200 MHz), Bruker AV 300 (300 MHz), Bruker DRX 400 (400 MHz), and Bruker AVII 600 (600 MHz) devices at room temperature. ^1^H and ^13^C{^1^H} NMR spectra were referenced against the (residual) solvent signals. Boron trifluoride diethyl etherate (BF_3_ **⋅**OEt_2_) was used as an external reference for ^11^B{^1^H}. Trichlorofluoromethane (CFCl_3_) was used as an external reference for ^19^F{^1^H}. Chemical shifts are reported in ppm (parts per million). ^11^B{^1^H}, ^13^C{^1^H}, and ^19^F{^1^H} NMR spectra were obtained by applying composite pulse proton decoupling. Coupling constants (*J*) are reported in Hertz (Hz), and splitting patterns are indicated as s (singlet), d (doublet), t (triplet), q (quartet), m (multiplet), sept (septet), or br (broad). NMR assignments were made by using additional 2D NMR experiments and the position of the aryl signals are indicated by *i* (*ipso*), *o* (*ortho*), *m* (*meta*), and *p* (*para*). Elemental analysis was carried out with a Vario Micro Cube System. Unless otherwise indicated, all starting materials were obtained from Sigma–Aldrich, abcr, TCI, or Acros Organics and were purified if necessary. [(WCA‐IDipp)Li(toluene)] (**1 a**),^[33]^ [(WCA‐IMes)Li(toluene)] (**1 b**),^[32]^ and [(*m*‐XyF_6_)_3_B(IDipp)Li(toluene)] (**1 c**),^[32]^ IDipp^[43]^ and IMes^[43]^ were prepared according to literature procedures. Iodine was sublimed under an inert argon atmosphere and stored inside the glovebox at −40 °C. Tetraphenylphosphonium chloride (PPh_4_Cl) was recrystallized from dichloromethane and dried at 175 °C under high vacuum for 3 days.

### [(WCA‐IDipp)I⋅C_6_H_5_Me] (2 a⋅C_6_H_5_Me)

[(WCA‐IDipp)Li(toluene)] (**1 a**, 100 mg, 0.1 mmol, 1 equiv) was dissolved in THF (3 mL) and stirred at room temperature. Iodine (25.3 mg, 0.1 mmol, 1 equiv) was dissolved in THF (5 mL) and added dropwise to the stirred solution of **1 a**, yielding a discoloration of the iodine solution. The resulting yellow solution was stirred for 1 h and the solvent was removed under high vacuum. The resulting yellow solid was dissolved in toluene (2 mL), filtered through a pad of Celite^®^, and layered with *n*‐hexane. After 24 h, the product **2 a⋅**C_6_H_5_Me could be isolated as colorless crystals (94 mg, 0.084 mmol, 84 %). Elemental analysis calc. (%) for C_52_H_43_BF_15_IN_2_: C 55.83, H 3.87, N 2.50; found: C 55.39, H 3.86, N 2.08; ^1^H NMR (300 MHz, [D_8_]THF): *δ*=7.57 (dd, ^3^ *J*_H,H_=7.72 Hz, ^3^ *J*_H,H_=7.26 Hz, 1 H, *p*‐Dipp), 7.51 (dd, ^3^ *J*_H,H_=7.84 Hz, ^3^ *J*_H,H_=7.84 Hz, 1 H, *p*‐Dipp), 7.41 (d, ^3^ *J*_H,H_=7.73 Hz, 2 H, *m*‐Dipp), 7.37 (s, 1 H, CH=CB), 7.25 (d, ^3^ *J*_H,H_=7.91 Hz, 2 H, *m*‐Dipp), 7.04–7.22 (m, 5 H, toluene), 2.74 (sept, ^3^ *J*_H,H_=6.75 Hz, 2 H, CH(CH_3_)_2_), 2.43 (sept, ^3^ *J*_H,H_=6.92 Hz, 2 H, CH(CH_3_)_2_), 2.30 (s, 3 H, CH_3_‐toluene), 1.30 (d, ^3^ *J*_H,H_=6.80 Hz, 6 H, CH(CH_3_)_2_), 1.26 (d, ^3^ *J*_H,H_=6.73 Hz, 6 H, CH(CH_3_)_2_), 1.16 (d, ^3^ *J*_H,H_=6.94 Hz, 6 H, CH(CH_3_)_2_, 0.91 ppm (d, ^3^ *J*_H,H_=6.41 Hz, 6 H, CH(CH_3_)_2_); ^11^B{^1^H} NMR (96 MHz, [D_8_]THF): *δ*=−15.00 ppm (s); ^19^F{^1^H} NMR (282 MHz, [D_8_]THF): *δ*=−130.1 (br s, 6 F, *o*‐F), −161.2 (m, 3 F, *p*‐F), −166.2 ppm (m, 6 F, *m*‐F).

Further drying afforded the toluene‐free product [(WCA‐IDipp)I] (**2 a**), which was characterized by NMR spectroscopy. ^1^H NMR (500 MHz, [D_8_]THF): *δ*=7.57 (dd, ^3^ *J*_H,H_=7.64 Hz, ^3^ *J*_H,H_=7.93 Hz, 1 H, *p*‐Dipp), 7.51 (dd, ^3^ *J*_H,H_=7.89 Hz, ^3^ *J*_H,H_=7.62 Hz, 1 H, *p*‐Dipp), 7.41 (d, ^3^ *J*_H,H_=7.74 Hz, 2 H, *m*‐Dipp), 7.37 (s, 1 H, CH=CB), 7.25 (d, ^3^ *J*_H,H_=7.74 Hz, 2 H, *m*‐Dipp), 2.74 (sept, ^3^ *J*_H,H_=6.78 Hz, 2 H, CH(CH_3_)_2_), 2.43 (sept, ^3^ *J*_H,H_=6.85 Hz, 2 H, CH(CH_3_)_2_), 1.29 (d, ^3^ *J*_H,H_=6.93 Hz, 6 H, CH(CH_3_)_2_), 1.25 (d, ^3^ *J*_H,H_=6.93 Hz, 6 H, CH(CH_3_)_2_), 1.16 (d, ^3^ *J*_H,H_=6.77 Hz, 6 H, CH(CH_3_)_2_, 0.91 ppm (d, ^3^ *J*_H,H_=6.41 Hz, 6 H, CH(CH_3_)_2_); ^11^B{^1^H} NMR (128 MHz, [D_8_]THF): *δ*=−15.02 ppm (s); ^13^C{^1^H} NMR (125 MHz, [D_8_]THF): *δ*=156.6 (q, CB=CH), 150.7 (m, aryl‐C_6_F_5_), 148.7 (m, aryl‐C_6_F_5_), 147.1 (s, 2×*o*‐Dipp), 146.1 (s, 2×*o*‐Dipp), 140.7 (m, aryl‐C_6_F_5_), 138.8 (m, aryl‐C_6_F_5_), 138.5 (m, aryl‐C_6_F_5_), 136.6 (m, aryl‐C_6_F_5_), 135.9 (s, *i*‐Dipp), 134.8 (m, CH=CB), 133.8 (s, *i*‐Dipp), 132.4 (s, *p*‐Dipp), 132.0 (s, *p*‐Dipp), 125.4 (s, 2×*m*‐Dipp), 125.3 (s, 2×*m*‐Dipp), 104.2 (s, NCN), 29.6 (s, 2×CH(CH_3_)_2_), 28.9 (s, 2×CH(CH_3_)_2_), 25.7 (s, 2×CH(CH_3_)_2_), 25.6 (s, CH(CH_3_)_2_), 24.5 (s, 2×CH(CH_3_)_2_), 23.2 (s, 2×CH(CH_3_)_2_), 23.1 ppm (s, CH(CH_3_)_2_); ^19^F{^1^H} NMR (376 MHz, [D_8_]THF): *δ*=−130.2 (br s, 6 F, *o*‐F), −161.2 (m, 3 F, *p*‐F), −166.3 ppm (s, 6 F, *m*‐F).

### [(WCA‐IDipp)I⋅C_6_H_5_Cl] (2 a⋅C_6_H_5_Cl)

[(WCA‐IDipp)Li(toluene)] (**1 a**, 500 mg, 0.5 mmol, 1 equiv) was suspended in chlorobenzene (5 mL) and stirred at room temperature. Iodine (127 mg, 0.5 mmol, 1 equiv) was dissolved portionwise in chlorobenzene (10 mL) and added dropwise to the stirred solution of **1 a**, yielding in a discoloration of the iodine solution. The resulting yellow solution was stirred for 2 h and the solution was filtered through a pad of Celite^®^. The solvent was removed under high vacuum and the resulting yellowish solid was washed with *n*‐hexane (4 mL). After evaporation of the solvent, the product **2 a⋅**C_6_H_5_Cl was isolated as a colorless solid (472 mg, 0.41 mmol, 82 %). Elemental analysis calc. (%) for C_51_H_43_BClF_15_IN_2_: C 53.78, H 3.54, N 2.46; found: C 53.96, H 3.507, N 2.40; ^1^H NMR (500 MHz, [D_8_]THF): *δ*=7.56 (t, ^3^ *J*_H,H_=7.86 Hz, 1 H, *p*‐Dipp), 7.50 (t, ^3^ *J*_H,H_=7.68 Hz, 1 H, *p*‐Dipp), 7.40 (d, ^3^ *J*_H,H_=7.95 Hz, 2 H, *m*‐Dipp), 7.33 (s, 1 H, CH=CB), 7.24 (d, ^3^ *J*_H,H_=7.81 Hz, 2 H, *m*‐Dipp), 2.74 (sept, ^3^ *J*_H,H_=6.53 Hz, 2 H, CH(CH_3_)_2_), 2.43 (sept, ^3^ *J*_H,H_=6.89 Hz, 2 H, CH(CH_3_)_2_), 1.29 (d, ^3^ *J*_H,H_=6.78 Hz, 6 H, CH(CH_3_)_2_), 1.26 (d, ^3^ *J*_H,H_=6.74 Hz, 6 H, CH(CH_3_)_2_), 1.16 (d, ^3^ *J*_H,H_=6.74 Hz, 6 H, CH(CH_3_)_2_), 0.91 ppm (d, ^3^ *J*_H,H_=6.35 Hz, 6 H, CH(CH_3_)_2_); ^11^B{^1^H} NMR (128 MHz, [D_8_]THF): *δ*=−15.02 ppm (s); ^13^C{^1^H} NMR (125 MHz, [D_8_]THF): *δ*=156.4 (q, ^1^ *J*_C,H_=62.47 Hz, CB=CH), 150.6 (m, aryl‐C_6_F_5_), 148.7 (m, aryl‐C_6_F_5_), 147.1 (s, 2×*o*‐Dipp), 146.1 (s, 2×*o*‐Dipp), 140.7 (m, aryl‐C_6_F_5_), 138.8 (m, aryl‐C_6_F_5_), 138.4 (m, aryl‐C_6_F_5_), 136.5 (m, aryl‐C_6_F_5_), 135.9 (s, Cl‐C and/or *i*‐Dipp), 134.7 (broad s, CH=CB), 133.8 (s, *i*‐Dipp), 132.3 (s, *p*‐Dipp), 131.9 (s, *p*‐Dipp), 130.5 (s, chlorobenzene), 129.1 (s, chlorobenzene), 127.4 (s, chlorobenzene), 125.4 (s, 2×*m*‐Dipp), 125.2 (s, 2×*m*‐Dipp), 105.5 (s, NCN), 29.5 (s, 2×CH(CH_3_)_2_), 28.9 (s, 2×CH(CH_3_)_2_), 25.6 (s, 2×CH(CH_3_)_2_), 24.4 (s, CH(CH_3_)_2_), 24.5 (s, 2×CH(CH_3_)_2_), 23.2 (s, 2×CH(CH_3_)_2_), 23.1 ppm (s, CH(CH_3_)_2_); ^19^F{^1^H} NMR (376 MHz, [D_8_]THF): *δ*=−131.6 (br s, 6 F, *o*‐F), −161.3 (m, 3 F, *p*‐F), −166.3 ppm (s, 6 F, *m*‐F).

### [(WCA‐IDipp)I⋅ONMe_3_] (2 a⋅ONMe_3_)

[(WCA‐IDipp)I**⋅**C_6_H_5_Cl] (**2 a⋅**C_6_H_5_Cl, 50 mg, 0.043 mmol, 1 equiv) and trimethyl *N*‐oxide (3.3 mg, 0.043 mmol, 1 equiv) were dissolved in chlorobenzene (5 mL) and stirred for 2 h at room temperature. The reaction mixture was concentrated to a minimum and layered with *n*‐hexane. After 24 h, the product **2 a⋅**ONMe_3_ was isolated as colorless crystals (34 mg, 0.03 mmol, 71 %). Elemental analysis calc. (%) for C_48_H_44_BF_15_IN_3_O**⋅**0.5 *n*‐hexane=C_51_H_51_BF_15_IN_3_O: C 53.51, H 4.49, N 3.67; found: C 53.16, H 4.964, N 3.21; ^1^H NMR (500 MHz, [D_8_]THF): *δ*=7.50 (t, ^3^ *J*_H,H_=7.81 Hz, 1 H, *p*‐Dipp), 7.44 (t, ^3^ *J*_H,H_=7.94 Hz, 1 H, *p*‐Dipp), 7.35 (d, ^3^ *J*_H,H_=7.89 Hz, 2 H, *m*‐Dipp), 7.19 (d, ^3^ *J*_H,H_=7.75 Hz, 2 H, *m*‐Dipp), 7.06 (s, 1 H, CH=CB), 2.84 (s, 9 H, ONMe_3_), 2.78 (sept, ^3^ *J*_H,H_=6.72 Hz, 2 H, CH(CH_3_)_2_), 2.48 (sept, ^3^ *J*_H,H_=7.03 Hz, 2 H, CH(CH_3_)_2_), 1.29 (m, 12 H, CH(CH_3_)_2_), 1.13 (d, ^3^ *J*_H,H_=6.80 Hz, 6 H, CH(CH_3_)_2_), 0.89 ppm (m, 6 H, CH(CH_3_)_2_); ^11^B{^1^H} NMR (128 MHz, [D_8_]THF): *δ*=−15.14 ppm (s); ^13^C{^1^H} NMR (125 MHz, [D_8_]THF): *δ*=154.0 (q, ^1^ *J*_C,H_=60.35 Hz, CB=CH), 150.6 (m, aryl‐C_6_F_5_), 148.7 (m, aryl‐C_6_F_5_), 147.2 (s, 2×*o*‐Dipp), 146.2 (s, 2×*o*‐Dipp), 140.6 (m, aryl‐C_6_F_5_), 138.6 (m, aryl‐C_6_F_5_), 136.5 (m+s, aryl‐C_6_F_5_+*i*‐Dipp), 134.4 (s, *i*‐Dipp), 133.2 (br s, CH=CB), 131.7 (s, *p*‐Dipp), 131.3 (s, *p*‐Dipp), 125.0 (s, 2×*m*‐Dipp), 124.8 (s, 2×*m*‐Dipp), 117.2 (s, NCN), 59.9 (s, ONMe_3_), 29.4 (s, 2×CH(CH_3_)_2_), 28.8 (s, 2×CH(CH_3_)_2_), 25.8 (s, 2×CH(CH_3_)_2_), 25.6 (s, CH(CH_3_)_2_), 24.5 (s, 2×CH(CH_3_)_2_), 23.1 ppm (s, 2×CH(CH_3_)_2_); ^19^F{^1^H} NMR (376 MHz, [D_8_]THF): *δ*=−130.5 (br s, 6 F, *o*‐F), −161.7 (m, 3 F, *p*‐F), −166.6 ppm (s, 6 F, *m*‐F).

### [(WCA‐IMes)I] (2 b)

[(WCA‐IMes)Li(toluene)] (**1 b**, 500 mg, 0.54 mmol, 1 equiv) was suspended in chlorobenzene (5 mL) and stirred at room temperature. Iodine (138 mg, 0.5 mmol, 1 equiv) was dissolved portionwise in chlorobenzene (10 mL) and added dropwise to the stirred solution of **1 b**, yielding a discoloration of the iodine solution. The resulting yellow solution was stirred for 2 h and the solution was filtered through a pad of Celite^®^. The solvent was removed under high vacuum and the resulting yellowish solid was washed with *n*‐hexane (4 mL). After evaporation of the solvent, the product **2 b** was isolated as a colorless solid (438 mg, 0.46 mmol, 86 %). Elemental analysis calc. (%) for C_39_H_23_BF_15_IN_2_: C 49.71, H 2.46, N 2.97; found: C 50.15, H 2.603, N 2.97; ^1^H NMR (500 MHz, [D_8_]THF): *δ*=7.69 (s, 1 H, CH=CB), 7.16 (s, 2 H, aryl CH), 6.78 (s, 2 H, aryl CH), 2.37 (s, 3 H, *p*‐CH_3_), 2.26 (s, 3 H, *p*‐CH_3_), 2.13 (s, 6 H, *o*‐CH_3_), 1.82 ppm (s, 6 H, *o*‐CH_3_); ^11^B{^1^H} NMR (160 MHz, [D_8_]THF): *δ*=−15.96 ppm (s); ^13^C{^1^H} NMR (125 MHz, [D_8_]THF): *δ*=152.7 (q, ^1^ *J*_C‐B_=59.1 Hz, CB=CH), 149.9 (m, aryl‐C_6_F_5_), 148.0 (m, aryl‐C_6_F_5_), 141.8 (s, *p*‐Mes), 140.8 (s, *p*‐Mes), 140.5 (m, aryl‐C_6_F_5_), 138.5 (m, aryl‐C_6_F_5_), 136.3 (m, aryl‐C_6_F_5_), 135.9 (s, 2 C, *o*‐Mes), 135.5 (s, 2 C, *o*‐Mes), 135.2 (s, *i*‐Mes), 134.1 (s, *i*‐Mes), 131.7 (br s, CH=CB), 130.3 (s, 2 C, *m*‐Mes), 129.8 (s, *m*‐Mes), 122.9 (m, *i*‐C_6_F_5_), 102.7 (m, NCN), 20.9 (s, *p*‐CH_3_), 20.5 (s, *p*‐CH_3_), 17.8 (s, 2 C, *o*‐CH_3_), 17.5 ppm (s, 2 C, *o*‐CH_3_); ^19^F{^1^H} NMR (470 MHz, [D_8_]THF): *δ*=−128.5 (br s, 6 F, *o*‐F), −161.8 (m, 3 F, *p*‐F), −166.1 ppm (br s, 6 F, *m*‐F).

### [(*m*‐XyF_6_)_3_B(IDipp)I] (2 c)

[(*m*‐XyF_6_)_3_B(IDipp)Li(toluene)] (**1 c**, 100 mg, 0.087 mmol, 1 equiv) was suspended in chlorobenzene (5 mL) and stirred at room temperature. Iodine (22.3 mg, 0.087 mmol, 1 equiv) was dissolved portionwise in chlorobenzene (10 mL) and added dropwise to the stirred solution of **1 c**, yielding a discoloration of the iodine solution. The resulting yellow solution was stirred for 3 h and the solution was filtered through a pad of Celite^®^. The solvent was removed under high vacuum and the resulting colorless solid was washed with *n*‐hexane (4 mL). After evaporation of the solvent, the product **2 c** was isolated as a colorless solid (65 mg, 0.055 mmol, 64 %). Elemental analysis calc. (%) for C_51_H_44_BF_18_IN_2_: C 52.60, H 3.81, N 2.41; found: C 53.11, H 3.771, N 1.79; ^1^H NMR (500 MHz, [D_8_]THF): *δ*=7.72–7.24 (br s, CH *m*‐XyF_6_), 7.72 (s, 1 H, CH=CB), 7.61 (t, ^3^ *J*_H,H_=8.02 Hz, 1 H, *p*‐Dipp), 7.47 (d, ^3^ *J*_H,H_=7.83 Hz, 2 H, *m*‐Dipp), 7.44 (t, ^3^ *J*_H,H_=7.73 Hz, 1 H, *p*‐Dipp), 7.16 (d, ^3^ *J*_H,H_=7.82 Hz, 2 H, *m*‐Dipp), 2.53 (sept, ^3^ *J*_H,H_=7.00 Hz, 2 H, CH(CH_3_)_2_), 2.34 (sept, ^3^ *J*_H,H_=6.86 Hz, 2 H, CH(CH_3_)_2_), 1.33 (d, ^3^ *J*_H,H_=6.80 Hz, 6 H, CH(CH_3_)_2_), 1.30 (d, ^3^ *J*_H,H_=6.80 Hz, 6 H, CH(CH_3_)_2_), 1.20 (d, ^3^ *J*_H,H_=6.81 Hz, 6 H, CH(CH_3_)_2_, 0.50 ppm (d, ^3^ *J*_H,H_=6.69 Hz, 6 H, CH(CH_3_)_2_); ^11^B{^1^H} NMR (128 MHz, [D_8_]THF): *δ*=−8.04 ppm (s); ^13^C{^1^H} NMR (125 MHz, [D_8_]THF): *δ*=160.0 (m, CB=CH), 157.6 (m, *i*‐C *m*‐XyF_6_), 146.0 (s, 2×*o*‐Dipp), 145.5 (s, 2×*o*‐Dipp), 135.8 (br s, CH *m*‐XyF_6_), 135.7 (br s, *o*‐C *m*‐XyF_6_), 134.6 (s, CH=CB), 133.8 (s, *i*‐Dipp), 132.7 (s, *p*‐Dipp), 132.4 (s, *p*‐Dipp), 129.9 (br s, *m*‐C *m*‐XyF_6_), 126.1 (s, *m*‐C *m*‐XyF_6_), 125.8 (s, 2×*m*‐Dipp), 125.5 (s, 2×*m*‐Dipp), 119.4 (s, *p*‐C *m*‐XyF_6_), 106.2 (s, NCN), 29.9 (s, 2×CH(CH_3_)_2_), 29.6 (s, 2×CH(CH_3_)_2_), 25.2 (s, 2×CH(CH_3_)_2_), 24.5 (s, CH(CH_3_)_2_), 23.4 (s, 2×CH(CH_3_)_2_), 22.5 ppm (s, 2×CH(CH_3_)_2_); ^19^F{^1^H} NMR (376 MHz, [D_8_]THF): *δ*=−62.3 ppm (s, 18 F).

For the synthesis of compounds **3 a** and **3 b**, elemental bromine (0.03 mL) was diluted in chlorobenzene (5 mL) to give a solution containing 20 mg of bromine per milliliter.

### [(WCA‐IDipp)Br] (3 a)

[(WCA‐IDipp)Li(toluene)] (**1 a**, 250 mg, 0.25 mmol, 1 equiv) was suspended in chlorobenzene (5 mL) and stirred at room temperature. Bromine (40 mg, 0.25 mmol, 1 equiv) was added dropwise via the prepared bromine solution (2 mL), yielding a discoloration of the bromine. The resulting suspension was stirred for 2.5 h at room temperature. The orange solution was filtered through a pad of Celite^®^ and the solvent was removed under high vacuum. The resulting yellowish solid was washed with *n*‐hexane (3×1.5 mL) and dried under high vacuum. The product **3 a** was isolated as a colorless solid (182 mg, 0.18 mmol, 74 %). Elemental analysis calc. (%) for C_45_H_35_BBrF_15_N_2_: C 55.18, H 3.60, N 2.86; found: C 55.66, H 3.777, N 2.50; ^1^H NMR (500 MHz, [D_8_]THF): *δ*=7.58 (t, ^3^ *J*_H,H_=7.96 Hz, 1 H, *p*‐Dipp), 7.51 (t, ^3^ *J*_H,H_=7.84 Hz, 1 H, *p*‐Dipp), 7.42 (d, ^3^ *J*_H,H_=7.84 Hz, 2 H, *m*‐Dipp), 7.36 (s, 1 H, CH=CB), 7.25 (d, ^3^ *J*_H,H_=7.82 Hz, 2 H, *m*‐Dipp), 2.76 (sept, ^3^ *J*_H,H_=6.80 Hz, 2 H, CH(CH_3_)_2_), 2.46 (sept, ^3^ *J*_H,H_=6.82 Hz, 2 H, CH(CH_3_)_2_), 1.26 (d, ^3^ *J*_H,H_=6.93 Hz, 6 H, CH(CH_3_)_2_), 1.18 (d, ^3^ *J*_H,H_=6.73 Hz, 6 H, CH(CH_3_)_2_), 1.17 (d, ^3^ *J*_H,H_=6.77 Hz, 6 H, CH(CH_3_)_2_), 0.95 ppm (d, ^3^ *J*_H,H_=6.53 Hz, 6 H, CH(CH_3_)_2_); ^11^B{^1^H} NMR (160 MHz, [D_8_]THF): *δ*=−15.18 ppm (s); ^13^C{^1^H} NMR (125 MHz, [D_8_]THF): *δ*=155.2 (q, ^1^ *J*_C,B_=58.89 Hz, CB=CH), 150.6 (m, aryl‐C_6_F_5_), 148.7 (m, aryl‐C_6_F_5_), 147.1 (s, 2×*o*‐Dipp), 146.1 (s, 2×*o*‐Dipp), 140.8 (m, aryl‐C_6_F_5_), 138.8 (m, aryl‐C_6_F_5_), 138.5 (m, aryl‐C_6_F_5_), 136.5 (m, aryl‐C_6_F_5_), 133.5 (s, *i*‐Dipp), 133.3 (m, CH=CB), 132.6 (s, *i*‐Dipp), 132.3 (s, *p*‐Dipp), 131.7 (s, *p*‐Dipp), 125.5 (s, 2×*m*‐Dipp), 125.1 (s, 2×*m*‐Dipp), 124.4 (s, NCN), 29.6 (s, 2×CH(CH_3_)_2_), 28.9 (s, 2×CH(CH_3_)_2_), 25.6 (s, 2×CH(CH_3_)_2_), 24.1 (s, 2×CH(CH_3_)_2_), 23.4 (s, 2×CH(CH_3_)_2_), 22.7 ppm (s, 2×CH(CH_3_)_2_); ^19^F{^1^H} NMR (470 MHz, [D_8_]THF): *δ*=−128.6 (br s, 6 F, *o*‐F), −161.0 (m, 3 F, *p*‐F), −166.2 pp, (m, 6 F, *m*‐F).

### [(WCA‐IMes)Br] (3 b)

[(WCA‐IMes)Li(toluene)] (**1 b**, 228 mg, 0.25 mmol, 1 equiv) was suspended in chlorobenzene (5 mL) and stirred at room temperature. The bromine (40 mg, 0.25 mmol, 1 equiv) was added dropwise via the synthesized bromine solution (2 mL), yielding a discoloration of the bromine. The resulting suspension was stirred for 2.5 h at room temperature. The orange solution was filtered through a pad of Celite^®^ and the solvent was removed under high vacuum. The resulting yellowish solid was washed with *n*‐hexane (3×1.5 mL) and dried under high vacuum. The product **3 b** was isolated as a colorless solid (175 mg, 0.18 mmol, 78 %). Elemental analysis calc. (%) for C_39_H_23_BBrF_15_N_2_: C 52.32, H 2.59, N 3.13; found: C 52.01, H 3.097, N 3.79; ^1^H NMR (500 MHz, [D_8_]THF): *δ*=7.70 (s, 1 H, CH=CB), 7.18 (s, 2 H, aryl CH), 6.79 (s, 2 H, aryl CH), 2.37 (s, 3 H, *p*‐CH_3_), 2.25 (s, 3 H, *p*‐CH_3_), 2.15 (s, 6 H, *o*‐CH_3_), 1.85 ppm (s, 6 H, *o*‐CH_3_); ^11^B{^1^H} NMR (160 MHz, [D_8_]THF): *δ*=−16.05 ppm (s); ^13^C{^1^H} NMR (125 MHz, [D_8_]THF): *δ*=150.1 (m, CB=CH), 147.8 (m, aryl‐C_6_F_5_), 142.2 (s, *p*‐Mes), 141.5 (s, *p*‐Mes), 140.9 (m, aryl‐C_6_F_5_), 138.4 (m, aryl‐C_6_F_5_), 136.5 (m, aryl‐C_6_F_5_), 135.8 (s, 2 C, *o*‐Mes), 135.6 (s, 2 C, *o*‐Mes), 133.4 (s, *i*‐Mes), 132.2 (s, *i*‐Mes), 130.4 (s, 3 C, CH=CB+*m*‐Mes), 129.9 (s, 2 C, *m*‐Mes), 124.1 (m, NCN), 20.9 (s, *p*‐CH_3_), 20.5 (s, *p*‐CH_3_), 17.4 (s, 2 C, *o*‐CH_3_), 17.3 ppm (s, 2 C, *o*‐CH_3_); ^19^F{^1^H} NMR (470 MHz, [D_8_]THF): *δ*=−128.6 (br s, 6 F, *o*‐F), −161.8 (m, 3 F, *p*‐F), −166.0 ppm (s, 6 F, *m*‐F).

### [(WCA‐IDipp)Cl] (4)

[(WCA‐IDipp)Li(toluene)] (**1 a**, 150 mg, 0.15 mmol,1 equiv) was suspended in chlorobenzene (3 mL) and stirred at room temperature. Tetrachloromethane (CCl_4_, 46 mg, 0.30 mmol, 2 equiv) was added dropwise to the stirred suspension and the resulting mixture was stirred for 2.5 h at room temperature. The resulting brown solution was concentrated and filtered through a pad of Celite^®^. The solvent was removed under high vacuum and the resulting brownish solid was suspended in toluene (2 mL). The solid was filtered off and washed with toluene (5 mL). Further drying afforded the product **4** as a colorless solid (66 mg, 0.07 mmol, 47 %). Elemental analysis calc. (%) for C_45_H_35_BClF_15_N_2_: C 57.81, H 3.77, N 3.00; found: C 58.15, H 3.971, N 2.87; ^1^H NMR (500 MHz, [D_8_]THF): *δ*=7.59 (t, ^3^ *J*_H,H_=7.97 Hz, 1 H, *p*‐Dipp), 7.52 (t, ^3^ *J*_H,H_=7.95 Hz, 1 H, *p*‐Dipp), 7.44 (d, ^3^ *J*_H,H_=7.86 Hz, 2 H, *m*‐Dipp), 7.32 (s, 1 H, CH=CB), 7.25 (d, ^3^ *J*_H,H_=7.82 Hz, 2 H, *m*‐Dipp), 2.78 (sept, ^3^ *J*_H,H_=6.94 Hz, 2 H, CH(CH_3_)_2_), 2.46 (sept, ^3^ *J*_H,H_=6.83 Hz, 2 H, CH(CH_3_)_2_), 1.25 (d, ^3^ *J*_H,H_=6.90 Hz, 6 H, CH(CH_3_)_2_), 1.20 (d, ^3^ *J*_H,H_=6.83 Hz, 6 H, CH(CH_3_)_2_), 1.13 (d, ^3^ *J*_H,H_=6.80 Hz, 6 H, CH(CH_3_)_2_), 0.98 ppm (d, ^3^ *J*_H,H_=6.61 Hz, 6 H, CH(CH_3_)_2_); ^11^B{^1^H} NMR (128 MHz, [D_8_]THF): *δ*=−15.31 ppm (s); ^13^C{^1^H} NMR (125 MHz, [D_8_]THF): *δ*=154.2 (m, CB=CH), 150.8 (m, aryl‐C_6_F_5_), 148.5 (m, aryl‐C_6_F_5_), 147.3 (s, 2×*o*‐Dipp), 146.1 (s, 2×*o*‐Dipp), 141.1 (m, aryl‐C_6_F_5_), 138.6 (m, aryl‐C_6_F_5_), 136.3 (m, aryl‐C_6_F_5_), 132.7 (s, *p*‐Dipp), 132.4 (s, *p*‐Dipp), 131.8 (m, CH=CB+NCN), 130.5 (s, *i*‐Dipp), 130.3 (s, *i*‐Dipp), 125.5 (s, 2×*m*‐Dipp), 125.1 (s, 2×*m*‐Dipp), 29.6 (s, 2×CH(CH_3_)_2_), 28.9 (s, 2×CH(CH_3_)_2_), 25.6 (s, CH(CH_3_)_2_), 25.4 (s, CH(CH_3_)_2_), 25.2 (s, CH(CH_3_)_2_), 24.0 (s, 2×CH(CH_3_)_2_), 23.5 (s, 2×CH(CH_3_)_2_), 22.5 ppm (s, CH(CH_3_)_2_); ^19^F{^1^H} NMR (376 MHz, [D_8_]THF): *δ*=−128.6 (br s, 6 F, *o*‐F), −160.9 (m, 3 F, *p*‐F), −166.1 pm (m, 6 F, *m*‐F).

### [(WCA‐IDipp)I(IDipp)] (5 a)

[(WCA‐IDipp)I**⋅**C_6_H_5_Cl] (**2 a⋅**C_6_H_5_Cl, 50 mg, 0.043 mmol, 1 equiv) and the free carbene IDipp (17.0 mg, 0.043 mmol, 1 equiv) were dissolved in chlorobenzene (1.5 mL) and layered with *n*‐hexane (4 mL). Colorless crystals were obtained by cooling the reaction mixture to −40 °C for 24 h. The supernatant solution was removed, and the crystals were washed with *n*‐hexane. The crystals were dried under high vacuum and afforded the product **5 a** as a colorless solid (30 mg, 0.021 mmol, 49 %). Elemental analysis calc. (%) for C_72_H_71_BF_15_IN_4_ **⋅**0.5 C_6_H_14_: C 61.78, H 5.39, N 3.84; found: C 61.91, H 5.377, N 4.11. Owing to the occurring decomposition in solution leading to the formation of **9 a** and partial dissociation, the assigned NMR signals must be handled with care. ^1^H NMR (500 MHz, [D_8_]THF) with X=H or B: *δ*=7.51 (t, ^3^ *J*_H,H_=7.93 Hz, 1 H, *p*‐Dipp), 7.46 (t, ^3^ *J*_H,H_=7.93 Hz, 2 H, *p*‐Dipp), 7.32 (m, 3 H, *p*‐Dipp+CH=CX), 7.23 (d, ^3^ *J*_H,H_=7.62 Hz, 1 H, *m*‐Dipp), 7.20 (d, ^3^ *J*_H,H_=7.62 Hz, 3 H, *m*‐Dipp), 7.10 (d, ^3^ *J*_H,H_=7.62 Hz, 2 H, *m*‐Dipp), 6.90 (d, ^3^ *J*_H,H_=7.62 Hz, 2 H, *m*‐Dipp), 6.49 (s, 1 H, CH=CX), 2.68–2.55 (m, 2 H, CH(CH_3_)_2_), 2.36–2.20 (m, 6 H, CH(CH_3_)_2_), 1.07 (d, ^3^ *J*_H,H_=7.01 Hz, 6 H, CH(CH_3_)_2_), 1.04 (d, ^3^ *J*_H,H_=6.71 Hz, 3 H, CH(CH_3_)_2_), 0.97 (d, ^3^ *J*_H,H_=6.40 Hz, 6 H, CH(CH_3_)_2_), 0.92–0.81 (m, 24 H, CH(CH_3_)_2_), 0.75 ppm (d, ^3^ *J*_H,H_=6.10 Hz, 6 H, CH(CH_3_)_2_); ^11^B{^1^H} NMR (160 MHz, [D_8_]THF): *δ*=−15.34 ppm (s); ^13^C{^1^H} NMR (125 MHz, [D_8_]THF): *δ* =170.7 (s, NCN), 156.5 (s, WCA‐NCN), 150.5 (m, CB=CH), 148.6 (m, aryl‐C_6_F_5_), 146.7 (s, 2×*o*‐Dipp), 145.7 (s, 2×*o*‐Dipp), 145.6 (s, 2×*o*‐Dipp), 142.6 (m, aryl‐C_6_F_5_), 140.3 (m, aryl‐C_6_F_5_), 138.3 (m, aryl‐C_6_F_5_), 136.4 (m+s, aryl‐C_6_F_5_+*i*‐Dipp), 135.5 (s, *i*‐Dipp), 134.8 (s, *i*‐Dipp), 134.4 (s, *i*‐Dipp), 131.8 (s, CH=CX), 131.4 (s, *p*‐Dipp), 130.8 (s, *p*‐Dipp), 130.7 (s, *p*‐Dipp), 130.5 (s, *p*‐Dipp), 124.9 (s, 2×*m*‐Dipp), 124.6 (s, 2×*m*‐Dipp), 124.3 (s, CH=CX), 124.2 (s, 2×*m*‐Dipp), 124.0 (s, 2×*m*‐Dipp), 29.4 (s, 2×CH(CH_3_)_2_), 29.2 (s, 2×CH(CH_3_)_2_), 28.8 (s, 2×CH(CH_3_)_2_), 28.3 (s, 2×CH(CH_3_)_2_), 26.6 (s, 2×CH(CH_3_)_2_), 26.4 (s, 2×CH(CH_3_)_2_), 24.6 (s, 2×CH(CH_3_)_2_), 24.3 (s, 2×CH(CH_3_)_2_), 24.2 (2×s, 4×CH(CH_3_)_2_), 24.0 (s, 2×CH(CH_3_)_2_), 22.9 ppm (s, 2×CH(CH_3_)_2_); ^19^F{^1^H} NMR (470 MHz, [D_8_]THF): *δ*=−128.6 (br s, 6 F, *o*‐F), −162.5 (m, 3 F, *p*‐F), −166.9 ppm (m, 6 F, *m*‐F).

### [(WCA‐IDipp)I(IMes)] (5 b)

[(WCA‐IDipp)I**⋅**C_6_H_5_Cl] (**2 a⋅**C_6_H_5_Cl, 50 mg, 0.043 mmol, 1 equiv) and the free carbene IMes (13.3 mg, 0.043 mmol, 1 equiv) were dissolved in chlorobenzene (1.5 mL) and layered with *n*‐hexane (4 mL). Colorless crystals were obtained by cooling the reaction mixture to −40 °C for 24 h. The supernatant solution was removed, and the crystals were washed with *n*‐hexane. The crystals were dried under high vacuum and afforded the product **5 b** as a colorless solid (25 mg, 0.018 mmol, 43 %). Elemental analysis calc. (%) for C_66_H_59_BF_15_IN_4_: C 59.56, H 4.47, N 4.21; found: C 59.26, H 4.583, N 4.16; ^1^H NMR (500 MHz, [D_8_]THF): *δ*=7.46–7.32 (m, 2 H, *p*‐Dipp), 7.21 (s, 2 H, CH=CH IMes), 7.14 (d, ^3^ *J*_H,H_=7.77 Hz, 2 H, *m*‐Dipp), 6.96 (d, ^3^ *J*_H,H_=7.73 Hz, 2 H, *m*‐Dipp), 6.89 (s, 4 H, *m*‐Mes), 6.46 (s, 1 H, CH=CB), 2.64 (sept, ^3^ *J*_H,H_=6.70 Hz, 2 H, CH(CH_3_)_2_), 2.40 (s, 6 H, *p*‐CH_3_ Mes), 2.34 (sept, ^3^ *J*_H,H_=6.90 Hz, 2 H, CH(CH_3_)_2_), 1.73 (s, 12 H, *o*‐CH_3_ Mes), 0.99 (d, ^3^ *J*_H,H_=6.80 Hz, 6 H, CH(CH_3_)_2_), 0.94 (d, ^3^ *J*_H,H_=6.85 Hz, 6 H, CH(CH_3_)_2_), 0.86 (d, ^3^ *J*_H,H_=6.80 Hz, 6 H, CH(CH_3_)_2_), 0.77 ppm (d, ^3^ *J*_H,H_=6.55 Hz, 6 H, CH(CH_3_)_2_); ^11^B{^1^H} NMR (160 MHz, [D_8_]THF): *δ*=−15.48 (s), −16.28 ppm (s); ^13^C{^1^H} NMR (125 MHz, [D_8_]THF): *δ*=163.0 (s, NCN‐IMes), 150.8 (s, NCN‐(WCA‐IDipp)), 150.5 (m, CB=CH), 148.6 (m, aryl‐C_6_F_5_), 147.4 (s, *o*‐Dipp+*o*‐Mes), 146.9 (s, *o*‐Dipp+*o*‐Mes), 146.3 (s, *o*‐Dipp+*o*‐Mes), 146.2 (s, *o*‐Dipp+*o*‐Mes), 140.3 (m, aryl‐C_6_F_5_), 139.6 (s, *p*‐Mes), 138.3 (m, aryl‐C_6_F_5_), 137.2 (s, *i*‐Dipp), 136.3 (m, aryl‐C_6_F_5_), 135.1 (s, *i*‐Dipp), 135.1 (s, *i*‐Mes), 134.9 (s, *i*‐Mes), 130.4 (s, *p*‐Dipp+CH=CB), 130.0 (s, *p*‐Dipp+*m*‐Mes), 124.1 (s, *m*‐Dipp+*m*‐Mes), 124.0 (s, *m*‐Dipp), 123.7 (s, *m*‐Mes), 122.9 (s, CH=CH), 28.7 (s, 2×CH(CH_3_)_2_), 28.3 (s, 2×CH(CH_3_)_2_), 25.8 (s, 2×CH(CH_3_)_2_), 24.3 (s, 2×CH(CH_3_)_2_), 23.3 (s, 2×CH(CH_3_)_2_), 23.1 (s, 2×CH(CH_3_)_2_), 21.0 (s, 2×*p*‐CH_3_‐Mes), 17.2 ppm (s, 4×*o*‐CH_3_‐Mes); ^19^F{^1^H} NMR (470 MHz, [D_8_]THF): *δ*=−128.5 (br s, 6 F, *o*‐F), −162.6 (m, 3 F, *p*‐F), −167.1 ppm (br s, 6 F, *m*‐F).

### [PPh_4_][WCA‐IDipp] (6)

[(WCA‐IDipp)Li(toluene)] (**1 a**, 150 mg, 0.15 mmol, 1 equiv) and tetraphenylphosphonium chloride (56 mg, 0.15 mmol, 1 equiv) were suspended in chlorobenzene (3 mL) and stirred at room temperature for 2.5 h. The resulting orange solution was filtered through a pad of Celite^®^. The solvent was removed under high vacuum and the resulting pale‐orange solid was washed with toluene (2 mL) and *n*‐hexane (2 mL). Further drying afforded the product **6** as a pale‐orange solid (130 mg, 0.10 mmol, 70 %). Elemental analysis calc. (%) for C_69_H_55_BF_15_N_2_P: C 66.89, H 4.47, N 2.26; found: C 66.73, H 4.512, N 1.91; ^1^H NMR (400 MHz, [D_8_]THF): *δ*=7.88 (m, 4 H, *p*‐PPh_4_), 7.72 (m, 16 H, *o*‐ and *m*‐PPh_4_), 7.17 (m, 1 H, *p*‐Dipp), 7.08 (m, 2 H, *m*‐Dipp), 7.02 (m, 1 H, *p*‐Dipp), 6.84 (d, ^3^ *J*_H,H_=7.72 Hz, 2 H, *m*‐Dipp), 6.11 (s, 1 H, CH=CB), 3.15 (sept, ^3^ *J*_H,H_=6.35 Hz, 2 H, CH(CH_3_)_2_), 3.06 (sept, ^3^ *J*_H,H_=6.71 Hz, 2 H, CH(CH_3_)_2_), 1.09 (d, ^3^ *J*_H,H_=6.82 Hz, 6 H, CH(CH_3_)_2_), 1.05 (d, ^3^ *J*_H,H_=6.88 Hz, 6 H, CH(CH_3_)_2_), 1.01 (d, ^3^ *J*_H,H_=6.76 Hz, 6 H, CH(CH_3_)_2_), 0.96 ppm (d, ^3^ *J*_H,H_=6.81 Hz, 6 H, CH(CH_3_)_2_); ^11^B{^1^H} NMR (128 MHz, [D_8_]THF): *δ*=−15.34 ppm (s); ^13^C{^1^H} NMR (100 MHz, [D_8_]THF): *δ*=218.8 (s, NCN), 150.7 (m, CB=CH), 148.1 (m+s, aryl‐C_6_F_5_+2×*o*‐Dipp), 147.0 (s, 2×*o*‐Dipp), 146.5 (s, *i*‐Dipp), 140.5 (s, *i*‐Dipp), 139.9 (m, aryl‐C_6_F_5_), 138.2 (m, aryl‐C_6_F_5_), 137.5 (m, aryl‐C_6_F_5_), 136.2 (d, ^4^ *J*_C,P_=2.99 Hz, *p*‐PPh_4_), 135.3 (d, ^2^ *J*_C,P_=10.36 Hz, *o*‐PPh_4_), 131.1 (d, ^3^ *J*_C,P_=12.96 Hz, *m*‐PPh_4_), 128.9 (m, CH=CB), 127.8 (s, *p*‐Dipp), 127.3 (s, *p*‐Dipp), 123.1 (s, 2×*m*‐Dipp), 121.7 (s, 2×*m*‐Dipp), 118.8 (d, ^1^ *J*_C,P_=89.43 Hz, *i*‐PPh_4_), 28.2 (s, 2×CH(CH_3_)_2_), 28.0 (s, 2×CH(CH_3_)_2_), 26.5 (s, 2×CH(CH_3_)_2_), 24.4 (s, 2×CH(CH_3_)_2_), 24.3 (s, 2×CH(CH_3_)_2_), 21.6 ppm (s, 2×CH(CH_3_)_2_); ^19^F{^1^H} NMR (376 MHz, [D_8_]THF): *δ*=−127.8 (br s, 6 F, *o*‐F), −164.7 (m, 3 F, *p*‐F), −168.0 ppm (m, 6 F, *m*‐F); ^31^P{^1^H} NMR (162 MHz, [D_8_]THF): *δ*=23.8 ppm (s).

### [PPh_4_][(WCA‐IDipp)_2_I] (7 a)

[(WCA‐IDipp)Li(toluene)] (**1 a**, 100 mg, 0.10 mmol, 1 equiv) and tetraphenylphosphonium chloride (37.4 mg, 0.10 mmol, 1 equiv) were suspended in chlorobenzene (3 mL) and stirred at room temperature for 2 h. The reaction mixture turned orange. [(WCA‐IDipp)I**⋅**C_6_H_5_Cl] (**2 a⋅**C_6_H_5_Cl, 114 mg, 0.10 mmol, 1 equiv) was dissolved in chlorobenzene (1 mL) and added dropwise to the stirred solution. The resulting mixture became colorless and was stirred for 2.5 h at room temperature. The solution was filtered through a pad of Celite^®^, concentrated to a minimum and layered with *n*‐hexane. After 24 h, the raw product was obtained as grayish crystals, which were washed with toluene (4×2 mL) and *n*‐hexane (2×2 mL). Further drying afforded the product **7 a** as a grayish solid (126 mg, 0.055 mmol, 57 %). Elemental analysis calc. (%) for C_114_H_90_B_2_lF_30_IN_4_P: C 60.44, H 4.00, N 2.47; found: C 60.77, H 4.353, N 2.34; ^1^H NMR (500 MHz, [D_8_]THF): *δ*=7.93 (m, 4 H, *p*‐PPh_4_), 7.77 (m, 16 H, *o*‐ and *m*‐PPh_4_), 7.33 (m, 2 H, *p*‐Dipp), 7.25 (m, 2 H, *p*‐Dipp), 7.05 (d, ^3^ *J*_H,H_=7.73 Hz, 4 H, *m*‐Dipp), 6.85 (d, ^3^ *J*_H,H_=7.70 Hz, 4 H, *m*‐Dipp), 6.25 (s, 2 H, CH=CB), 2.65 (sept, ^3^ *J*_H,H_=6.51 Hz, 4 H, CH(CH_3_)_2_), 2.36 (sept, ^3^ *J*_H,H_=6.51 Hz, 4 H, CH(CH_3_)_2_), 0.91 (d, ^3^ *J*_H,H_=6.61 Hz, 12 H, CH(CH_3_)_2_), 0.87 (d, ^3^ *J*_H,H_=6.74 Hz, 12 H, CH(CH_3_)_2_), 0.80 (d, ^3^ *J*_H,H_=6.90 Hz, 12 H, CH(CH_3_)_2_), 0.73 ppm (d, ^3^ *J*_H,H_=6.51 Hz, 12 H, CH(CH_3_)_2_); ^11^B{^1^H} NMR (160 MHz, [D_8_]THF): *δ*=−15.34 ppm (s); ^13^C{^1^H} NMR (100 MHz, [D_8_]THF): *δ*=156.2 (s, 2×NCN), 150.5 (m, aryl‐C_6_F_5_), 148.6 (m, 2×CB=CH), 146.4 (s, 4×*o*‐Dipp), 145.9 (s, 4×*o*‐Dipp), 140.2 (m, aryl‐C_6_F_5_), 138.2 (m, aryl‐C_6_F_5_), 137.4 (s, 2×*i*‐Dipp), 136.1 (d, ^4^ *J*_C,P_=2.97 Hz, *p*‐PPh_4_), 135.7 (s, 2×*i*‐Dipp), 135.4 (d, ^2^ *J*_C,P_=10.36 Hz, *o*‐PPh_4_), 131.1 (s, 2×CH=CB), 131.1 (d, ^3^ *J*_C,P_=13.25 Hz, *m*‐PPh_4_), 130.1 (s, 2×*p*‐Dipp), 129.7 (s, 2×*p*‐Dipp), 124.4 (s, 4×*m*‐Dipp), 124.0 (s, 4×*m*‐Dipp), 118.9 (d, ^1^ *J*_C,P_=89.71 Hz, *i*‐PPh_4_), 28.6 (s, 4×CH(CH_3_)_2_), 28.2 (s, 4×CH(CH_3_)_2_), 26.8 (s, 4×CH(CH_3_)_2_), 24.8 (s, 4×CH(CH_3_)_2_), 24.4 (s, 4×CH(CH_3_)_2_), 22.7 ppm (s, 4×CH(CH_3_)_2_); ^19^F{^1^H} NMR (470 MHz, [D_8_]THF): *δ*=−128.7 (br s, 6 F, *o*‐F), −163.0 (m, 3 F, *p*‐F), −167.6 ppm (m, 6 F, *m*‐F); ^31^P{^1^H} NMR (202 MHz, [D_8_]THF): *δ*=23.9 ppm.

### [PPh_4_][(WCA‐IDipp)I(WCA‐IMes)] (7 b)

[(WCA‐IDipp)Li(toluene)] (**1 a**, 50 mg, 0.05 mmol, 1 equiv) and tetraphenylphosphonium chloride (18 mg, 0.05 mmol, 1 equiv) were suspended in chlorobenzene (3 mL) and stirred at room temperature for 1 h. The reaction mixture turned orange. [(WCA‐IMes)I] (**2 b**, 47.2 mg, 0.05 mmol, 1 equiv) was dissolved in chlorobenzene (1 mL) and added dropwise to the stirred solution. The resulting mixture became colorless and was stirred for 2.5 h at room temperature. The solution was filtered through a pad of Celite^®^ and the solvent was removed. The resulting solid was stirred and washed with *n*‐hexane (5 mL) and further drying afforded the raw product, which was recrystallized from a concentrated THF solution layered with *n*‐hexane. The product **7 b** was obtained as a colorless solid (74 mg, 0.033 mmol, 67 %). Elemental analysis calc. (%) for C_108_H_78_B_2_F_30_IN_4_P: C 59.47, H 3.60, N 2.57; found: C 59.69, H 3.789, N 2.43; ^1^H NMR (500 MHz, [D_8_]THF): *δ*=7.93 (m, 4 H, *p*‐PPh_4_), 7.77 (m, 16 H, *o*+*m*‐PPh_4_), 7.35 (m, 1 H, *p*‐Dipp), 7.27 (m, 1 H, *p*‐Dipp), 7.08 (d, ^3^ *J*_H,H_=7.42 Hz, 2 H, *m*‐Dipp), 6.90 (d, ^3^ *J*_H,H_=7.62 Hz, 2 H, *m*‐Dipp), 6.84 (s, 2 H, *m*‐Mes), 6.67 (s, 1 H, CH=CB), 6.39 (s, 2 H, *m*‐Mes), 6.22 (s, 1 H, CH=CB), 2.65 (sept, ^3^ *J*_H,H_=6.69 Hz, 2 H, CH(CH_3_)_2_), 2.37 (sept+s, ^3^ *J*_H,H_=7.06 Hz, 2 H, CH(CH_3_)_2_+*p*‐CH_3_ Mes), 2.24 (s, 3 H, *p*‐CH_3_ Mes), 1.77 (s, 6 H, *o*‐CH_3_ Mes), 1.47 (s, 6 H, *o*‐CH_3_ Mes), 0.94 (d, ^3^ *J*_H,H_=6.88 Hz, 6 H, CH(CH_3_)_2_), 0.90 (d, ^3^ *J*_H,H_=6.89 Hz, 6 H, CH(CH_3_)_2_), 0.81 (d, ^3^ *J*_H,H_=6.87 Hz, 6 H, CH(CH_3_)_2_), 0.74 ppm (d, ^3^ *J*_H,H_=6.62 Hz, 6 H, CH(CH_3_)_2_); ^11^B{^1^H} NMR (160 MHz, [D_8_]THF): *δ*=−15.51 (s), −16.28 ppm (s); ^13^C{^1^H} NMR (125 MHz, [D_8_]THF): *δ*=164.3 (s, NCN), 150.6 (s, NCN), 150.4 (m, aryl‐C_6_F_5_), 149.8 (m, aryl‐C_6_F_5_), 148.6 (m, aryl‐C_6_F_5_), 147.9 (m, aryl‐C_6_F_5_), 146.9 (s, *o*‐Dipp), 146.2 (s, *o*‐Dipp), 142.9 (m, aryl‐C_6_F_5_), 140.1 (m, aryl‐C_6_F_5_), 138.8 (s, *p*‐Mes), 138.1 (s, m, aryl‐C_6_F_5_), 137.5 (s, *p*‐Mes), 136.3 (s, *i*‐Dipp), 136.2 (d, ^4^ *J*_C,P_=3.10 Hz, *p*‐PPh_4_), 135.7 (s, *i*‐Dipp), 135.5 (s, *i*‐Mes), 135.4 (d, ^2^ *J*_C,P_=10.31 Hz, *o*‐PPh_4_), 135.3 (s, *i*‐Mes), 131.1 (d, ^3^ *J*_C,P_=12.89 Hz, *m*‐PPh_4_), 129.9 (s, CH=CB), 129.8 (s, *p*‐Dipp+*m*‐Mes), 129.4 (s, *p*‐Dipp), 129.2 (s, *m*‐Mes), 129.1 (s, C_aryl_‐Mes), 126.8 (s, CH=CB), 123.7 (s, 2×*m*‐Dipp), 123.3 (s, 2×*m*‐Dipp), 118.8 (d, ^1^ *J*_C,P_=89.25 Hz, *i*‐PPh_4_), 28.5 (s, 2×CH(CH_3_)_2_), 28.1 (s, 2×CH(CH_3_)_2_), 26.0 (s, 2×CH(CH_3_)_2_), 23.5 (s, 2×CH(CH_3_)_2_), 23.3 (s, 2×CH(CH_3_)_2_), 23.0 (s, 2×CH(CH_3_)_2_), 21.1 (s, *p*‐CH_3_‐Mes), 20.8 (s, *p*‐CH_3_‐Mes), 17.9 (s, 2×*o*‐CH_3_‐Mes), 17.5 ppm (s, 2×*o*‐CH_3_‐Mes); ^19^F{^1^H} NMR (470 MHz, [D_8_]THF): *δ*=−128.6 (br s, 6 F, *o*‐F), −163.2 (m, 3 F, *p*‐F), −163.4 (br s, 6 F, *p*‐F), −167.0 (br s, 6 F, *m*‐F), −167.4 ppm (m, 6 F, *m*‐F); ^31^P{^1^H} NMR (202 MHz, [D_8_]THF): *δ*=23.9 ppm (s).

### [PPh_4_][(WCA‐IDipp)_2_Br] (8)

[(WCA‐IDipp)Br] (**3 a**, 23.7 mg, 0.024 mmol, 1 equiv) and [PPh_4_][WCA‐IDipp] (**6**, 30 mg, 0.024 mmol, 1 equiv) were suspended in toluene (2 mL). Chlorobenzene was added dropwise until the solution became clear. The solution was filtered and layered with *n*‐hexane. Colorless crystals were obtained after 16 h at room temperature. The supernatant solution was removed, and the crystals were washed with *n*‐hexane. The crystals were dried and afforded the product **5 b** as a colorless solid (50 mg, 0.022 mmol, 93 %). Elemental analysis calc. (%) for C_114_H_90_B_2_BrF_30_N_4_P**⋅**C_6_H_5_Cl=C_120_H_95_B_2_BrClF_30_N_4_: C 61.83, H 4.11, N 2.40; found: C 62.34, H 4.494, N 1.73. Owing to the decomposition occurring in solution leading to the formation of **9 a** and partial dissociation, the assigned NMR signals must be handled with care. ^1^H NMR (500 MHz, [D_8_]THF): *δ*=7.93–7.85 (m, 4 H, *p*‐PPh_4_), 7.78–7.70 (m, 16 H, *o*‐ and *m*‐PPh_4_), 7.55–7.01 (m, 12 H, aryl‐Dipp), 6.75 (br s, 2 H, CH=CB), 2.90 (sept, ^3^ *J*_H,H_=6.61 Hz, 4 H, CH(CH_3_)_2_), 2.69 (sept, 4 H, ^3^ *J*_H,H_=6.74 Hz, CH(CH_3_)_2_), 1.17–1.13 (m, 12 H, CH(CH_3_)_2_), 1.10 (d, ^3^ *J*_H,H_=6.77 Hz, 12 H, CH(CH_3_)_2_), 1.08–1.03 (m, 12 H, CH(CH_3_)_2_), 0.94 ppm (d, ^3^ *J*_H,H_=6.77 Hz, 12 H, CH(CH_3_)_2_); ^11^B{^1^H} NMR (128 MHz, [D_8_]THF): *δ*=−15.25 ppm (s); ^13^C{^1^H} NMR (125 MHz, [D_8_]THF): *δ*=150.5 (m, CB=CH), 148.6 (m, aryl‐C_6_F_5_), 147.5 (s, 4×*o*‐Dipp), 146.4 (s, 4×*o*‐Dipp), 140.3 (m, aryl‐C_6_F_5_), 138.3 (m, aryl‐C_6_F_5_), 137.3 (s, 2×*i*‐Dipp), 136.3 (m, aryl‐C_6_F_5_), 136.2 (d, ^4^ *J*_C,P_=2.93 Hz, *p*‐PPh_4_), 135.4 (s, 2×*i*‐Dipp), 135.4 (d, ^2^ *J*_C,P_=10.42 Hz, *o*‐PPh_4_), 132.9 (s, 2×*p*‐Dipp), 131.3 (s, 2×CH=CB), 131.2 (s, 2×*p*‐Dipp), 131.1 (d, ^3^ *J*_C,P_=12.89 Hz, *m*‐PPh_4_), 124.9 (s, 2×*m*‐Dipp), 124.4 (s, 2×*m*‐Dipp), 124.0 (s, 2×*m*‐Dipp), 123.6 (s, 2×*m*‐Dipp), 118.9 (d, ^1^ *J*_C,P_=89.42 Hz, *i*‐PPh_4_), 28.9 (2×s, 4×CH(CH_3_)_2_), 28.6 (s, 4×CH(CH_3_)_2_), 26.4 (s, 4×CH(CH_3_)_2_), 25.9 (s, 4×CH(CH_3_)_2_), 24.2 (s, 4×CH(CH_3_)_2_), 22.2 ppm (s, 4×CH(CH_3_)_2_); ^19^F{^1^H} NMR (470 MHz, [D_8_]THF): *δ*=−128.2 (br s, 12 F, *o*‐F), −162.8 (m, 6 F, *p*‐F), −167.1 ppm (m, 12 F, *m*‐F); ^31^P{^1^H} NMR (202 MHz, [D_8_]THF): *δ*=23.9 ppm.

### Crystal structures

Crystallographic details and presentations of all compounds, including selected packing diagrams, can be found in the Supporting Information.

Deposition numbers 2034606, 2034607, 2034608, 2034609, 2034610, 2034617, 2034618, 2034619, 2034620, 2034621, 20346222, 2034623, 2034624, 2034625, 2034626, 2034628, 2034629, 2034630, 2034631, and 2034632 contain the supplementary crystallographic data for this paper. These data are provided free of charge by the joint Cambridge Crystallographic Data Centre and Fachinformationszentrum Karlsruhe Access Structures service.

### Computational details

Details of the DFT calculations and energies for all optimized structures can be found in the Supporting Information. Coordinates of all structures are available as mol files.

## Conflict of interest

The authors declare no conflict of interest.

## Supporting information

As a service to our authors and readers, this journal provides supporting information supplied by the authors. Such materials are peer reviewed and may be re‐organized for online delivery, but are not copy‐edited or typeset. Technical support issues arising from supporting information (other than missing files) should be addressed to the authors.

Supplementary

## References

[1a] H. Waldmann , Organic Synthesis Highlights II, VCH, Weinheim, 1995;

[1b] P. J. Stang , V. V. Zhdankin , Chem. Rev. 1996, 96, 1123; 11848783 10.1021/cr940424+

[1c] V. V. Zhdankin , P. J. Stang , Chem. Rev. 2002, 102, 2523; 12105935 10.1021/cr010003+

[1d] T. Wirth , Y. Kita , Hypervalent Iodine Chemistry. Modern Developments in Organic Synthesis, Springer, Berlin, 2003;

[1e] T. Wirth , Angew. Chem. Int. Ed. 2005, 44, 3656; 10.1002/anie.200500115 15828037

[1f] V. V. Zhdankin , P. J. Stang , Chem. Rev. 2008, 108, 5299; 18986207 10.1021/cr800332c PMC2736367

[1g] V. V. Zhdankin , Hypervalent Iodine Chemistry, John Wiley & Sons Ltd, Chichester, UK, 2013;

[1h] T. Dohi , Y. Kita , Iodine Chemistry and Applications (Ed.: T. Kaiho ), Wiley, Hoboken, New Jersey, 2015, pp. 103–157;

[1i] T. Wirth , Topics in Current Chemistry, Vol. 373, Springer, Cham, 2016; 10.1007/128_2015_639 26044514

[1j] L. Catalano , G. Cavallo , P. Metrangolo , G. Resnati , G. Terraneo , Top. Curr. Chem. 2016, 373, 289; 26809623 10.1007/128_2015_666

[1k] A. Yoshimura , V. V. Zhdankin , Chem. Rev. 2016, 116, 3328; 26861673 10.1021/acs.chemrev.5b00547

[1l] F. C. Sousa E. Silva , A. F. Tierno , S. E. Wengryniuk , Molecules 2017, 22, 1290; 10.3390/molecules22050780 PMC6154742 28498333

[1m] X. Li , P. Chen , G. Liu , Beilstein J. Org. Chem. 2018, 14, 1813; 30112085 10.3762/bjoc.14.154 PMC6071704

[1n] I. F. D. Hyatt , L. Dave , N. David , K. Kaur , M. Medard , C. Mowdawalla , Org. Biomol. Chem. 2019, 17, 7822. 31372624 10.1039/c9ob01267b

[2] J. C. Martin , Science 1983, 221, 509. 17830935 10.1126/science.221.4610.509

[3a] K. Sonnenberg , L. Mann , F. A. Redeker , B. Schmidt , S. Riedel , Angew. Chem. Int. Ed. 2020, 59, 5464; 10.1002/anie.201903197 31090163

[3b] H. Haller , S. Riedel , Z. anorg. allg. Chem. 2014, 640, 1281;

[3c] L. Kloo , Comprehensive Inorganic Chemistry II, From Elements to Applications (Eds.: J. Reedijk , K. R. Poeppelmeier , A. M. Abakumov , A. V. Shevel′kov , Á. R. Álvarez , E. V. Antipov , J. W. Niemantsverdriet , L. Casella , N. Revaprasadu , P. O'Brien , N. Revaprasadu , S. Kitagawa; R. L. Bedard , L. Casella , R. Schlögl , J. W. Niemantsverdriet , V. W. W. Yam , S. Álvarez ), Elsevier, Amsterdam, 2013, pp. 233–249;

[3d] P. H. Svensson , L. Kloo , Chem. Rev. 2003, 103, 1649. 12744691 10.1021/cr0204101

[4] L. Turunen , M. Erdélyi , Chem. Soc. Rev. 2020, 49, 2688. 32211673 10.1039/d0cs00034e

[5a] S. Lindblad , K. Mehmeti , A. X. Veiga , B. Nekoueishahraki , J. Gräfenstein , M. Erdélyi , J. Am. Chem. Soc. 2018, 140, 13503; 30234293 10.1021/jacs.8b09467 PMC6209183

[5b] A.-C. C. Carlsson , K. Mehmeti , M. Uhrbom , A. Karim , M. Bedin , R. Puttreddy , R. Kleinmaier , A. A. Neverov , B. Nekoueishahraki , J. Gräfenstein , K. Rissanen , M. Erdélyi , J. Am. Chem. Soc. 2016, 138, 9853; 27265247 10.1021/jacs.6b03842 PMC4981895

[5c] M. Bedin , A. Karim , M. Reitti , A.-C. C. Carlsson , F. Topić , M. Cetina , F. Pan , V. Havel , F. Al-Ameri , V. Sindelar , K. Rissanen , J. Gräfenstein , M. Erdélyi , Chem. Sci. 2015, 6, 3746; 29218144 10.1039/c5sc01053e PMC5707496

[5d] A.-C. C. Carlsson , J. Gräfenstein , A. Budnjo , J. L. Laurila , J. Bergquist , A. Karim , R. Kleinmaier , U. Brath , M. Erdélyi , J. Am. Chem. Soc. 2012, 134, 5706. 22384818 10.1021/ja301341h

[6] W. B. Farnham , J. C. Calabrese , J. Am. Chem. Soc. 1986, 108, 2449. 22175602 10.1021/ja00269a055

[7] A. C. Reiersølmoen , S. Battaglia , S. Øien-Ødegaard , A. K. Gupta , A. Fiksdahl , R. Lindh , M. Erdélyi , Chem. Sci. 2020, 11, 7979. 10.1039/d0sc02076a PMC8163095 34094166

[8] M. L. H. Green , G. Parkin , Dalton Trans. 2016, 45, 18784. 27845802 10.1039/c6dt03570a

[9] R. H. Crabtree , Chem. Soc. Rev. 2017, 46, 1720. 28240328 10.1039/c6cs00688d

[10] A. J. Arduengo , R. L. Harlow , M. Kline , J. Am. Chem. Soc. 1991, 113, 361.

[11] A. J. Arduengo , M. Kline , J. C. Calabrese , F. Davidson , J. Am. Chem. Soc. 1991, 113, 9704.

[12] A. J. Arduengo , H. V. R. Dias , R. L. Harlow , M. Kline , J. Am. Chem. Soc. 1992, 114, 5530.

[13] A. S. Mikherdov , A. S. Novikov , V. P. Boyarskiy , V. Y. Kukushkin , Nat. Commun. 2020, 11, 2921. 32523100 10.1038/s41467-020-16748-x PMC7286913

[14a] O. Hassel , Science 1970, 170, 497; 17799698 10.1126/science.170.3957.497

[14b] P. Politzer , P. Lane , M. C. Concha , Y. Ma , J. S. Murray , J. Mol. Model. 2007, 13, 305; 17013631 10.1007/s00894-006-0154-7

[14c] P. Metrangolo , G. Resnati , Science 2008, 321, 918; 18703728 10.1126/science.1162215

[14d] M. Erdélyi , Chem. Soc. Rev. 2012, 41, 3547; 22334193 10.1039/c2cs15292d

[14e] G. R. Desiraju , P. S. Ho , L. Kloo , A. C. Legon , R. Marquardt , P. Metrangolo , P. Politzer , G. Resnati , K. Rissanen , Pure Appl. Chem. 2013, 85, 1711;

[14f] G. Cavallo , P. Metrangolo , R. Milani , T. Pilati , A. Priimagi , G. Resnati , G. Terraneo , Chem. Rev. 2016, 116, 2478; 26812185 10.1021/acs.chemrev.5b00484 PMC4768247

[14g] P. Metrangolo , H. Neukirch , T. Pilati , G. Resnati , Acc. Chem. Res. 2005, 38, 386. 15895976 10.1021/ar0400995

[15] M. H. Kolář , P. Hobza , Chem. Rev. 2016, 116, 5155. 26840433 10.1021/acs.chemrev.5b00560

[16] P. Politzer , J. S. Murray , ChemPhysChem 2013, 14, 278. 23303575 10.1002/cphc.201200799

[17a] S. Schindler , S. M. Huber , Halogen Bonding II: Impact on Materials Chemistry and Life Sciences (Eds.: P. Metrangolo , G. Resnati ), Springer International Publishing, Cham, 2015, pp. 167–203;

[17b] D. Bulfield , S. M. Huber , Chem. Eur. J. 2016, 22, 14434; 27465662 10.1002/chem.201601844

[17c] S. Guha , I. Kazi , A. Nandy , G. Sekar , Eur. J. Org. Chem. 2017, 5497; 10.1021/acs.joc.0c02672 33491457

[17d] R. Tepper , U. S. Schubert , Angew. Chem. Int. Ed. 2018, 57, 6004; 10.1002/anie.201707986 29341377

[17e] J. Bamberger , F. Ostler , O. G. Mancheño , ChemCatChem 2019, 11, 5198; 31894187 10.1002/cctc.201901215 PMC6919929

[17f] R. L. Sutar , S. M. Huber , ACS Catal. 2019, 9, 9622.

[18] M. Breugst , J. J. Koenig , Eur. J. Org. Chem. 2020, 5471.

[19a] A. Vargas Jentzsch , Pure Appl. Chem. 2015, 87, 15;

[19b] A. Brown , P. D. Beer , Chem. Commun. 2016, 52, 8645; 10.1039/c6cc03638d 27273600

[19c] J. Pancholi , P. D. Beer , Coord. Chem. Rev. 2020, 416, 213281.

[20] J. Y. C. Lim , P. D. Beer , Chem 2018, 4, 731.

[21a] L. C. Gilday , S. W. Robinson , T. A. Barendt , M. J. Langton , B. R. Mullaney , P. D. Beer , Chem. Rev. 2015, 115, 7118; 26165273 10.1021/cr500674c

[21b] R. W. Troff , T. Mäkelä , F. Topić , A. Valkonen , K. Raatikainen , K. Rissanen , Eur. J. Org. Chem. 2013, 1617.

[22] T. Clark , M. Hennemann , J. S. Murray , P. Politzer , J. Mol. Model. 2007, 13, 291. 16927107 10.1007/s00894-006-0130-2

[23a] V. Nesterov , D. Reiter , P. Bag , P. Frisch , R. Holzner , A. Porzelt , S. Inoue , Chem. Rev. 2018, 118, 9678; 29969239 10.1021/acs.chemrev.8b00079

[23b] A. Doddi , M. Peters , M. Tamm , Chem. Rev. 2019, 119, 6994. 30983327 10.1021/acs.chemrev.8b00791

[24] A. J. Arduengo III , M. Tamm , J. C. Calabrese , J. Am. Chem. Soc. 1994, 116, 3625.

[25] R. A. Squitieri , K. P. Fitzpatrick , A. A. Jaworski , K. A. Scheidt , Chem. Eur. J. 2019, 25, 10069. 31112630 10.1002/chem.201902298

[26] M. L. Cole , C. Jones , P. C. Junk , New J. Chem. 2002, 26, 1296.

[27] S. Pal , M. A. Manae , V. V. Khade , S. Khan , J. Indian Chem. Soc. 2018, 95, 765.

[28a] N. Schulz , S. Schindler , S. M. Huber , M. Erdelyi , J. Org. Chem. 2018, 83, 10881; 30110550 10.1021/acs.joc.8b01567

[28b] N. Schulz , P. Sokkar , E. Engelage , S. Schindler , M. Erdelyi , E. Sanchez-Garcia , S. M. Huber , Chem. Eur. J. 2018, 24, 3464. 29160593 10.1002/chem.201705032

[29a] D. Holschumacher , T. Bannenberg , C. G. Hrib , P. G. Jones , M. Tamm , Angew. Chem. Int. Ed. 2008, 47, 7428; 10.1002/anie.200802705 18666192

[29b] D. Holschumacher , C. Taouss , T. Bannenberg , C. G. Hrib , C. G. Daniliuc , P. G. Jones , M. Tamm , Dalton Trans. 2009, 6927; 20449131 10.1039/b908074k

[29c] D. Holschumacher , T. Bannenberg , K. Ibrom , C. G. Daniliuc , P. G. Jones , M. Tamm , Dalton Trans. 2010, 39, 10590; 20936221 10.1039/c0dt01045f

[29d] D. Holschumacher , C. G. Daniliuc , P. G. Jones , M. Tamm , Z. Naturforsch. B 2011, 66, 371;

[29e] S. Kronig , E. Theuergarten , D. Holschumacher , T. Bannenberg , C. G. Daniliuc , P. G. Jones , M. Tamm , Inorg. Chem. 2011, 50, 7344; 21718018 10.1021/ic201290g

[29f] E. L. Kolychev , T. Bannenberg , M. Freytag , C. G. Daniliuc , P. G. Jones , M. Tamm , Chem. Eur. J. 2012, 18, 16938; 23150467 10.1002/chem.201202840

[29g] E. Theuergarten , T. Bannenberg , M. D. Walter , D. Holschumacher , M. Freytag , C. G. Daniliuc , P. G. Jones , M. Tamm , Dalton Trans. 2014, 43, 1651. 24217215 10.1039/c3dt52742e

[30] A. Nasr , A. Winkler , M. Tamm , Coord. Chem. Rev. 2016, 316, 68.

[31a] S. Kronig , E. Theuergarten , C. G. Daniliuc , P. G. Jones , M. Tamm , Angew. Chem. Int. Ed. 2012, 51, 3240; 10.1002/anie.201108813 22337636

[31b] A. Igarashi , E. L. Kolychev , M. Tamm , K. Nomura , Organometallics 2016, 35, 1778;

[31c] G. Nagai , T. Mitsudome , K. Tsutsumi , S. Sueki , T. Ina , M. Tamm , K. Nomura , J. Jpn. Petrol. Inst. 2017, 60, 256;

[31d] K. Nomura , G. Nagai , I. Izawa , T. Mitsudome , M. Tamm , S. Yamazoe , ACS Omega 2019, 4, 18833; 31737845 10.1021/acsomega.9b02828 PMC6854829

[31e] K. Nomura , G. Nagai , A. Nasr , K. Tsutsumi , Y. Kawamoto , K. Koide , M. Tamm , Organometallics 2019, 38, 3233;

[31f] J. Frosch , M. Freytag , P. G. Jones , M. Tamm , J. Organomet. Chem. 2020, 918, 121311;

[31g] M. Koneczny , L. Phong Ho , A. Nasr , M. Freytag , P. G. Jones , M. Tamm , Adv. Synth. Catal. 2020, 362, 3663.

[32] E. L. Kolychev , S. Kronig , K. Brandhorst , M. Freytag , P. G. Jones , M. Tamm , J. Am. Chem. Soc. 2013, 135, 12448. 23883399 10.1021/ja406529c

[33] A. Winkler , K. Brandhorst , M. Freytag , P. G. Jones , M. Tamm , Organometallics 2016, 35, 1160.

[34] L. P. Ho , A. Nasr , P. G. Jones , A. Altun , F. Neese , G. Bistoni , M. Tamm , Chem. Eur. J. 2018, 24, 18922. 30357989 10.1002/chem.201804714

[35a] L. P. Ho , M.-K. Zaretzke , T. Bannenberg , M. Tamm , Chem. Commun. 2019, 55, 10709; 10.1039/c9cc05739k 31429453

[35b] L. P. Ho , L. Körner , T. Bannenberg , M. Tamm , Dalton Trans. 2020, 49, 13207. 32785308 10.1039/d0dt02392b

[36a] A. J. A. Baskar , A. S. Rajpurohit , M. Panneerselvam , M. Jaccob , D. RoopSingh , V. Kannappan , Chem. Data Collect. 2017, 7–8, 80;

[36b] B. B. Bhowmik , S. P. Chattopadhyay , Spectrochim. Acta A 1981, 37, 135;

[36c] H. A. Benesi , J. H. Hildebrand , J. Am. Chem. Soc. 1949, 71, 2703.

[37] M. Mantina , A. C. Chamberlin , R. Valero , C. J. Cramer , D. G. Truhlar , J. Phys. Chem. A 2009, 113, 5806. 19382751 10.1021/jp8111556 PMC3658832

[38] Q. J. Shen , X. Pang , X. R. Zhao , H. Y. Gao , H.-L. Sun , W. J. Jin , CrystEngComm 2012, 14, 5027.

[39] M. Bujak , H.-G. Stammler , S. Blomeyer , N. W. Mitzel , Chem. Commun. 2018, 54, 175. 10.1039/c8cc08980a 30519685

[40a] P. M. J. Szell , B. Gabidullin , D. L. Bryce , Acta Crystallogr. Sect. B 2017, 73, 153; 10.1107/S2052520617000944 28362277

[40b] L. C. Roper , C. Präsang , V. N. Kozhevnikov , A. C. Whitwood , P. B. Karadakov , D. W. Bruce , Cryst. Growth Des. 2010, 10, 3710;

[40c] L. C. Gilday , N. G. White , P. D. Beer , Dalton Trans. 2013, 42, 15766. 24056495 10.1039/c3dt52093e

[41] D. Dolenc , B. Modec , New J. Chem. 2009, 33, 2344.

[42] A. J. Arduengo , F. Davidson , H. V. R. Dias , J. R. Goerlich , D. Khasnis , W. J. Marshall , T. K. Prakasha , J. Am. Chem. Soc. 1997, 119, 12742.

[43] A. J. Arduengo , R. Krafczyk , R. Schmutzler , H. A. Craig , J. R. Goerlich , W. J. Marshall , M. Unverzagt , Tetrahedron 1999, 55, 14523.

[44] N. Kuhn , A. Abu-Rayyan , K. Eichele , S. Schwarz , M. Steimann , Inorg. Chim. Acta 2004, 357, 1799.

[45a] N. Kuhn , A. Abu-Rayyan , K. Eichele , C. Piludu , M. Steimann , Z. anorg. allg. Chem. 2004, 630, 495;

[45b] N. Kuhn , A. Abu-Rayyan , M. Göhner , M. Steimann , Z. anorg. allg. Chem. 2002, 628, 1721;

[45c] N. Kuhn , J. Fahl , R. Fawzi , C. Maichle-Mößmer , M. Steimann , Z. Naturforsch. B 1998, 53, 720.

[46] A. L. Spek , Acta Crystallogr. Sect. C 2015, 71, 9. 10.1107/S2053229614024929 25567569

[47] A. A. Danopoulos , P. Braunstein , Chem. Commun. 2014, 50, 3055. 10.1039/c3cc49517e 24515245

[48] A. Caballero , N. G. White , P. D. Beer , Angew. Chem. Int. Ed. 2011, 50, 1845; 10.1002/anie.201006916 21328653

[49a] A. A. Neverov , H. X. Feng , K. Hamilton , R. S. Brown , J. Org. Chem. 2003, 68, 3802; 12737557 10.1021/jo020750m

[49b] Y. Kim , E. J. Mckinley , K. E. Christensen , N. H. Rees , A. L. Thompson , Cryst. Growth Des. 2014, 14, 6294;

[49c] L. C. F. Morgan , Y. Kim , J. N. Blandy , C. A. Murray , K. E. Christensen , A. L. Thompson , Chem. Commun. 2018, 54, 9849. 10.1039/c8cc05430d 30112538

[50] S. Grimme , J. Comput. Chem. 2006, 27, 1787. 16955487 10.1002/jcc.20495

[51] N. J. M. Amezaga , S. C. Pamies , N. M. Peruchena , G. L. Sosa , J. Phys. Chem. A 2010, 114, 552. 19919022 10.1021/jp907550k

[52a] M. G. Sarwar , B. Dragisic , L. J. Salsberg , C. Gouliaras , M. S. Taylor , J. Am. Chem. Soc. 2010, 132, 1646; 20078063 10.1021/ja9086352

[52b] M. G. Chudzinski , M. S. Taylor , J. Org. Chem. 2012, 77, 3483; 22432921 10.1021/jo300279m

[52c] S. Kozuch , J. M. L. Martin , J. Chem. Theory Comput. 2013, 9, 1918; 26583543 10.1021/ct301064t

[52d] N. Nagels , Y. Geboes , B. Pinter , F. de Proft , W. A. Herrebout , Chem. Eur. J. 2014, 20, 8433; 24898290 10.1002/chem.201402116

[52e] R. Tepper , B. Schulze , M. Jäger , C. Friebe , D. H. Scharf , H. Görls , U. S. Schubert , J. Org. Chem. 2015, 80, 3139; 25671504 10.1021/acs.joc.5b00028

[52f] S. V. Rosokha , E. A. Loboda , J. Phys. Chem. A 2015, 119, 3833; 25825078 10.1021/acs.jpca.5b01600

[52g] L. Maugeri , J. Asencio-Hernández , T. Lébl , D. B. Cordes , A. M. Z. Slawin , M.-A. Delsuc , D. Philp , Chem. Sci. 2016, 7, 6422; 28451098 10.1039/c6sc01974a PMC5355977

[52h] K.-N. Truong , J. M. Rautiainen , K. Rissanen , R. Puttreddy , Cryst. Growth Des. 2020, 20, 5330;

[52i] L. N. Anderson , F. W. Aquino , A. E. Raeber , X. Chen , B. M. Wong , J. Chem. Theory Comput. 2018, 14, 180; 29202234 10.1021/acs.jctc.7b01078

[52j] S. J. Ang , C. T. Ser , M. W. Wong , J. Comput. Chem. 2019, 40, 1829. 30950537 10.1002/jcc.25835

[53] R. Puttreddy , J. M. Rautiainen , T. Mäkelä , K. Rissanen , Angew. Chem. Int. Ed. 2019, 58, 18610; 10.1002/anie.201909759 31613414

[54] D. C. Georgiou , P. Butler , E. C. Browne , D. J. D. Wilson , J. L. Dutton , Aust. J. Chem. 2013, 66, 1179.

[55a] J. P. Wagner , P. R. Schreiner , J. Chem. Theory Comput. 2016, 12, 231; 26606127 10.1021/acs.jctc.5b01100

[55b] D. J. Liptrot , P. P. Power , Nat. Rev. Chem. 2017, 1, 0004.

[56] S. Grimme , J. Antony , S. Ehrlich , H. Krieg , J. Chem. Phys. 2010, 132, 154104. 20423165 10.1063/1.3382344

[57a] A. D. Becke , J. Chem. Phys. 1993, 98, 5648;

[57b] B. Miehlich , A. Savin , H. Stoll , H. Preuss , Chem. Phys. Lett. 1989, 157, 200;

[57c] C. Lee , W. Yang , R. G. Parr , Phys. Rev. B 1988, 37, 785. 10.1103/physrevb.37.785 9944570

[58] S. B. Hakkert , M. Erdélyi , J. Phys. Org. Chem. 2015, 28, 226.

[59a] R. F. W. Bader , Atoms in Molecules. A Quantum Theory, Clarendon Press, Oxford, 2003;

[59b] C. F. Matta , R. J. Boyd , The Quantum Theory of Atoms in Molecules. From Solid State to DNA and Drug Design, Wiley-VCH, Weinheim, 2007;

[59c] R. F. W. Bader , Chem. Rev. 1991, 91, 893.

[60a] J. Molina Molina , J. A. Dobado , Theor. Chem. Acc. 2001, 105, 328;

[60b] D. J. R. Duarte , G. L. Sosa , N. M. Peruchena , J. Mol. Model. 2013, 19, 2035; 23076553 10.1007/s00894-012-1624-8

[60c] A. Forni , S. Pieraccini , D. Franchini , M. Sironi , J. Phys. Chem. A 2016, 120, 9071; 27718571 10.1021/acs.jpca.6b07578

[60d] N. Han , Y. Zeng , C. Sun , X. Li , Z. Sun , L. Meng , J. Phys. Chem. A 2014, 118, 7058; 25102351 10.1021/jp502558p

[60e] O. A. Syzgantseva , V. Tognetti , L. Joubert , J. Phys. Chem. A 2013, 117, 8969; 24000903 10.1021/jp4059774

[60f] M. Domagała , A. Lutyńska , M. Palusiak , J. Phys. Chem. A 2018, 122, 5484; 29809012 10.1021/acs.jpca.8b03735

[60g] E. Bartashevich , I. Yushina , K. Kropotina , S. Muhitdinova , V. Tsirelson , Acta Crystallogr. Sect. B 2017, 73, 217. 10.1107/S2052520617002931 28362285

[61] K. Lamberts , P. Handels , U. Englert , E. Aubert , E. Espinosa , CrystEngComm 2016, 18, 3832.

[62a] P. L. A. Popelier , The Chemical Bond. Fundamental Aspects of Chemical Bonding (Eds.: G. Frenking , S. S. Shaik ), Wiley-VCH, Weinheim, 2014, pp. 271–308;

[62b] R. F. W. Bader , H. Essén , J. Chem. Phys. 1984, 80, 1943.

[63a] S. Shahbazian , Chem. Eur. J. 2018, 24, 5401; 29283195 10.1002/chem.201705163

[63b] R. F. W. Bader , J. Phys. Chem. A 2009, 113, 10391; 19722600 10.1021/jp906341r

[63c] S. Grimme , C. Mück-Lichtenfeld , G. Erker , G. Kehr , H. Wang , H. Beckers , H. Willner , Angew. Chem. Int. Ed. 2009, 48, 2592; 10.1002/anie.200805751 19229914

[63d] L. Zhao , S. Pan , N. Holzmann , P. Schwerdtfeger , G. Frenking , Chem. Rev. 2019, 119, 8781. 31251603 10.1021/acs.chemrev.8b00722

