# Supplementary material for: Halogen Complexes of Anionic N‐Heterocyclic Carbenes
Source: Chemistry. 2020 Dec 21;27(13):4349–63. doi: 10.1002/chem.202004418 (PMC7986712; doi:10.1002/chem.202004418)
Supplement: Supplementary file 1 — Supplementary [file CHEM-27-4349-s001.pdf]

Chemistry–A European Journal

Supporting Information

Halogen Complexes of Anionic N-Heterocyclic Carbenes

Jenni Frosch, Marvin Koneczny, Thomas Bannenberg, and Matthias Tamm*[a]

Contents

1. Experimental Details 3

1.1. Materials and Methods 3

2. NMR spectra 4

2.1. [(WCA-IDipp)I·C_6H_5Me] (**2a**·C_6H_5Me) 4

2.2. Toluene free Product [(WCA-IDipp)I] (**2a**)........ 5

2.3. [(WCA-IDipp)I·C_6H_5Cl] (**2a**·C_6H_5Cl) 8

2.4. [(WCA-IDipp)I·$ONMe_3$] (**2a**·$ONMe_3$) 11

2.5. [(WCA-IMes)I] (**2b**) 14

2.6. [(*m*-$XyF_6)_3$B(IDipp)I] (**2c**)........ 17

2.7. [(WCA-IDipp)Br] (**3a**) 20

2.8. [(WCA-IMes)Br] (**3b**)........ 23

2.9. [(WCA-IDipp)Cl] (**4**)........ 26

2.10. [(WCA-IDipp)I(IDipp)] (**5a**): 29

2.11. [(WCA-IDipp)I(IMes)] (**5b**):........ 33

2.12. [PPh_4][(WCA-IDipp)] (**6**):........ 37

2.13. [PPh_4][(WCA-IDipp)$_2$I] (**7a**):........ 41

2.14. [PPh_4][(WCA-IDipp)I(WCA-IMes)] (**7b**):........ 44

2.15. [PPh_4][(WCA-IDipp)$_2$Br] (**8**):........ 48

2.16. [(WCA-IDipp)H] (**9a**): 52

2.17. [(WCA-IMes)H] (**9b**):........ 55

3. X-ray crystal structure determinations 59

3.1. [(WCA-IDipp)I·Chlorobenzene] (**2a**·C_6H_5Cl) 60

3.2. [(WCA-IDipp)I·Toluene] (**2a**·C_6H_5Me) 61

3.3. [(WCA-IDipp)I·THF] (**2a**·THF)........ 62

3.4. [(WCA-IDipp)I·$ONMe_3$] (**2a**·$ONMe_3$)... 63
3.5. [(WCA-IDipp)I·CH_3CN] (**2a**·CH_3CN)... 64
3.6. [(WCA-IMes)I] (**2b**): 65
3.7. [(*m*-XyF_6)$_3$B(IDipp)I] (**2c**)... 67
3.8. [(WCA-IDipp)Br] (**3a**) 69
3.9. [(WCA-IMes)Br] (**3b**)... 71
3.10. [(WCA-IDipp)Cl] (**4**).. 73
3.11. [(WCA-IDipp)I(IDipp)] (**5a**) 75
3.12. [(WCA-IDipp)I(IMes)] (**5b**)... 76
3.13. [PPh_4][WCA-IDipp] (**6**) 77
3.14. [PPh_4][(WCA-IDipp)$_2$I] (**7a**)... 78
3.15. [PPh_4][(WCA-IDipp)I(WCA-IMes)] (**7b**) 79
3.16. [PPh_4][((*m*-XyF_6)$_3$B(IDipp))$_2$I] (**7d**).. 80
3.17. [PPh_4][(WCA-IDipp)$_2$Br] (**8**) 81
3.18. [(WCA-IDipp)H] (**9a**) 82
3.19. [(WCA-IMes)H] (**9b**)... 83
3.20. [$IDipp_2$I]I 84
3.21. [I^{t}BuH][(WCA-IDipp)$_2$I] 85
3.22. Table S1 (Part 1): Crystallographic Details... 86
Table S1 (Part 2): Crystallographic Details.. 87
Table S1 (Part 3): Crystallographic Details.. 88
Table S1 (Part 4): Crystallographic Details.. 89
Table S1 (Part 5): Crystallographic Details.. 90
Table S1 (Part 6): Crystallographic Details.. 91
Table S1 (Part 7): Crystallographic Details.. 92
4. Computational Details 93
5. Literature 97

1. Experimental Details

1.1. Materials and Methods

All operations with air- and moisture-sensitive compounds were performed in a glove box under a dry argon atmosphere (MBraun 200B) or on a vacuum line using Schlenk techniques. All solvents were distilled from Na/benzophenone or CaH_2, degassed prior to use and stored over molecular sieves (4 Å). The ^{1}H, $^{13}C\{^{1}H\}$, $^{11}B\{^{1}H\}$, $^{19}F\{^{1}H\}$ and $^{31}F\{^{1}H\}$ NMR spectra were recorded on Bruker DPX 200, Bruker AV 300, Bruker DRX 400, Bruker AV II 600, AVIII400 and AVIIIHD500 spectrometers at room temperature. ^{1}H and $^{13}C\{^{1}H\}$ NMR spectra were referenced against the (residual) solvent signals.[1] Boron trifluoride diethyl etherate ($BF_3{\cdot}OEt_2$) was used as external reference for $^{11}B\{^{1}H\}$.[2] Trichlorofluoromethane ($CFCl_3$) was used as external reference for $^{19}F\{^{1}H\}$. Chemical shifts are reported in ppm (parts per million). $^{11}B\{^{1}H\}$, $^{13}C\{^{1}H\}$ and $^{19}F\{^{1}H\}$ NMR spectra were obtained applying composite pulse proton decoupling. Coupling constants (J) are reported in Hertz (Hz), and splitting patterns are indicated as s (singlet), d (doublet), t (triplet), q (quartet), m (multiplet), sept (septet) and br (broad). NMR assignments were made using additional 2D NMR experiments.

Elemental analysis was carried out with a Vario Micro Cube System. Unless otherwise indicated, all starting materials were obtained from Sigma-Aldrich, ABRC, TCI, Acros or Fluka and were purified if necessary. [(WCA-IDipp)Li(Toluene)] (**1a**), [(WCA-IMes)Li(Toluene)] (**1b**), and [(*m*-$XyF_6)_3$B(IDipp)Li(Toluene)] (**1c**), were prepared according to literature procedures.[3,4] Iodine was sublimed under an inert argon atmosphere and stored inside the glove box. Tetraphenylphosphonium chloride (PPh_4Cl) was recrystallized from dichloromethane and dried at 175 °C under high vacuum for 3 days at.

2. NMR spectra

2.1. [(WCA-IDipp)I·C_6H_5Me] (**2a**·C_6H_5Me)

^{1}H NMR (300 MHz, THF-d_8) of **2a**·C_6H_5Me:

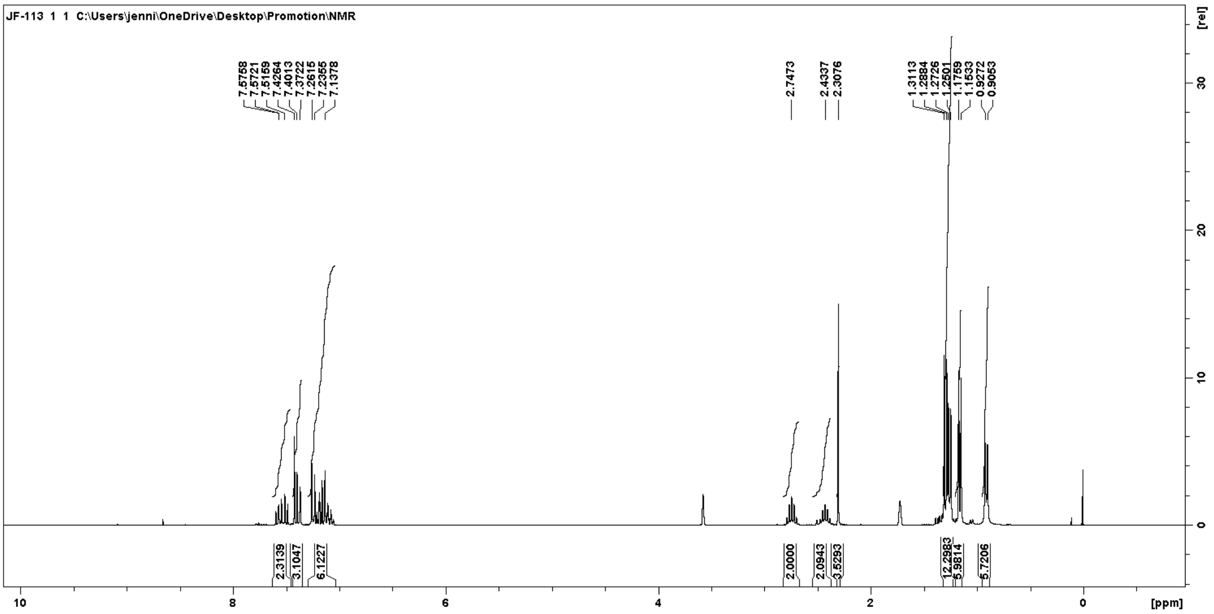

^{11}B{^{1}H} NMR (96 MHz, THF-d_8) of **2a**·C_6H_5Me:

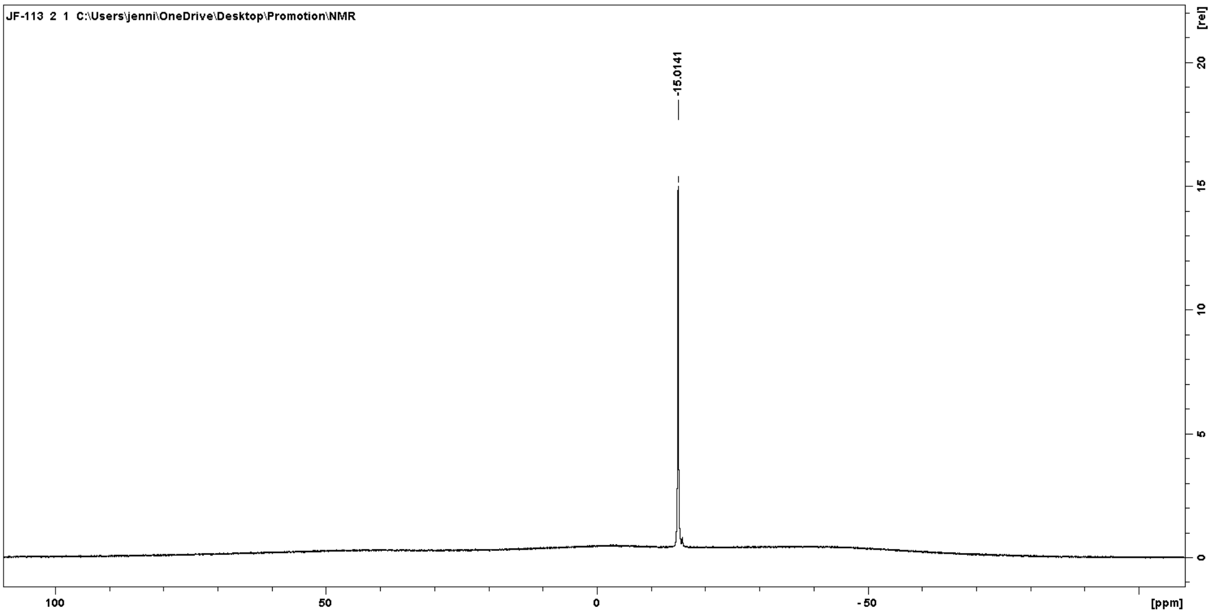

$^{19}F\{^1H\}$ NMR (282 MHz, THF-d_8) of **2a**·C_6H_5Me:

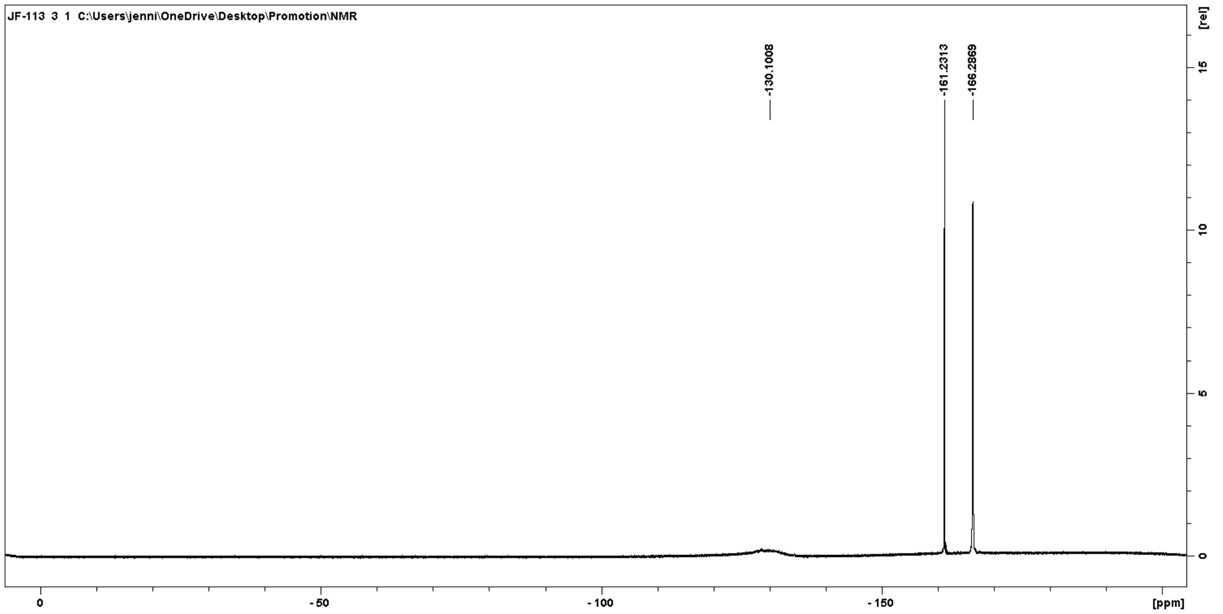

2.2. Toluene free Product [(WCA-IDipp)I] (2a)

^1H NMR (500 MHz, C_6D_6) of **2a**:

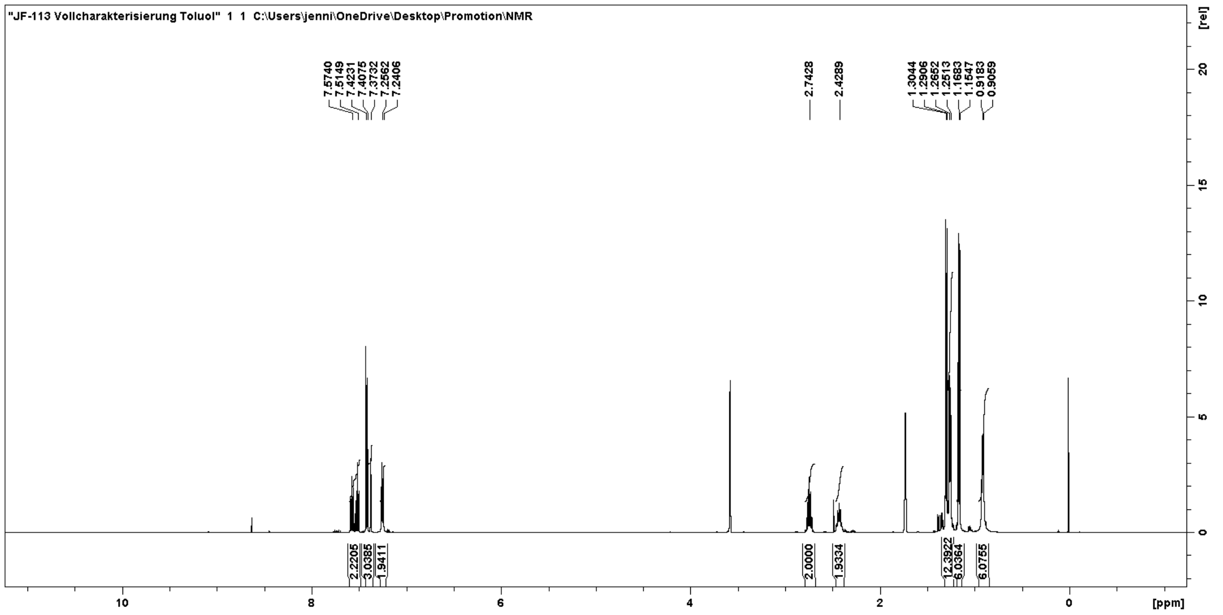

^{13}C NMR (125 MHz, C_6D_6) of **2a**:

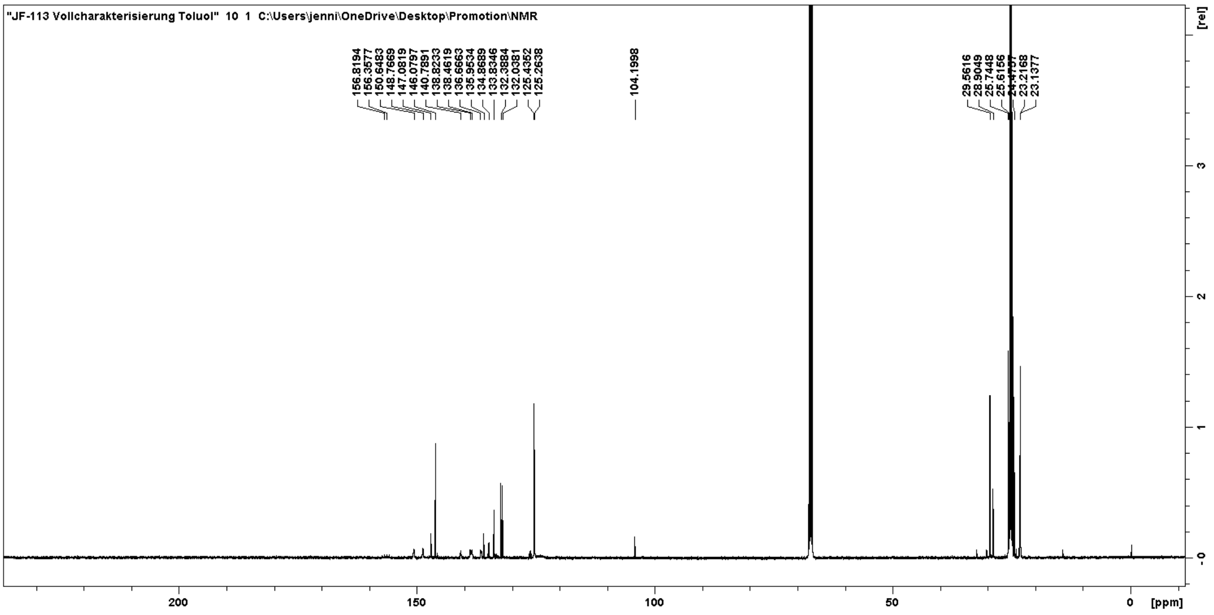

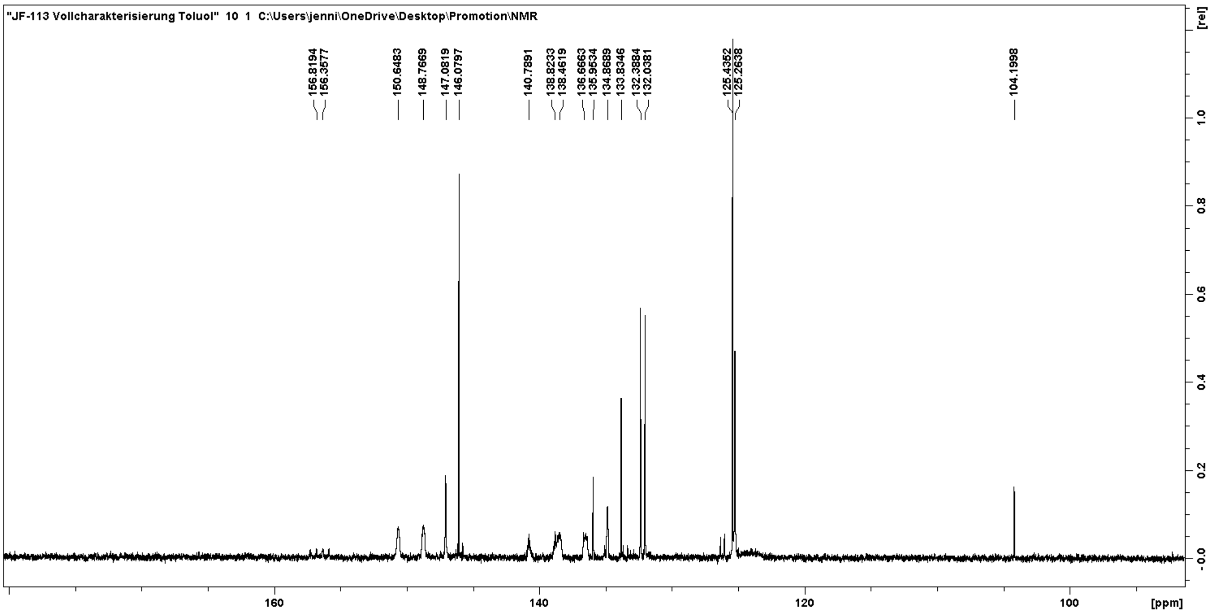

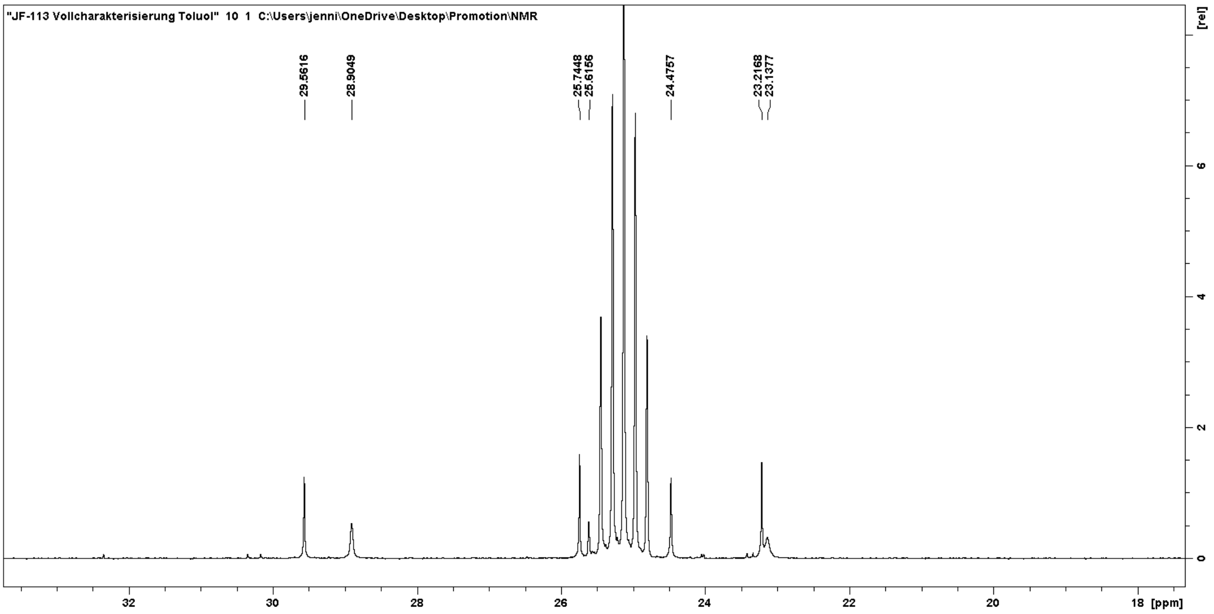

$^{11}B\{^{1}H\}$ NMR (128 MHz, C_6D_6) of **2a**:

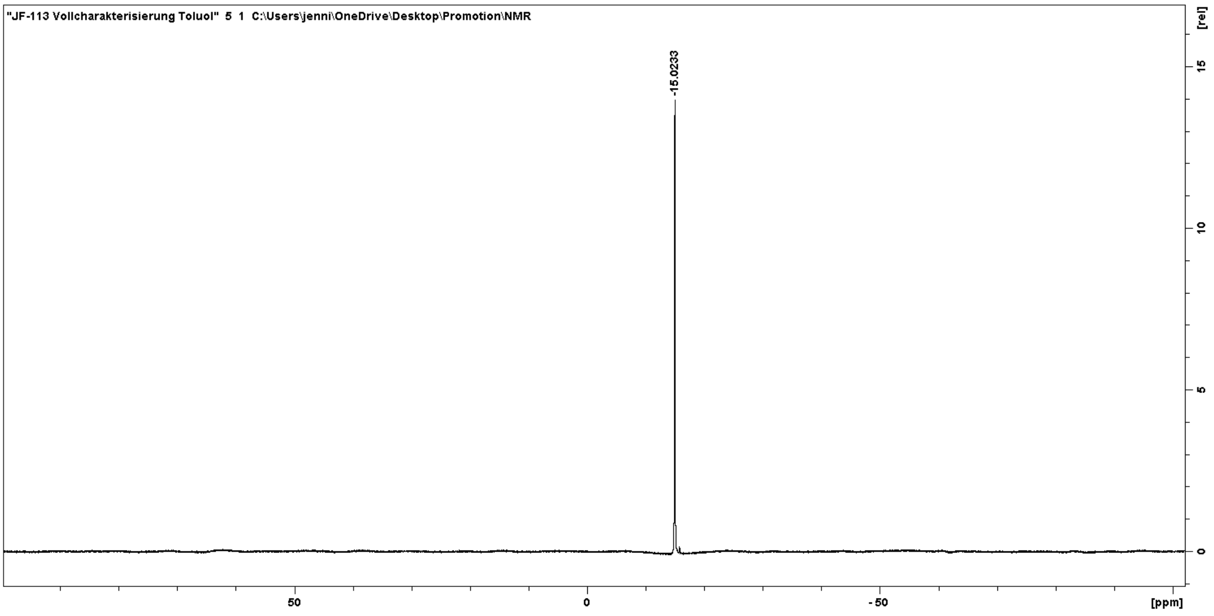

^{19}F{^{1}H} NMR (376 MHz, C_6D_6) of **2a**:

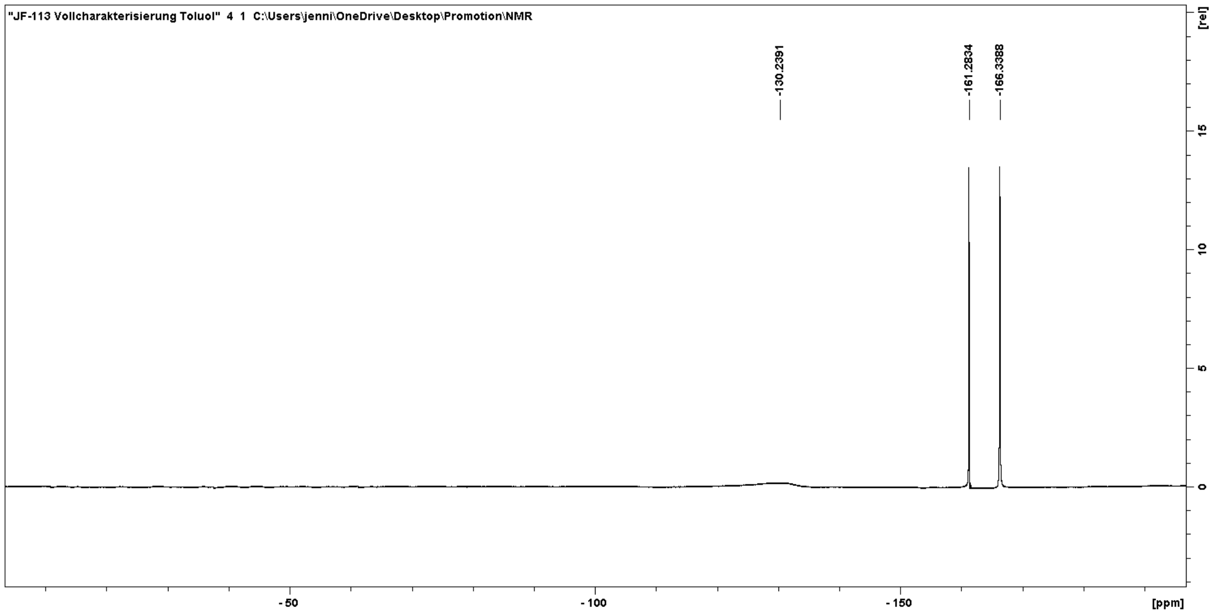

2.3. [(WCA-IDipp)I·C_6H_5Cl] (**2a**·C_6H_5Cl)

^{1}H NMR (500 MHz, THF-d_8) of **2a**·C_6H_5Cl:

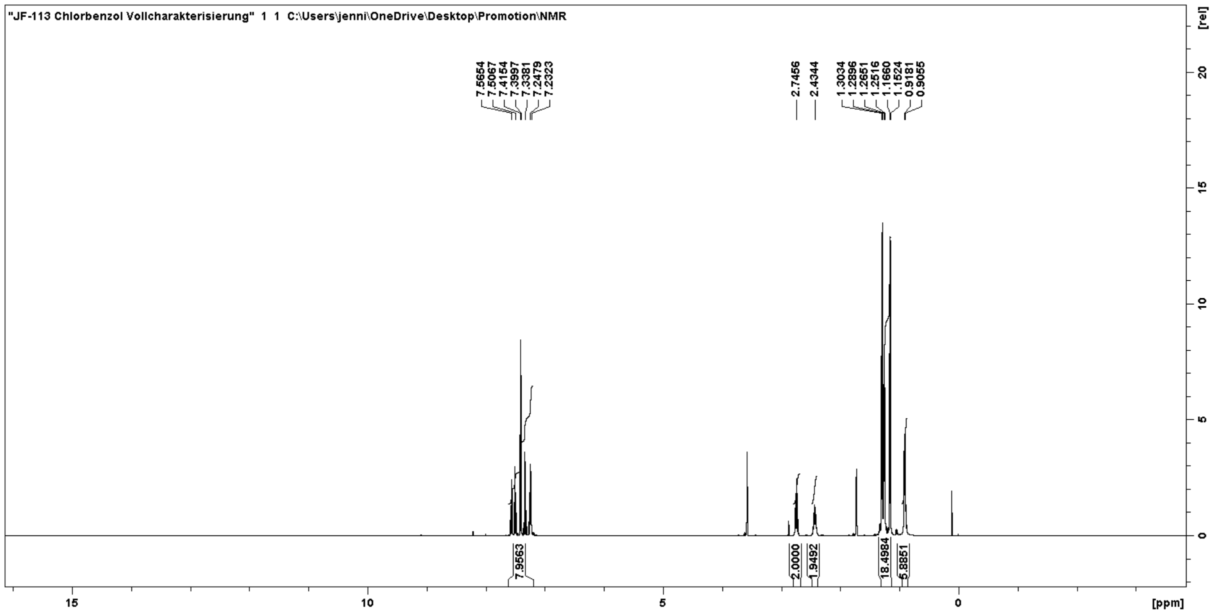

^{13}C NMR (125 MHz, THF-d_8) of **2a**·C_6H_5Cl:

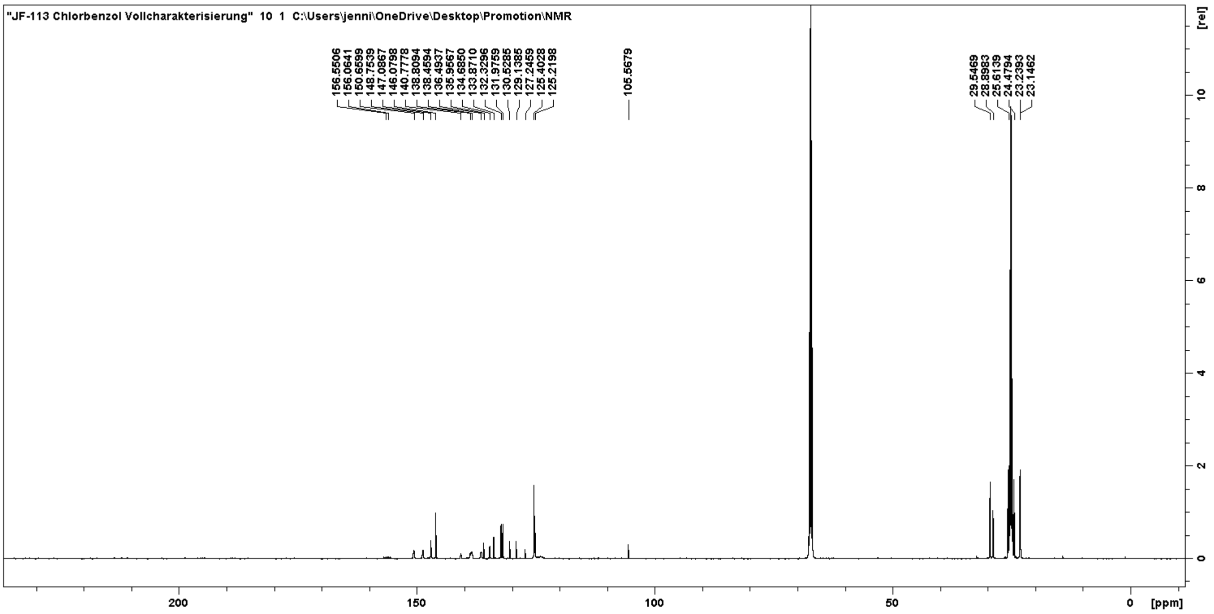

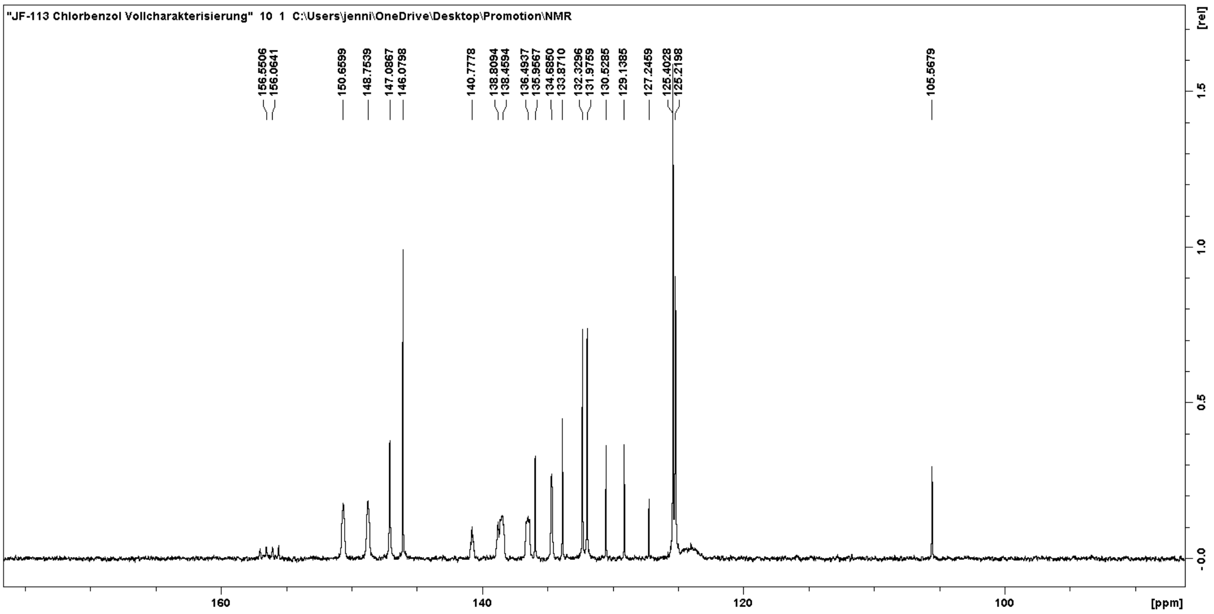

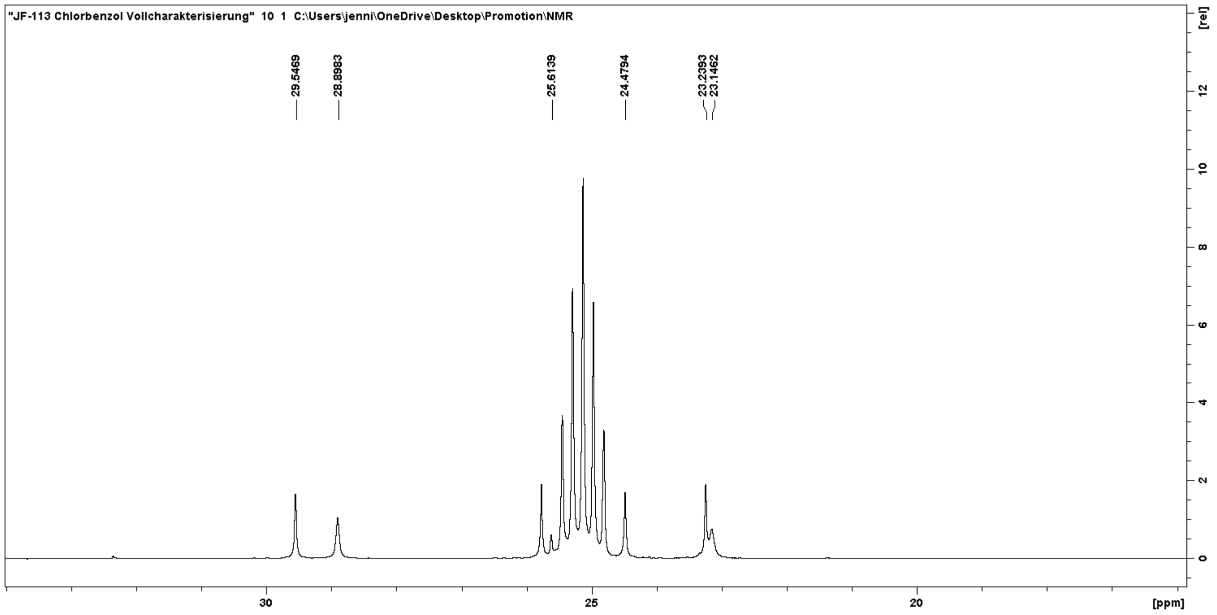

$^{11}B\{^{1}H\}$ **NMR** (128 MHz, THF-d_8) of **2a**·C_6H_5Cl:

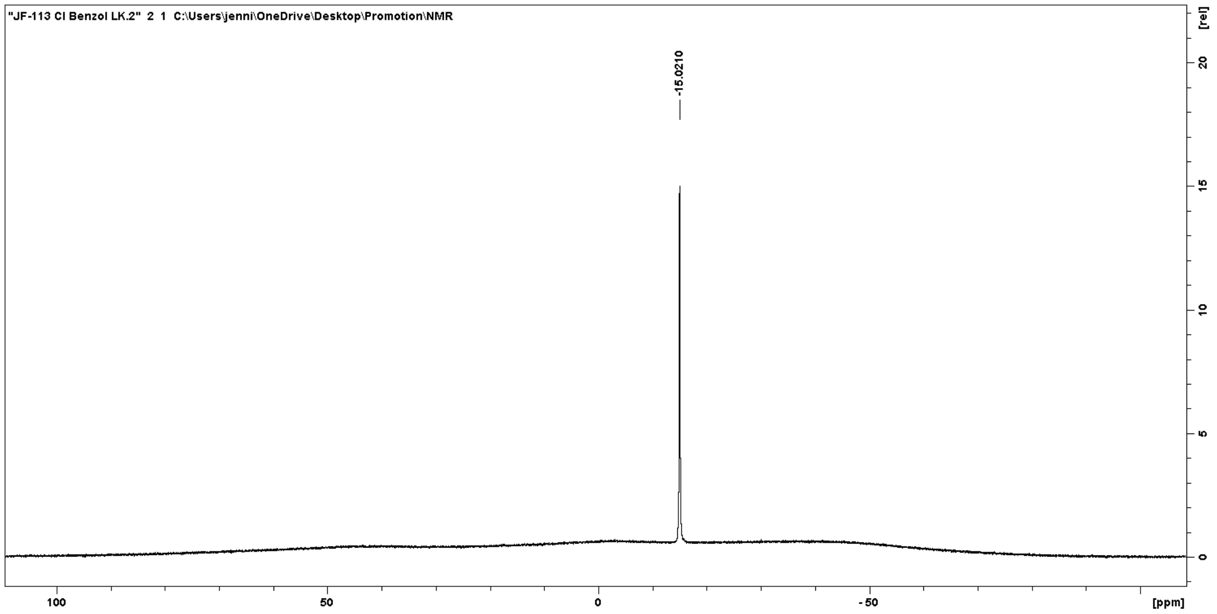

^{19}**F{^{1}H} NMR** (376 MHz, THF-d_8) of **2a**·C_6H_5Cl:

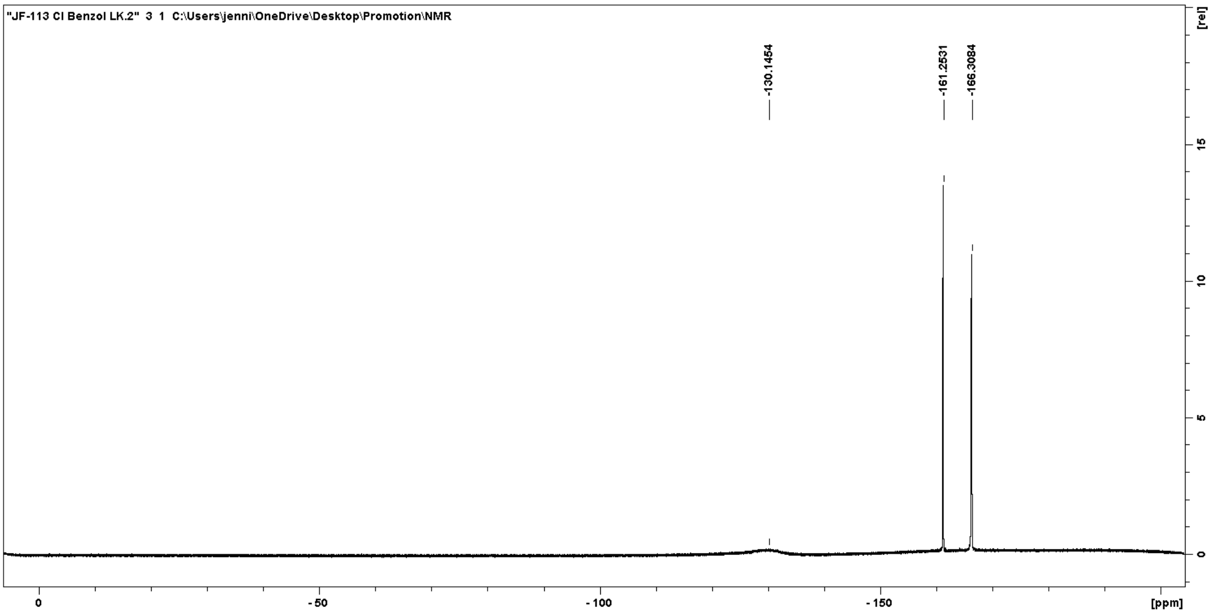

2.4. [(WCA-IDipp)I·ONMe$_3$] (2a·ONMe$_3$)

^{1}**H NMR** (500 MHz, THF-d_8) of **2a**·$ONMe_3$:

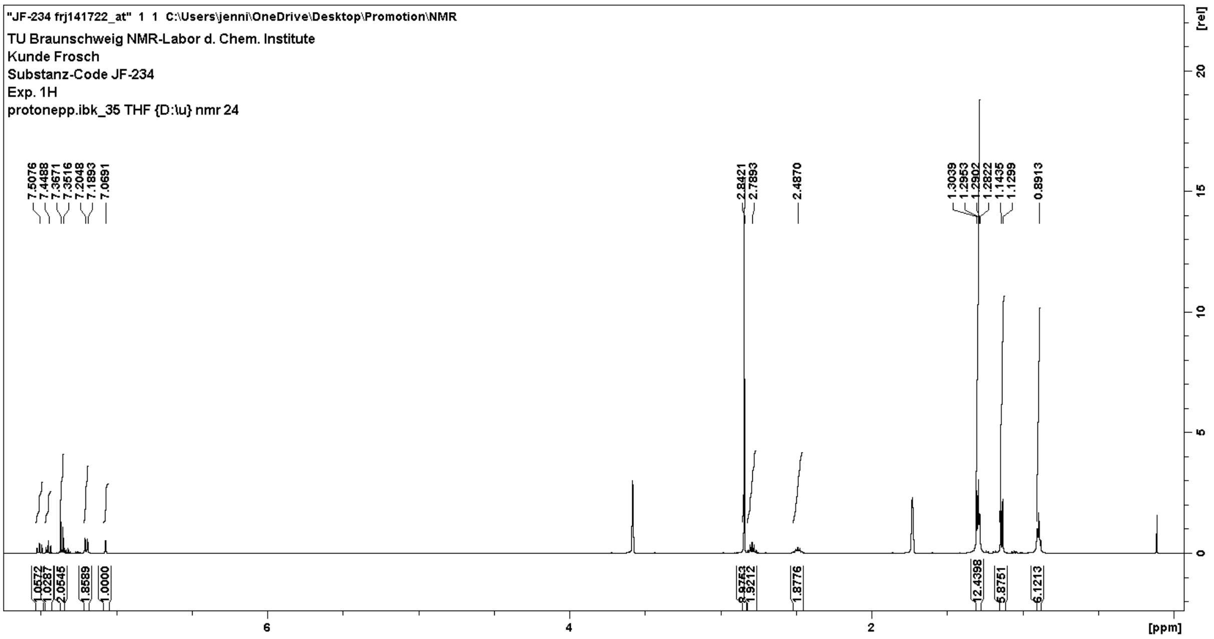

$^{13}C\{^{1}H\}$ NMR (125 MHz, THF-d_8) of **2a**·$ONMe_3$:

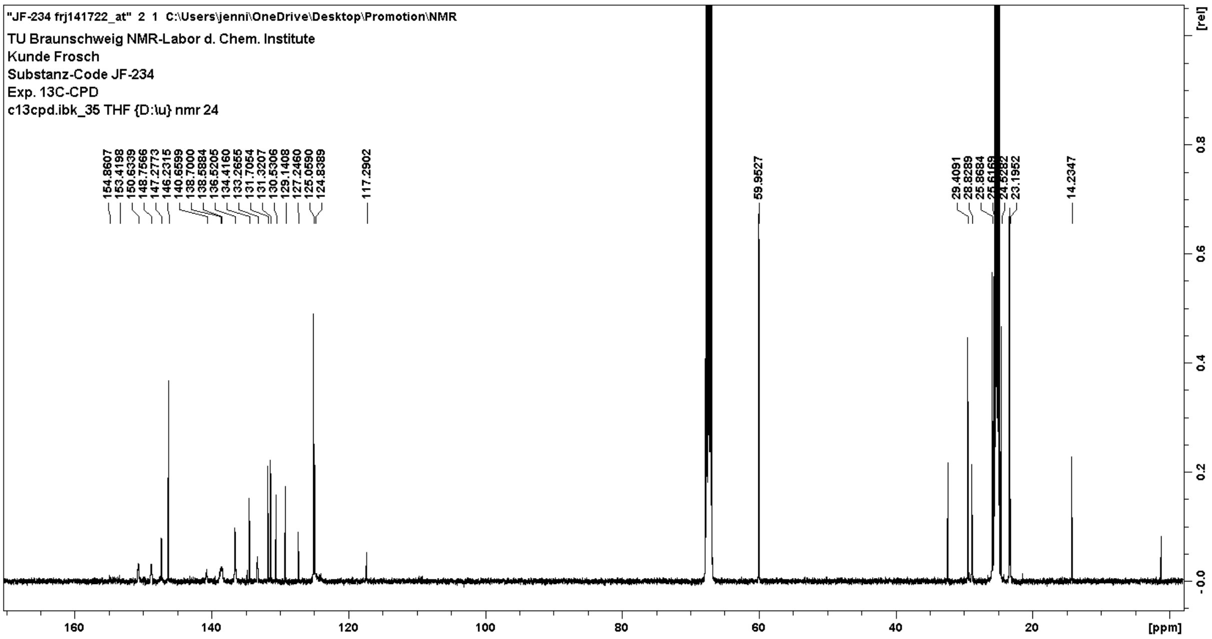

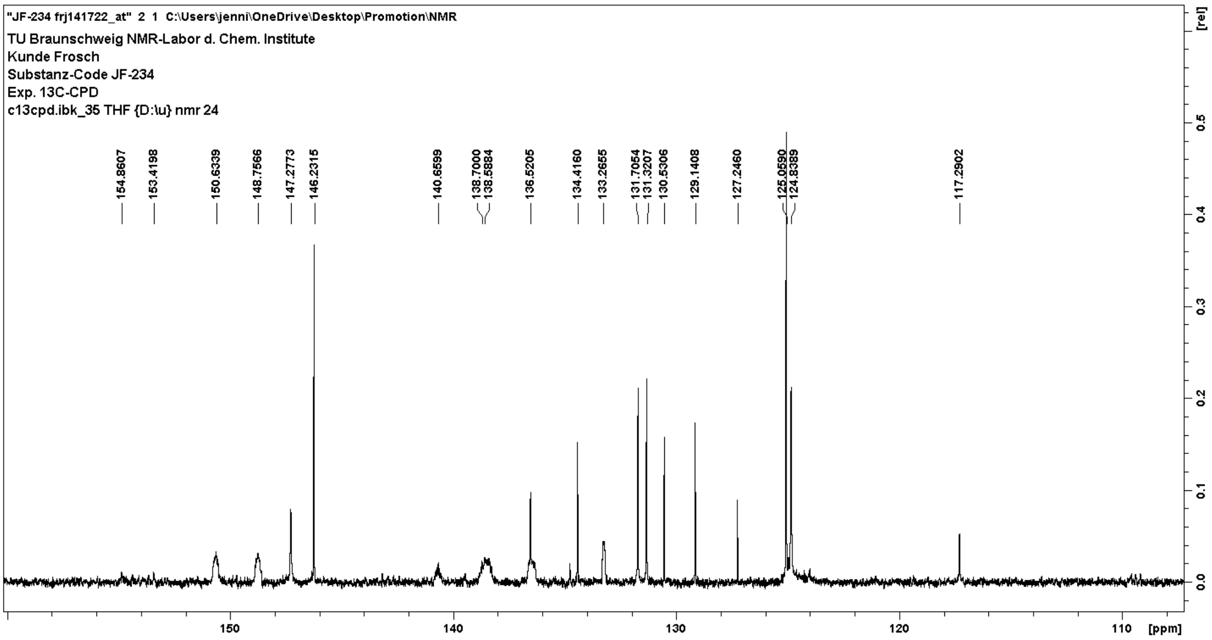

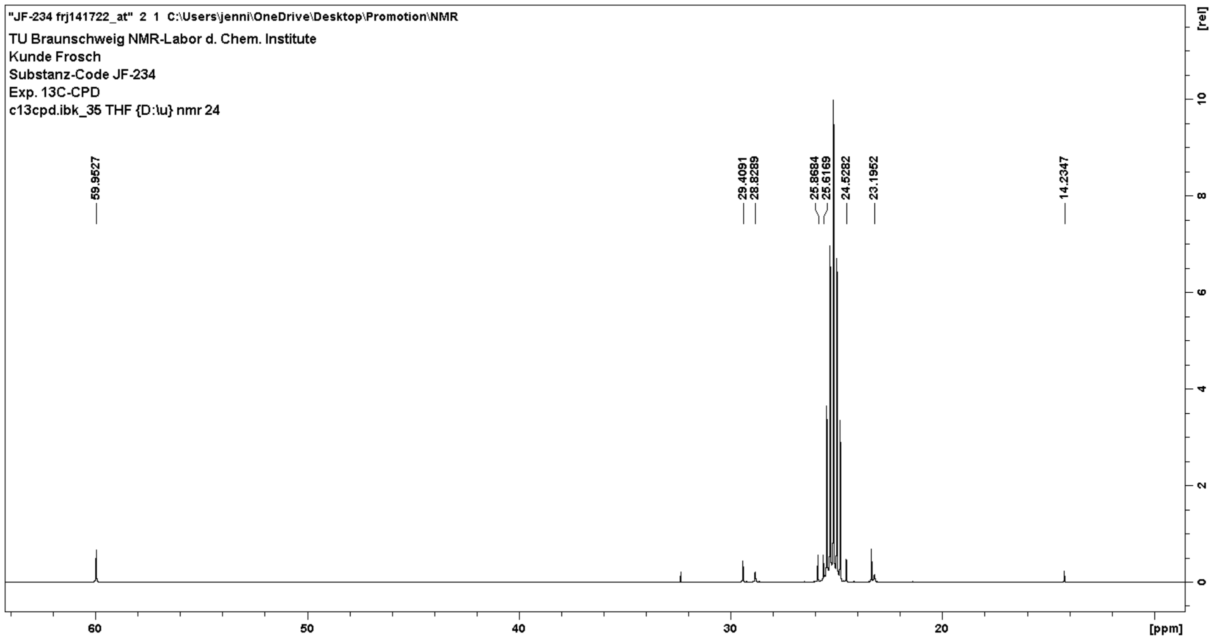

^{11}B{^{1}H} NMR (160 MHz, THF-d_8) of **2a**·ONMe$_3$:

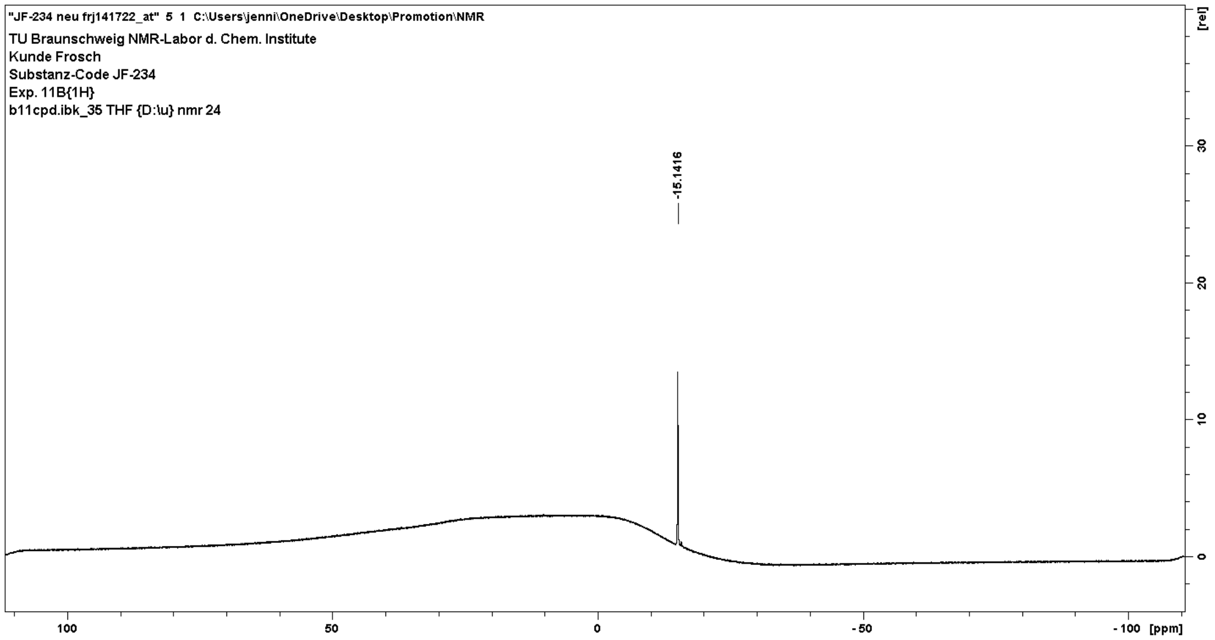

$^{19}F\{^{1}H\}$ **NMR** (470 MHz, THF-d_8) of **2a**·$ONMe_3$:

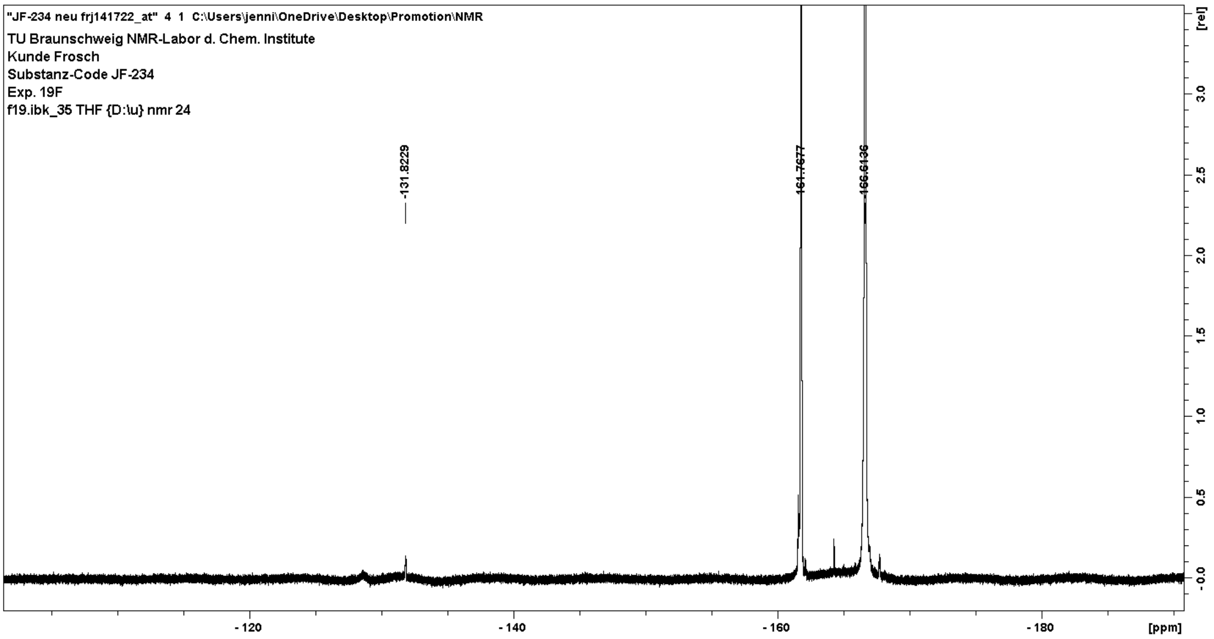

2.5. [(WCA-IMes)I] (2b)

^{1}**H NMR** (500 MHz, THF-d_8) of **2b**:

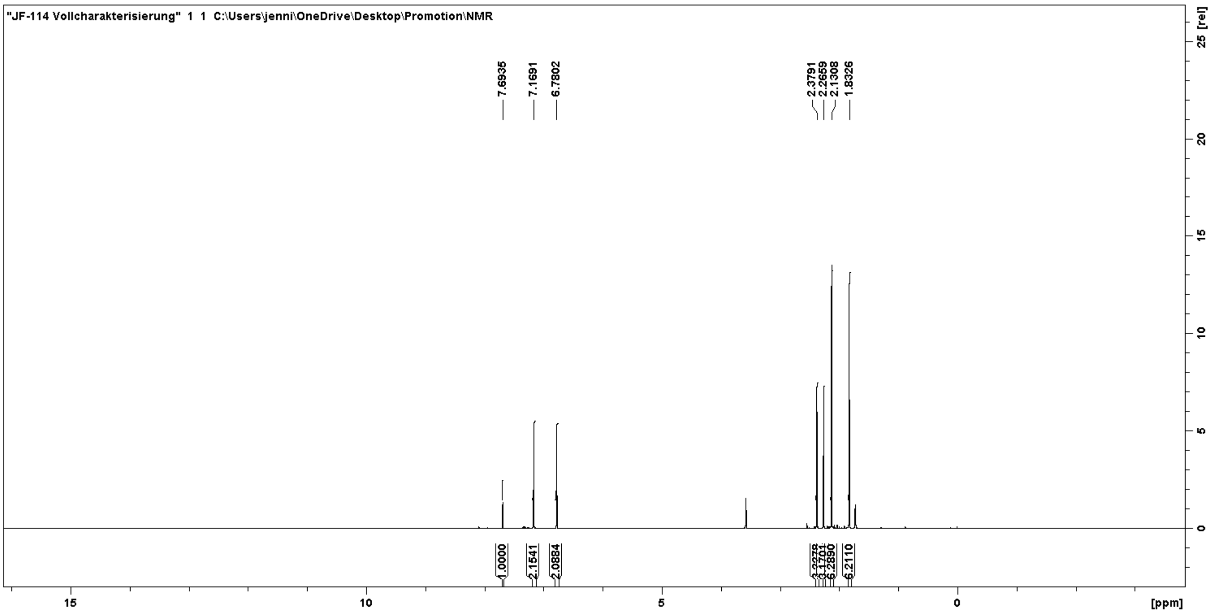

$^{13}C\{^{1}H\}$ NMR (125 MHz, THF-d_8) of **2b**:

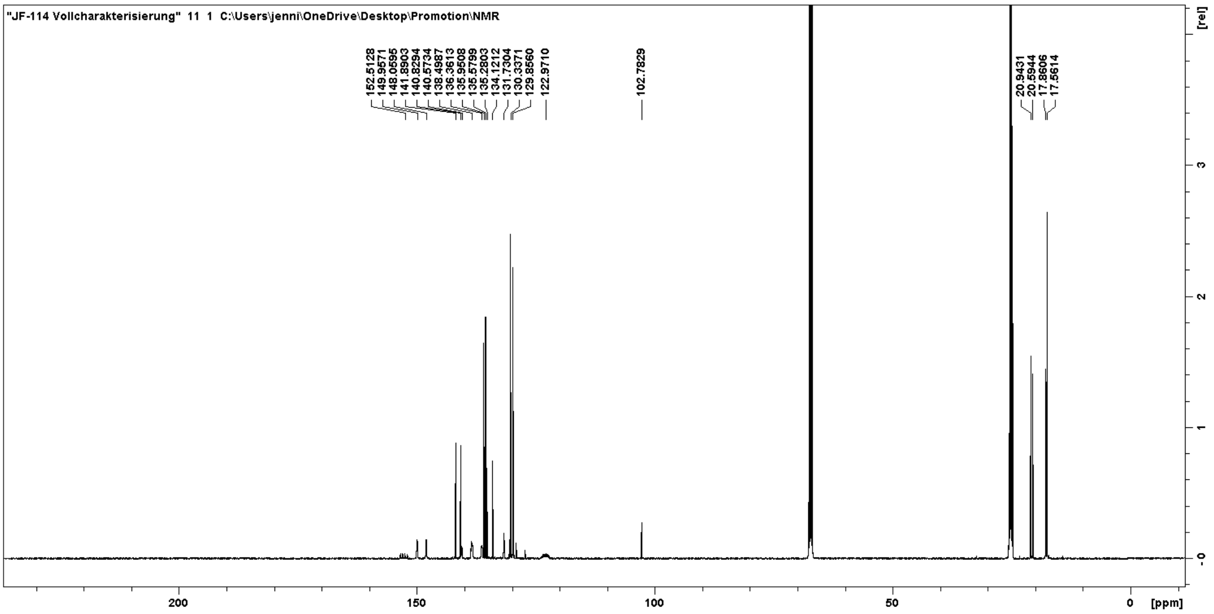

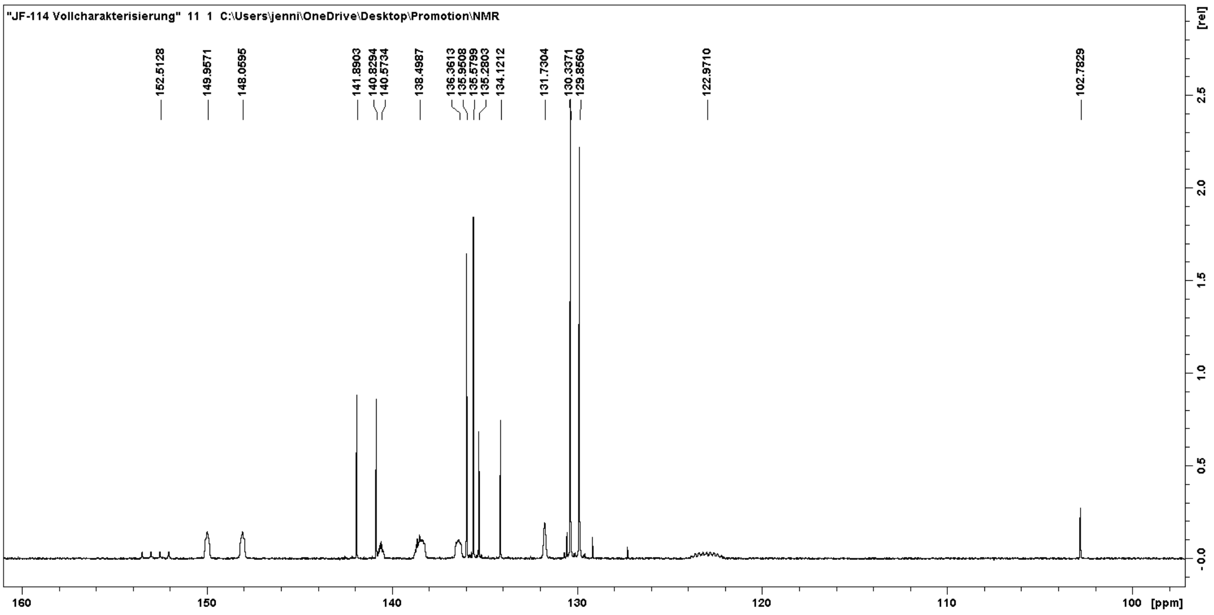

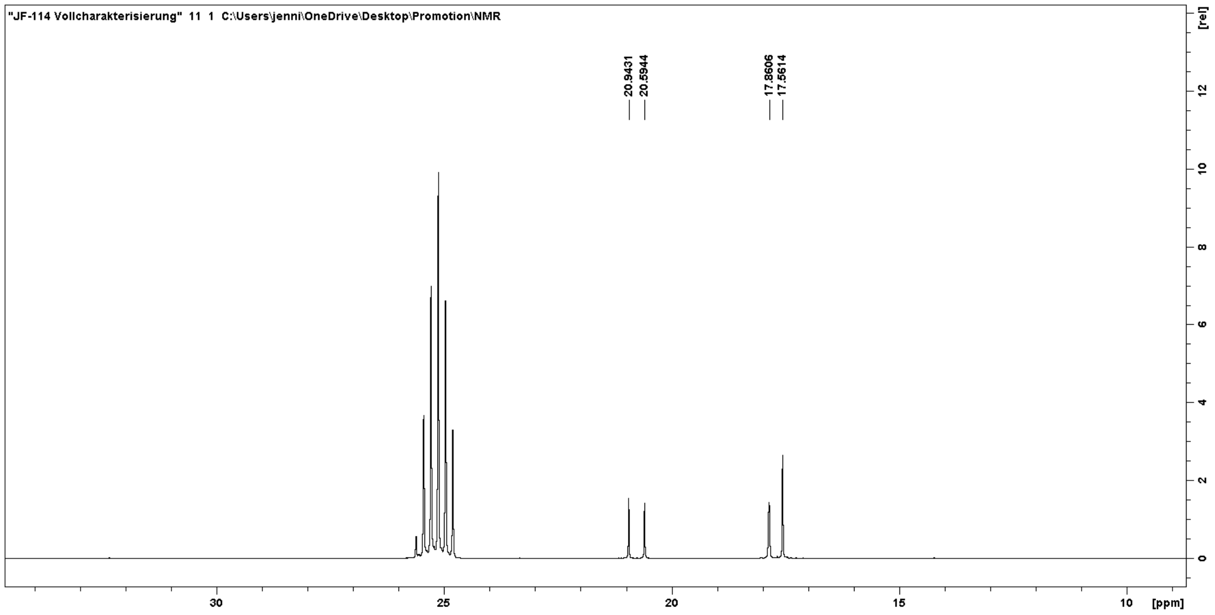

^{11}**B{^{1}H} NMR** (160 MHz, THF-*d*8) of **2b**:

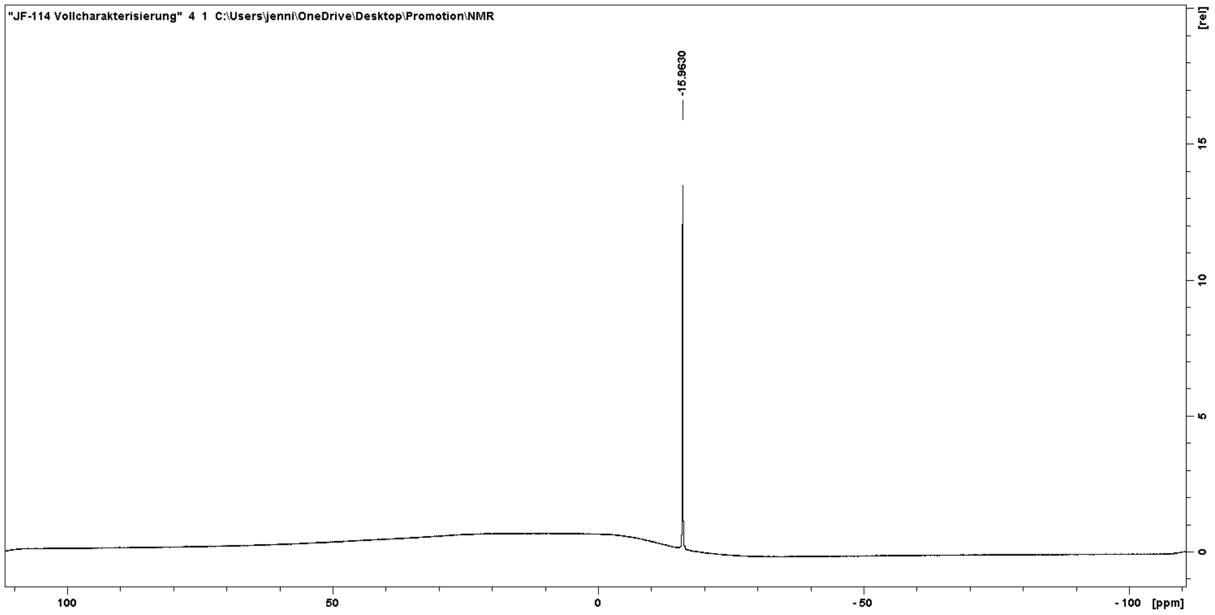

^{19}F{^{1}H} NMR (470 MHz, THF-d_8) of **2b**:

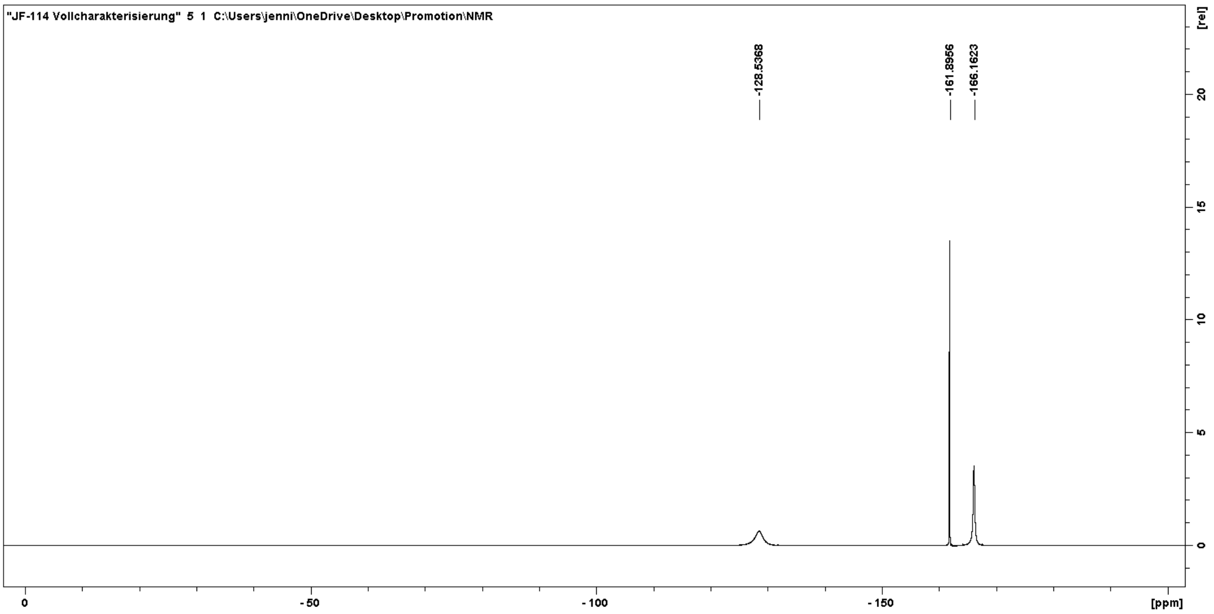

2.6. [(*m*-XyF$_6$)$_3$B(IDipp)I] (2c)

^{1}H NMR (500 MHz, THF-d_8) of **2c**:

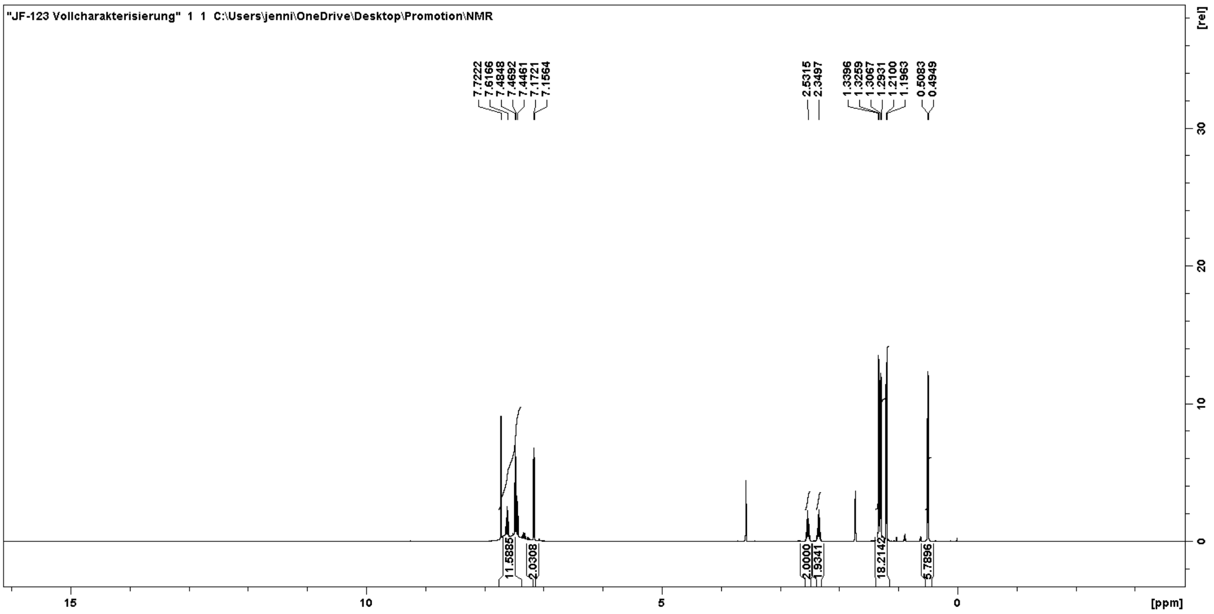

$^{13}C\{^{1}H\}$ NMR (125 MHz, THF-d_8) of **2c**:

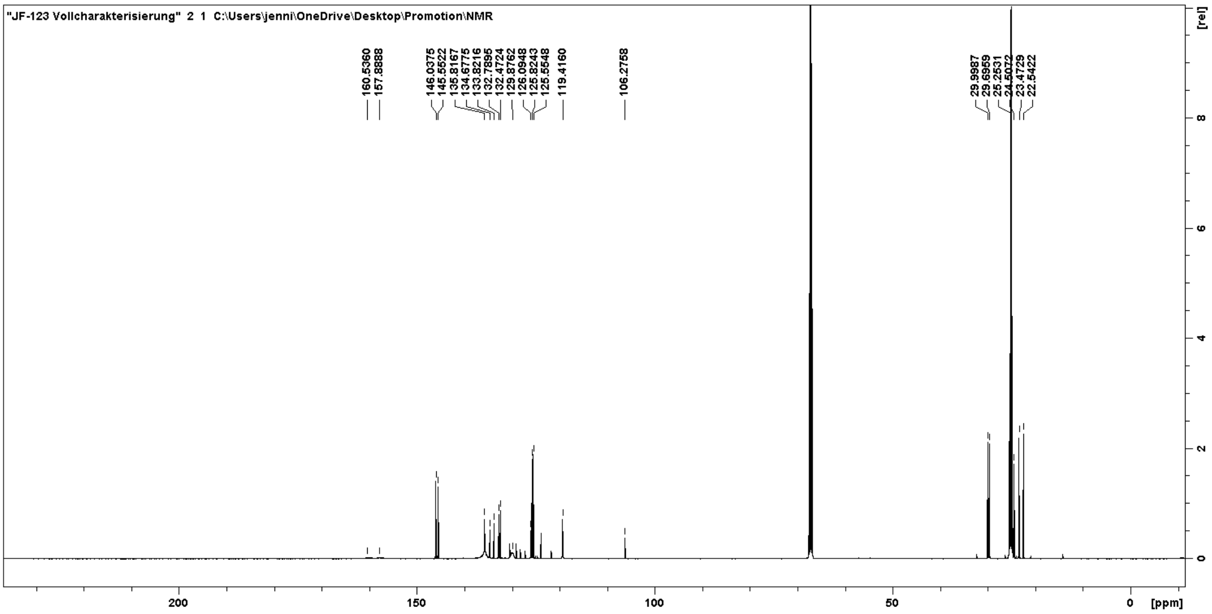

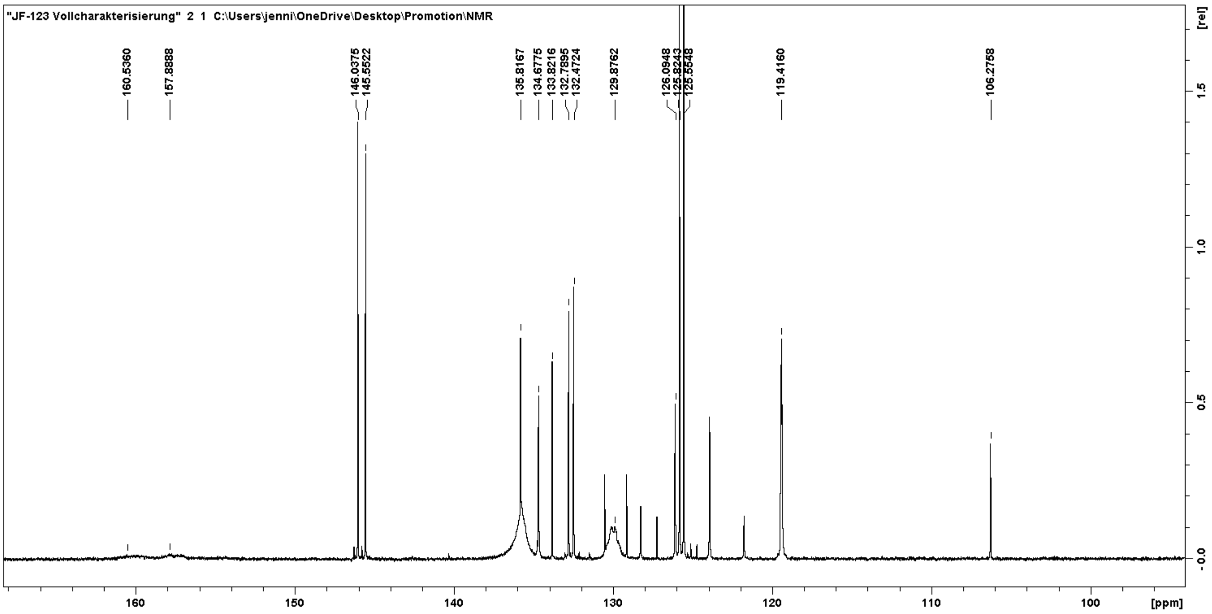

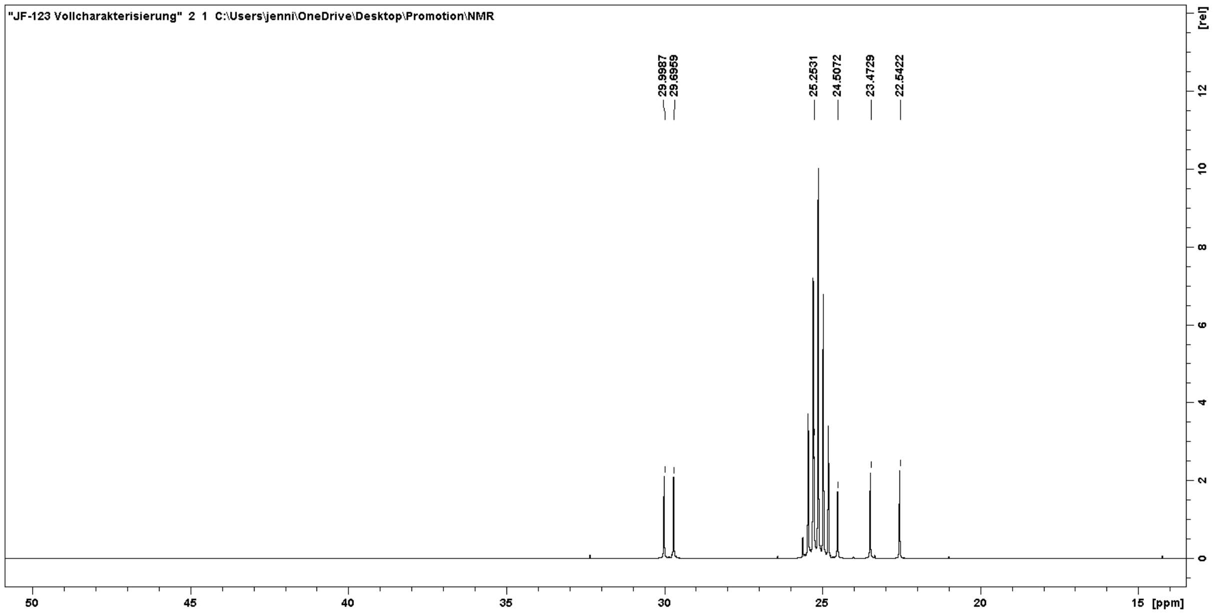

$^{11}B\{^1H\}$ NMR (128 MHz, THF-*d*8) of **2c**:

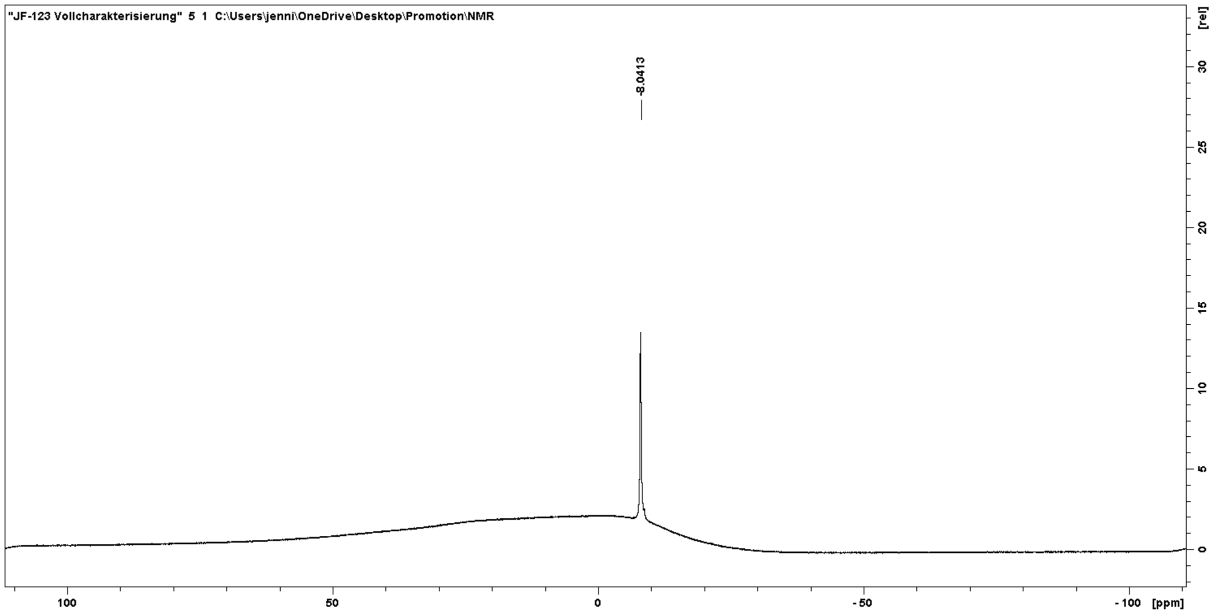

$^{19}F\{^{1}H\}$ NMR (376 MHz, THF-d_8) of **2c**:

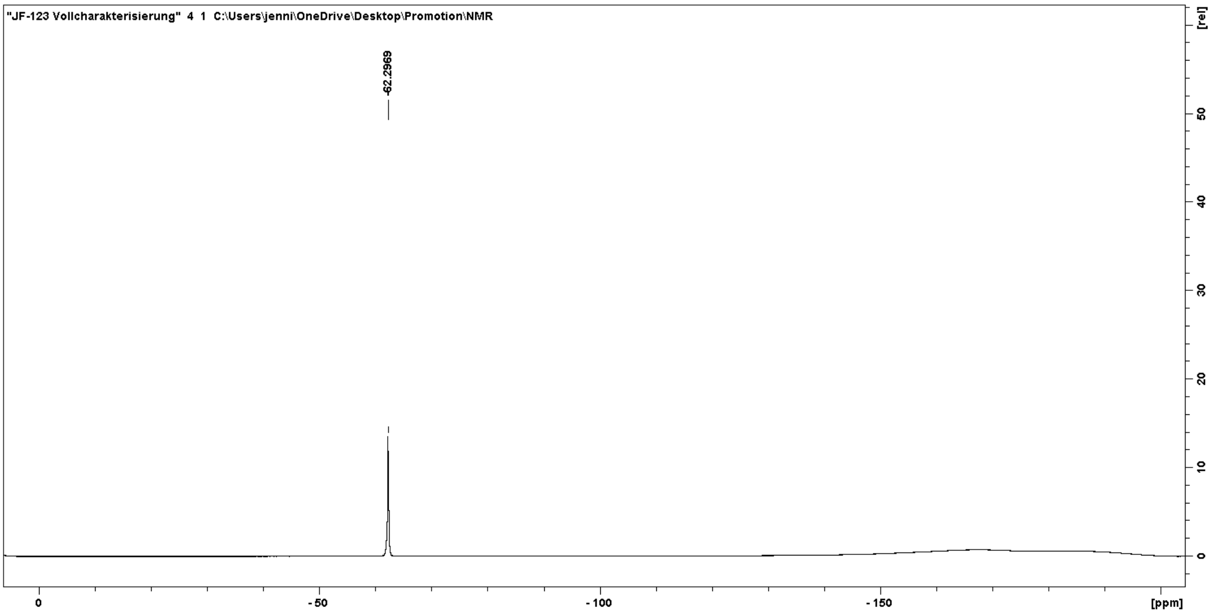

2.7. [(WCA-IDipp)Br] (3a)

^{1}H NMR (500 MHz, THF-d_8) of **3a**:

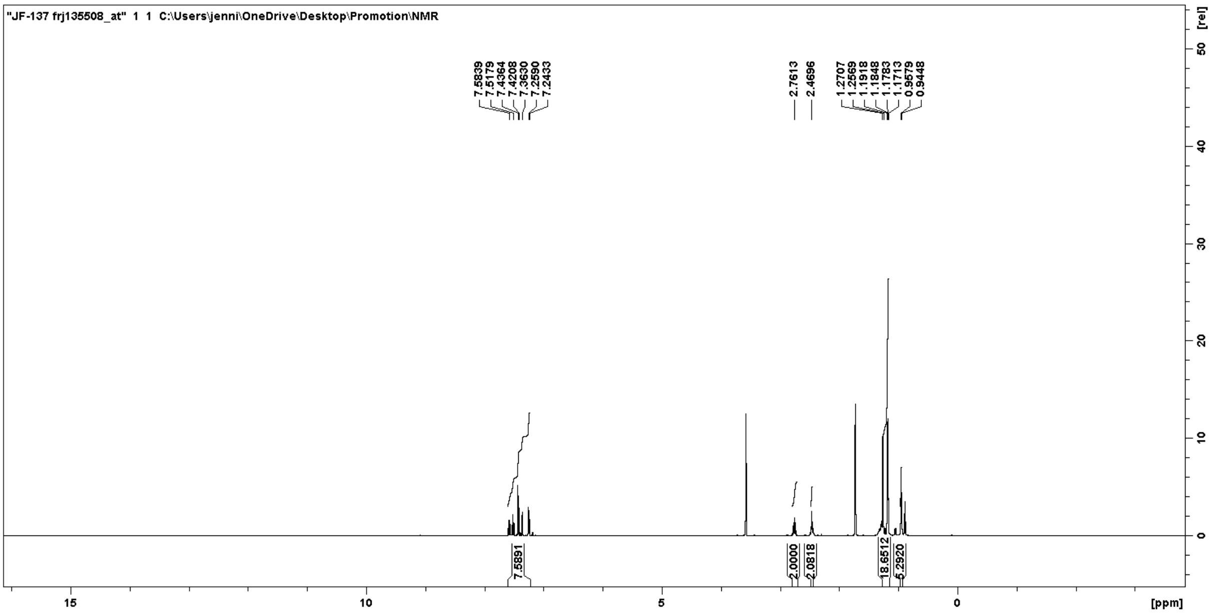

$^{13}C\{^1H\}$ NMR (125 MHz, THF-d_8) of **3a**:

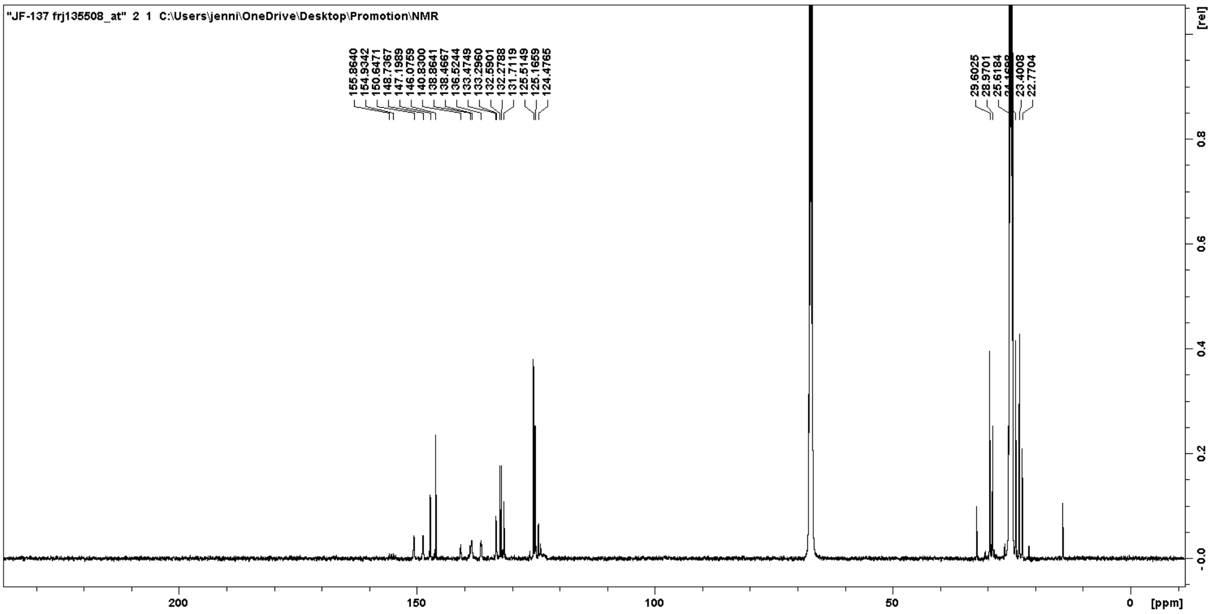

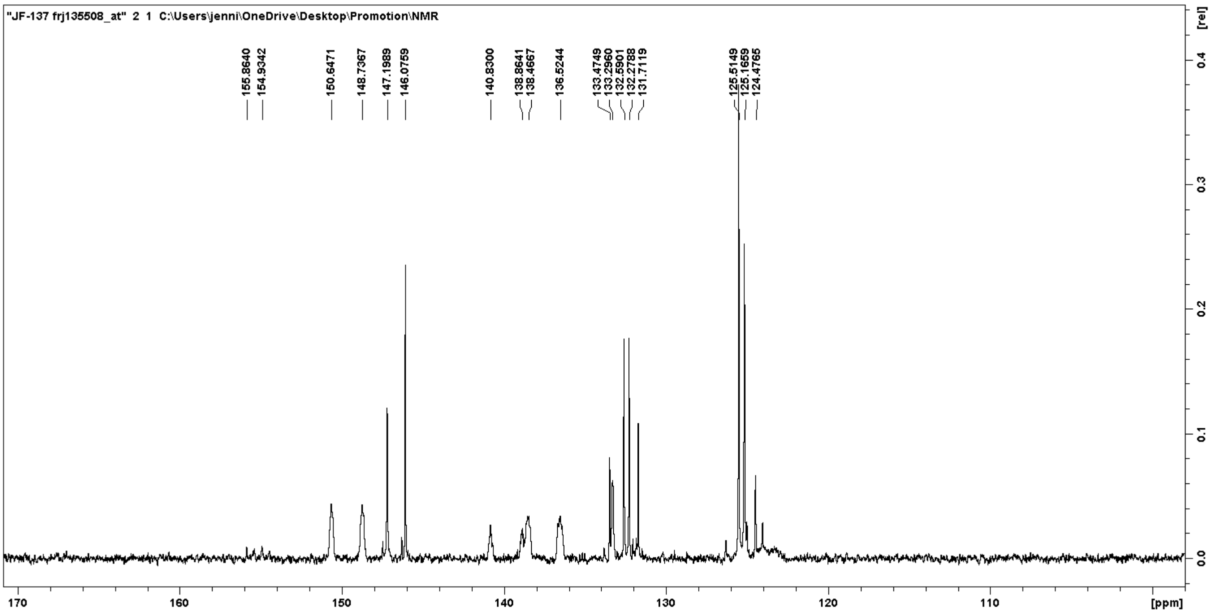

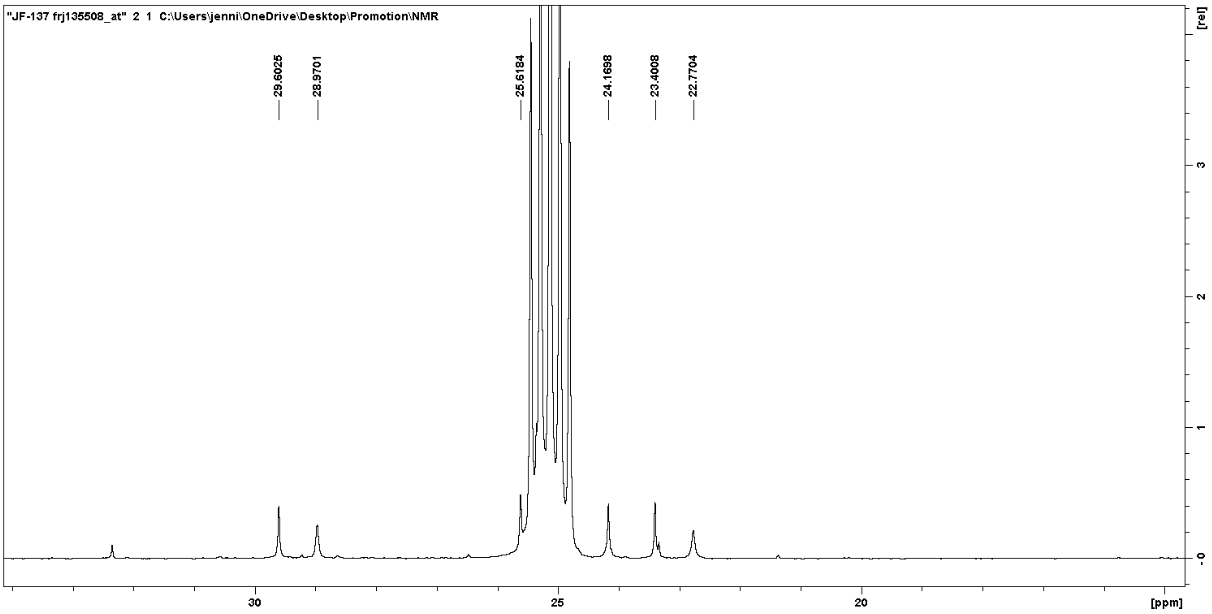

^{11}B{^{1}H} NMR (160 MHz, THF-d_8) of **3a**:

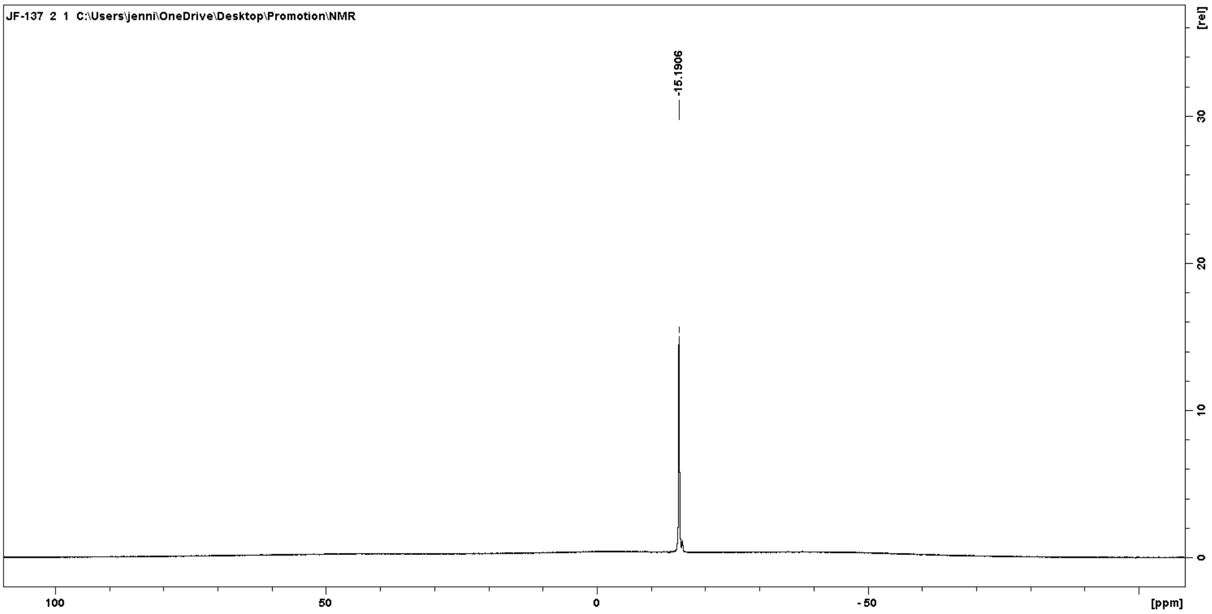

$^{19}F\{^{1}H\}$ NMR (470 MHz, THF-d_8):

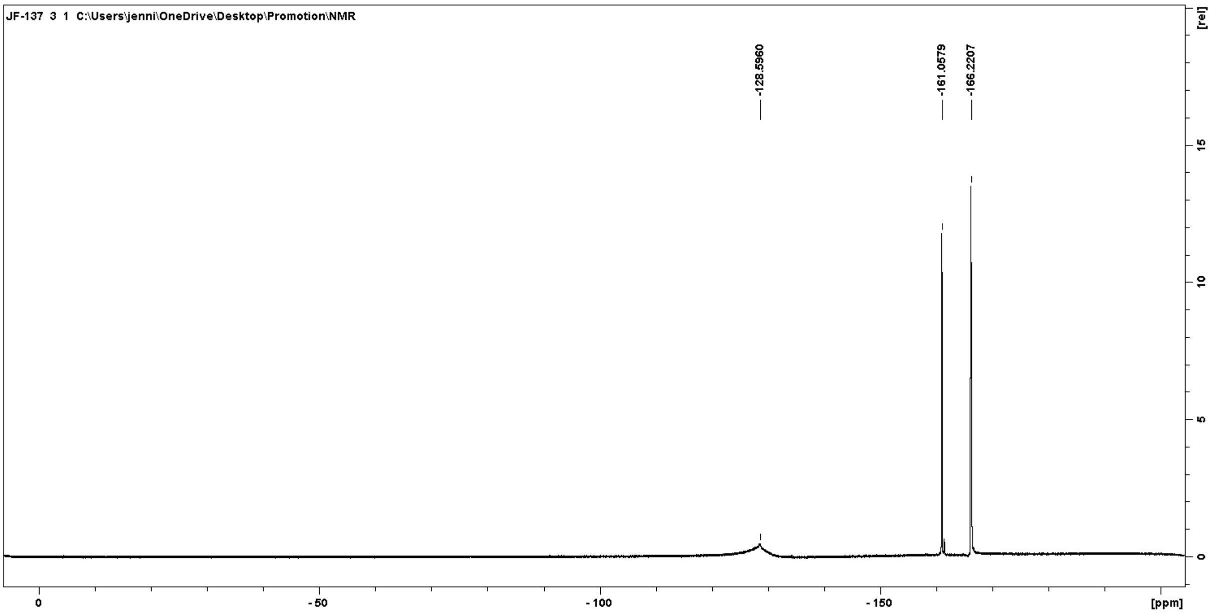

2.8. [(WCA-IMes)Br] (3b)

^{1}H NMR (300 MHz, THF-d_8) of **3b**:

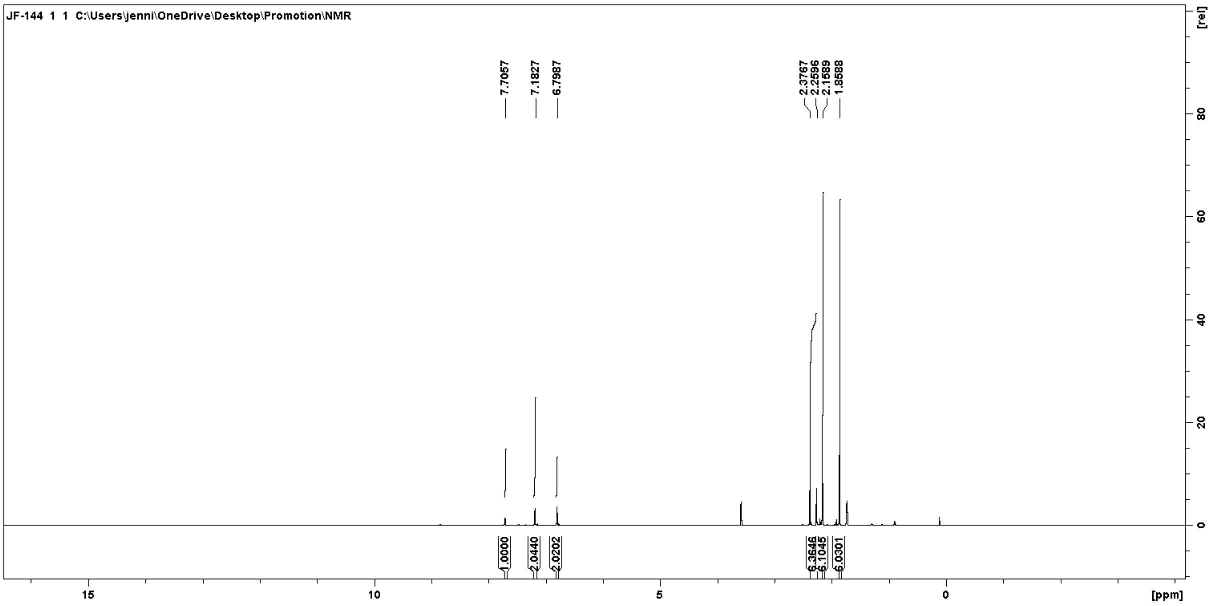

$^{13}C\{^{1}H\}$ NMR (100 MHz, THF-d_8) of **3b**: Due to the long measurement already small signals of the protonated carbene **9b** arose and are now present in the $^{13}C\{^{1}H\}$ NMR spectra. Only the picked signals are relevant for compound **3b**.

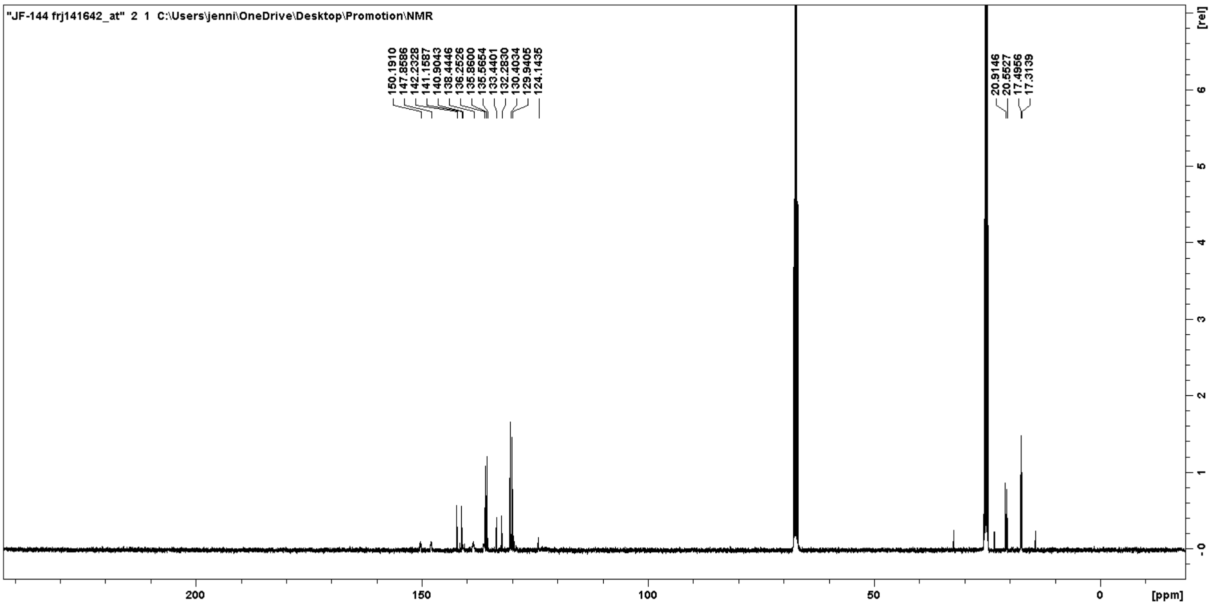

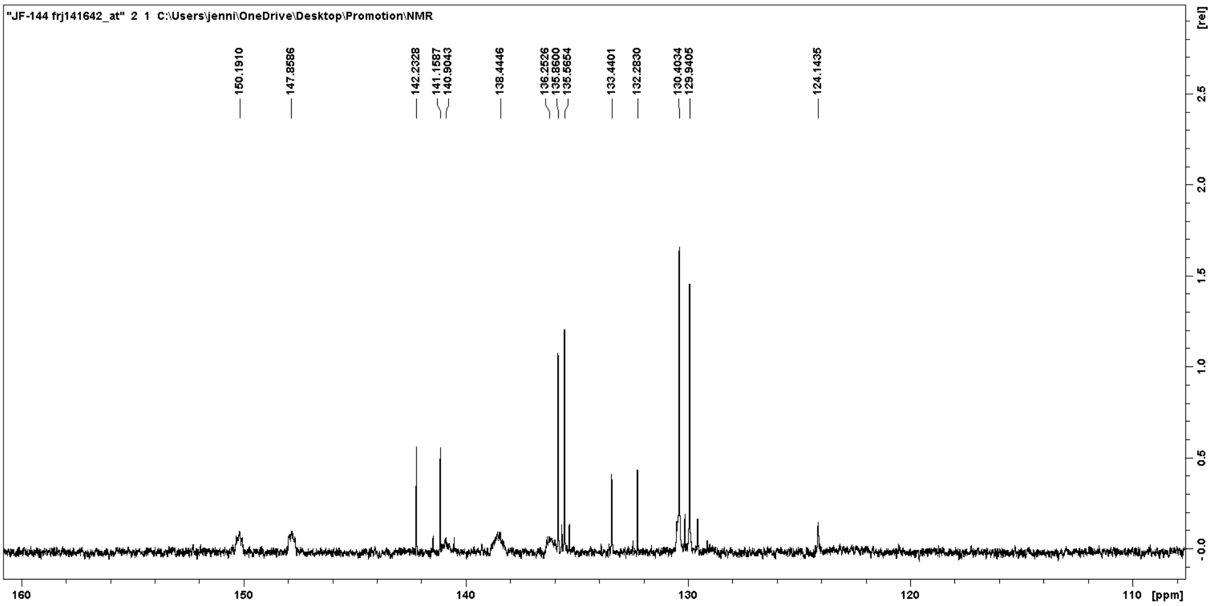

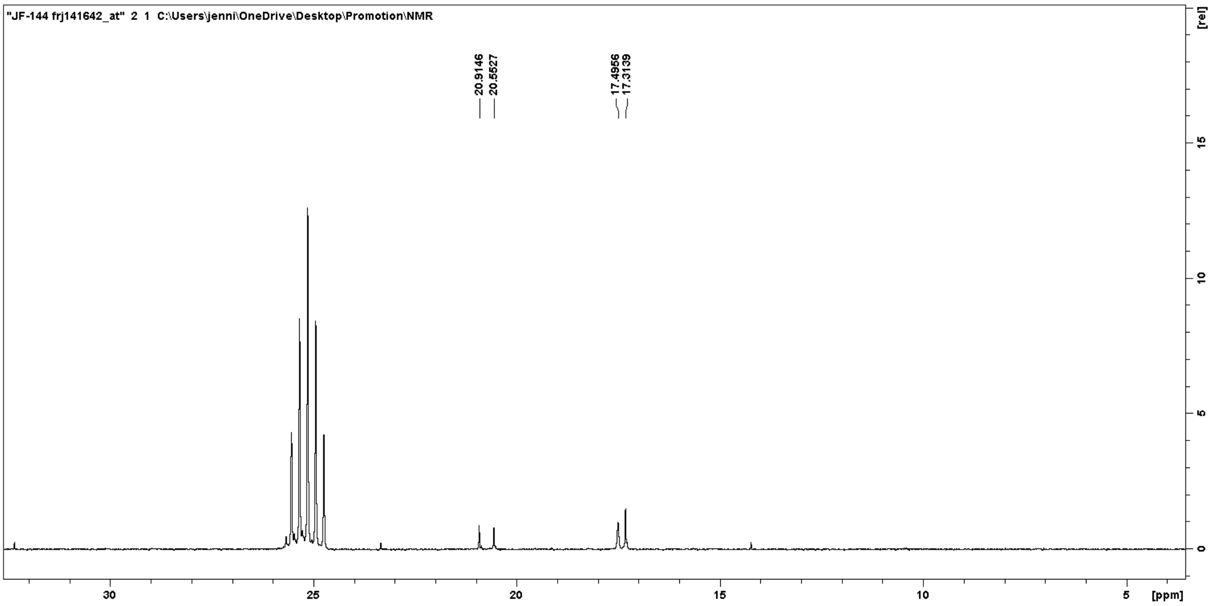

^{11}**B{^{1}H} NMR** (128 MHz, THF-d_8) of **3b**:

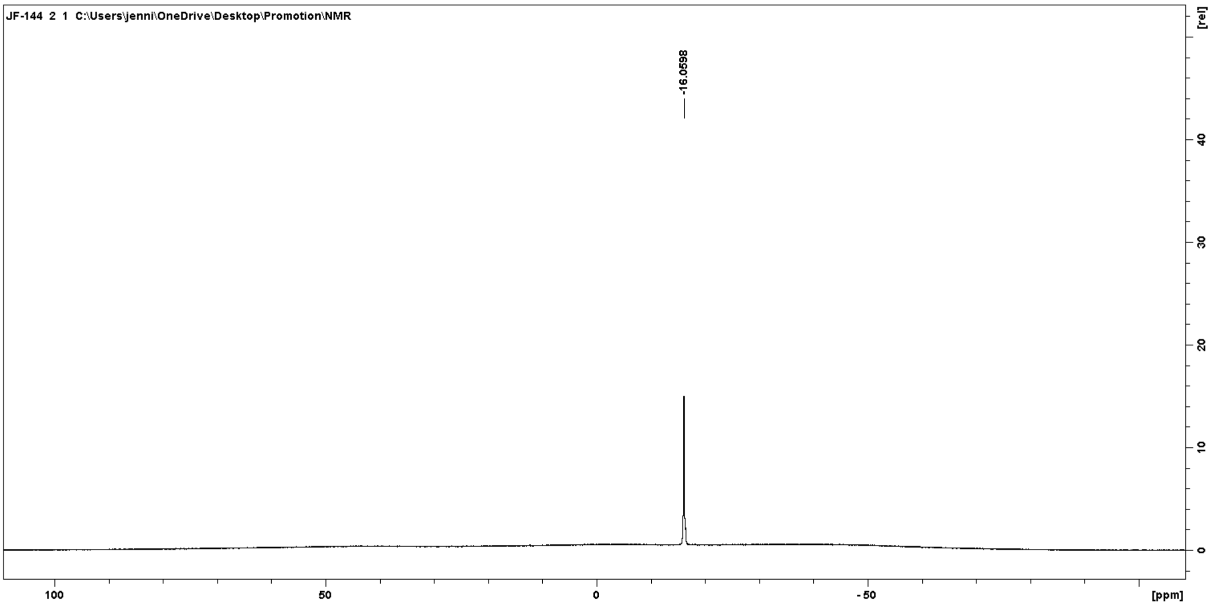

^{19}F{^{1}H} **NMR** (188 MHz, THF-d_8) of **3b**:

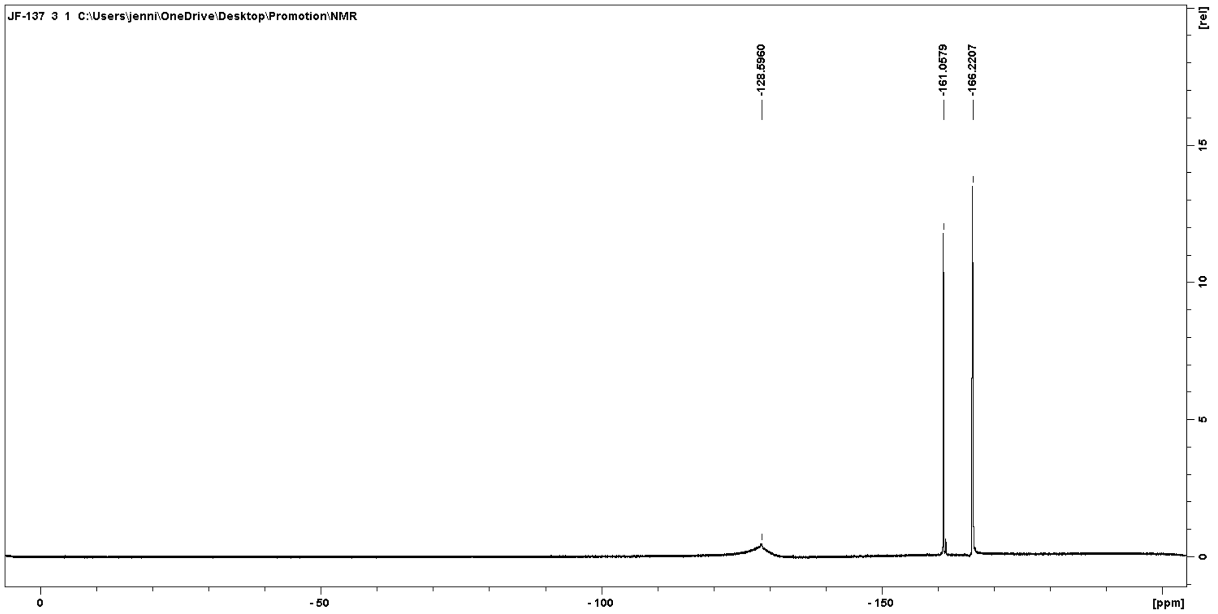

2.9. [(WCA-IDipp)Cl] (4)

^{1}H **NMR** (500 MHz, THF-d_8) of **4**:

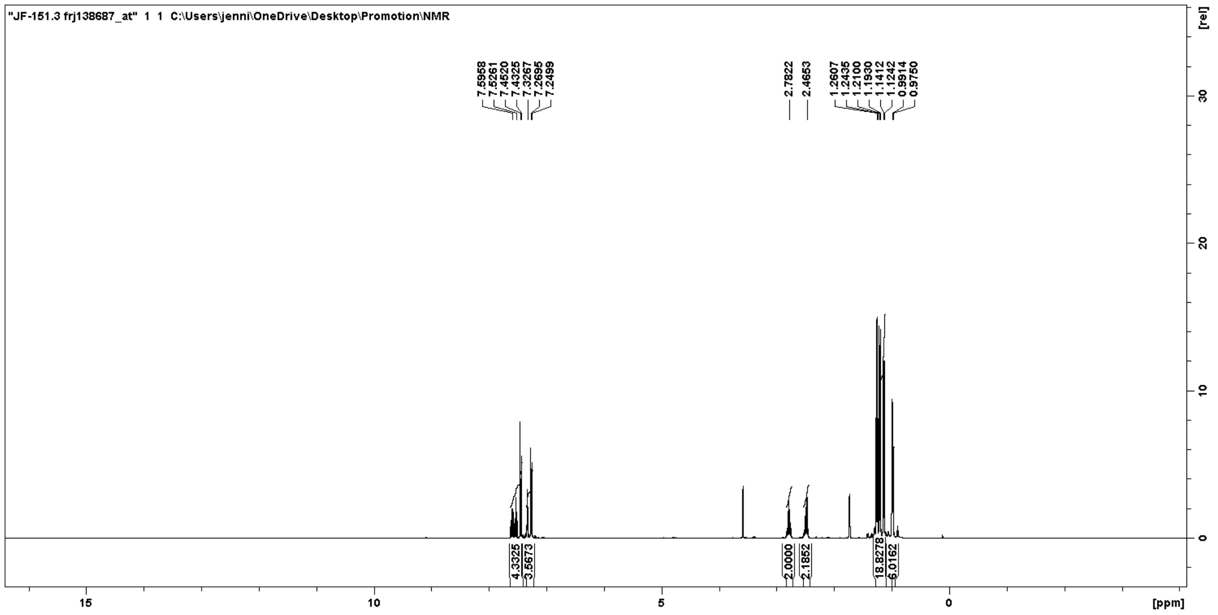

$^{13}C\{^1H\}$ NMR (125 MHz, THF-d_8) of **4**:

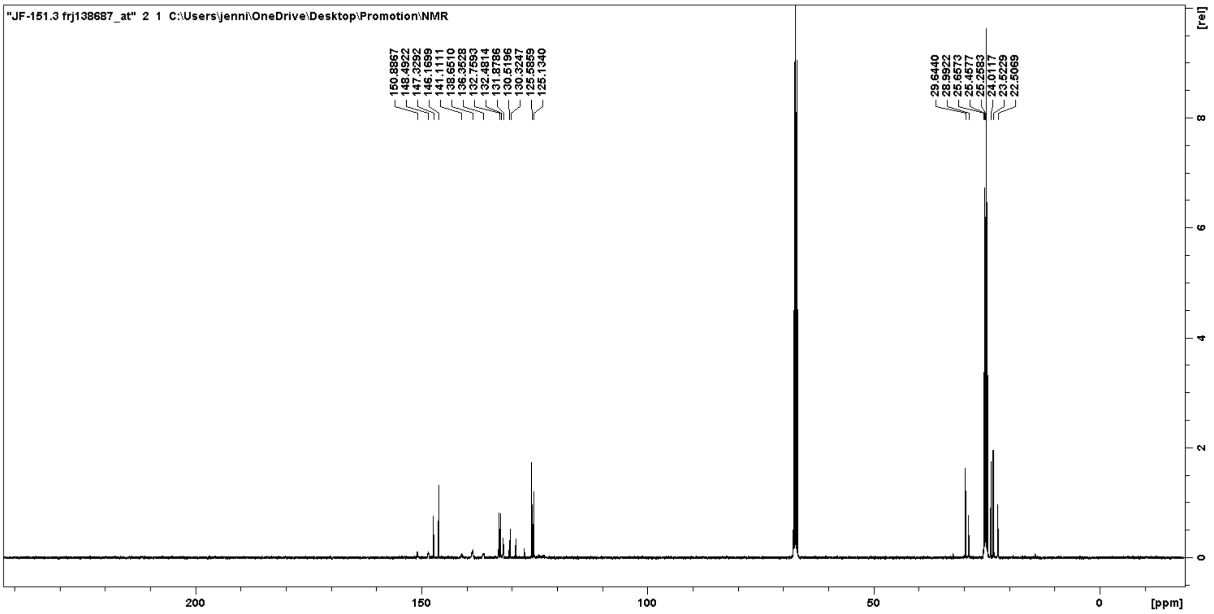

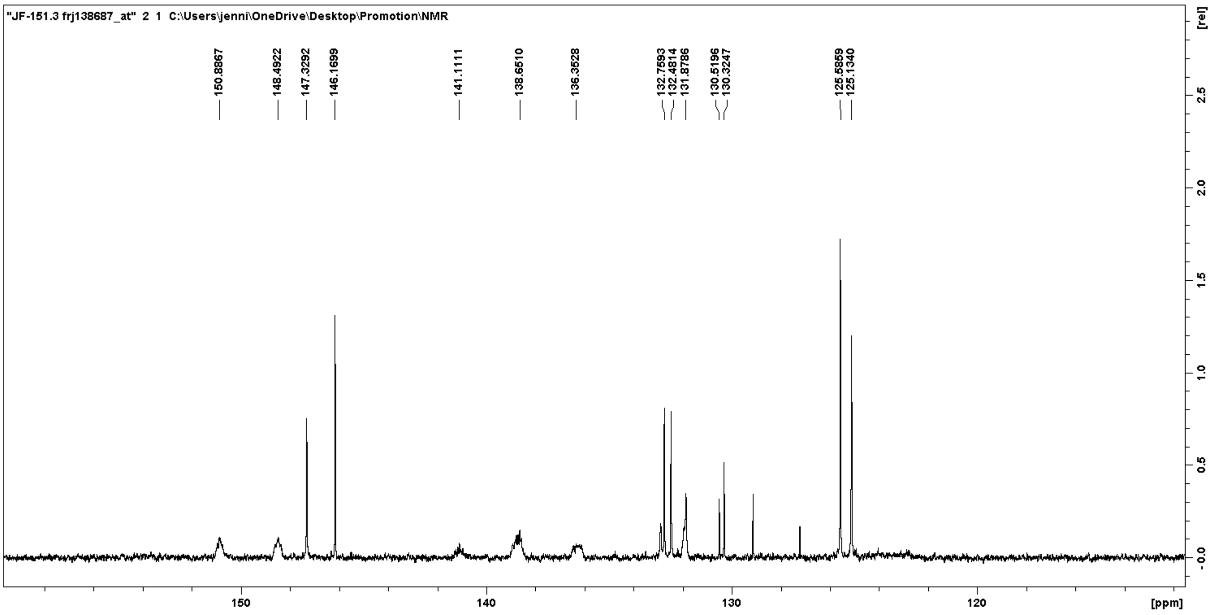

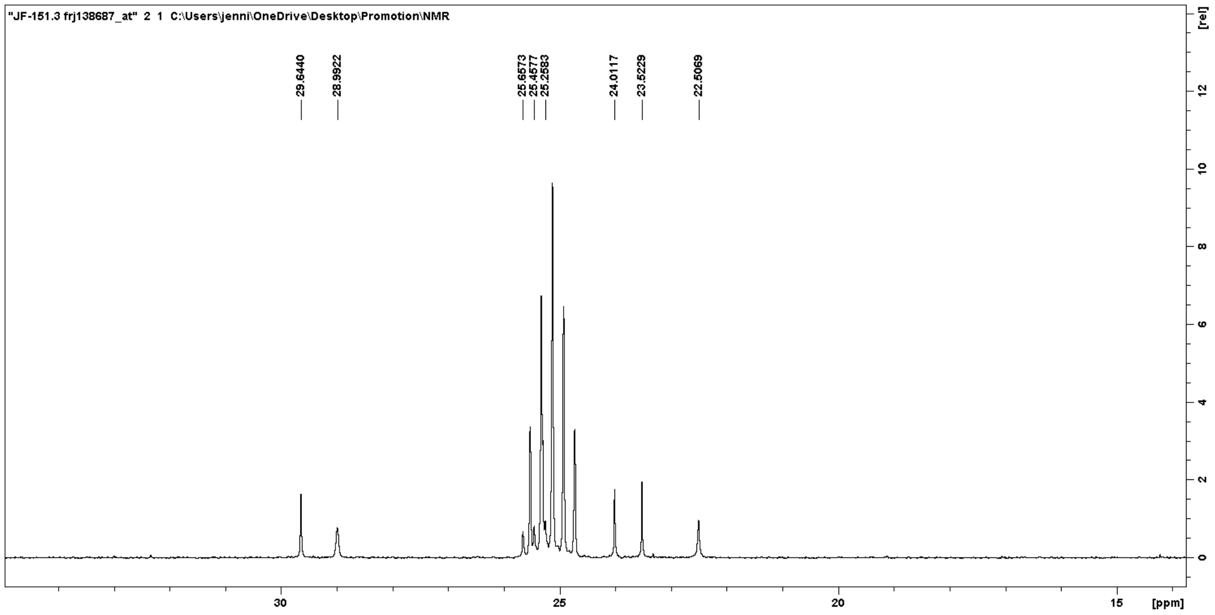

$^{11}B\{^{1}H\}$ NMR (128 MHz, THF-*d*8) of **4**:

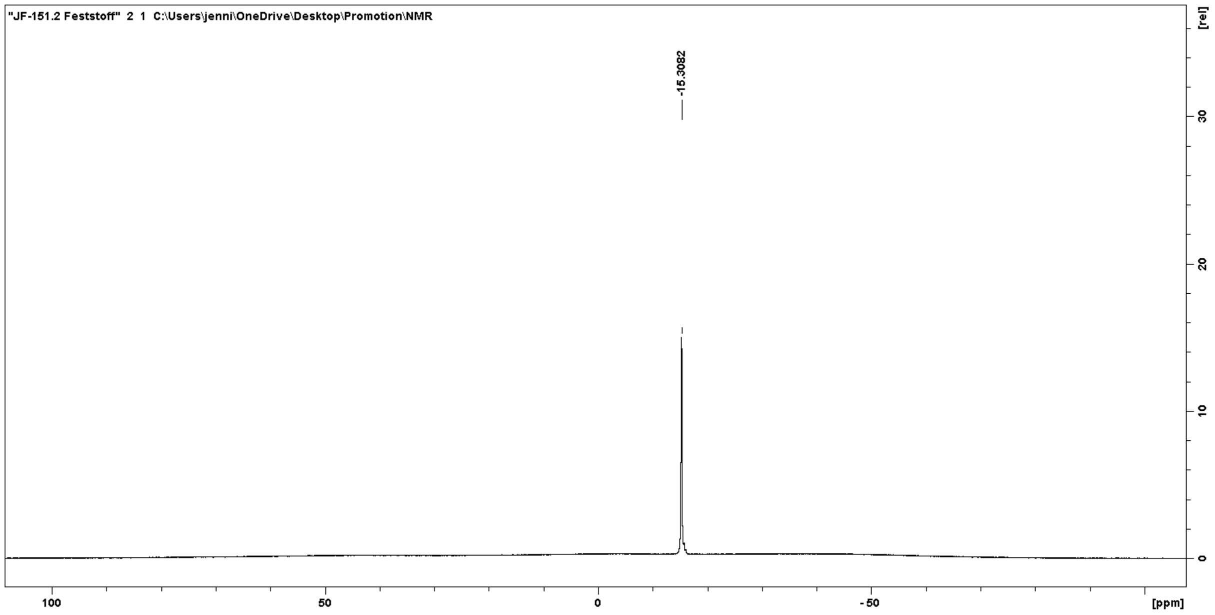

^{19}**F{^{1}H} NMR** (376 MHz, THF-*d*$_8$) of **4**:

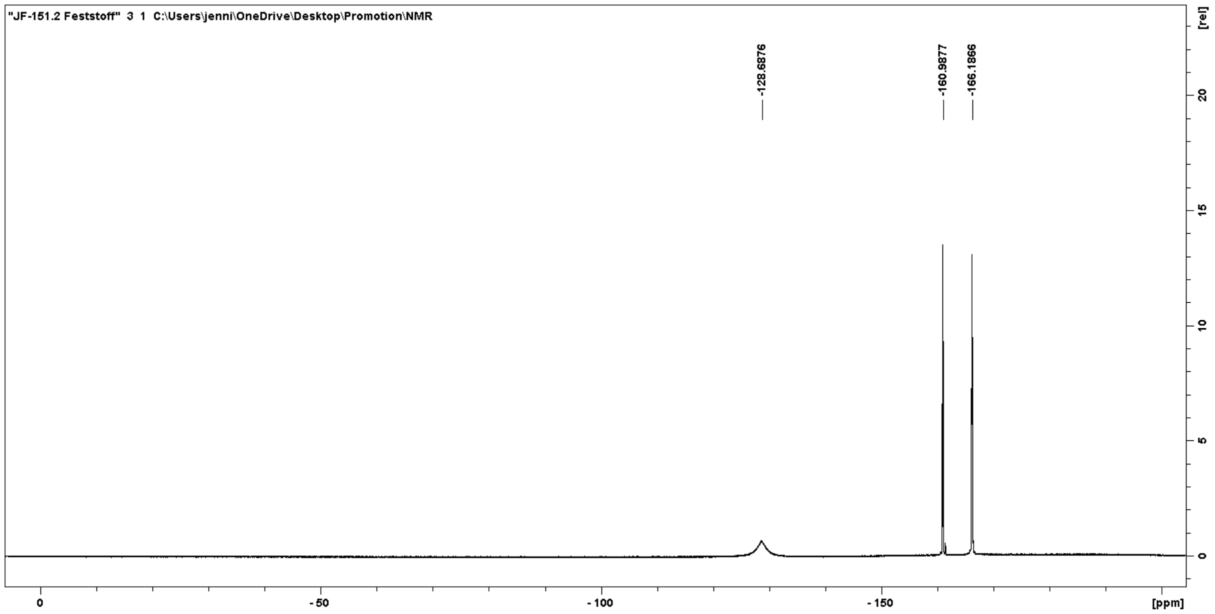

2.10. [(WCA-IDipp)I(IDipp)] (5a):

In the presented NMR spectra already small signals arise beside the desired signals due to quick decomposition of **5a** in solution, which lead to the formation of the protonated carbene **9a**, of which the predominant signals are marked with a exemplarily, and imidazoliumsalts like [IDipp$_2$I]I. Only the picked signals are relevant for **5a**.

^{1}**H NMR** (500 MHz, THF-*d*$_8$) of **5a**:

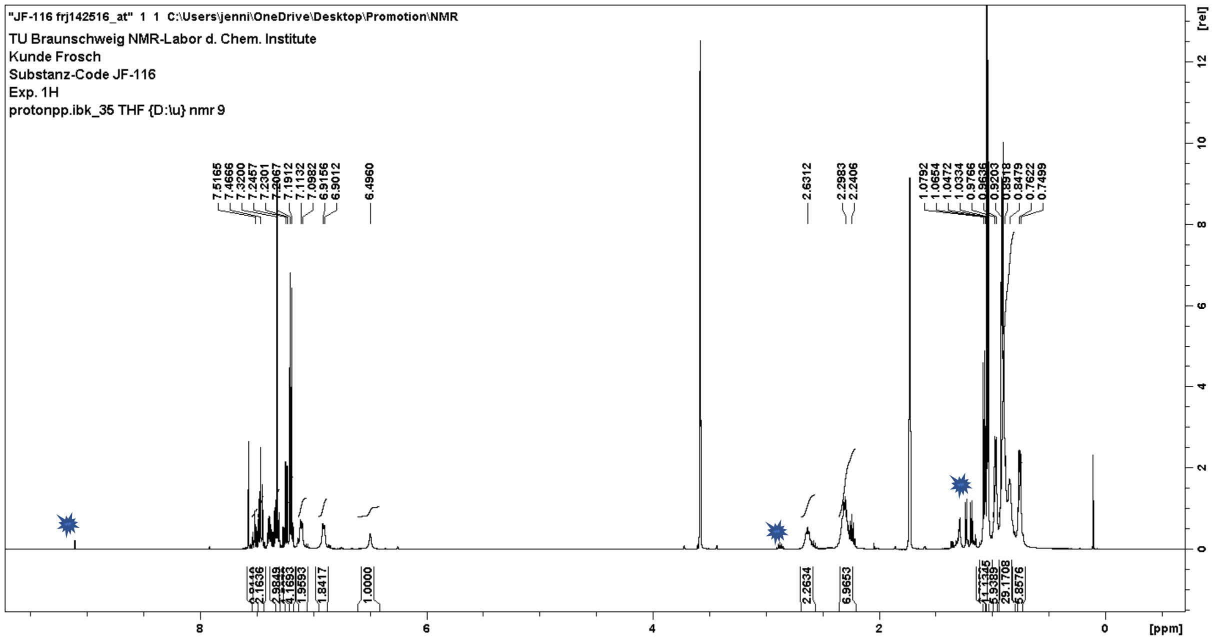

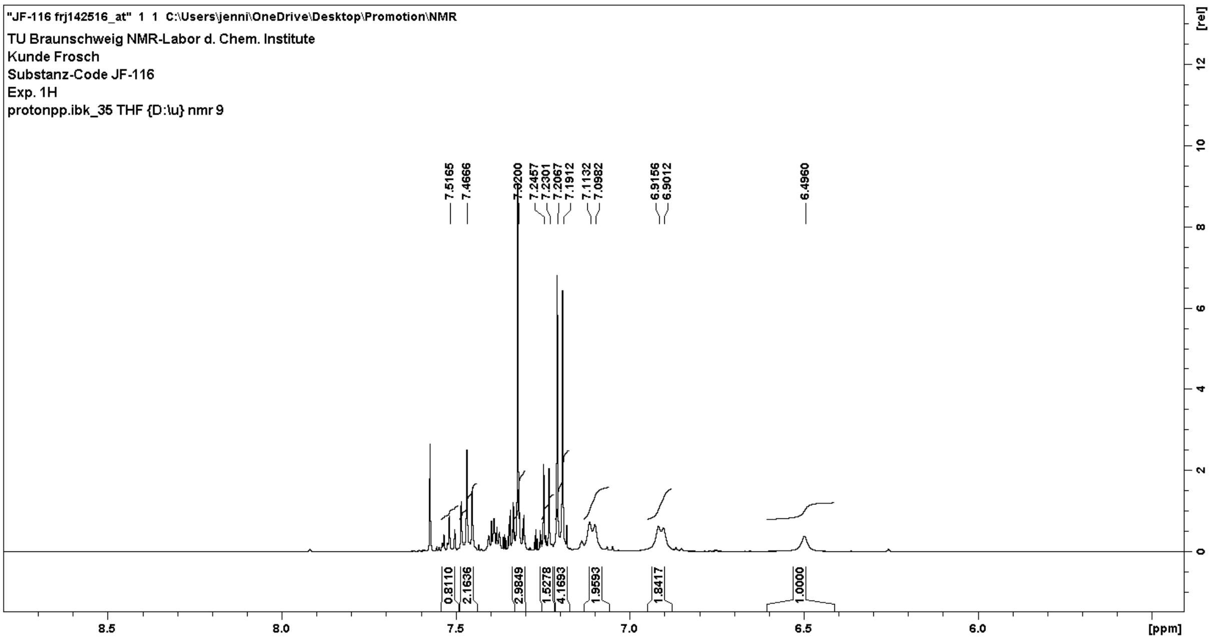

"JF-116 frj142516_at" 1 1 C:\Users\jenni\OneDrive\Desktop\Promotion\NMR
TU Braunschweig NMR-Labor d. Chem. Institute
Kunde Frosch
Substanz-Code JF-116
Exp. 1H
protonpp.ibk_35 THF {D:\u} nmr 9
7.5165
7.4666
7.3200
7.2457
7.2301
7.2067
7.1912
7.1132
7.0982
6.9156
6.9012
6.4960
0.8110
2.1636
2.9849
1.5278
4.1693
1.9593
1.8417
1.0000
[ppm]
[rel]

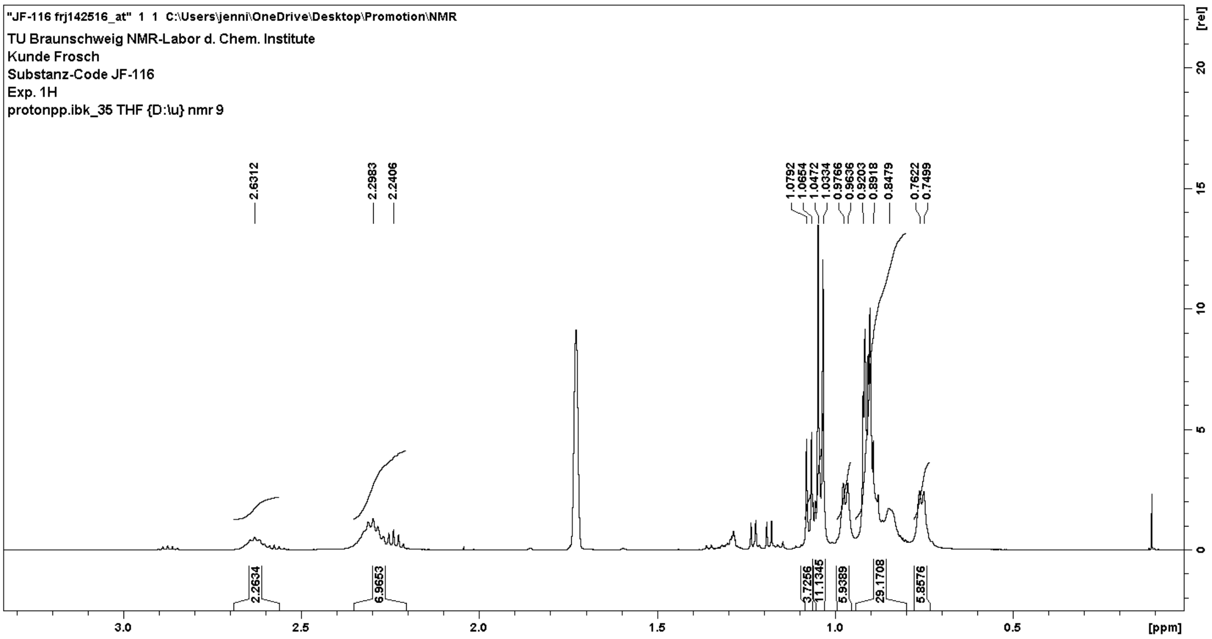

"JF-116 frj142516_at" 1 1 C:\Users\jenni\OneDrive\Desktop\Promotion\NMR
TU Braunschweig NMR-Labor d. Chem. Institute
Kunde Frosch
Substanz-Code JF-116
Exp. 1H
protonpp.ibk_35 THF {D:\u} nmr 9
2.6312
2.2983
2.2406
1.0792
1.0654
1.0472
1.0334
0.9766
0.9636
0.9203
0.8918
0.8479
0.7622
0.7499
2.2634
6.9653
3.7256
11.1345
5.9389
29.1708
5.8576
[ppm]
[rel]

^{13}**C NMR** (125 MHz, THF-d_8) of **5a**:

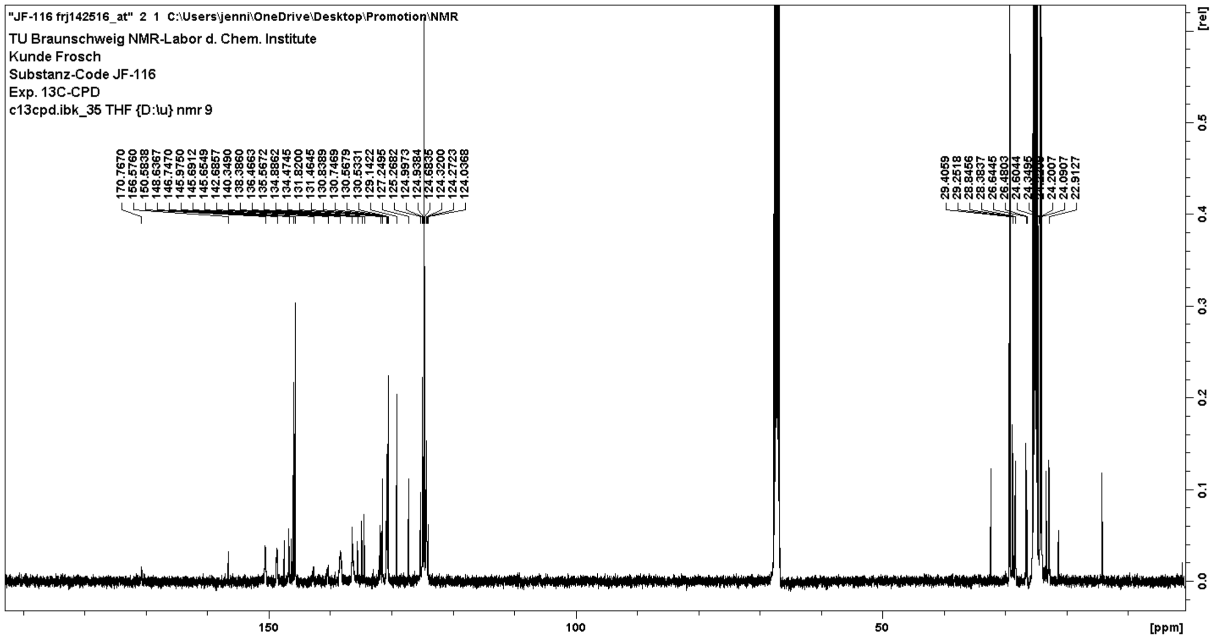

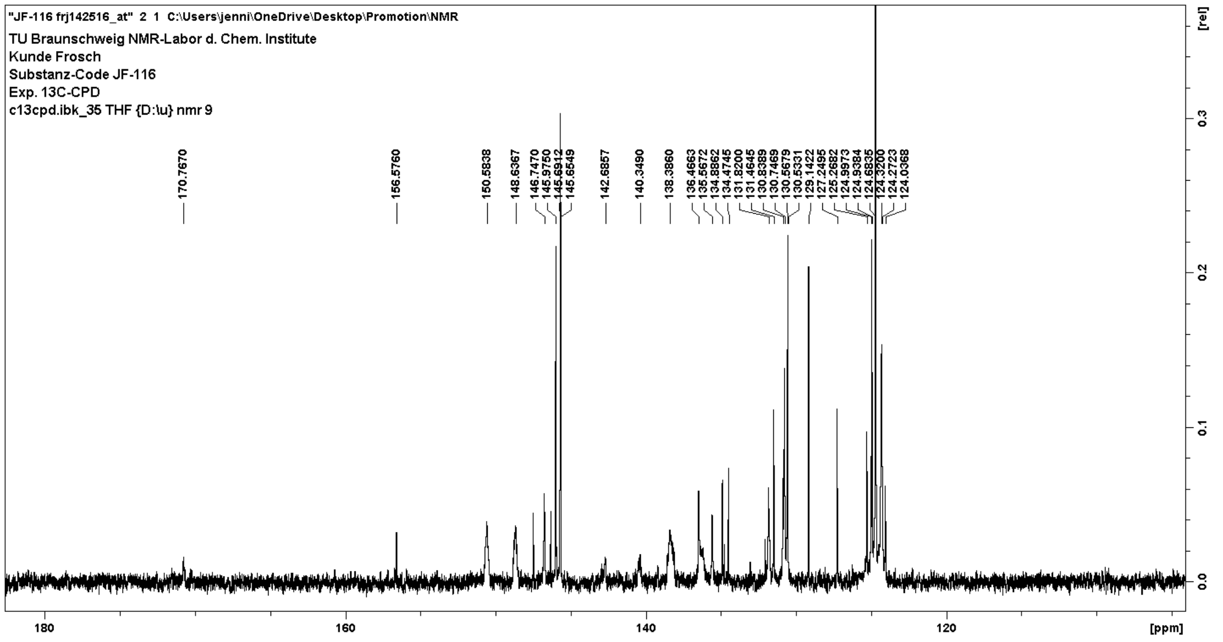

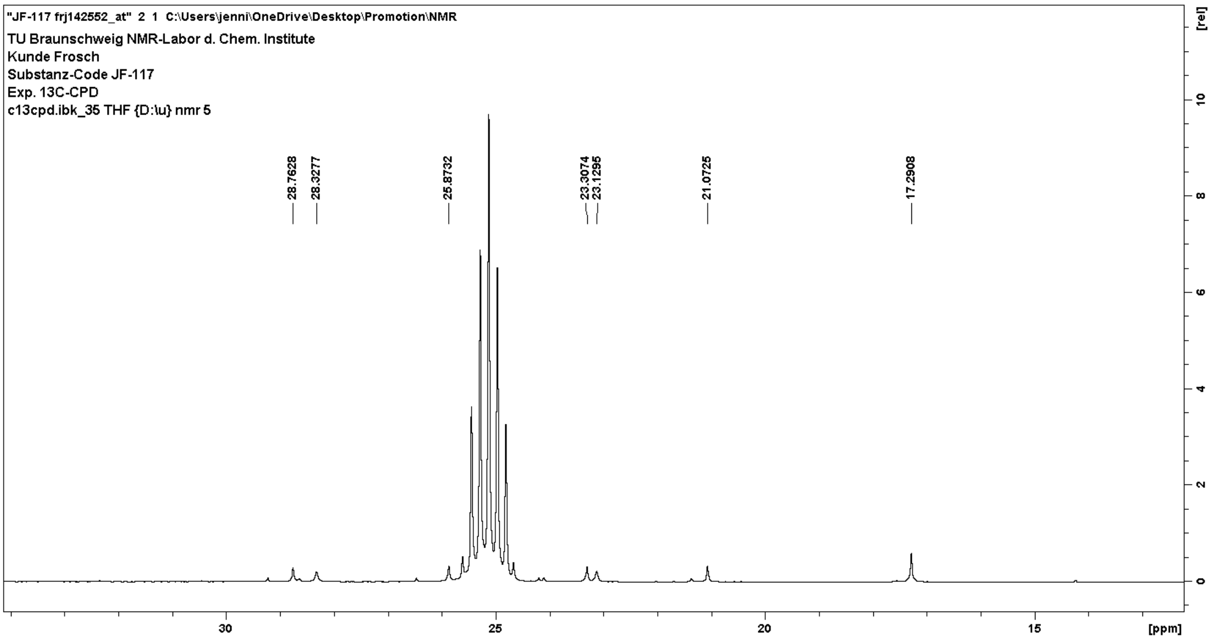

^{11}**B{^{1}H} NMR** (160 MHz, THF-d_8) of **5a**:

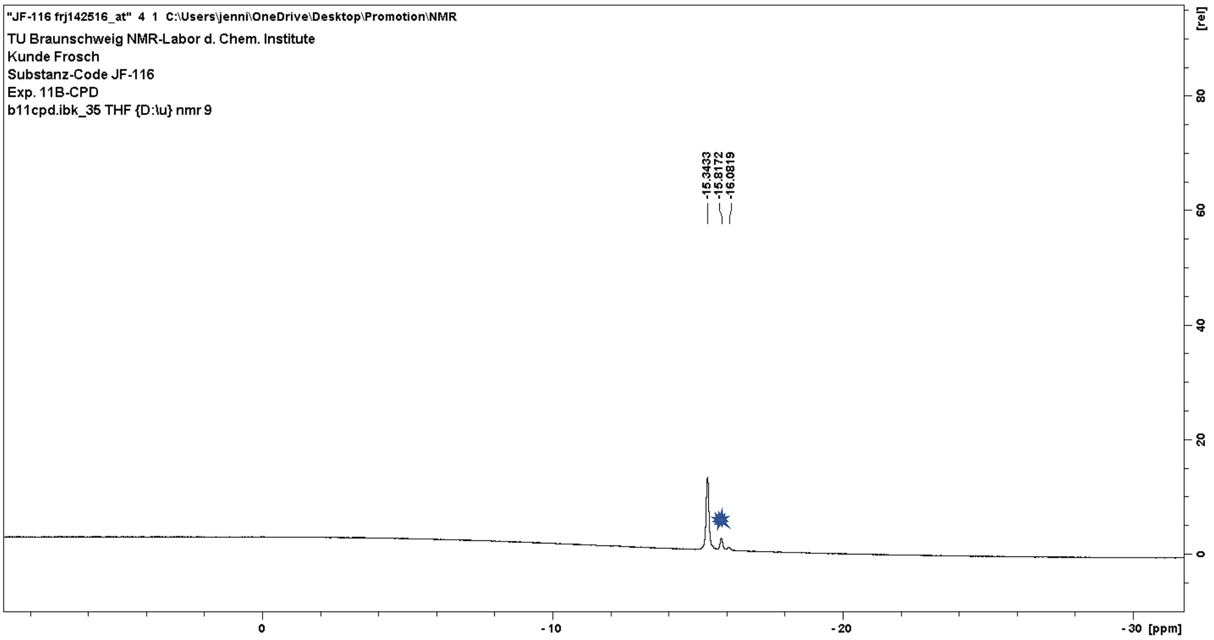

^{19}**F{^{1}H} NMR** (470 MHz, THF-d_8) of **5a**:

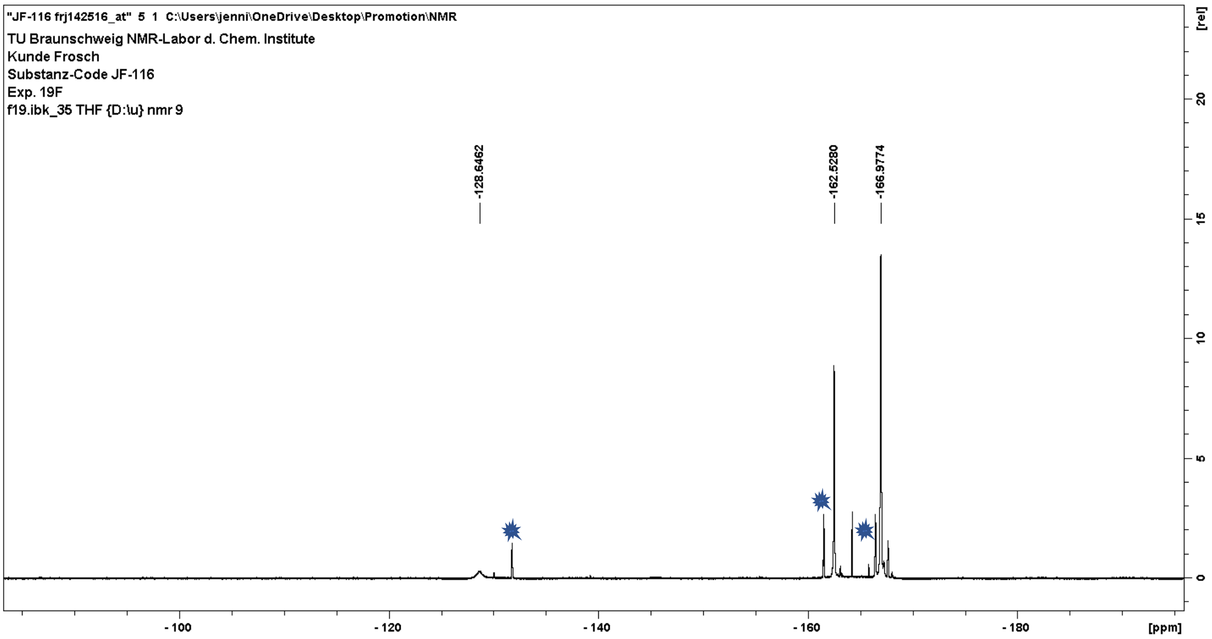

2.11. [(WCA-IDipp)I(IMes)] (5b):

In the presented NMR spectra already small signals arise beside the desired signals due to quick decomposition of **5b** in solution, which lead to the formation of the protonated carbene **9a** and imidazoliumsalts.

^{1}**H NMR** (500 MHz, THF-d_8) of **5b**:

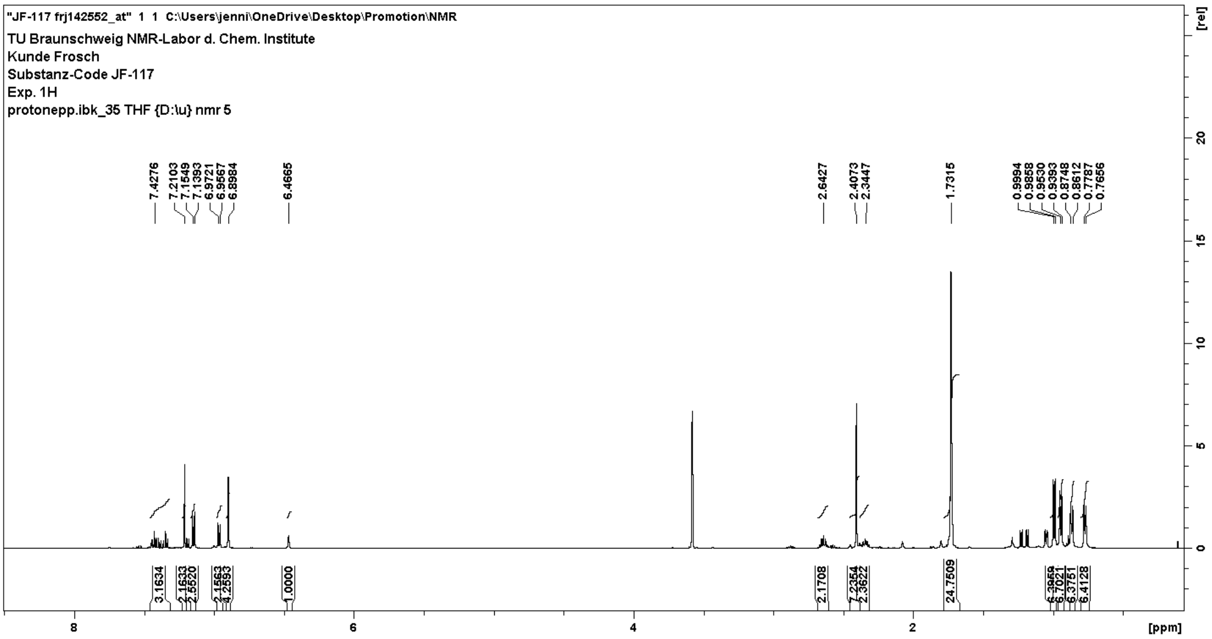

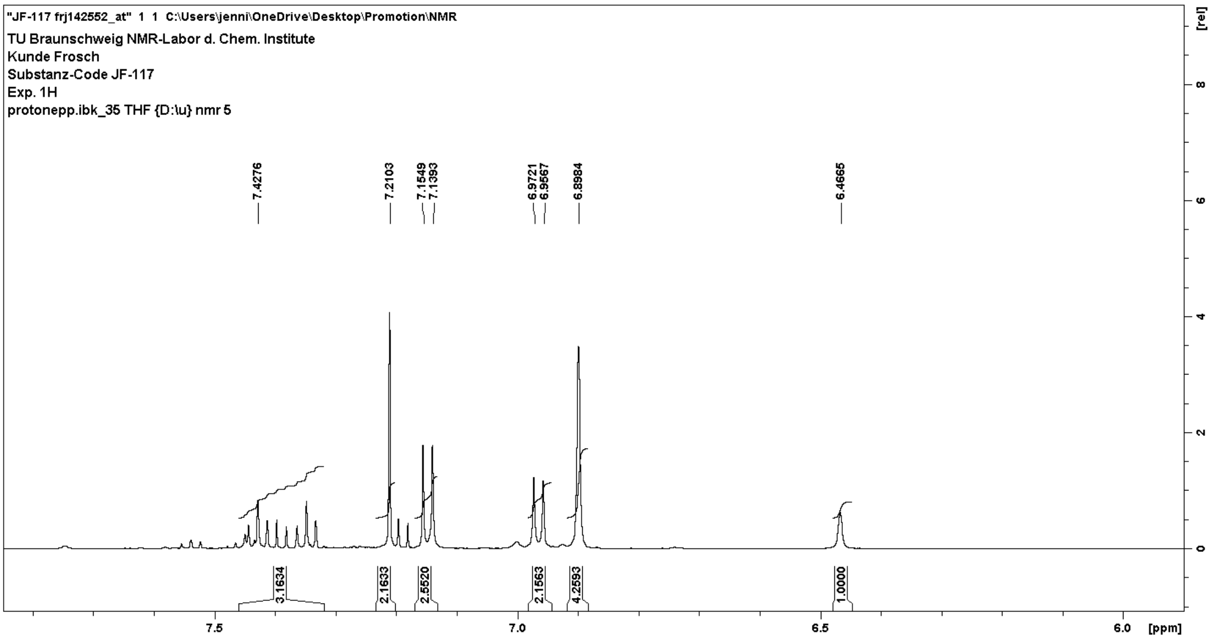
"JF-117 frj142552_at" 1 1 C:\Users\jenni\OneDrive\Desktop\Promotion\NMR
TU Braunschweig NMR-Labor d. Chem. Institute
Kunde Frosch
Substanz-Code JF-117
Exp. 1H
protonepp.ibk_35 THF {D:\u} nmr 5
7.4276
7.2103
7.1549
7.1393
6.9721
6.9567
6.8984
6.4665
3.1634
2.1633
2.5520
2.1563
4.2593
1.0000
7.5
7.0
6.5
6.0
[ppm]
[rel]

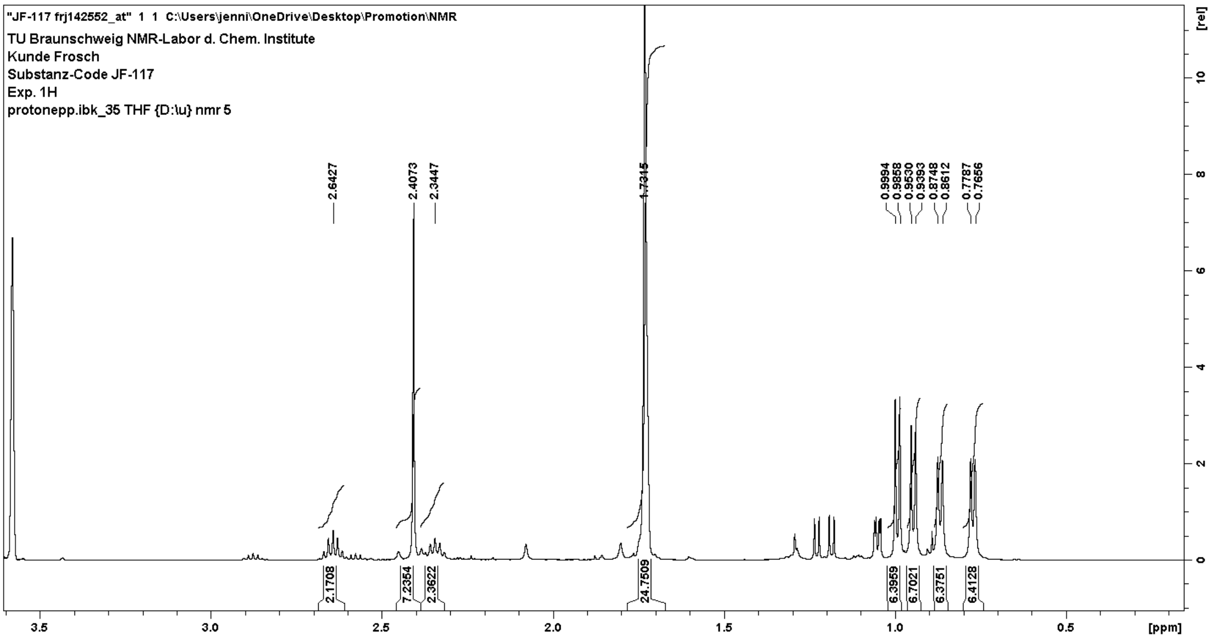
"JF-117 frj142552_at" 1 1 C:\Users\jenni\OneDrive\Desktop\Promotion\NMR
TU Braunschweig NMR-Labor d. Chem. Institute
Kunde Frosch
Substanz-Code JF-117
Exp. 1H
protonepp.ibk_35 THF {D:\u} nmr 5
2.6427
2.4073
2.3447
1.7315
0.9994
0.9858
0.9530
0.9393
0.8748
0.8612
0.7787
0.7656
2.1708
7.2354
2.3622
24.7509
6.3959
6.7021
6.3751
6.4128
3.5
3.0
2.5
2.0
1.5
1.0
0.5
[ppm]
[rel]

^{13}C NMR (125 MHz, THF-d_8) of **5b**:

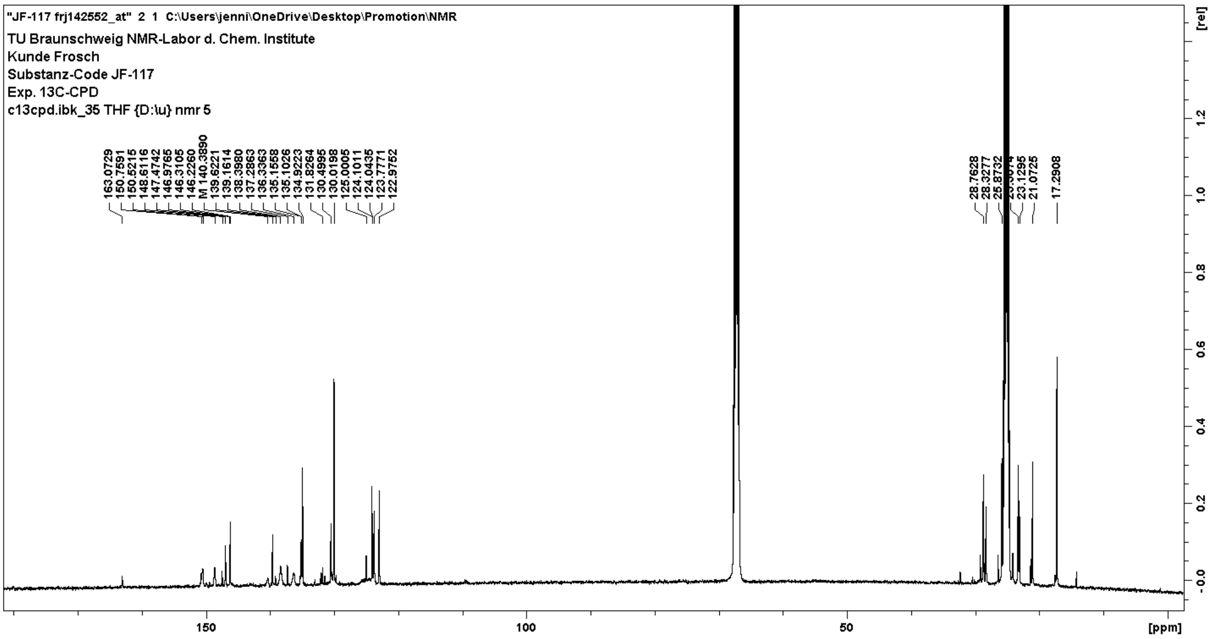

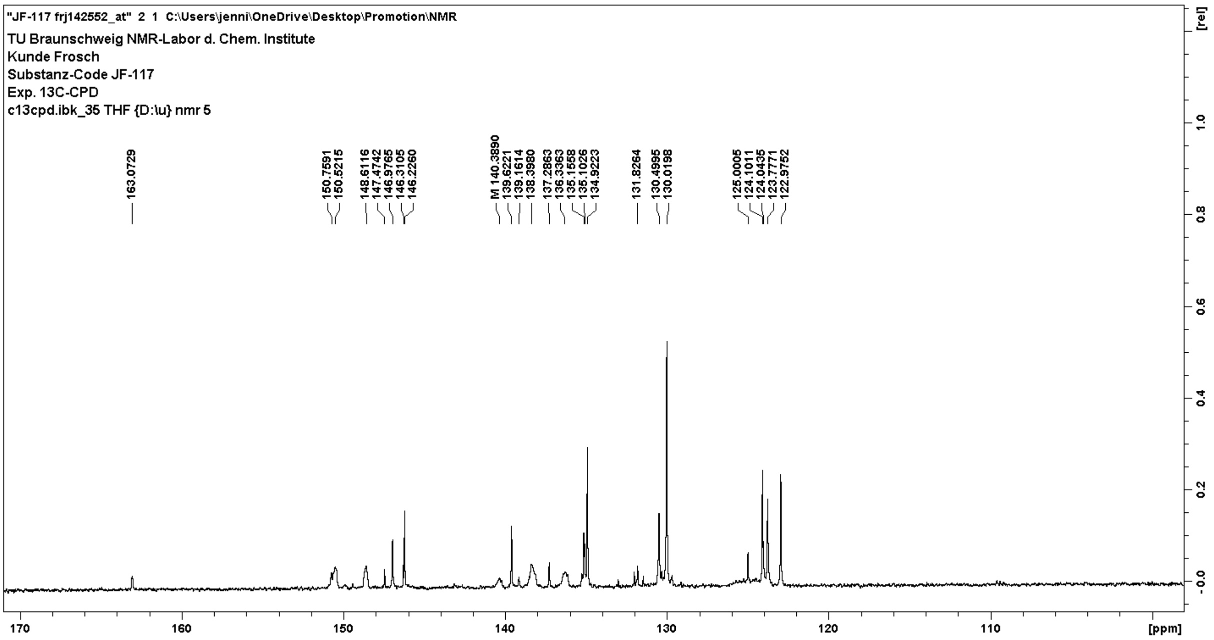

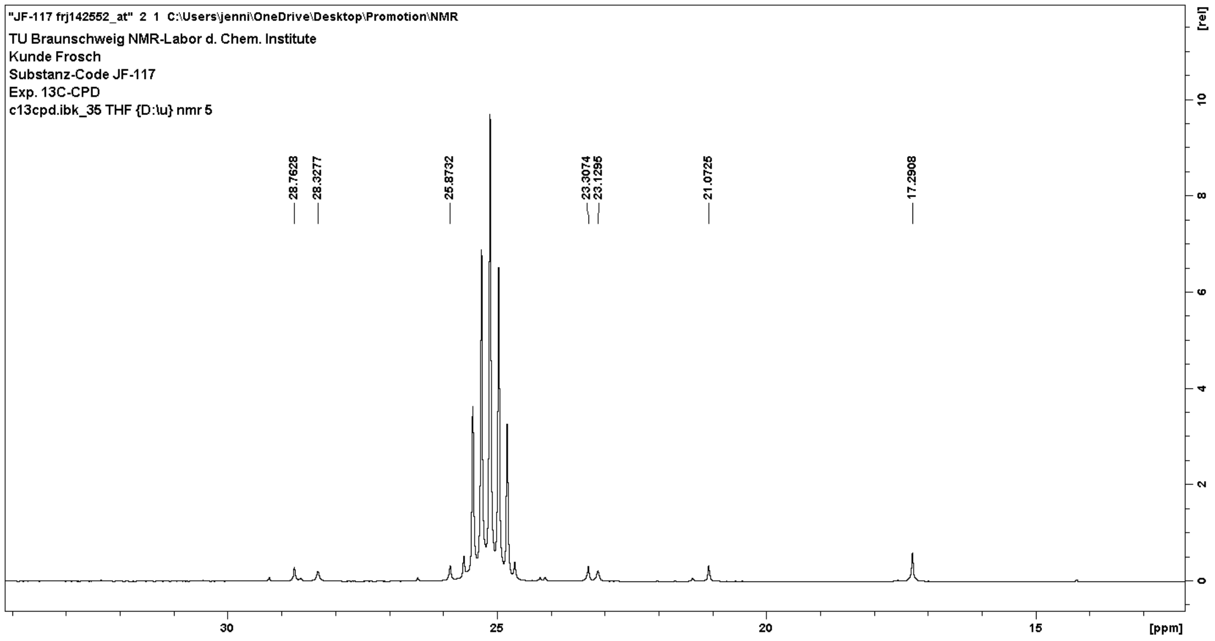

^{11}**B{^{1}H} NMR** (160 MHz, THF-d_8) of **5b**:

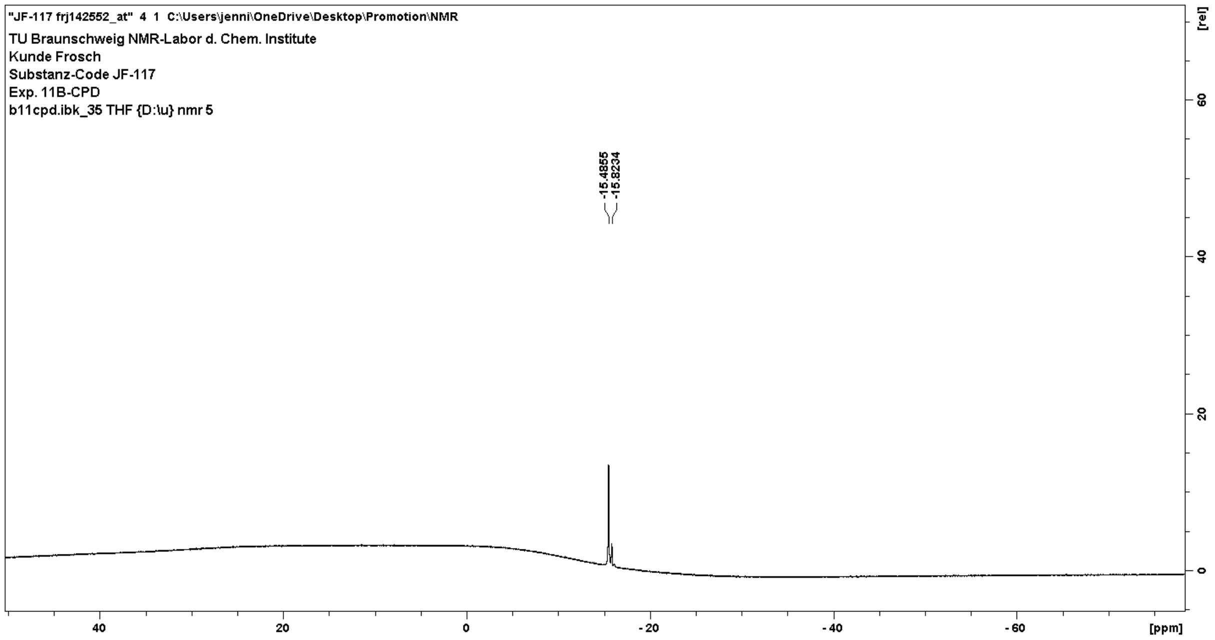

^{19}F{^{1}H} NMR (470 MHz, THF-d_8) **5b**:

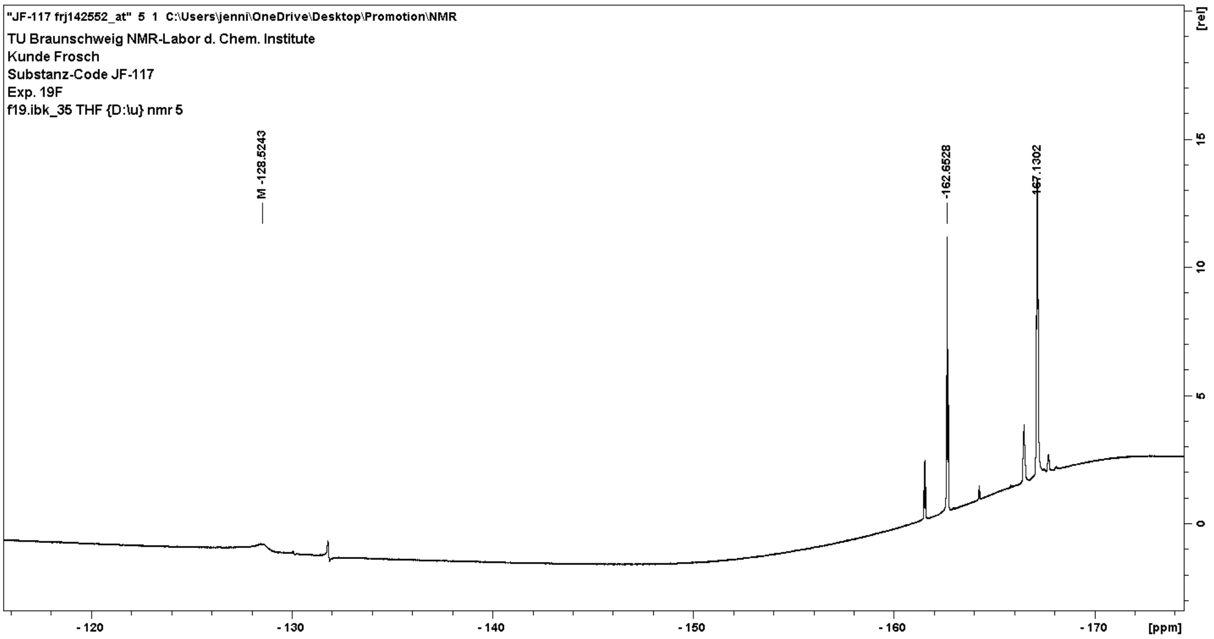

2.12. [PPh$_4$][(WCA-IDipp)] (6):

^{1}H NMR (600 MHz, THF-d_8) of **6**:

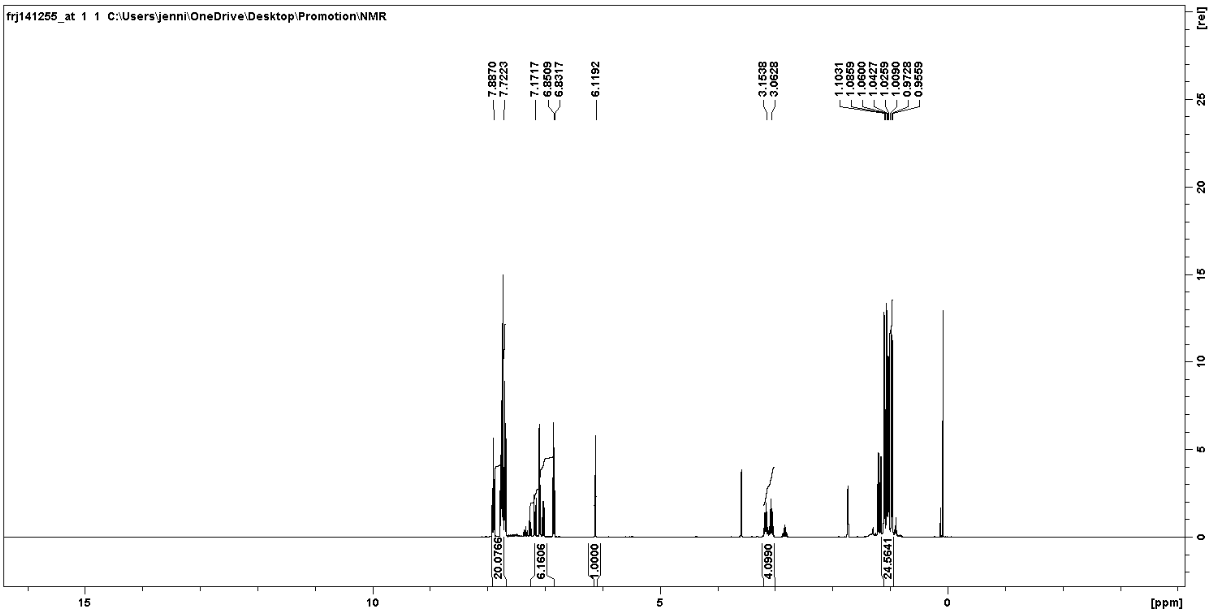

$^{13}C\{^1H\}$ **NMR** (100 MHz, THF-d_8) of **6**:

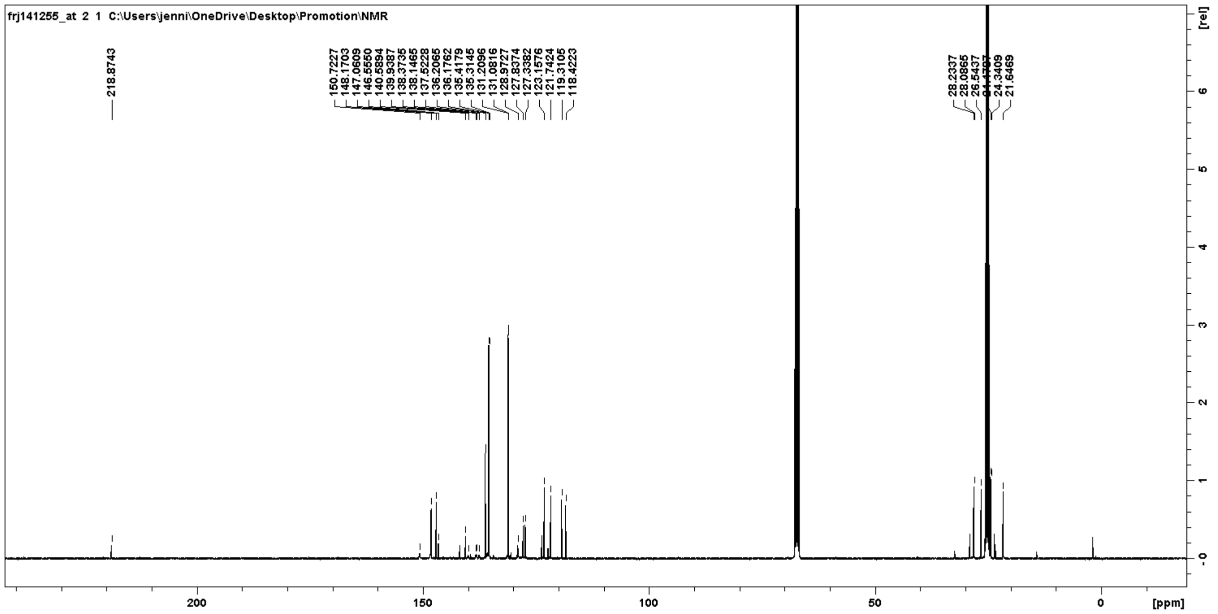

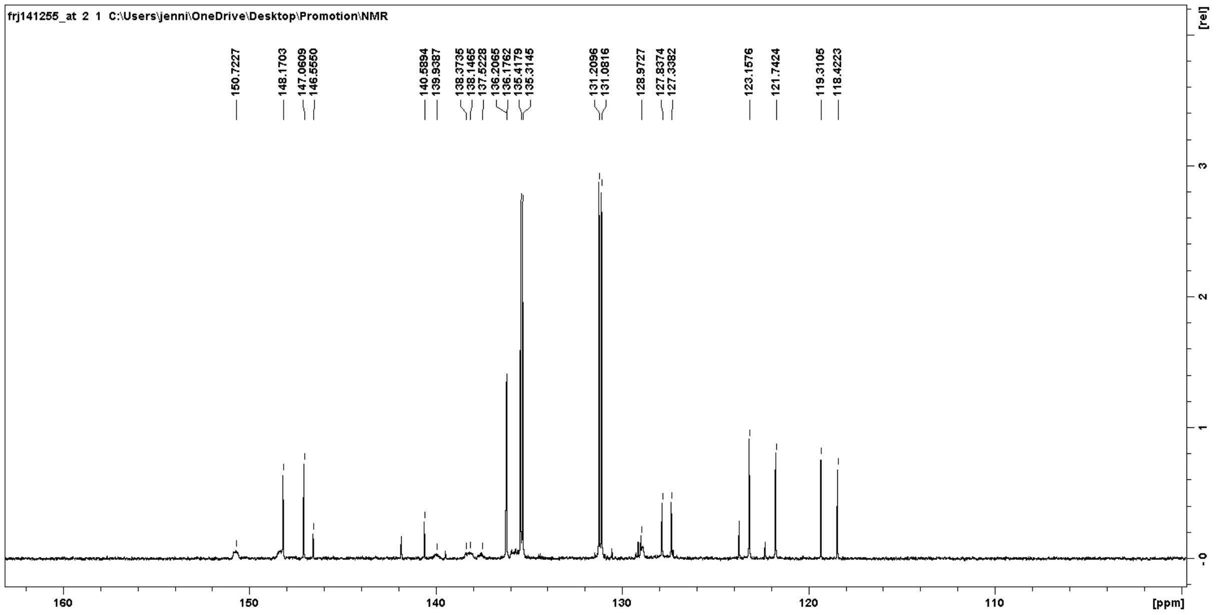

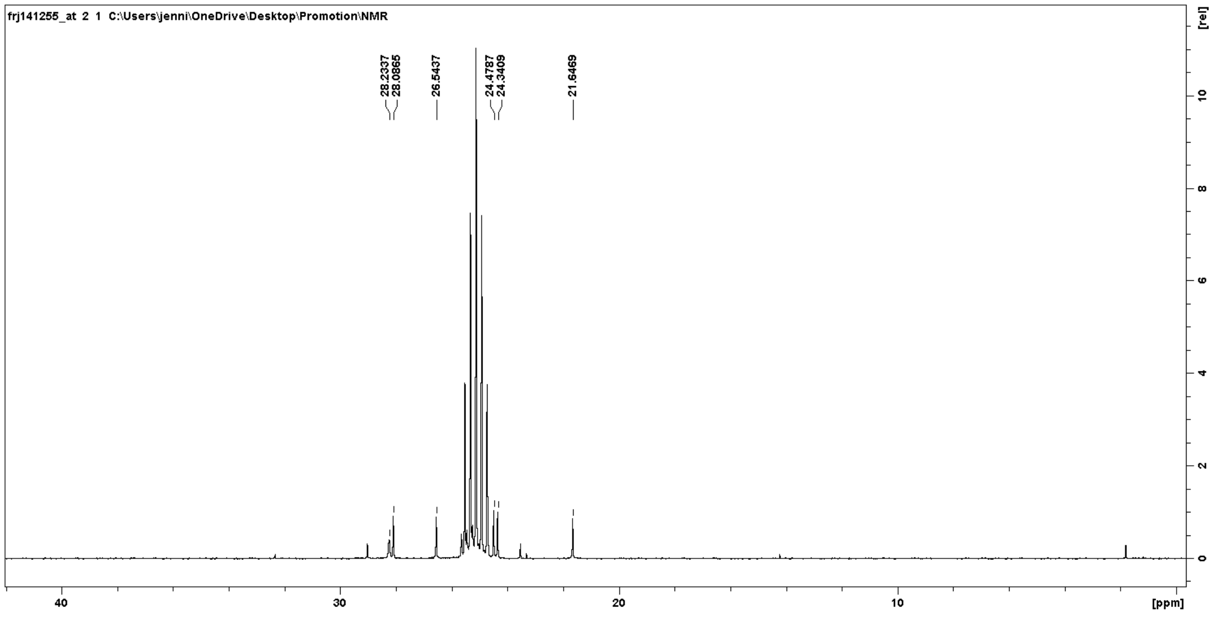

^{11}**B{**^{1}**H} NMR** (128 MHz, THF-d_8) of **6**:

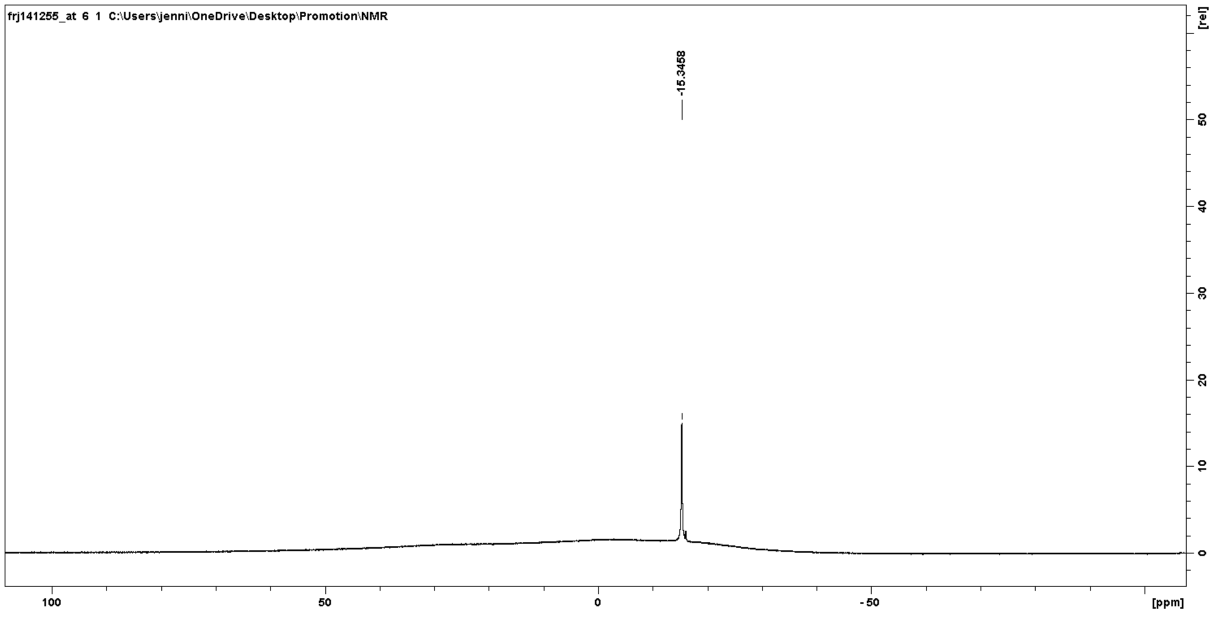

^{19}F{^{1}H} NMR (188 MHz, THF-d_8) of **6**:

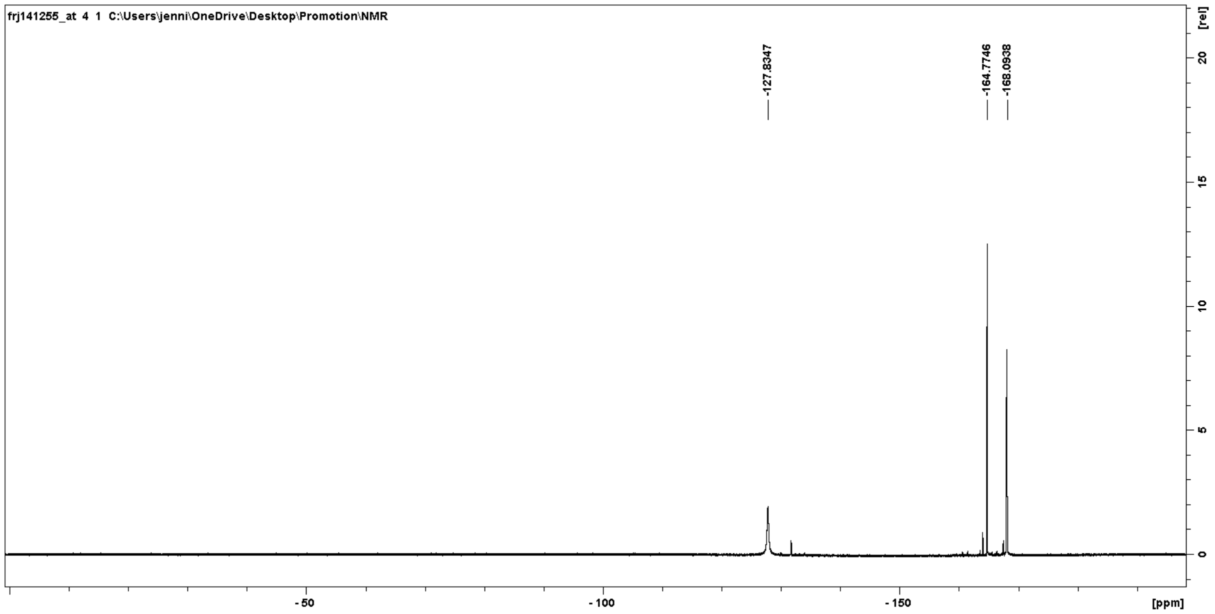

^{31}P{^{1}H} NMR (162 MHz, THF-d_8) of **6**:

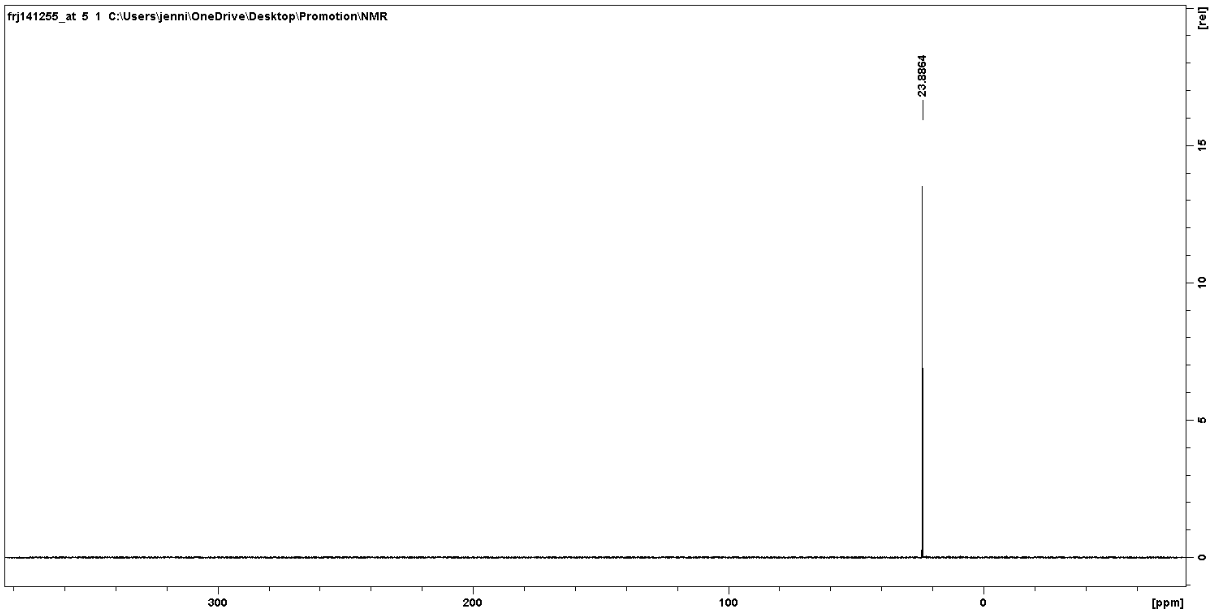

2.13. [PPh$_4$][(WCA-IDipp)$_2$I] (7a):

^{1}H NMR (600 MHz, THF-d_8) of **7a**:

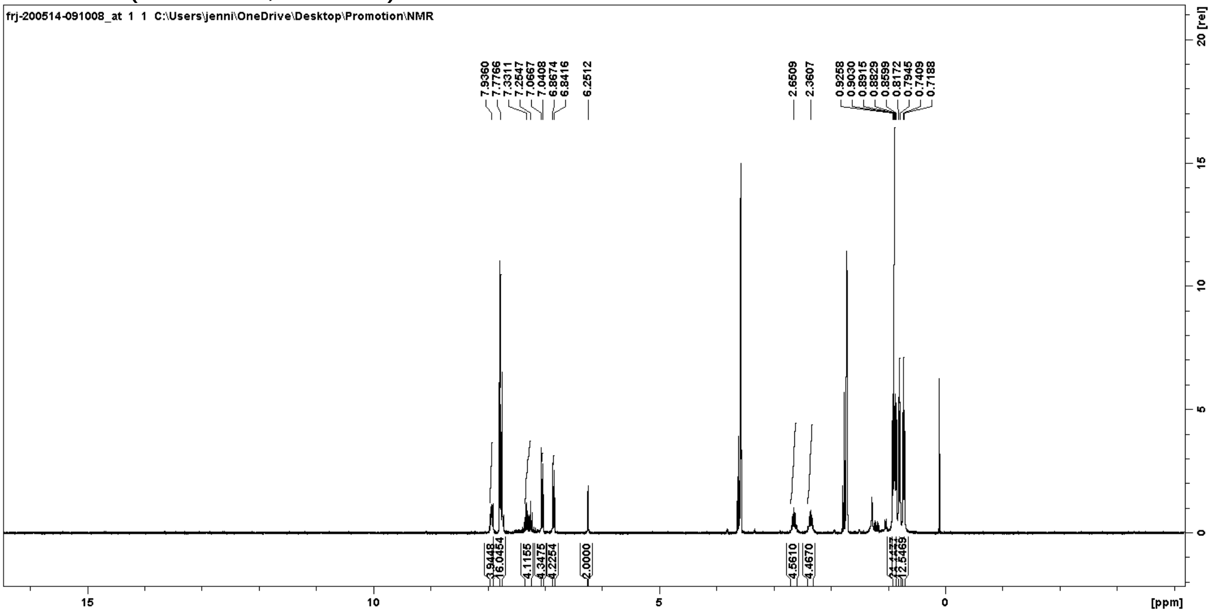

^{13}C{^{1}H} NMR (150 MHz, THF-d_8) of **7a**:

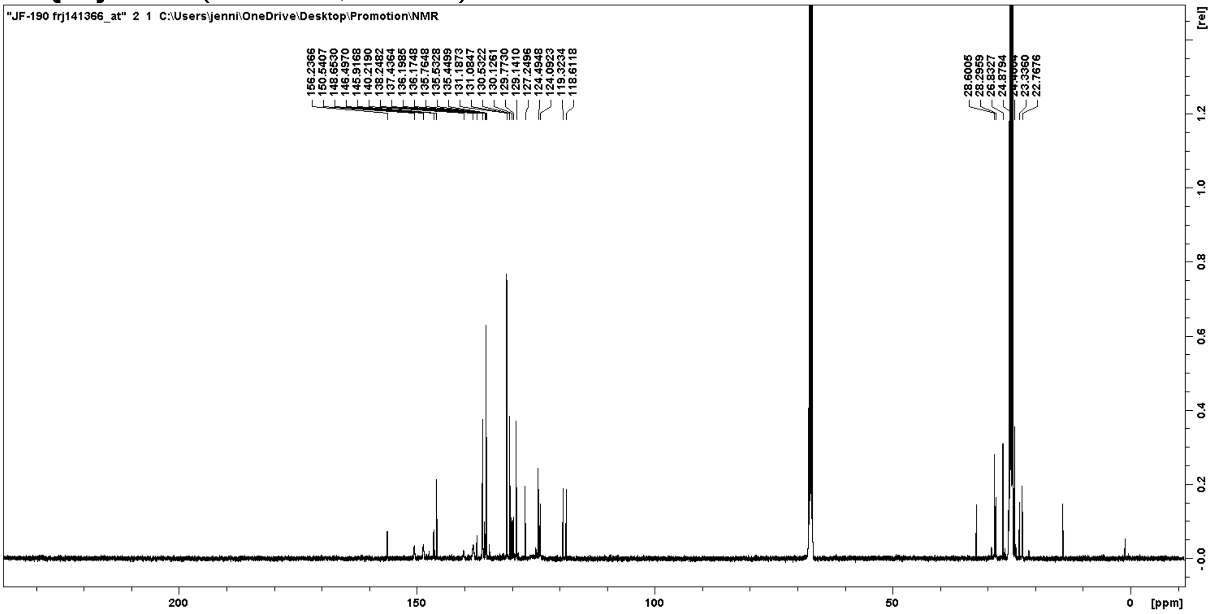

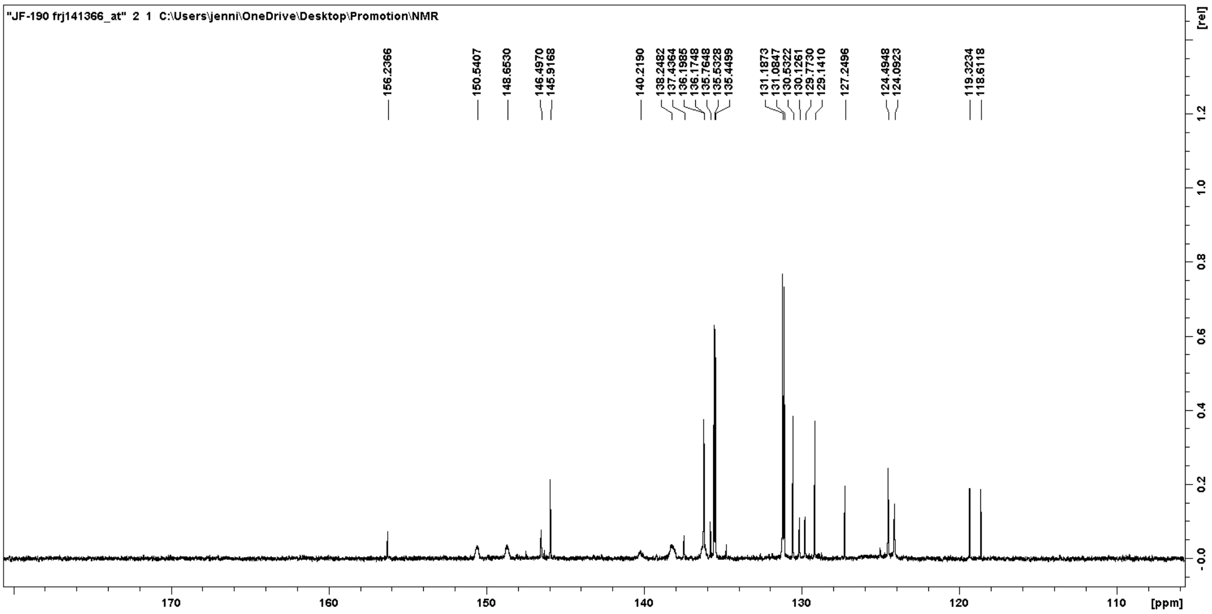
"JF-190 frj141366_at" 2 1 C:\Users\jenni\OneDrive\Desktop\Promotion\NMR
156.2366
150.5407
148.6530
146.4970
145.9168
140.2190
138.2482
137.4364
136.1985
136.1748
135.7648
135.5328
135.4499
131.1873
131.0847
130.5322
130.1261
129.7730
129.1410
127.2496
124.4948
124.0923
119.3234
118.6118
[rel]
170
160
150
140
130
120
110
[ppm]

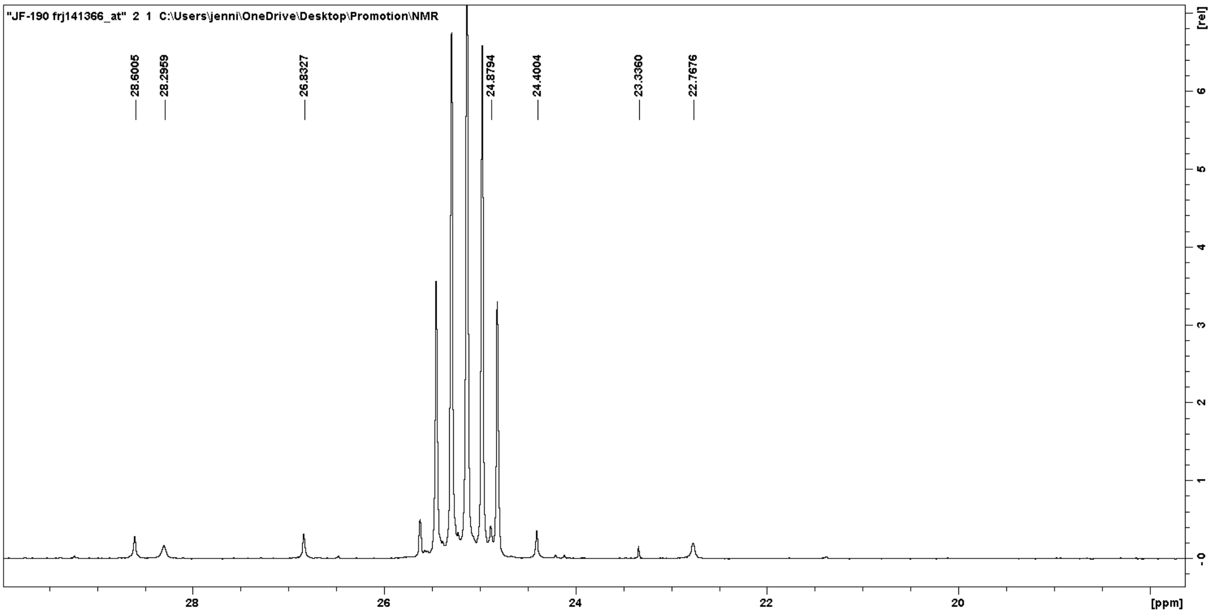
"JF-190 frj141366_at" 2 1 C:\Users\jenni\OneDrive\Desktop\Promotion\NMR
28.6005
28.2959
26.8327
24.8794
24.4004
23.3360
22.7676
[rel]
28
26
24
22
20
[ppm]

$^{11}B\{^1H\}$ NMR (128 MHz, THF-*d*8) of **7a**:

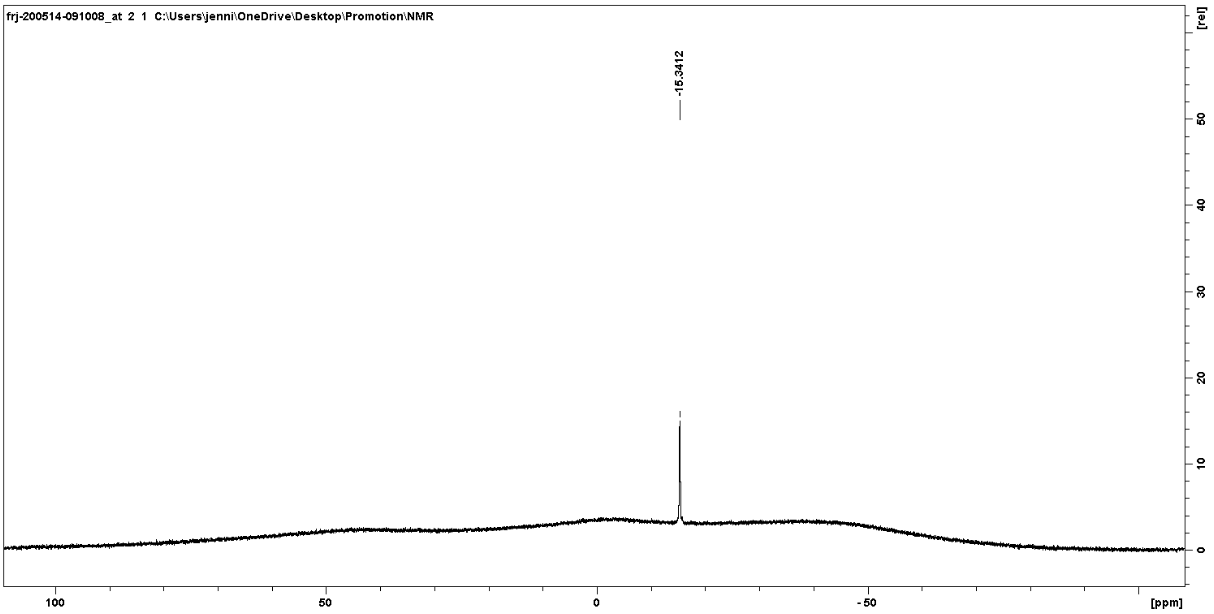

$^{19}F\{^1H\}$ NMR (188 MHz, THF-*d*8) of **7a**, where the baseline correction could not be done without the loss of the signal at −128.7 ppm.

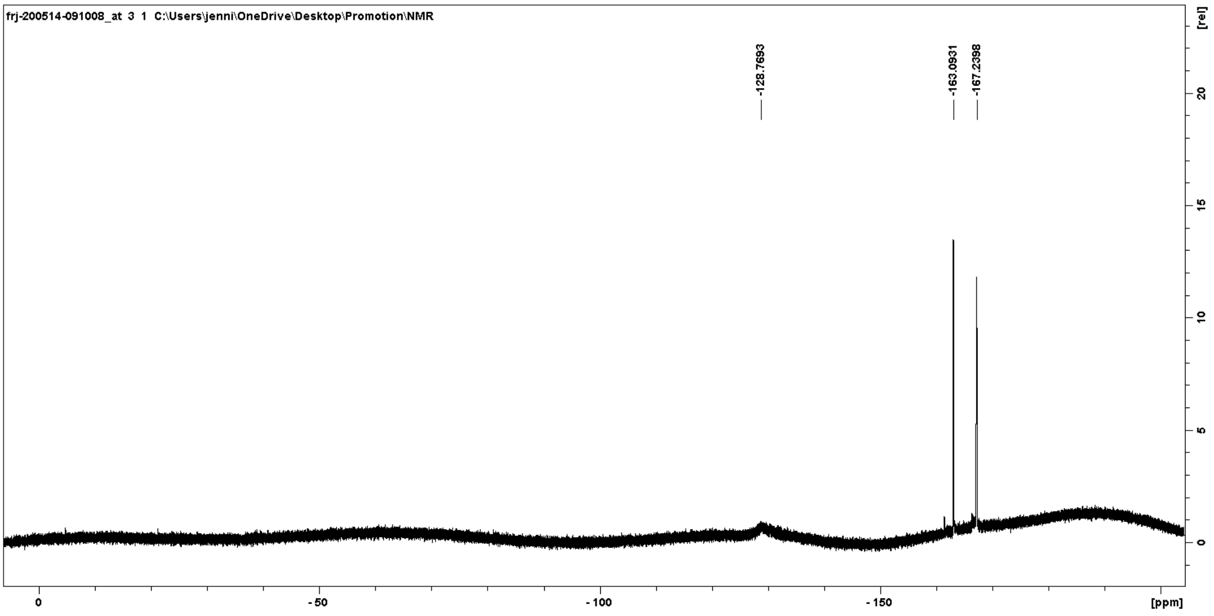

$^{31}P\{^{1}H\}$ **NMR** (162 MHz, THF-d_8) of **7a**:

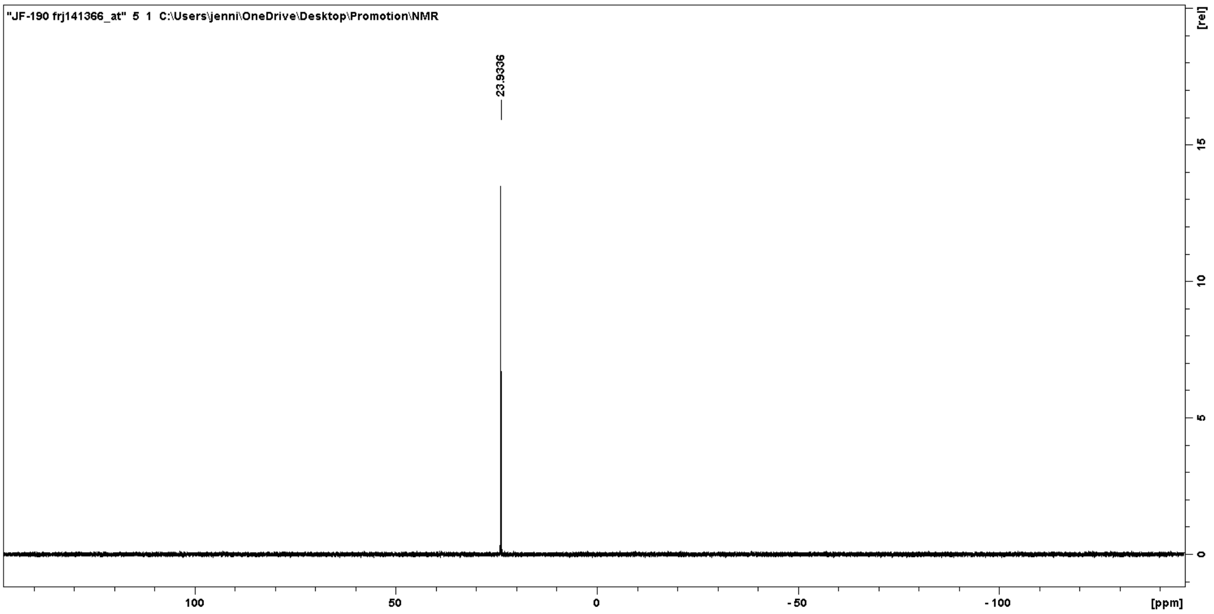

2.14. [PPh$_4$][(WCA-IDipp)I(WCA-IMes)] (7b):

^{1}H **NMR** (600 MHz, THF-d_8) of **7b**:

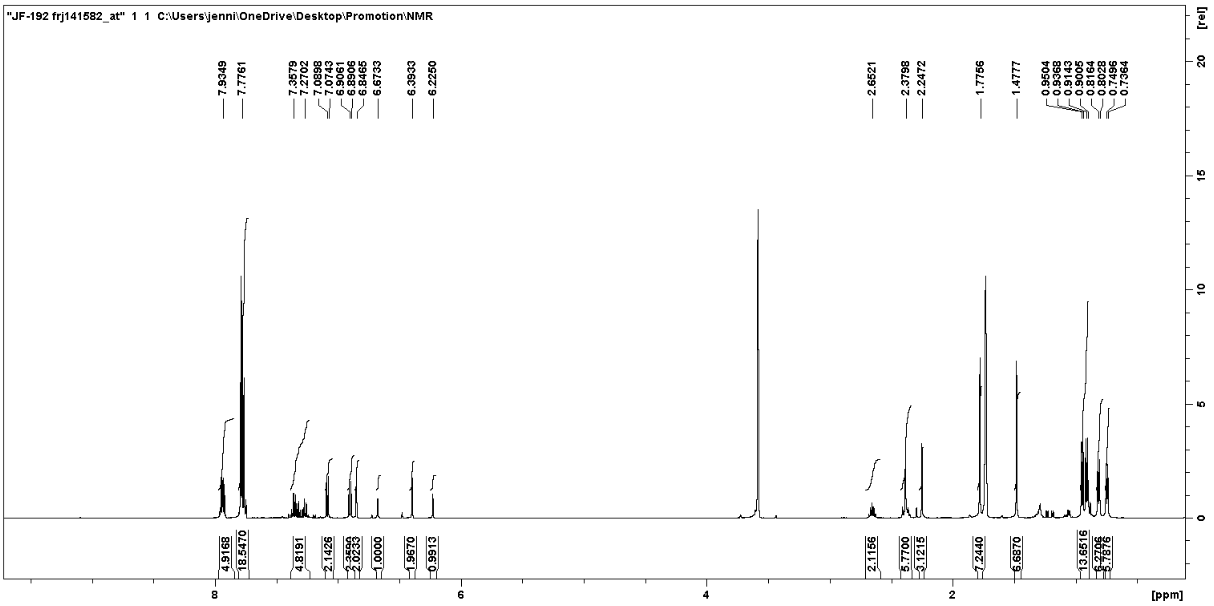

$^{13}C\{^1H\}$ NMR (100 MHz, THF-*d*8) of **7b**: A longer measurement revealed a better resolution of the signals in the aromatic region, but decomposition of the compound **7b** already lead to signals of the protonated carbene **9a**.

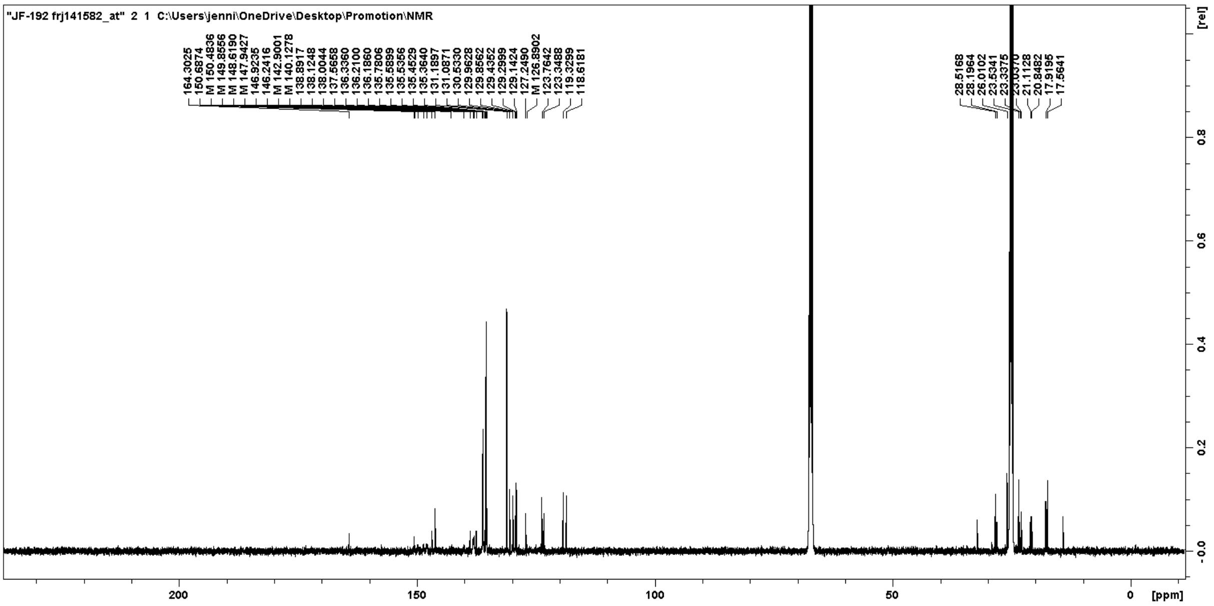

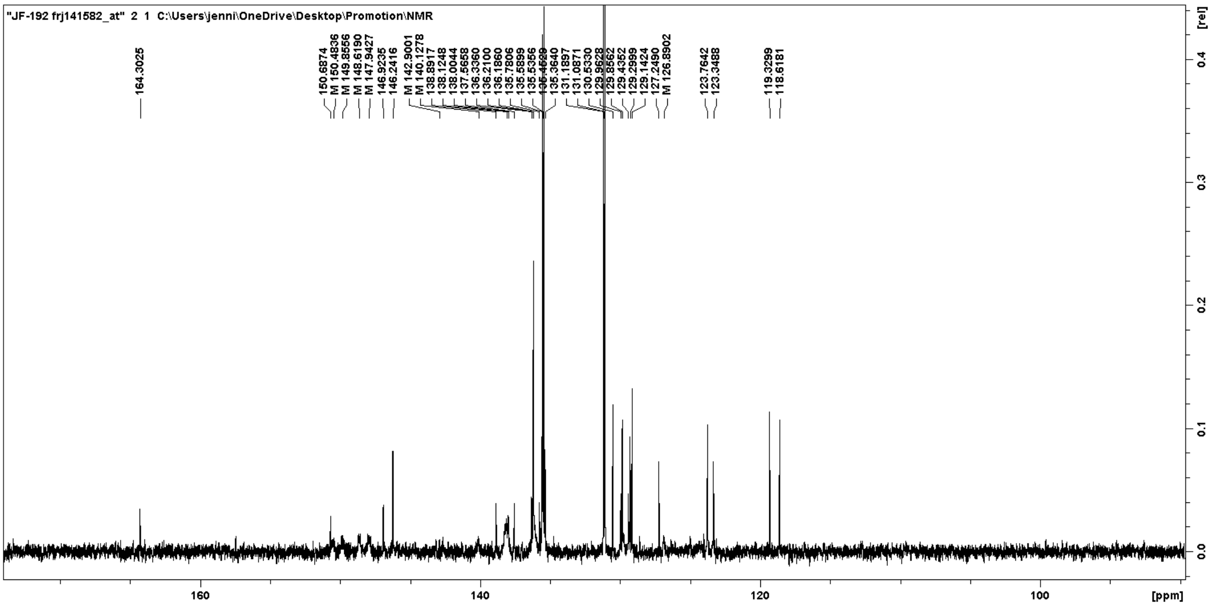

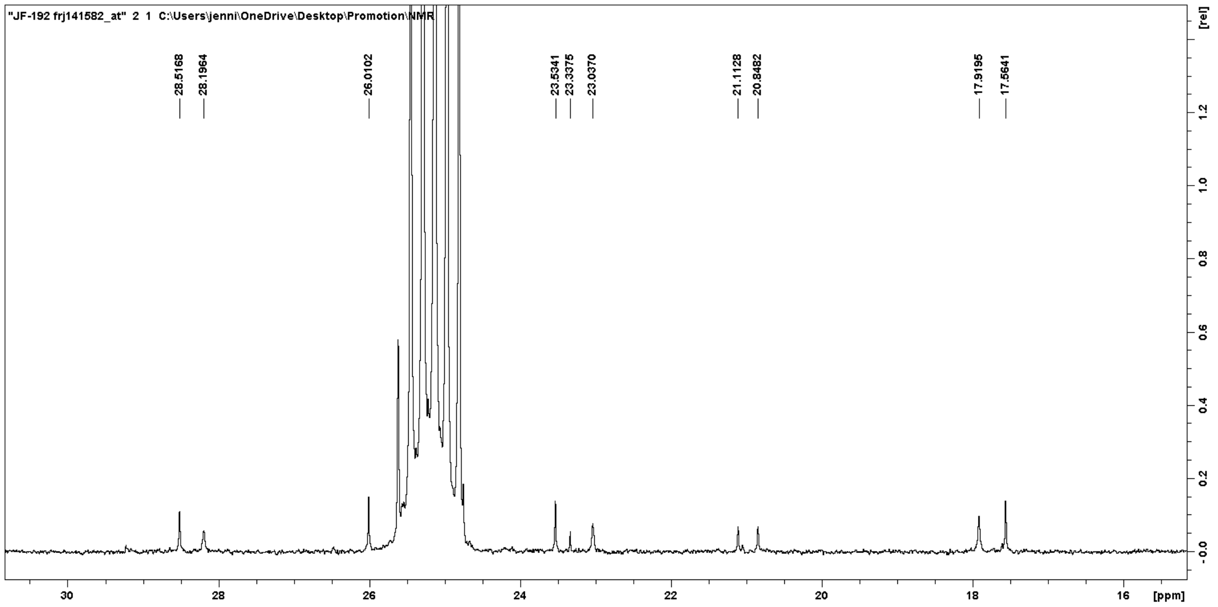

$^{11}B\{^1H\}$ NMR (128 MHz, THF-d_8) of **7b**:

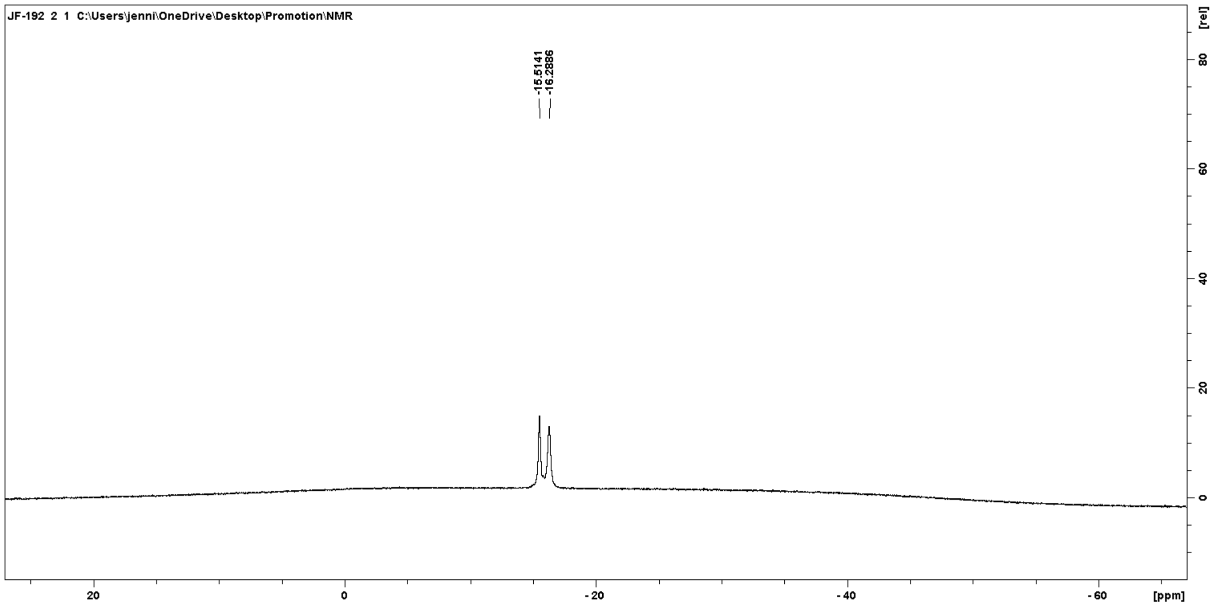

^{19}F{^{1}H} NMR (188 MHz, THF-*d*8) of **7b**:

Decomposition of the compound **7b** already lead to signals of the protonated carbene **9a**.

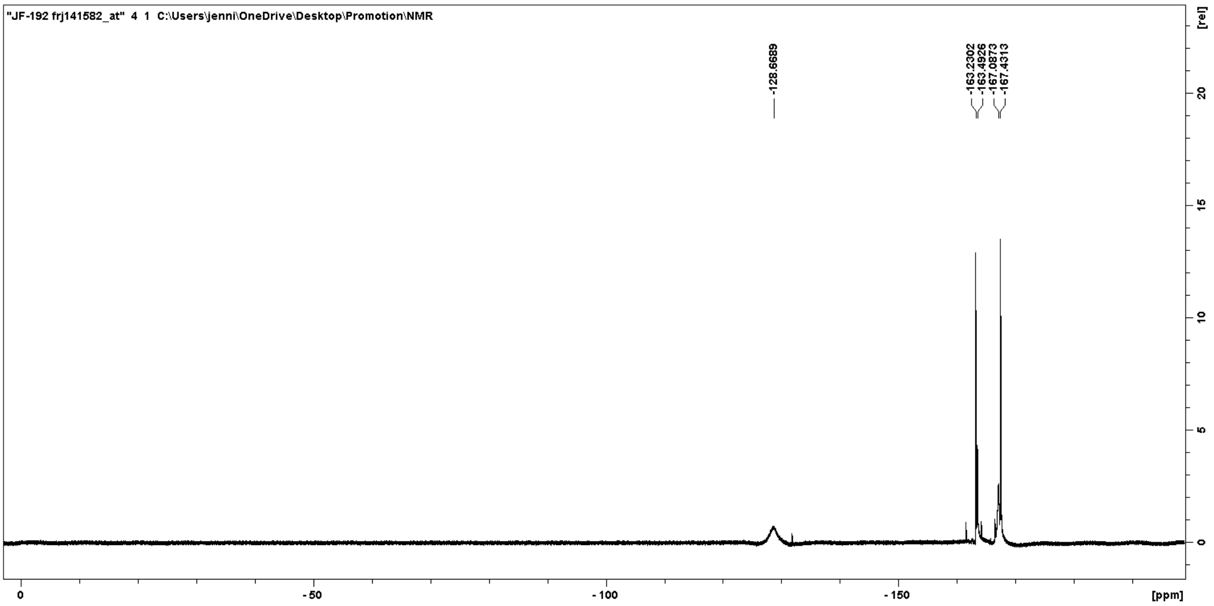

^{31}P{^{1}H} NMR (162 MHz, THF-*d*8) of **7b**:

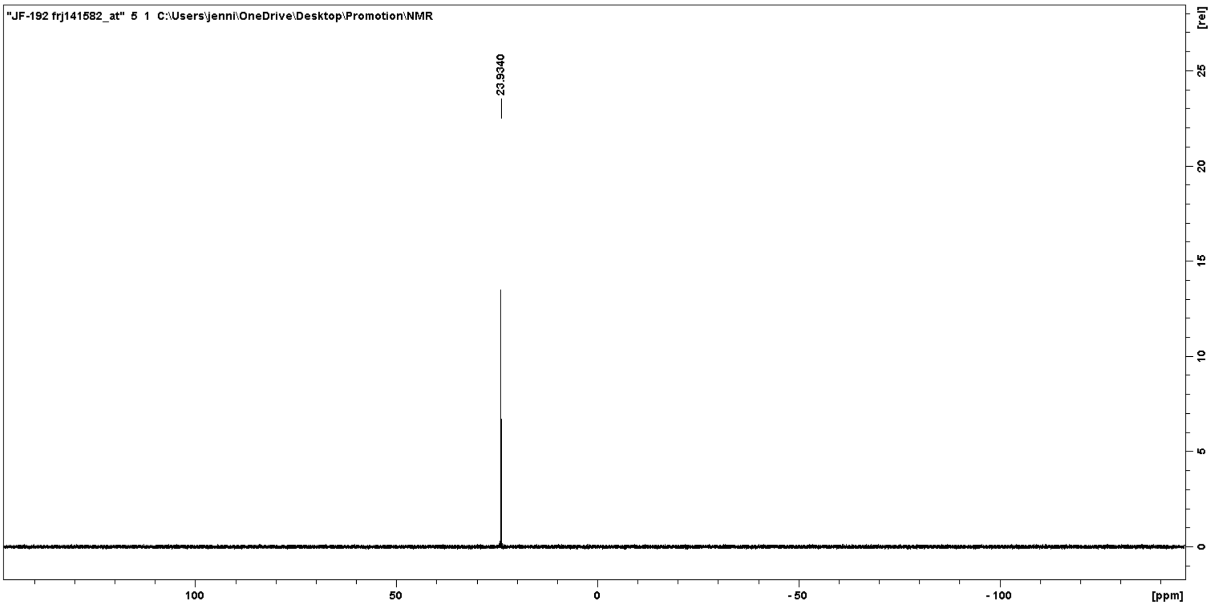

2.15. [PPh_4][(WCA-IDipp)$_2$Br] (8):

In the presented NMR spectra already, small signals arise beside the desired signals due to quick decomposition of **8** in solution, which lead to the formation of the protonated carbene **9a** and bromoimidazoliumsalts.

^{1}**H NMR** (500 MHz, THF-d_8) of **8**:

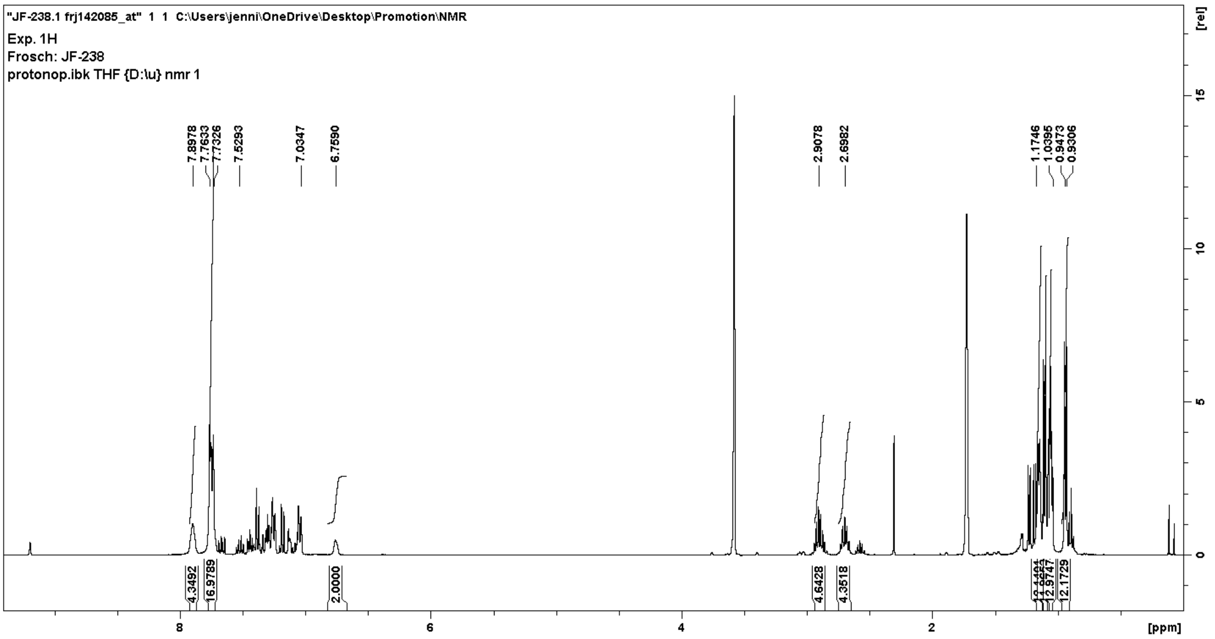

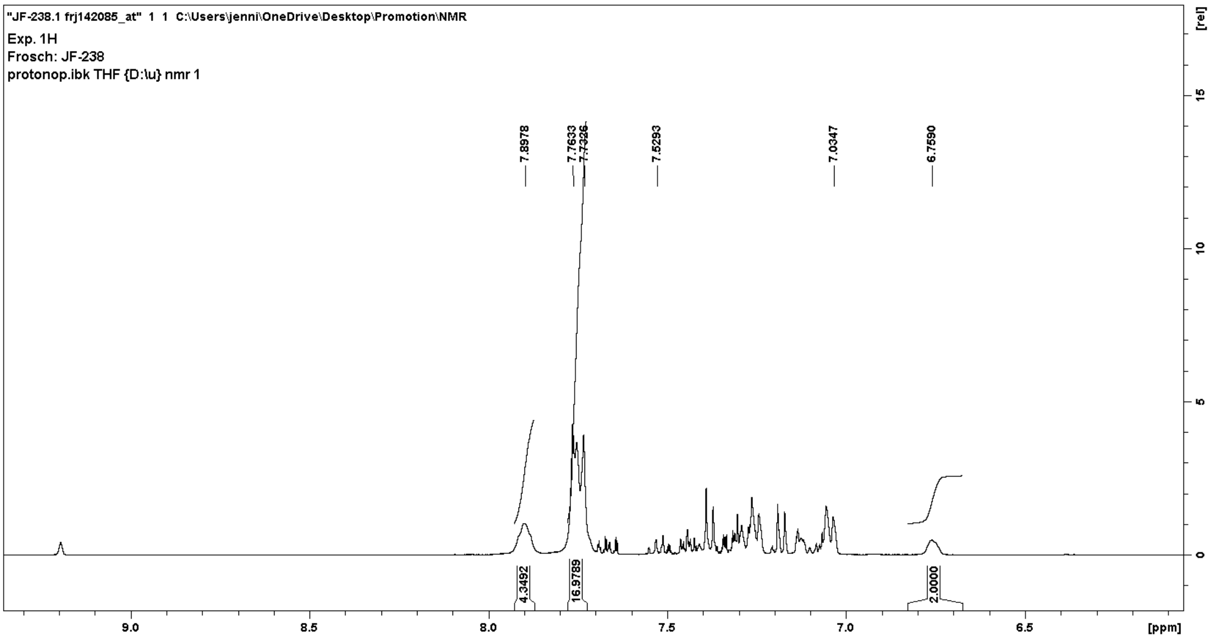

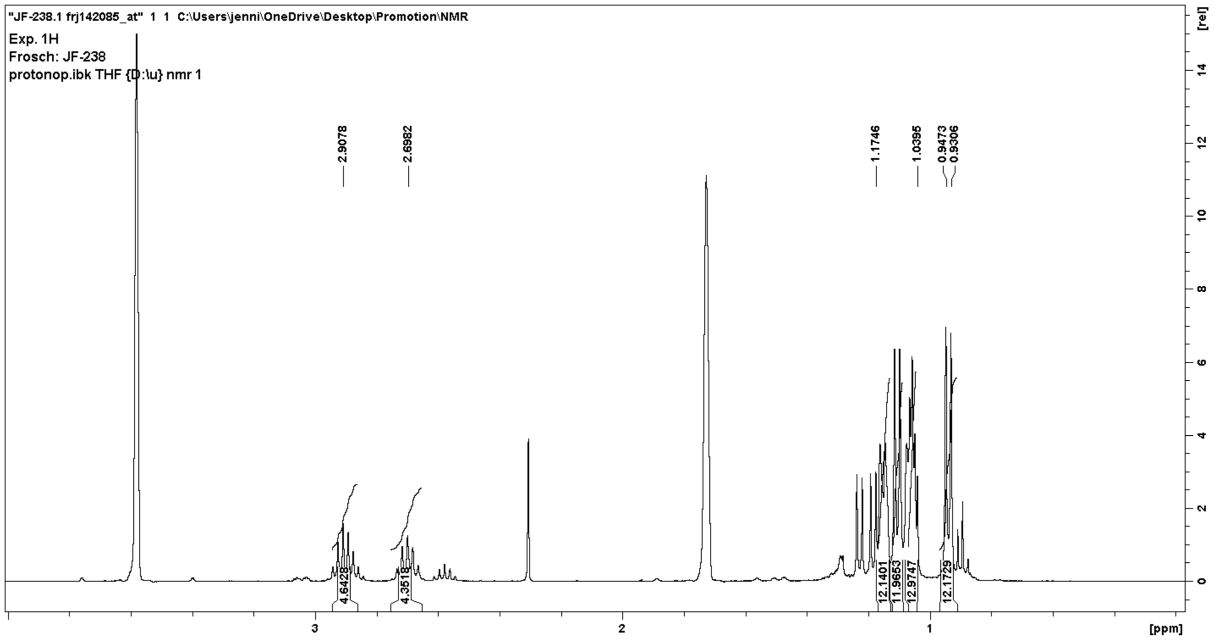

$^{13}C\{^{1}H\}$ NMR (125 MHz, THF-d_8) of **8**:

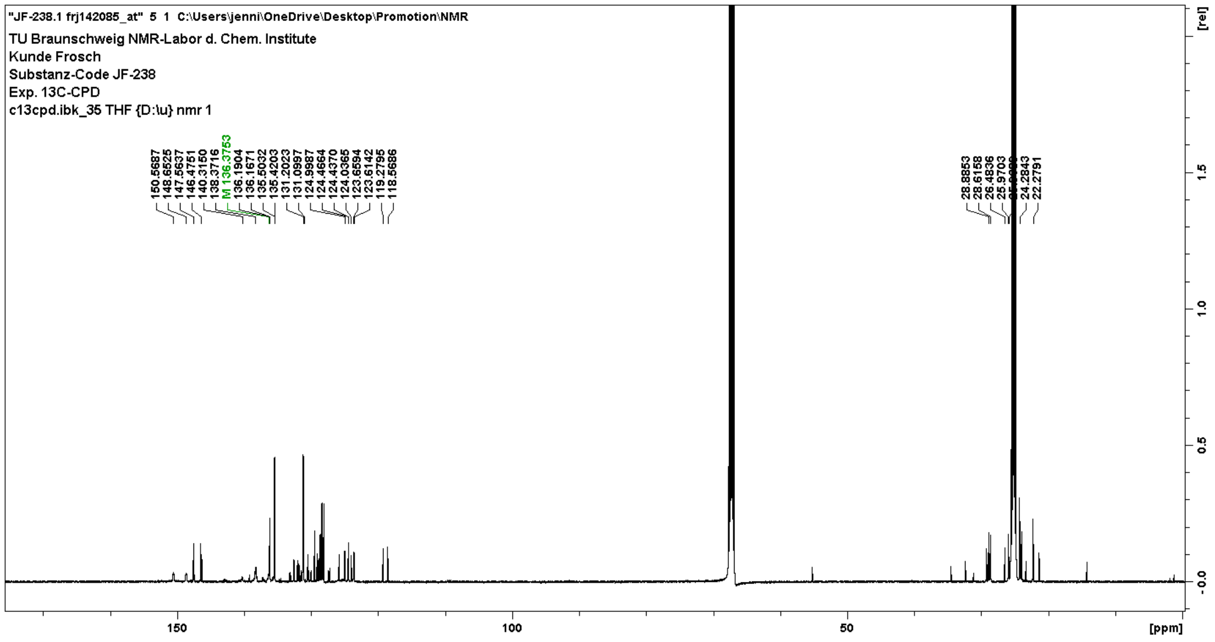

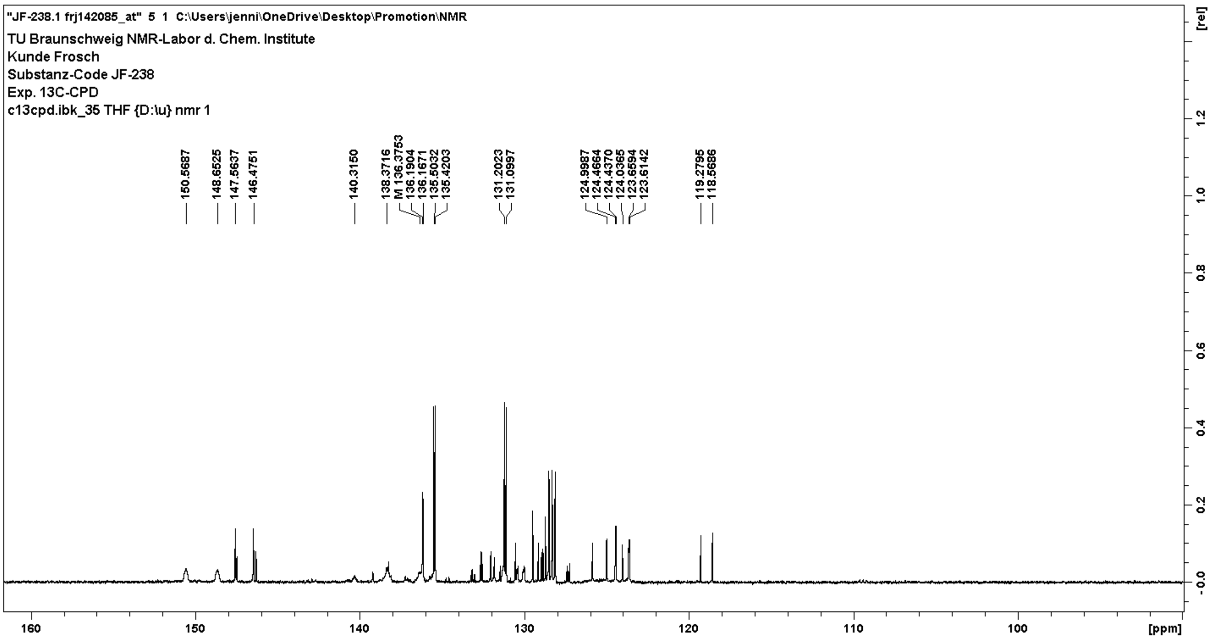
"JF-238.1 frj142085_at" 5 1 C:\Users\jenni\OneDrive\Desktop\Promotion\NMR
TU Braunschweig NMR-Labor d. Chem. Institute
Kunde Frosch
Substanz-Code JF-238
Exp. 13C-CPD
c13cpd.ibk_35 THF {D:\u} nmr 1
150.5687
148.6525
147.5637
146.4751
140.3150
138.3716
M 136.3753
136.1904
136.1671
135.5032
135.4203
131.2023
131.0997
124.9987
124.4664
124.4370
124.0365
123.6594
123.6142
119.2795
118.5686
[ppm]
[rel]

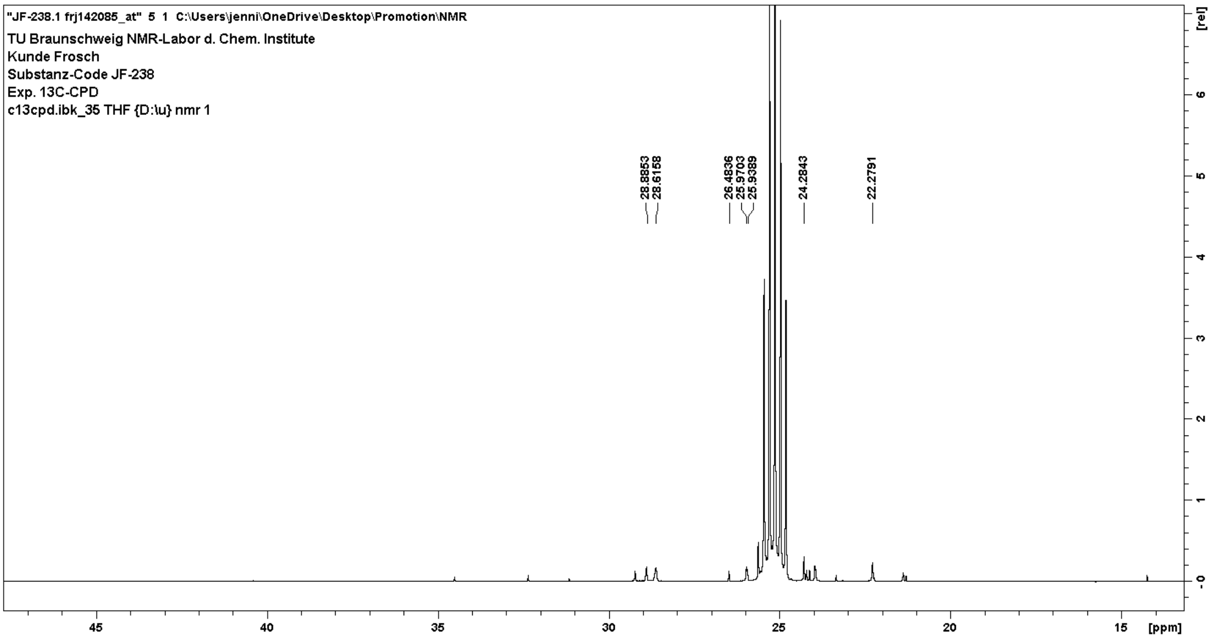
"JF-238.1 frj142085_at" 5 1 C:\Users\jenni\OneDrive\Desktop\Promotion\NMR
TU Braunschweig NMR-Labor d. Chem. Institute
Kunde Frosch
Substanz-Code JF-238
Exp. 13C-CPD
c13cpd.ibk_35 THF {D:\u} nmr 1
28.8853
28.6158
26.4836
25.9703
25.9389
24.2843
22.2791
[ppm]
[rel]

^{11}**B{^{1}H} NMR** (160 MHz, THF-*d*8) of **8**:

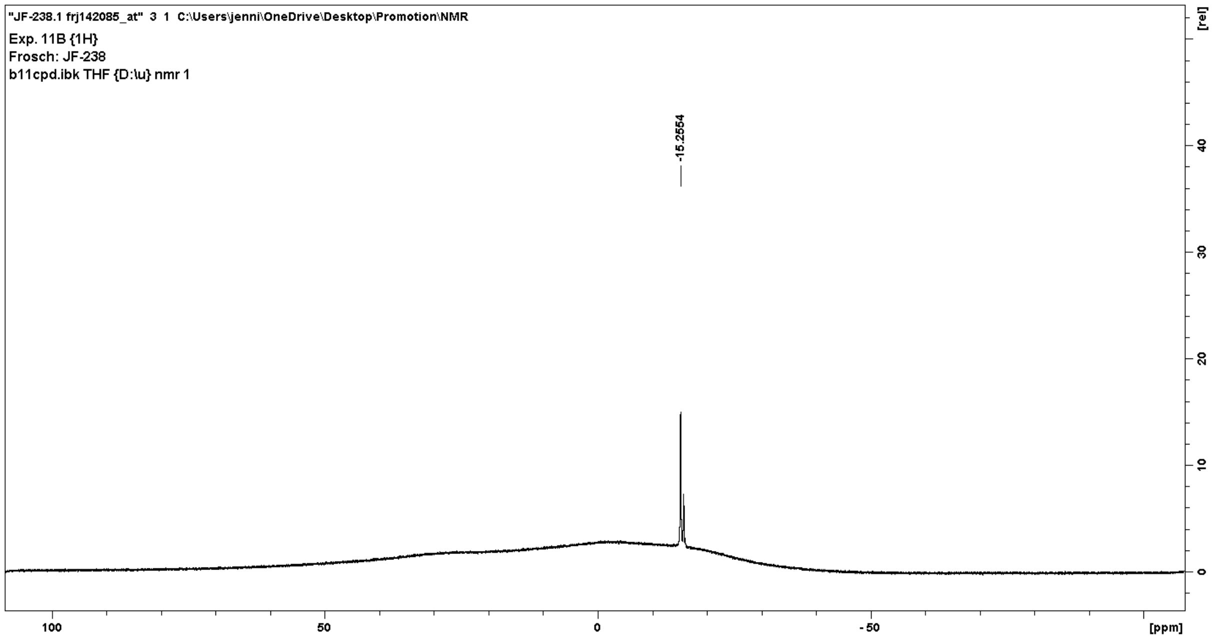

^{19}**F{^{1}H} NMR** (470 MHz, THF-*d*8) of **8**:

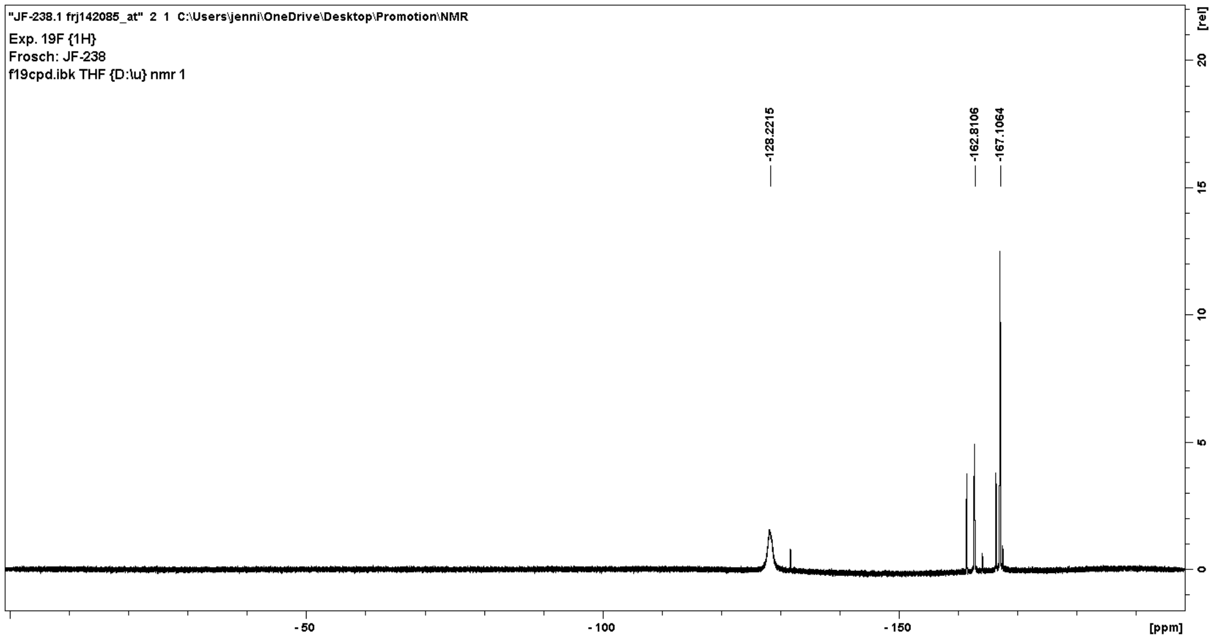

^{19}F{^{1}H} NMR (202 MHz, THF-*d*$_8$) of **8**:

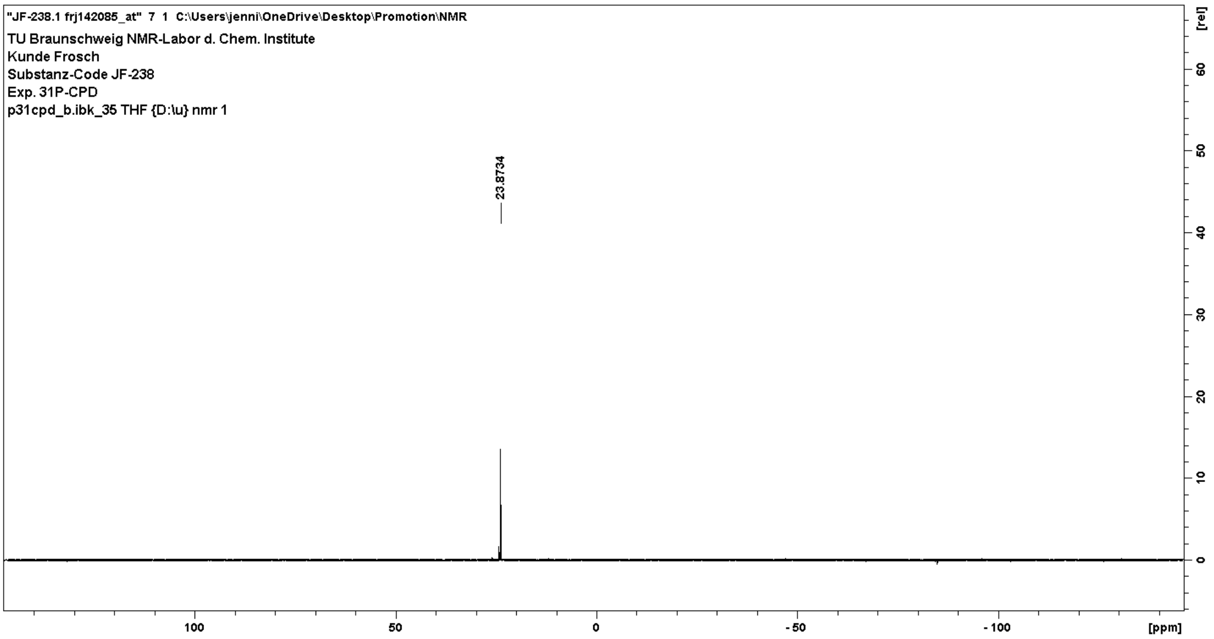

2.16. [(WCA-IDipp)H] (9a):

NMR data of **9a** in C_6D_6:[5]

^{1}H-NMR (300 MHz, C_6D_6): [5] *δ* [ppm] = 7.10 (t, $^{3}J_{H,H}$ = 7.79 Hz, 1 H, *p*-Dipp), 7.06, 6.89 (d, $^{4}J_{H,H}$ = 1.70 Hz, 1 H, CH), 6.85 (d, $^{3}J_{H,H}$ = 7.79 Hz, 4 H, *m*-Dipp), 6.80, 6.52 (br d, $^{4}J_{H,H}$ = 1.70 Hz, CH), 2.78 (sept, $^{3}J_{H,H}$ = 7.71 Hz, 4 H, C<u>H</u>(CH_3)$_2$), 2.31, 1.01 (d, $^{3}J_{H,H}$ = 6.71 Hz, 24 H, CH(C<u>H</u>$_3$)$_2$), 0.97, 0.94, 0.73.

^{13}C{^{1}H}-NMR (100 MHz, C_6D_6):[5] *δ* [ppm] = 21.2, 23.6, 24.5 26.5 (s, CH(<u>C</u>H$_3$)$_2$), 28.0 (br s, <u>C</u>H(CH_3)$_2$), 28.5 (s, <u>C</u>H(CH_3)$_2$), 123.6, 124.4 (s, *m*-Dipp), 130.2 (br s, <u>C</u>HIm), 131.6, 131.7 (s, *p*-Dipp), 135.5 (s, CHIm), 145.5, 146.5 (s, *o*-Dipp). The resonances of the carbon atoms in the C_6F_5-ring and the C_{ipso}-Dipp positions could not be detected and assigned properly.

^{11}B{^{1}H}-NMR (128 MHz, C_6D_6):[5] *δ* [ppm] = −15.8 (s).

^{19}F{^{1}H}-NMR (376 MHz, C_6D_6):[5] *δ* [ppm] = −129.2 (br s, 6 F, *o*-F), −159.3 (m, 3 F, *p*-F), −165.2 (s, 6 F, *m*-F).

In this paper mainly THF-*d*$_8$ was used as NMR solvent. **9a** was obtained multiple times as a side or decomposition product, but was not further characterized. In the following raw NMR data of **9a** are presented.

^{1}H-NMR (300 MHz, THF-*d*$_8$): δ [ppm] = 9.07 (d, $^4J_{H,H}$ = 1.71 Hz, 1 H, N-CH-N), 7.53 (dd, $^3J_{H,H}$ = 8.08 Hz, $^3J_{H,H}$ = 8.23 Hz, 1 H, *p*-Dipp), 7.44 (t, $^3J_{H,H}$ = 7.74 Hz, 1 H, *p*-Dipp), 7.38 (d, $^3J_{H,H}$ = 7.79 Hz, 2 H, *m*-Dipp), 7.18 (d, $^3J_{H,H}$ = 7.79 Hz, 2 H, *m*-Dipp), 7.13 (br s, 1 H, CH=CB), 2.87 (sept, $^3J_{H,H}$ = 6.72 Hz, 2 H, $C\underline{H}(CH_3)_2$), 2.57 (sept, $^3J_{H,H}$ = 6.88 Hz, 2 H, $C\underline{H}(CH_3)_2$), 1.22 (d, $^3J_{H,H}$ = 6.88 Hz, 6 H, $CH(C\underline{H}_3)_2$), 1.17 (d, $^3J_{H,H}$ = 6.79 Hz, 6 H, $CH(C\underline{H}_3)_2$), 1.04 (d, $^3J_{H,H}$ = 7.61 Hz, 12 H, $CH(C\underline{H}_3)_2$).

^{11}B{^{1}H}-NMR (96 MHz, THF-*d*$_8$): δ [ppm] = −15.81 (s).

^{19}F{^{1}H}-NMR (282 MHz, THF-*d*$_8$): δ [ppm] = −129.2 (br s, 6 F, *o*-F), −159.3 (m, 3 F, *p*-F), −165.2 (s, 6 F, *m*-F).

^{1}H-NMR (300 MHz, THF-*d*$_8$) of **9a**:

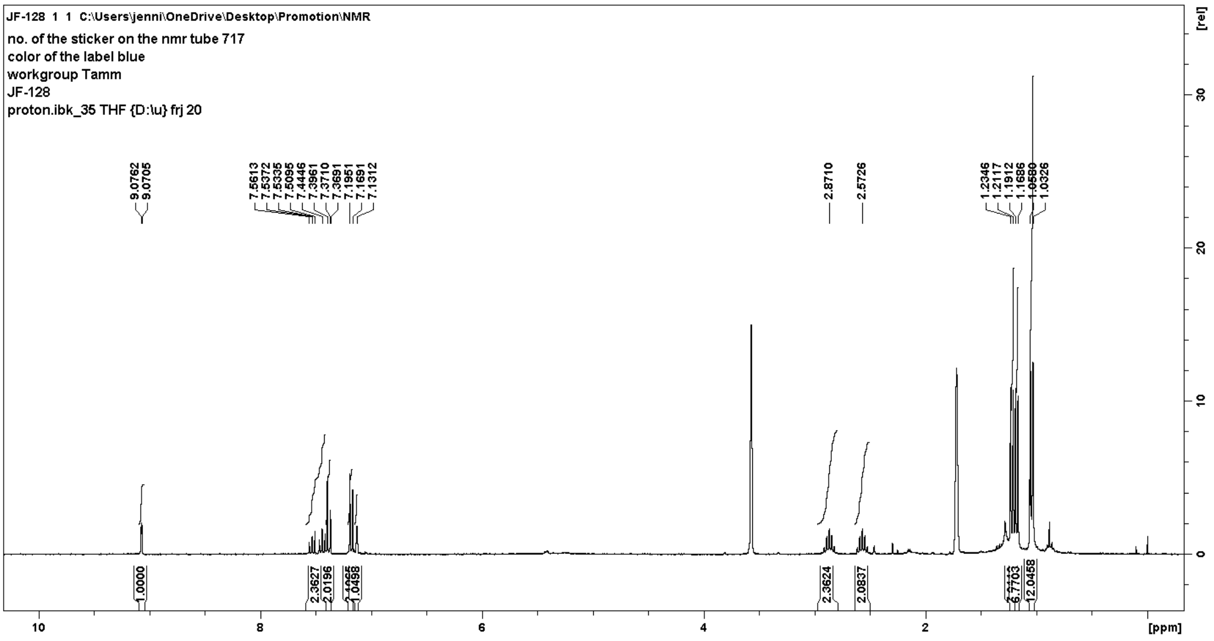

^{11}**B{^{1}H}-NMR** (96 MHz, THF-d_8) of **9a**:

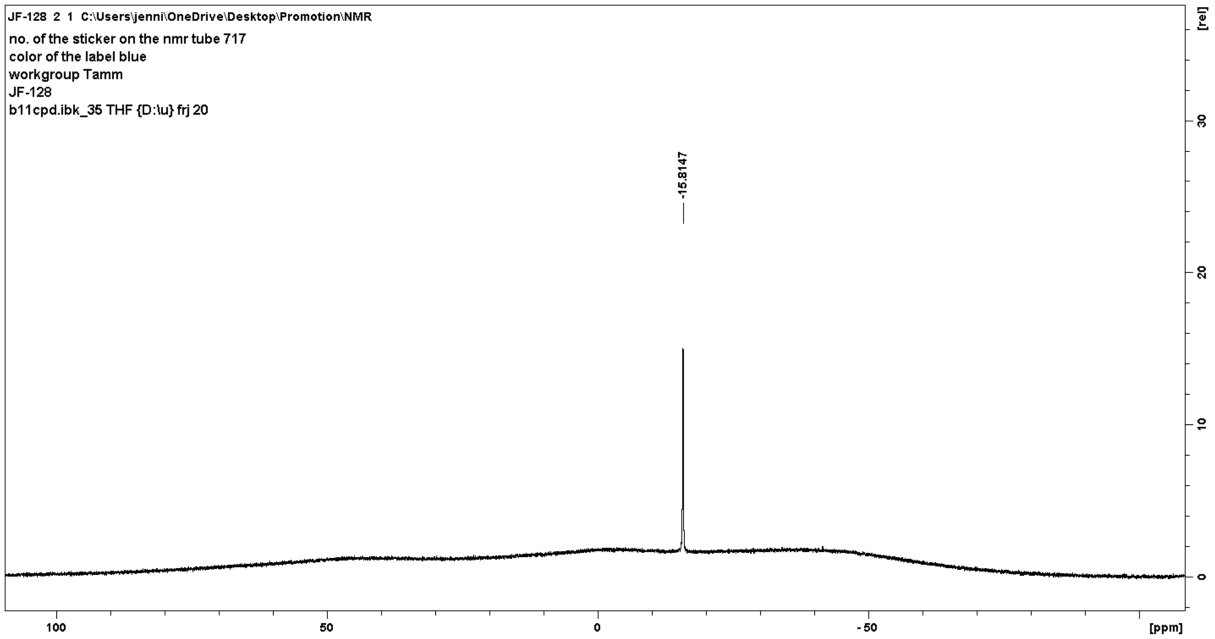

^{19}**F{^{1}H}-NMR** (282 MHz, THF-d_8) of **9a**:

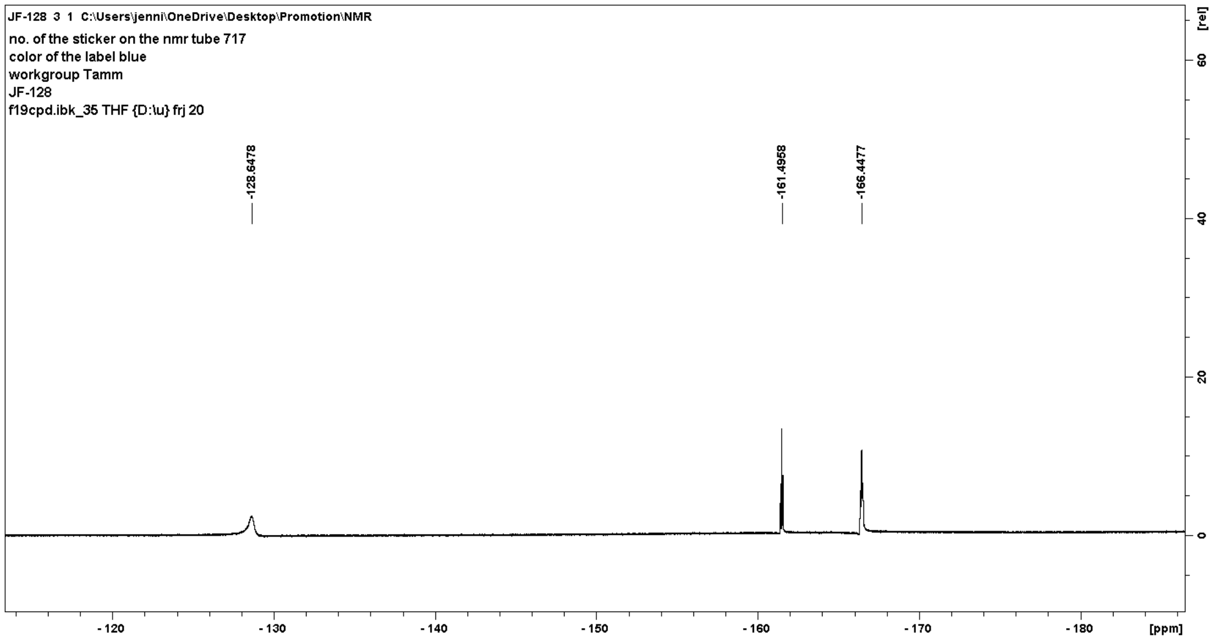

2.17. [(WCA-IMes)H] (9b):

9b was obtained multiple times as a side or decomposition product, but was not further characterized. In the following raw NMR data of **9b** in C_6D_6 are presented.

^{1}H-NMR (400 MHz, C_6D_6): δ [ppm] = 6.94 (s, 1 H, N-CH-N), 6.50 (d, $^4J_{H,H}$ = 0.6 Hz, 2 H, *m*-Mes), 6.41 (d, $^4J_{H,H}$ = 0.6 Hz, 2 H, *m*-Mes), 5.19 (d, $^3J_{H,H}$ = 1.7 Hz, 1 H, CH=CB N-CH-N), 2.08 (s, 3 H, *o*-CH_3-Mes), 2.01 (s, 3 H, *o*-CH_3-Mes), 1.71 (s, 6 H, *p*-CH_3-Mes), 1.67 (s, 6 H, *o*-CH_3-Mes).

^{11}B{^{1}H}-NMR (128 MHz, C_6D_6): δ [ppm] = −16.5 (s).

^{13}C{^{1}H}-NMR (100 MHz, C_6D_6): δ [ppm] = 150.2 (m, <u>C</u>B=CH), 147.6 (m, aryl-C_6F_5), 141.0 (s, *o*-Mes), 140.8 (m, aryl-C_6F_5), 140.3 (s, *o*-Mes), 138.4 (m, aryl-C_6F_5), 137.2 (m, aryl-C_6F_5), 135.9 (s, *p*-Mes), 134.8 (s, *p*-Mes), 134.4 (s, <u>C</u>H=C-B), 132.6 (s, *ipso*-Mes), 131.3 (s, *ipso*-Mes), 129.6 (s, *m*-Mes), 129.1 (s, *m*-Mes), 20.8 (s, *p*-CH_3-Mes), 20.5 (s, *p*-CH_3-Mes), 17.3 (s, *o*-CH_3-Mes), 16.9 (s,3 × *o*-CH_3-Mes).

^{19}F{^{1}H}-NMR (376 MHz, C_6D_6): δ [ppm] = −128.8 (s, 6 F, *o*-F), −159.9 (t, $^3J_{F,F}$ = 21.4 Hz, 3 F, *p*-F), −165.2 (s, 6 F, *m*-F).

^{1}H-NMR (400 MHz, C_6D_6) of **9b**:

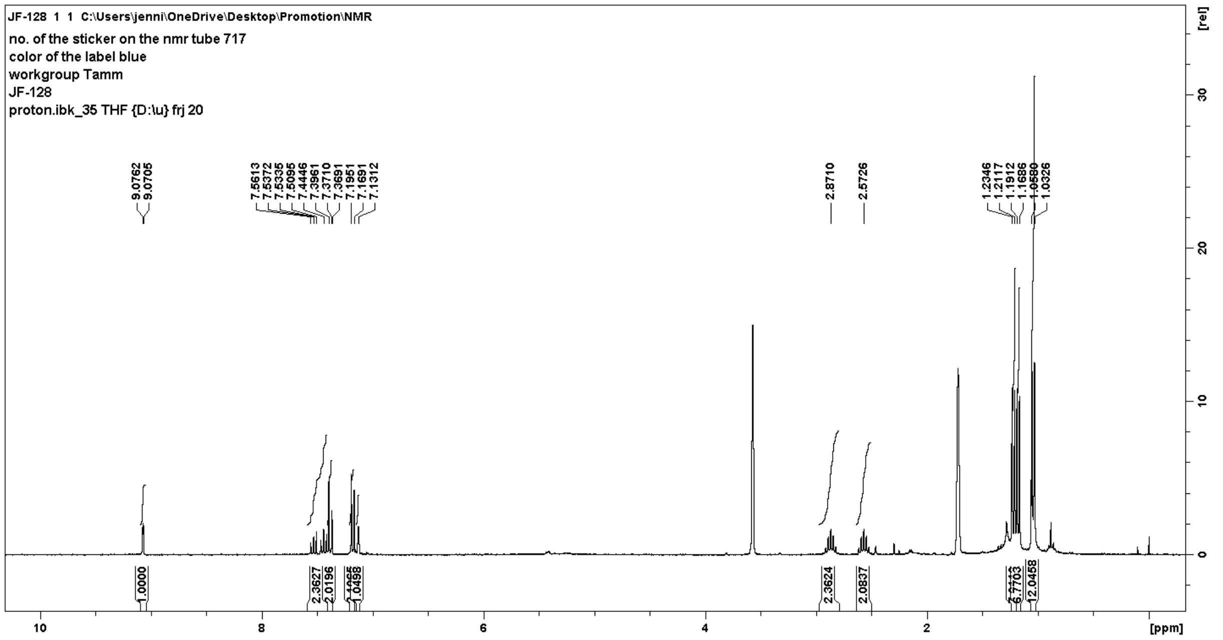

^{13}**C{^{1}H}-NMR** (100 MHz, C_6D_6) **9b**:

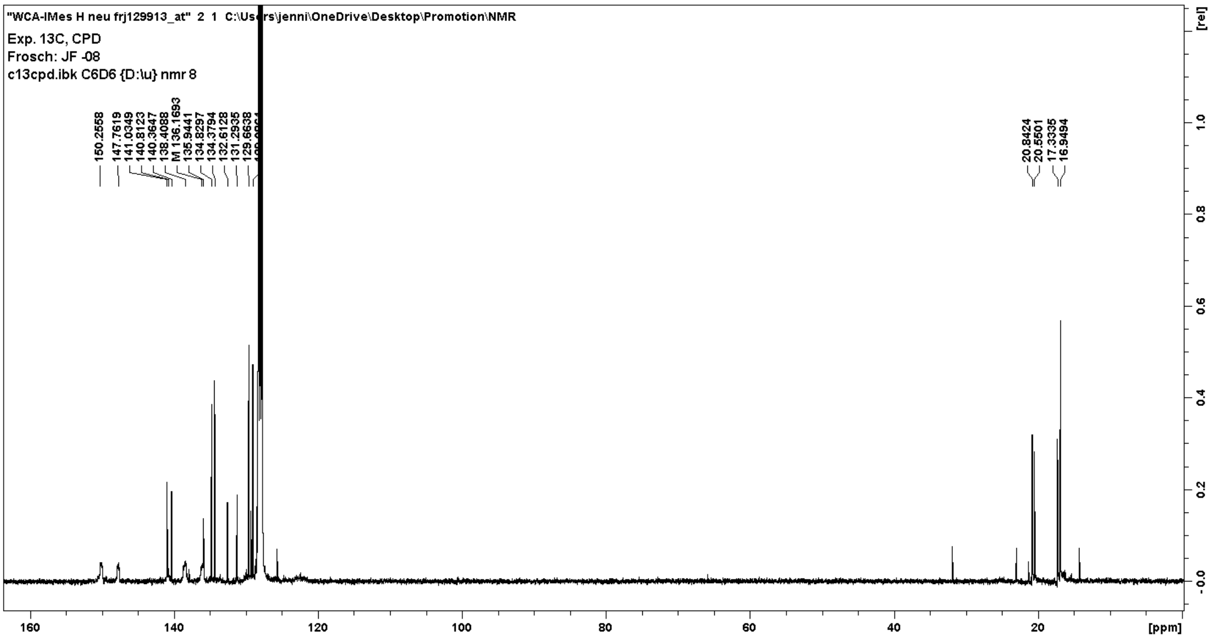

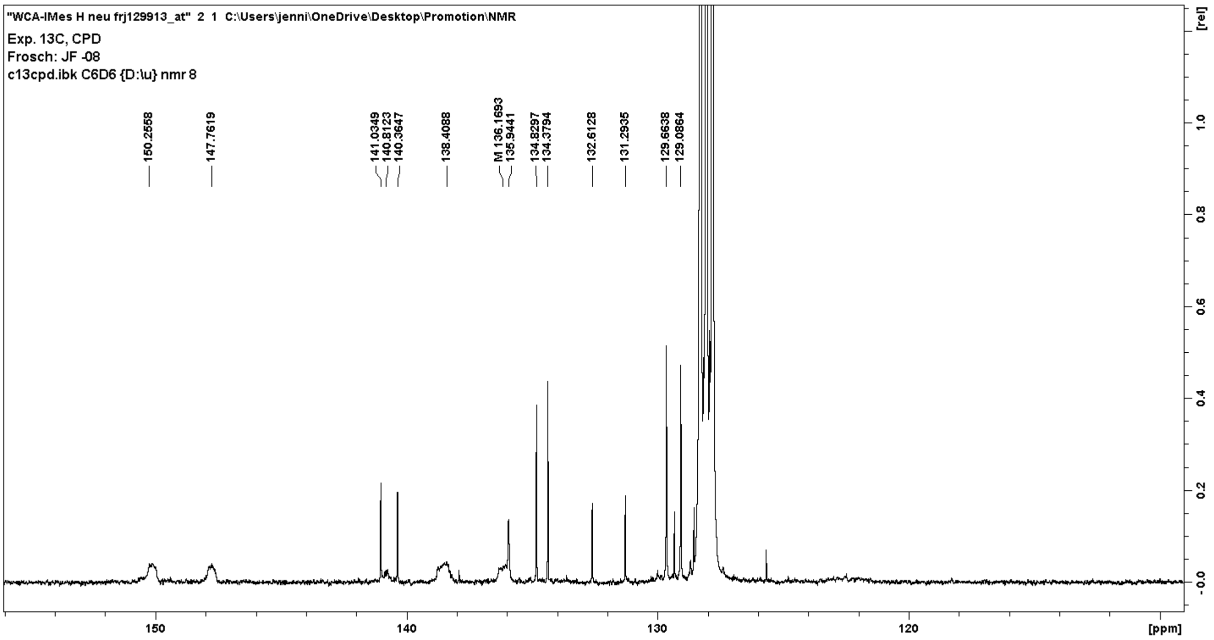

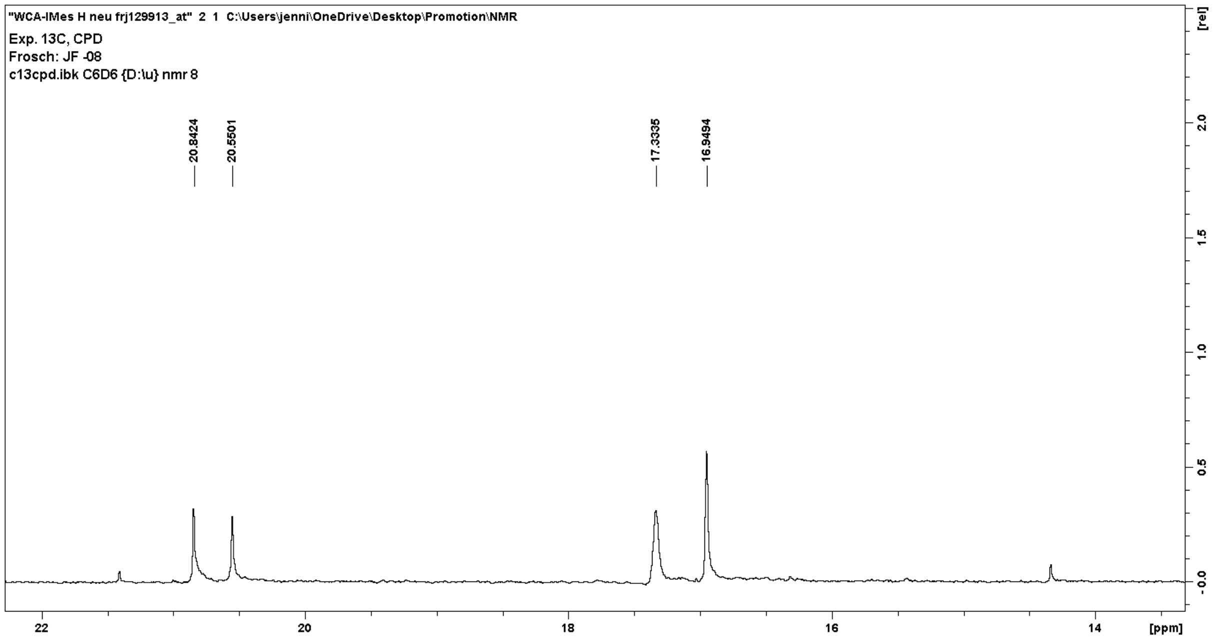

^{11}**B{^{1}H}-NMR** (128 MHz, C_6D_6) of **9b**:

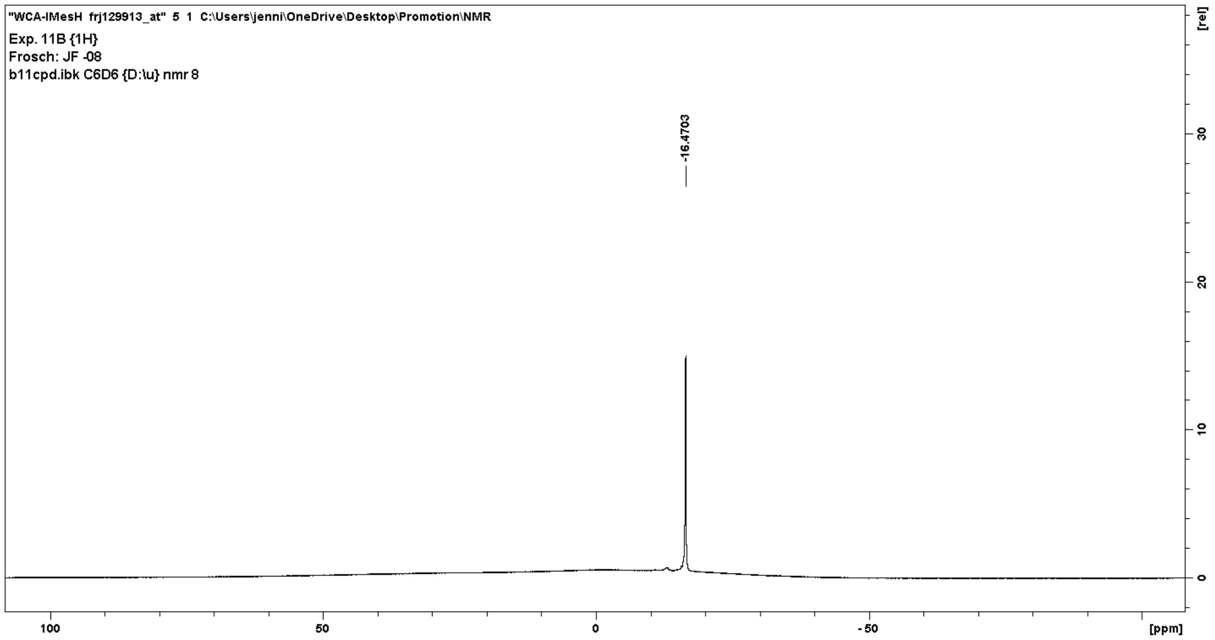

^{19}F{^{1}H}-NMR (376 MHz, C_6D_6) of **9b**:

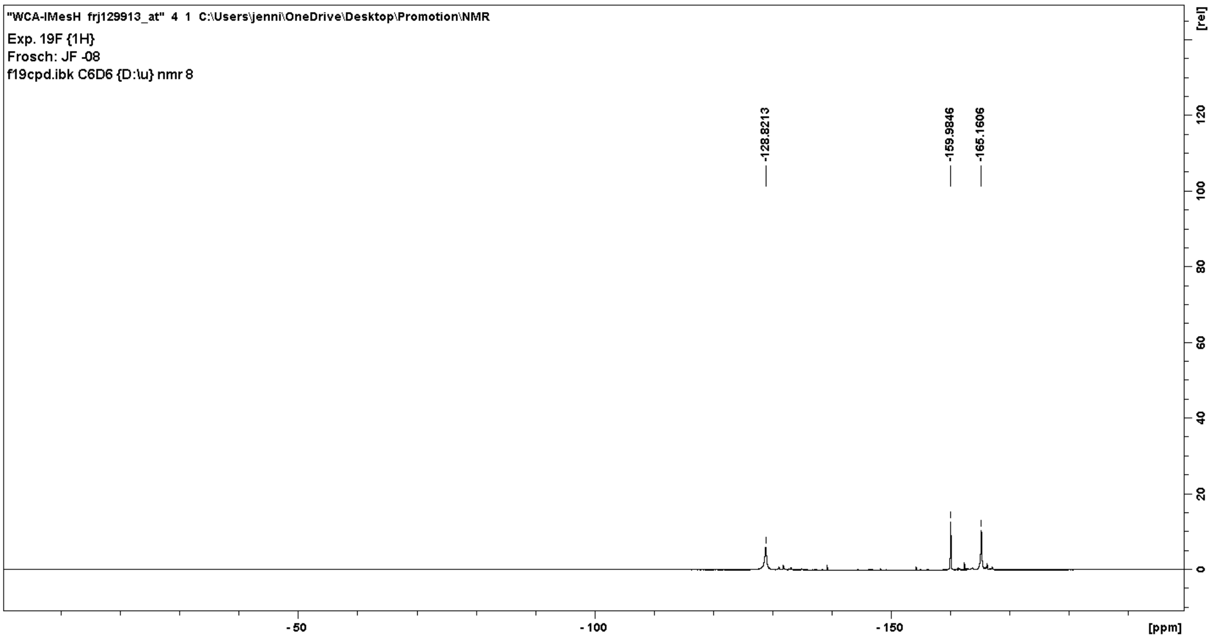

3. X-ray crystal structure determinations

For a summary of crystal data, see Table S1 (parts 1-3). Crystals were mounted on either glass fibers, human hair, MiTiGen or Hampton mounts in perfluorinated inert oil. Intensity measurements were performed at 100 K using a Rigaku XtaLAB Synergy S Single Source and an Oxford Diffraction Nova A diffractometer with mirror-focussed Cu*K*α radiation or a Rigaku XtaLAB Synergy S Single Source with mirror-focused Mo*K*α and an Oxford Diffraction Xcalibur Eos diffractometer with monochromated Mo*K*α radiation. Data reduction was performed with the CrysAlisPRO software.[6] Absorption corrections were based on multi-scans or face-indexation using a gaussian grid. The structures solved using either direct methods in SHELXS[7] or intrinsic phasing in SHELXT[8] and were refined anisotropically on F^2 using the program SHELXL.[9] The hydrogen atoms were included either as constituents of idealized rigid methyl groups allowed to rotate but not tip, or using a riding model starting from calculated positions. Further details are given below under the relevant compound.
CCDC 2034606−2034610, 2034617−2034626 and 2034628−2034632 contain the supplementary crystallographic data for this paper. These data are provided free of charge by the Cambridge Crystallographic Data Centre.

3.1. [(WCA-IDipp)I·Chlorobenzene] (2a·C_6H_5Cl)

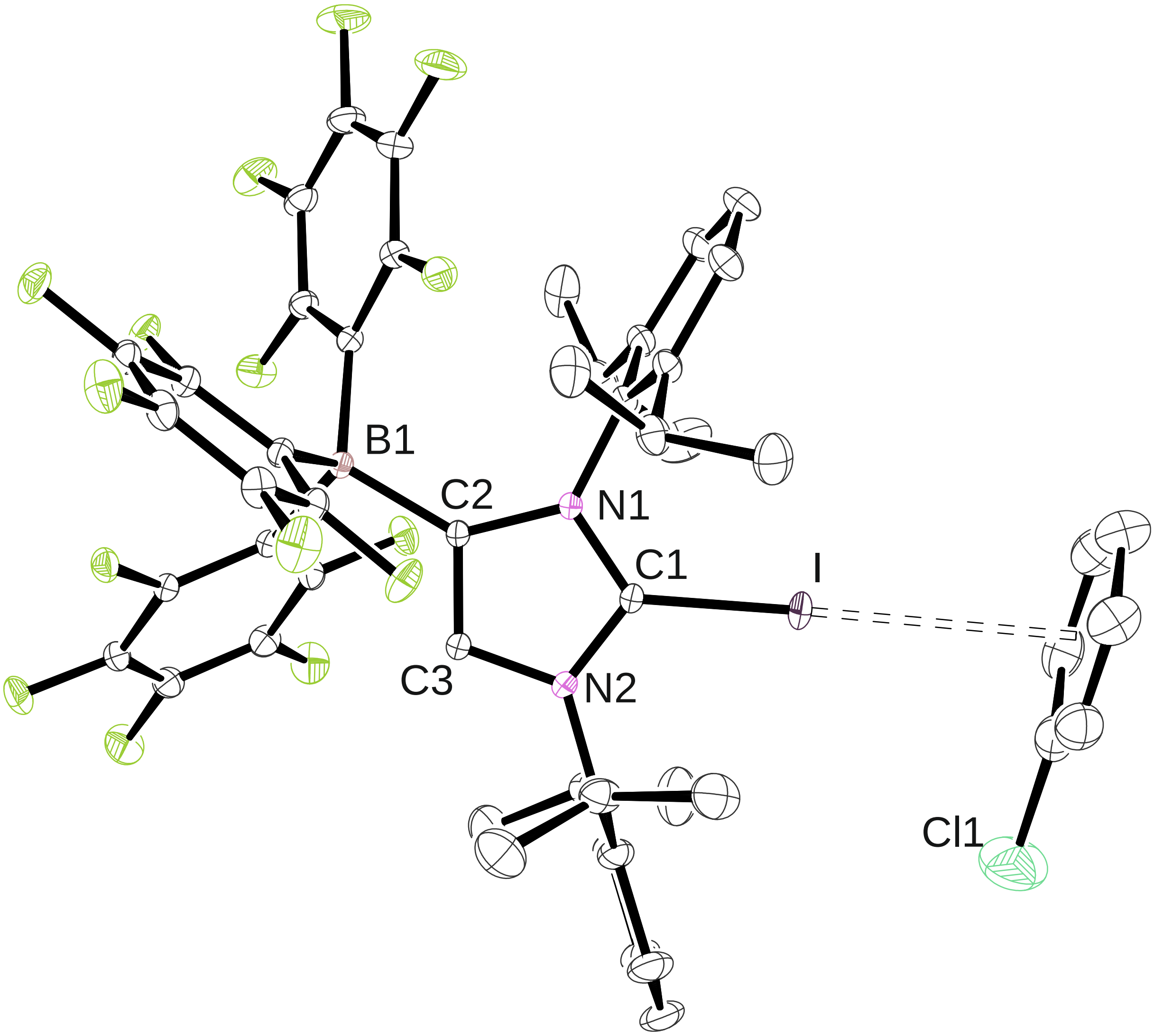

Figure S1. ORTEP diagram of **2a**·C_6H_5Cl with thermal displacement parameters drawn at 50% probability. All hydrogen atoms are omitted for clarity. Selected bond lengths [Å] and angles [°]: C1−I 2.0568(8), C1−N1 1.3491(10), C1−N2 1.3351(11), N1−C2 1.4142(10), C2−C3 1.3684(11), C3−N2 1.3768(10), C2−B1 1.6610(11), N1−C1−N2 108.50(7), N1−C1−I 127.83(6), N2−C1−I 123.54(6), I···$C_{centroid}$ 3.4814(9).

Refinement special details: **2a**·C_6H_5Cl was measured with a detector distance of 40mm.

3.2. [(WCA-IDipp)I·Toluene] ($2a·C_6H_5Me$)

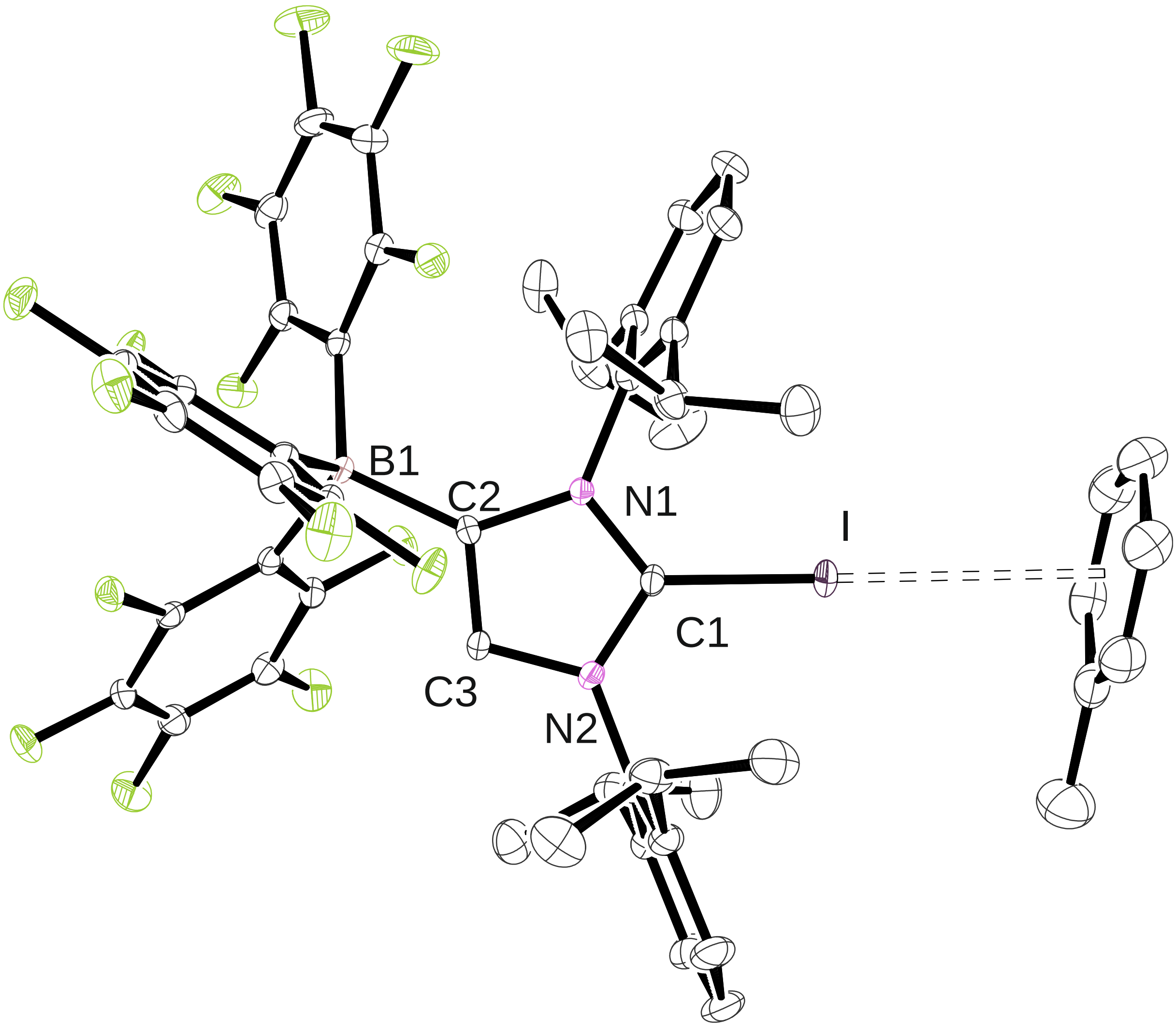

Figure S2. ORTEP diagram of **2a**$·C_6H_5Me$ with thermal displacement parameters drawn at 50% probability. All hydrogen atoms are omitted for clarity. Selected bond lengths [Å] and angles [°]: C1−I 2.0601(13), C1−N1 1.3477(16), C1−N2 1.3347(17), N1−C2 1.4166(16), C2−C3 1.3635(18), C3−N2 1.3771(16), C2−B1 1.6600(19), N1−C1−N2 108.37(11), N1−C1−I 127.93(10), N2−C1−I 123.58(9), I···$C_{centroid}$ 3.4171(10).

3.3. [(WCA-IDipp)I·THF] (2a·THF)

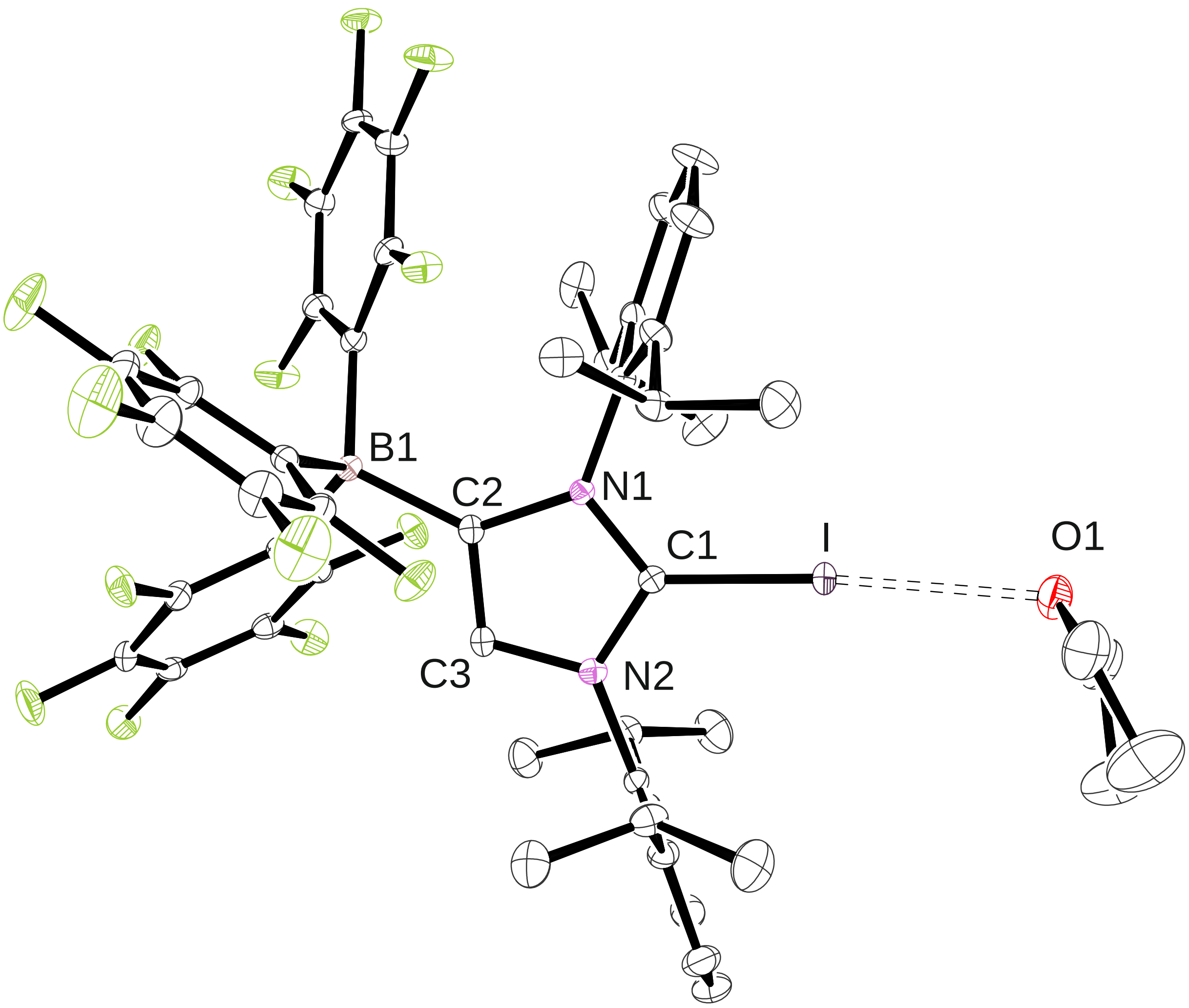

Figure S3. ORTEP diagram of **2a**·THF with thermal displacement parameters drawn at 50% probability. All hydrogen atoms are omitted for clarity. Selected bond lengths [Å] and angles [°]: C1−I 2.065(2), C1−N1 1.351(3), C1−N2 1.335(3), N1−C2 1.422(3), C2−C3 1.363(4), C3−N2 1.384(3), C2−B1 1.660(4), N1−C1−N2 108.4(2), N1−C1−I 128.07(17), N2−C1−I 123.54(17), I···O1 2.743(2).

3.4. [(WCA-IDipp)I·ONMe$_3$] (2a·ONMe$_3$)

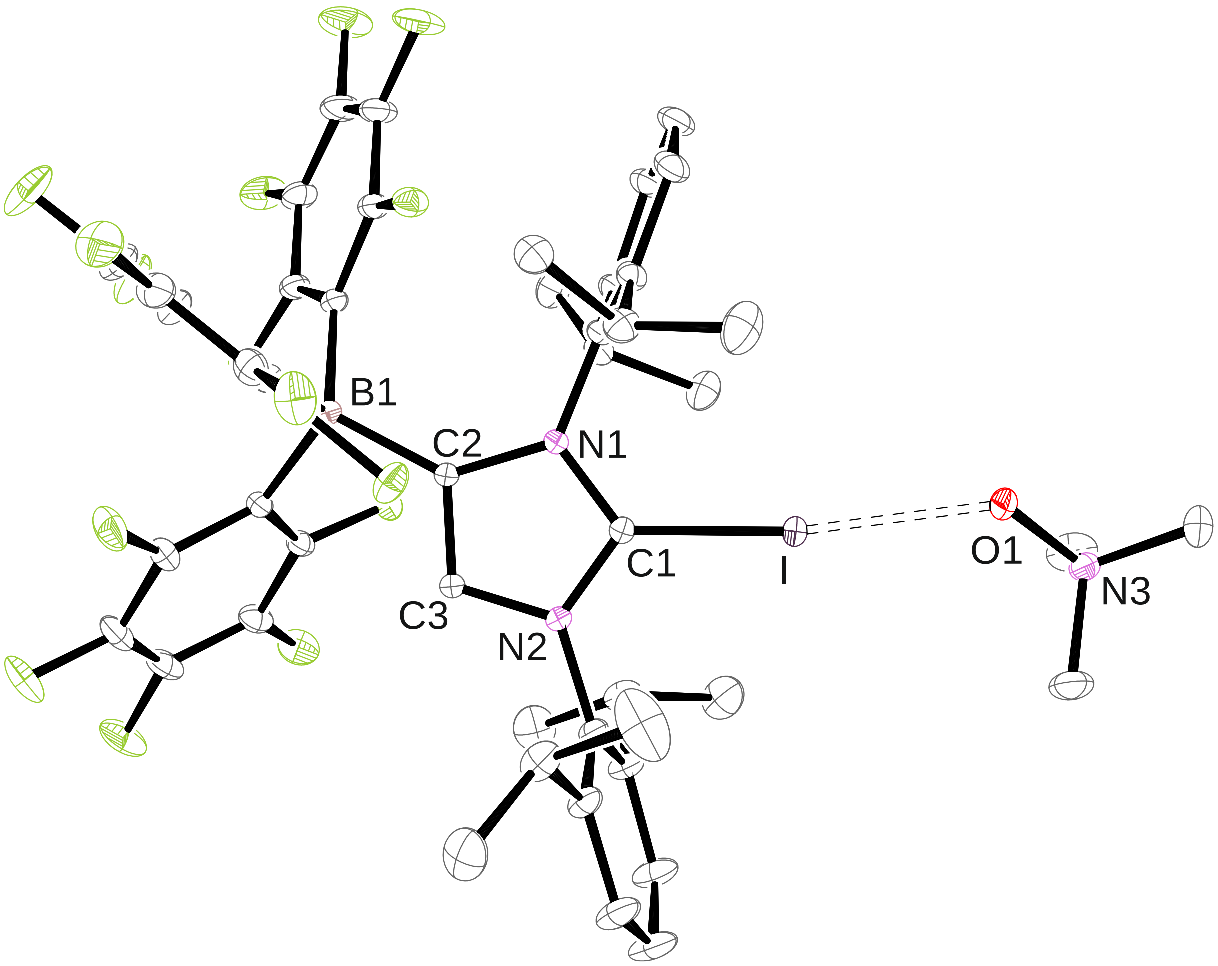

Figure S4. ORTEP diagram of **2a·ONMe$_3$**·*n*-hexane with thermal displacement parameters drawn at 50% probability. All hydrogen atoms are omitted for clarity. Selected bond lengths [Å] and angles [°]: C1−I 2.1091(8), I···O 2.5341(9), C1−N1 1.3544(10), C1−N2 1.3373(10), N1−C2 1.4195(10), C2−C3 1.3673(10), C3−N2 1.3804(10), C2−B1 1.6649(11), N1−C1−N2 107.33(7), N1−C1−I 127.36(6), N2−C1−I 125.30(6), C1−I−O1 171.68(3).

Refinement special details

One molecule of *n*-hexane was refined as disordered on an inversion center. To achieve a stable refinement of this fragement, several restraints were employed using the FragmentDB[10] as implemented in OLEX2[11] and the occupation was fixed at 0.5.

3.5. [(WCA-IDipp)I·CH_3CN] (2a·CH_3CN)

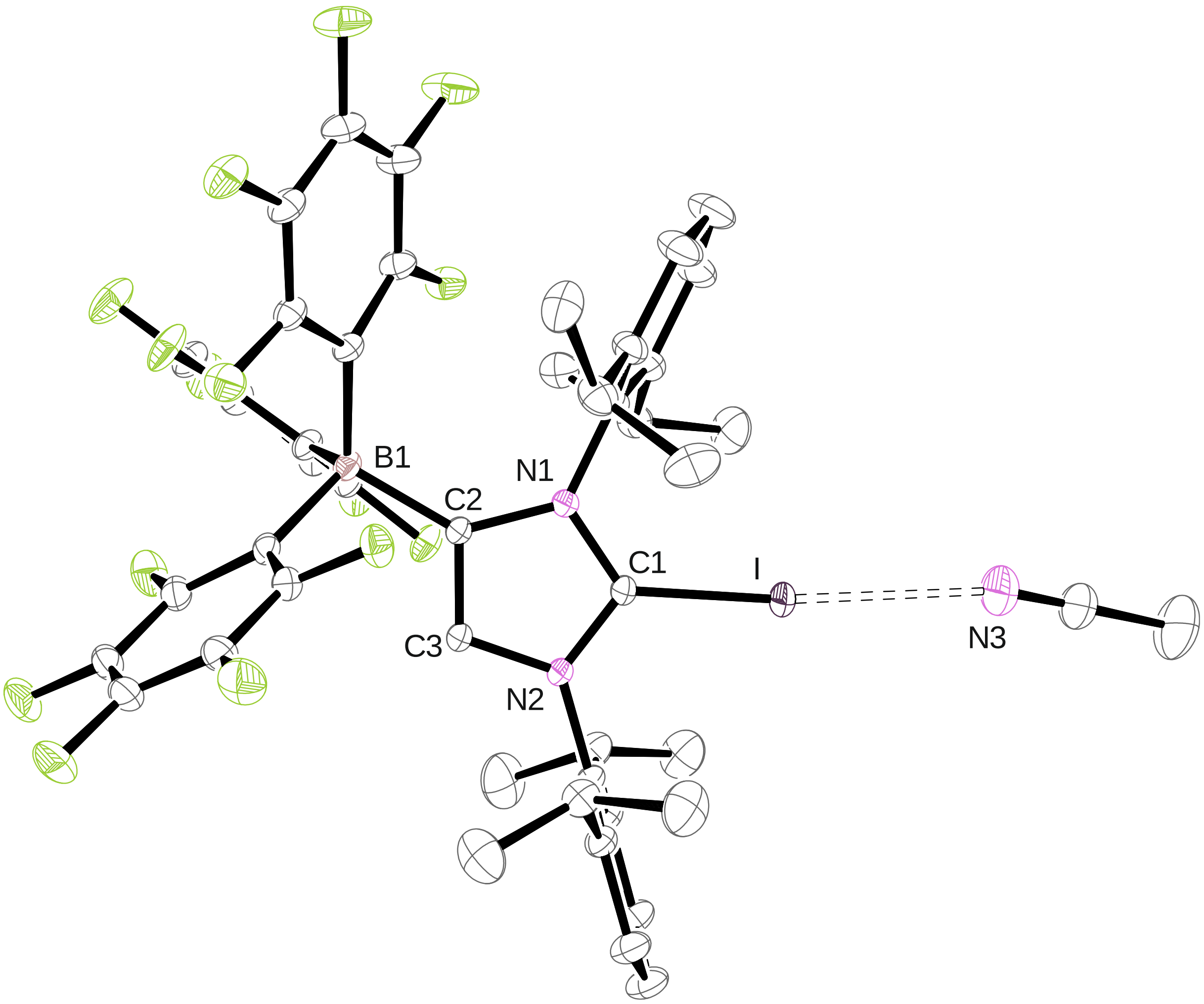

Figure S5. ORTEP diagram of **2a·CH_3CN**·CH_3CN·C_6H_5Cl with thermal displacement parameters drawn at 50% probability. All hydrogen atoms and solvent molecules are omitted for clarity. Selected bond lengths [Å] and angles [°]: C1−I 2.0637(12), I···N3 2.9156(17), C1−N1 1.3532(15), C1−N2 1.3339(16), N1−C2 1.4180(15), C2−C3 1.3701(16), C3−N2 1.3789(15), C2−B1 1.6602(17), N1−C1−N2 108.16(10), N1−C1−I 127.37(9), N2−C1−I 124.46(8), C1−I−N3 171.71(6).

Refinement special details

The Chlorobenzene molecule was refined using the ISOR restraint. A second position was checked but could not be refined satisfactorily.

3.6. [(WCA-IMes)I] (2b):

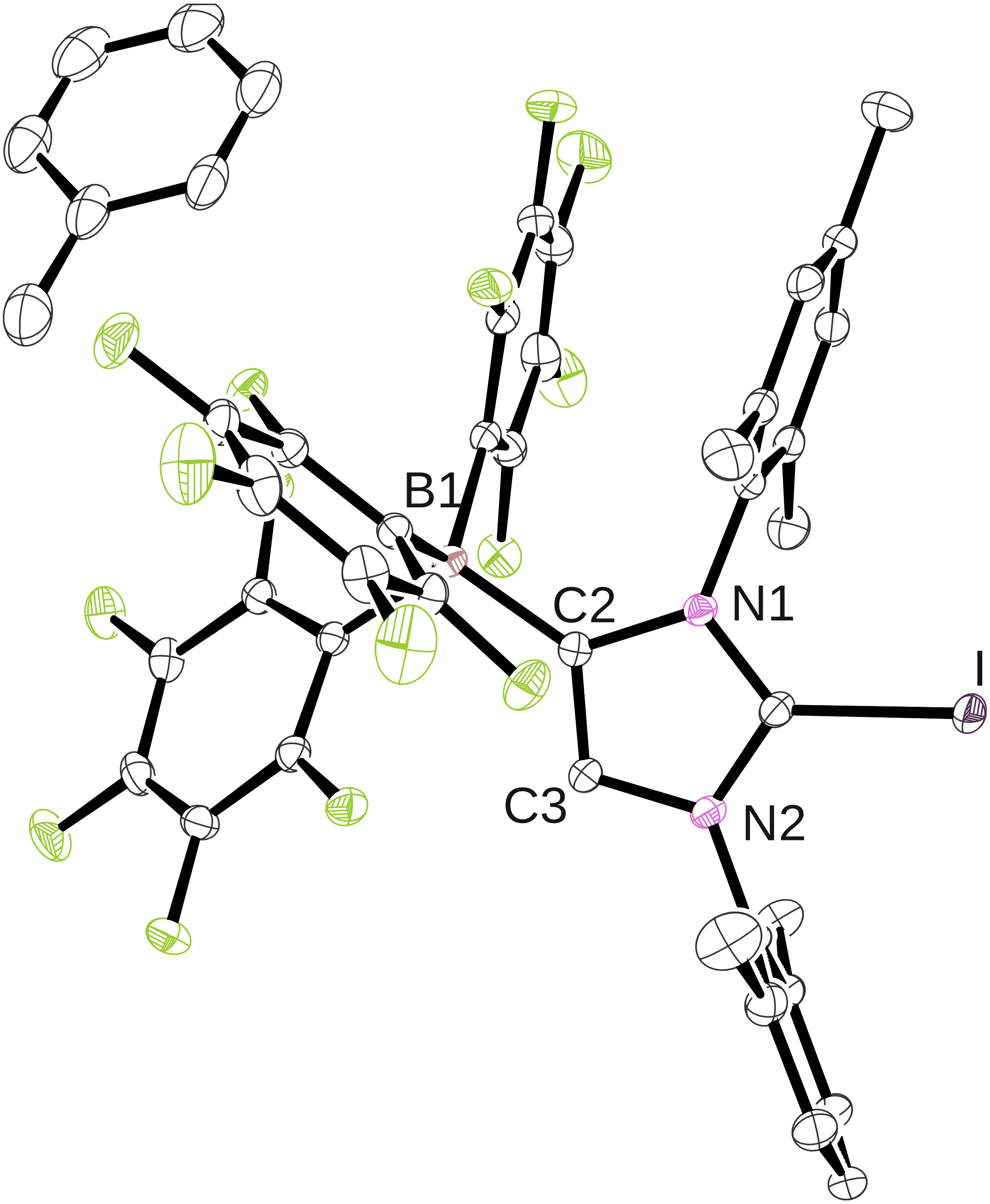

Figure S6. ORTEP diagram of **2b** with thermal displacement parameters drawn at 50% probability. All hydrogen atoms are omitted for clarity. Selected bond lengths [Å] and angles [°]: C1−I 2.0536(13), C1−N1 1.3458(16), C1−N2 1.3340(16), N1−C2 1.4222(16), C2−C3 1.3628(17), C3−N2 1.3848(16), C2−B1 1.6467(19), N1−C1−N2

108.51(11), N1−C1−I 128.03(9), N2−C1−I 123.44(9), I···$C_{centroid}$ 3.5422(6), C1−I···$C_{centroid}$ 161.53(5).

Packing of 2b:

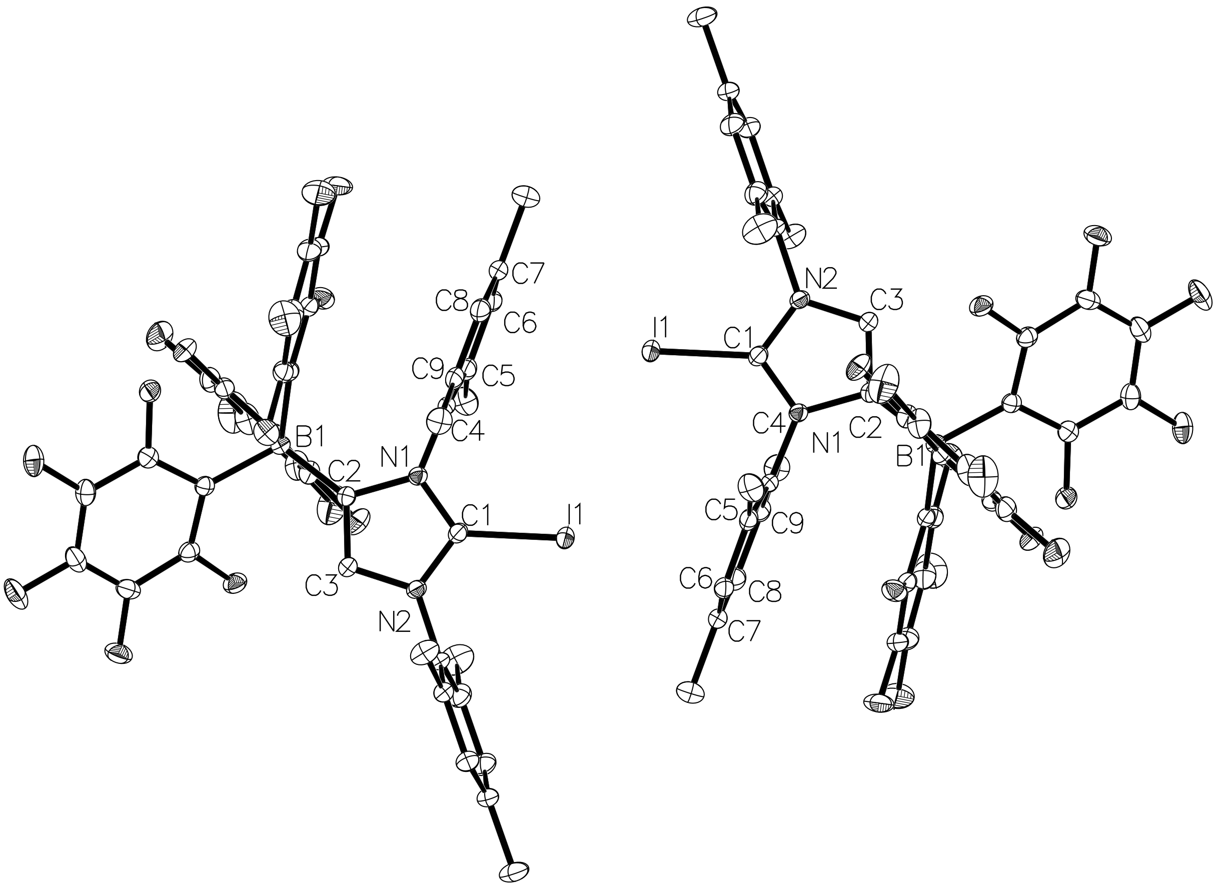

Figure S7. Diagram of two interacting molecules of **2b** with thermal displacement parameters drawn at 50% probability. All hydrogen atoms are omitted for clarity.

3.7. [(*m*-XyF$_6$)$_3$B(IDipp)I] (2c)

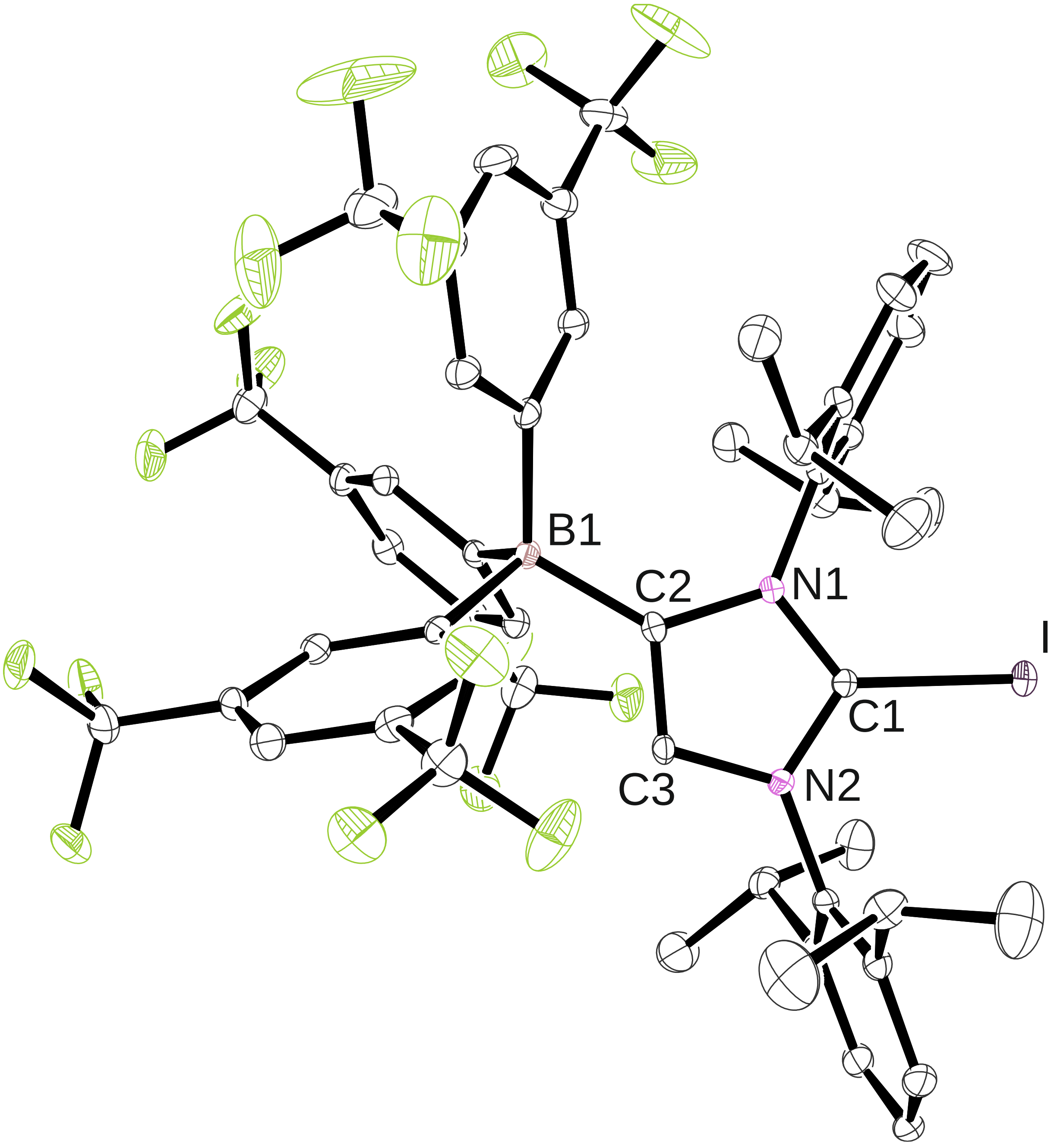

Figure S8. ORTEP diagram of **2c** with thermal displacement parameters drawn at 50% probability. All hydrogen atoms are omitted for clarity. Selected bond lengths [Å] and angles [°]: C1−I 2.0544(16), C1−N1 1.347(2), C1−N2 1.337(2), N1−C2 1.412(2), C2−C3 1.369(2), C3−N2 1.388(2), C2−B1 1.648(2), N1−C1−N2 108.43(14), N1−C1−I 126.75(12), N2−C1−I 124.81(12), I···$C_{centroid}$ 3.6444(9).

Packing of 2c:

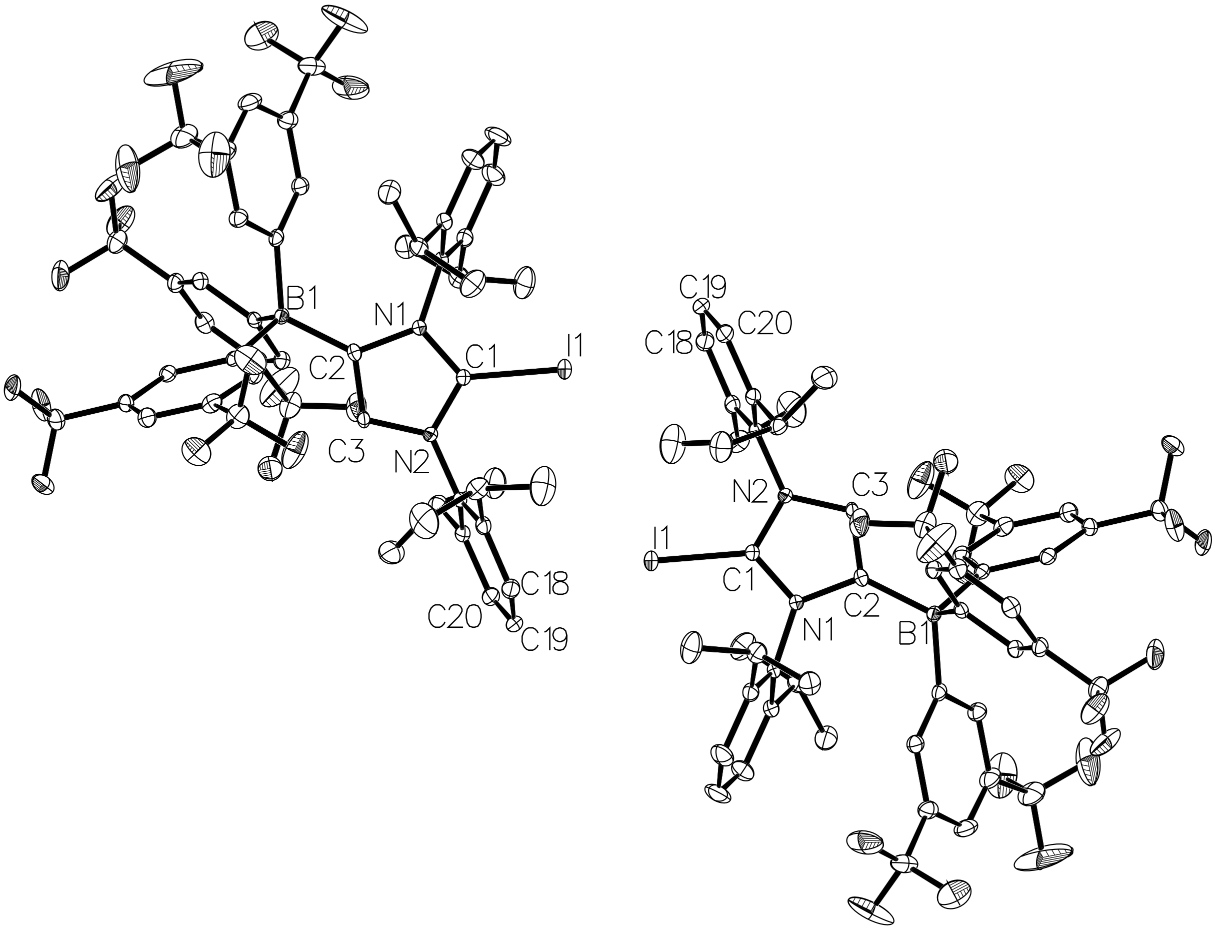

Figure S9. Diagram of two interacting molecules of **2c** with thermal displacement parameters drawn at 50% probability. All hydrogen atoms are omitted for clarity.

3.8. [(WCA-IDipp)Br] (3a)

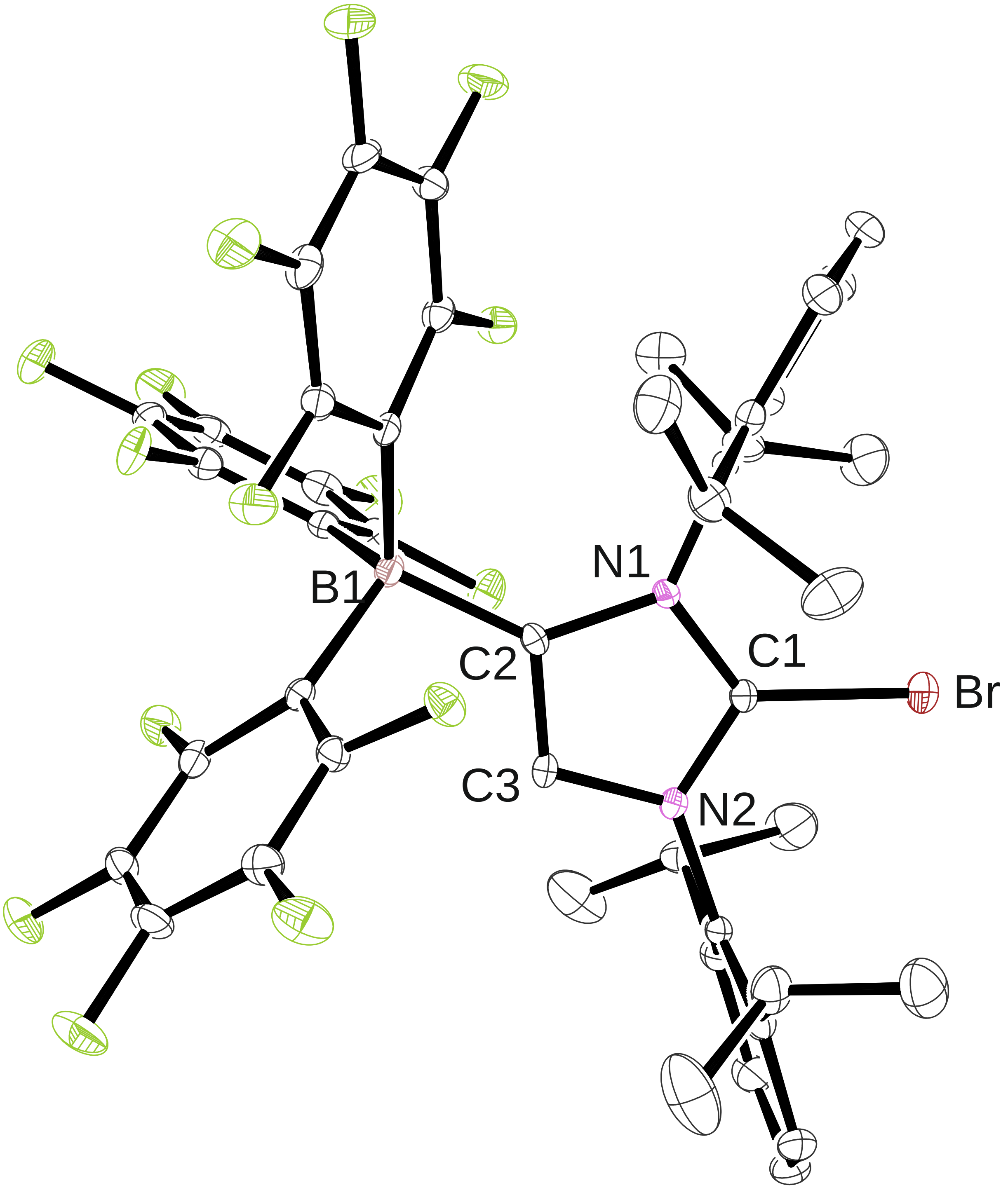

Figure S10. ORTEP diagram of **3a** with thermal displacement parameters drawn at 50% probability. All hydrogen atoms are omitted for clarity. Selected bond lengths [Å] and angles [°]: C1−Br 1.845(2), C1−N1 1.344(3), C1−N2 1.323(3), N1−C2 1.422(3), C2−C3 1.366(3), C3−N2 1.382(2), C2−B1 1.652(3), N1−C1−N2 110.22(17), N1−C1−Br 126.57(16), N2−C1−Br 123.18(15), Br···F_3 3.1824(19).

Refinement special details

Solvent accessible void shows no significant electron density. Squeeze was not used.

Packing of 3a:

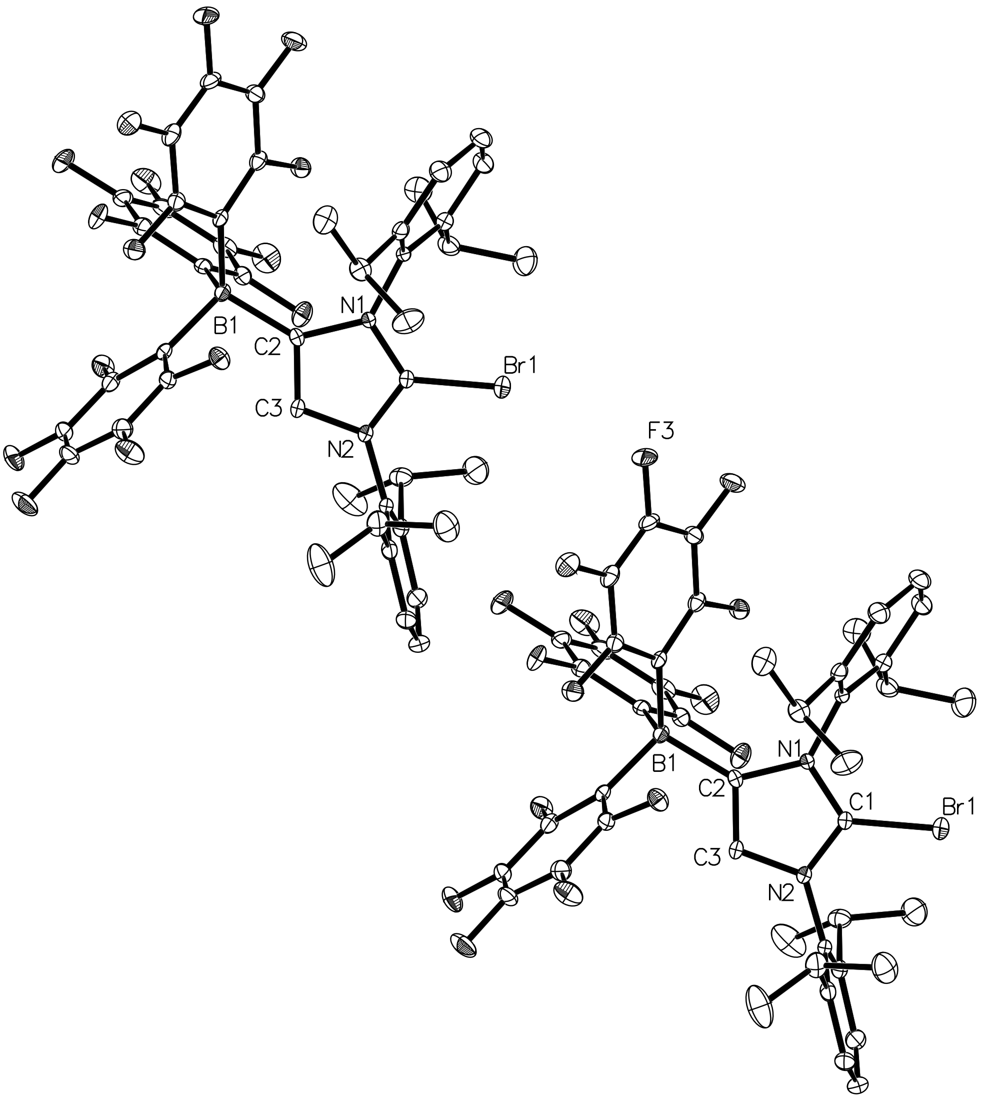

Figure S11. Diagram of two interacting molecules of **3a** with thermal displacement parameters drawn at 50% probability. All hydrogen atoms are omitted for clarity.

3.9. [(WCA-IMes)Br] (3b)

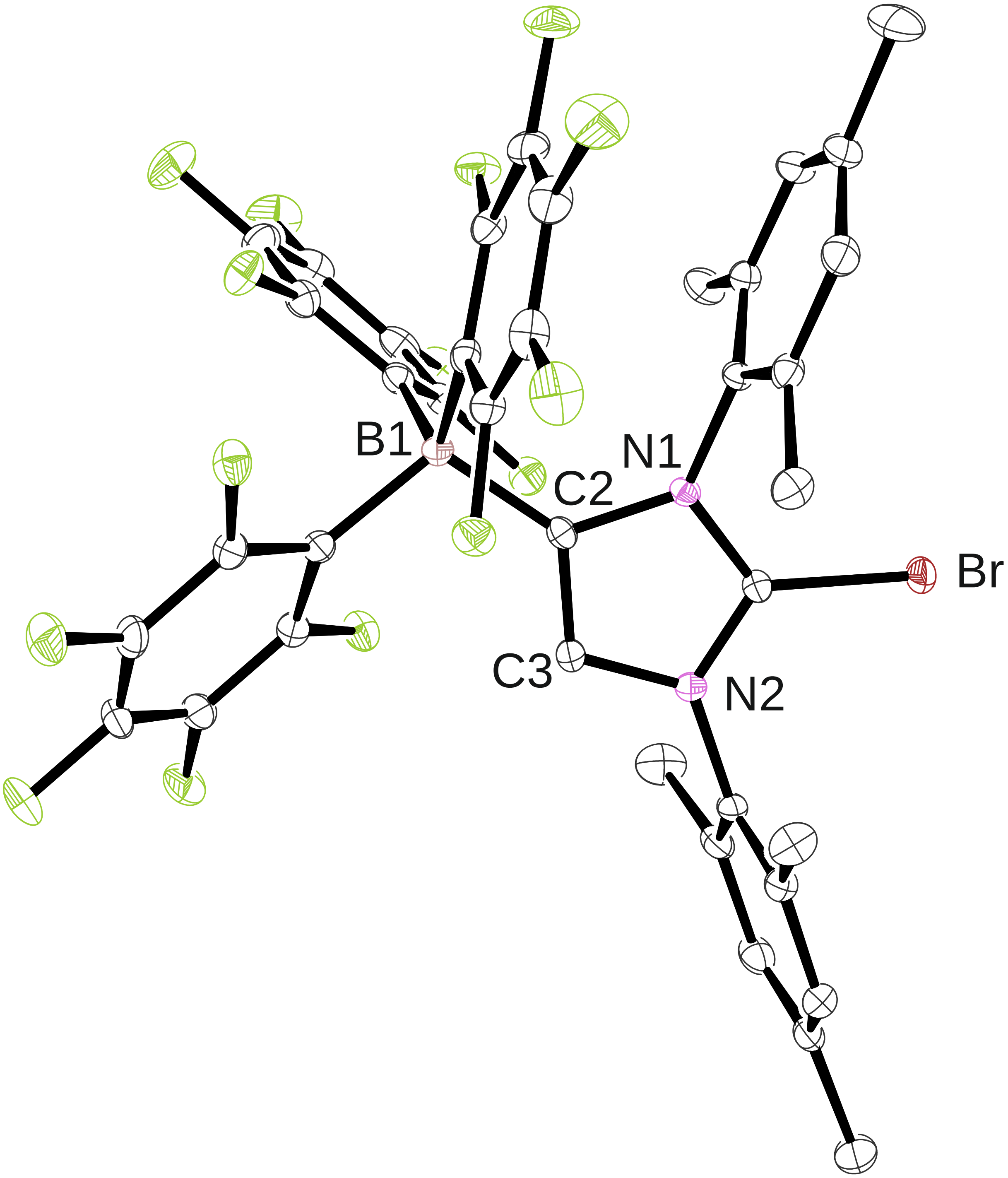

Figure S12. ORTEP diagram of **3b** with thermal displacement parameters drawn at 50% probability. All hydrogen atoms are omitted for clarity. Selected bond lengths [Å] and angles [°]: C1−Br 1.8396(15), C1−N1 1.3388(19), C1−N2 1.3317(19), N1−C2 1.4206(19), C2−C3 1.360(2), C3−N2 1.360(2), C2−B1 1.640(2), N1−C1−N2 109.47(13), N1−C1−Br 124.77(11), N2−C1−Br 125.72(11), Br···C17 3.3925(17) Br···C18 3.4470(17).

Packing of 3b:

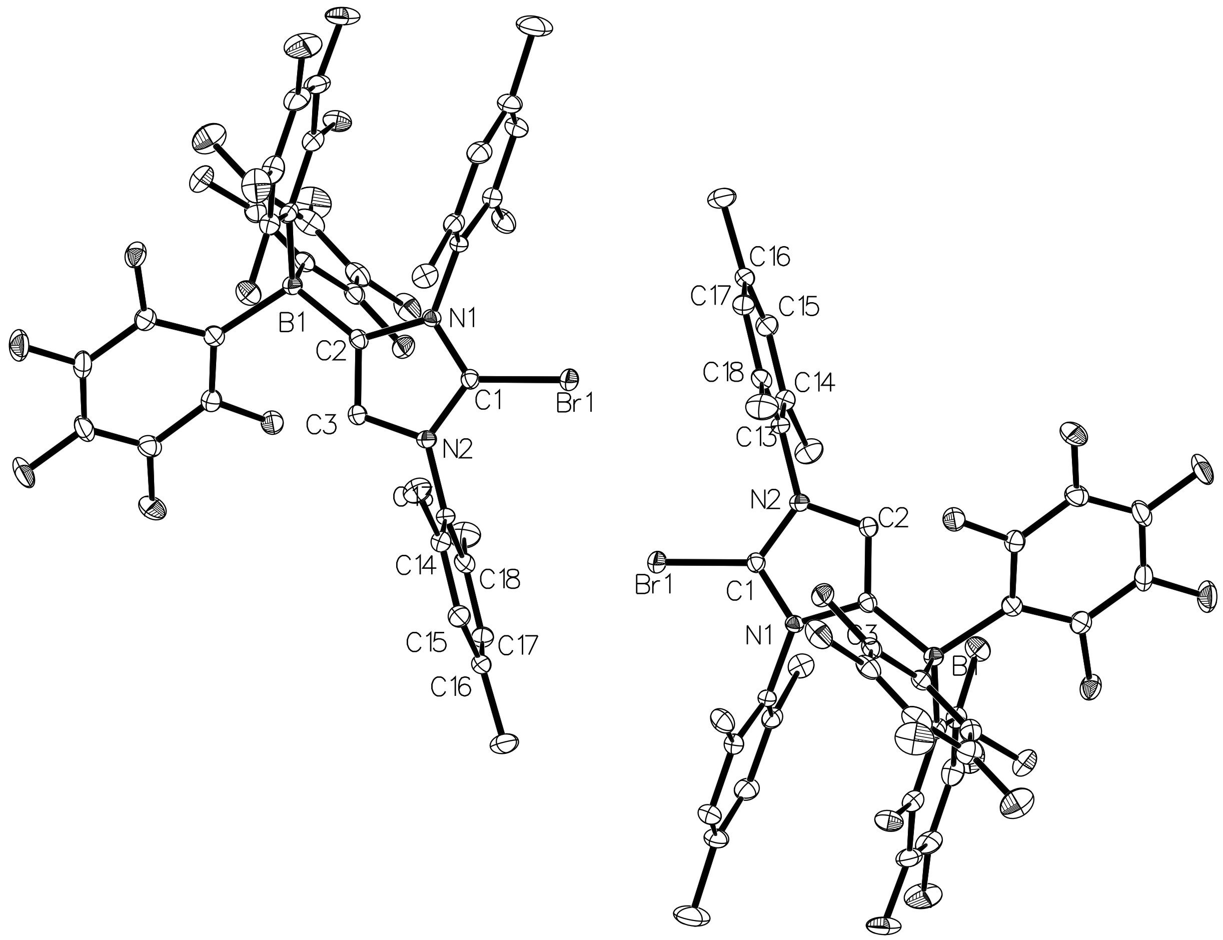

Figure S13. Diagram of two interacting molecules of **3b** with thermal displacement parameters drawn at 50% probability. All hydrogen atoms are omitted for clarity.

3.10. [(WCA-IDipp)Cl] (4)

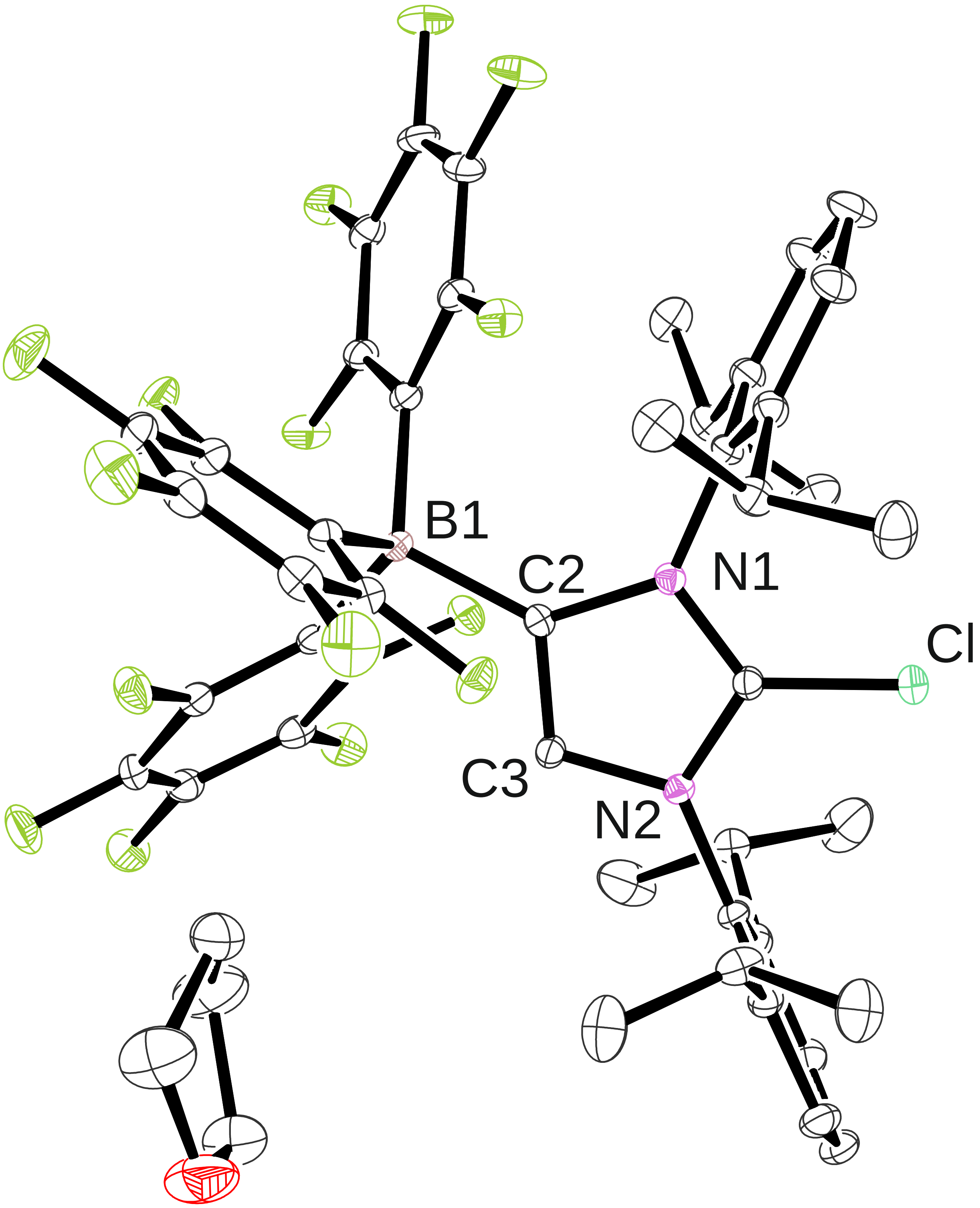

Figure S14. ORTEP diagram of **4·THF** with thermal displacement parameters drawn at 50% probability. All hydrogen atoms are omitted for clarity. Selected bond lengths [Å] and angles [°]: C1−Cl 1.6825(13), C1−N1 1.3406(16), C1−N2 1.3230(16), N1−C2 1.4228(16), C2−C3 1.3681(17), C3−N2 1.3820(16), C2−B1 1.6577(18), N1−C1−N2 110.24(11), N1−C1−Cl 126.99(10), N2−C1−Cl 122.76(10), Cl···Cl 3.2665(8).

Packing of 4:

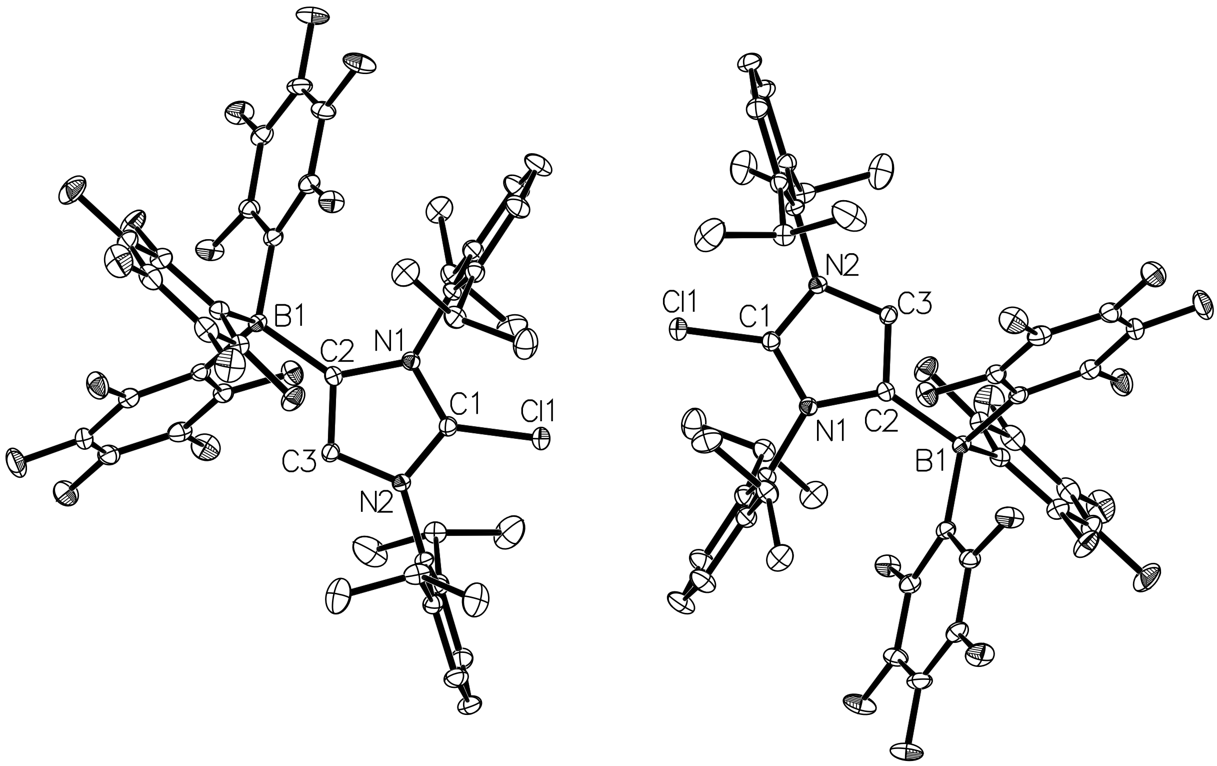

Figure S15. Diagram of two interacting molecules of **4** with thermal displacement parameters drawn at 50% probability. All hydrogen atoms are omitted for clarity.

3.11. [(WCA-IDipp)I(IDipp)] (5a)

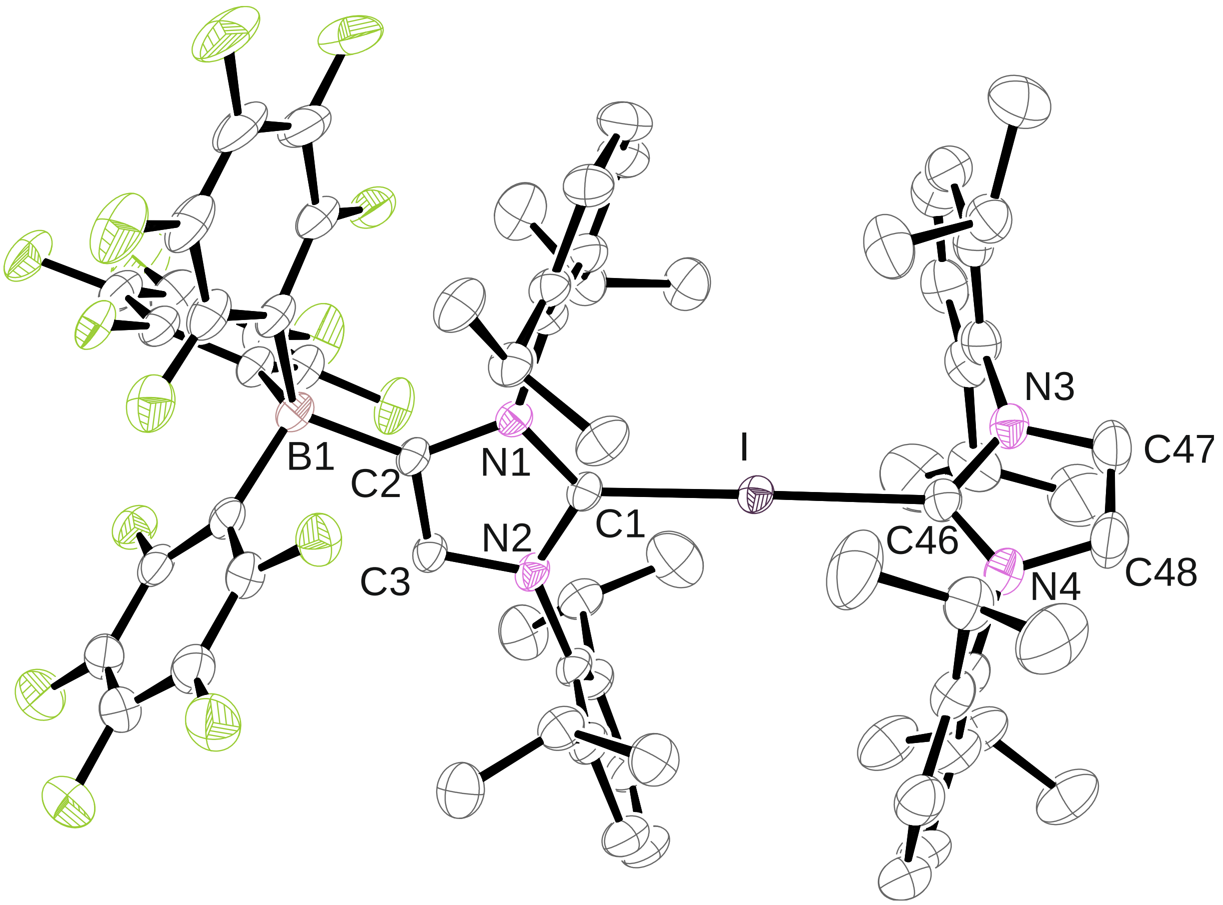

Figure S16. ORTEP diagram of **5a·[Solvent]$_n$** with thermal displacement parameters drawn at 50% probability. All hydrogen atoms are omitted for clarity. Selected bond lengths [Å] and angles [°]: C1−I 2.260(2), C46−I 2.441(2), C1−N1 1.364(3), C1−N2 1.336(3), N1−C2 1.424(3), C2−C3 1.362(3), C3−N2 1.383(3), C2−B1 1.660(3), C46−N3 1.350(3), C46−N4 1.358(3), C47−N3 1.391(3), C47−C48 1.336(4), C48−N4 1.389(3), N1−C1−N2 106.15(18), N1−C1−I 130.26(16), N2−C1−I 122.96(14), N3−C46−N4 104.1(2), N3−C46−I 126.03(16), N4−C46−I 129.56(16), C1−I−C46 177.02(7), interplanar angle of the NHC planes 49.70(9).

Refinement special details

Any attempts to model one of the chlorobenzenes or the *n*-hexane revealed at least two positions per molecule, which were additionally disordered over the inversion center, and therefore no stable refinement could be achieved. A solvent mask (aka SQUEEZE)[12] was calculated, as implemented in OLEX2,[11] and 157 electrons were found in a volume of 661 Å^3 in 1 void per unit cell. This is consistent with the presence of 1[C_6H_5Cl], 0.5[C_6H_{14}] per Asymmetric Unit which account for 166 electrons per unit

cell.

3.12. [(WCA-IDipp)I(IMes)] (5b)

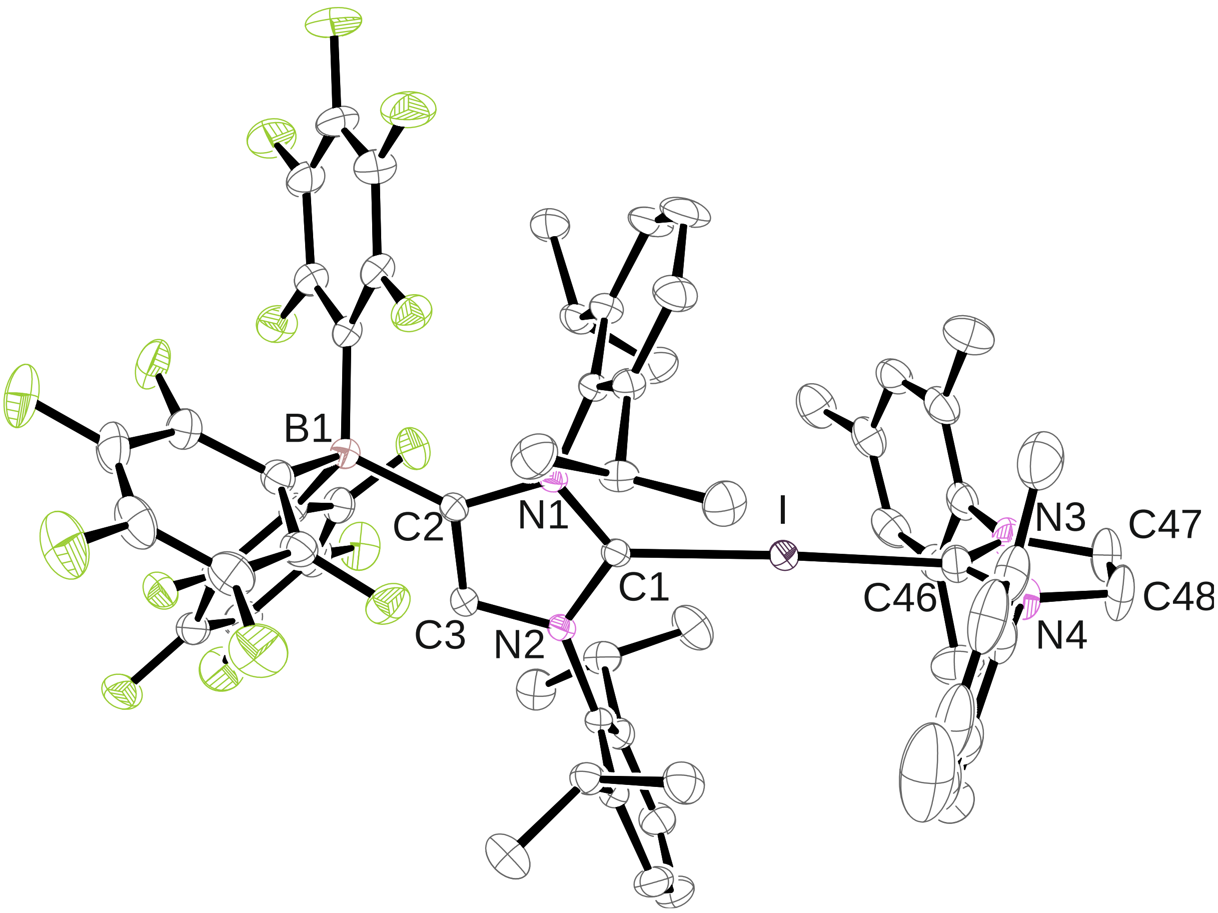

Figure S17. ORTEP diagram of **5b**·C_6H_5Cl·*n*-hexane with thermal displacement parameters drawn at 50% probability. All hydrogen atoms and solvent molecules are omitted for clarity. Selected bond lengths [Å] and angles [°]: C1−I 2.288(2), C46−I 2.361(2), C1−N1 1.355(3), C1−N2 1.339(3), N1−C2 1.425(3), C2−C3 1.363(3), C3−N2 1.380(3), C2−B1 1.656(3), C46−N3 1.383(3), C46−N4 1.346(3), C47−N3 1.383(3), C47−C48 1.340(4), C48−N4 1.386(3), N1−C1−N2 106.07(17), N1−C1−I 130.34(14), N2−C1−I 123.45(14), N3−C46−N4 105.2(2), N3−C46−I 127.66(18), N4−C46−I 127.15(17), C1−I−C46 177.44(8), interplanar angle of the NHC planes 92.78(10).

Refinement special details

One chlorobenzene molecule is disordered over a special position and therefore the occupation was restrained to 0.5. Additionally, several restraints were employed to yield a stable refinement of the solvent (chlorobenzene, *n*-hexane).

3.13. [PPh$_4$][WCA-IDipp] (6)

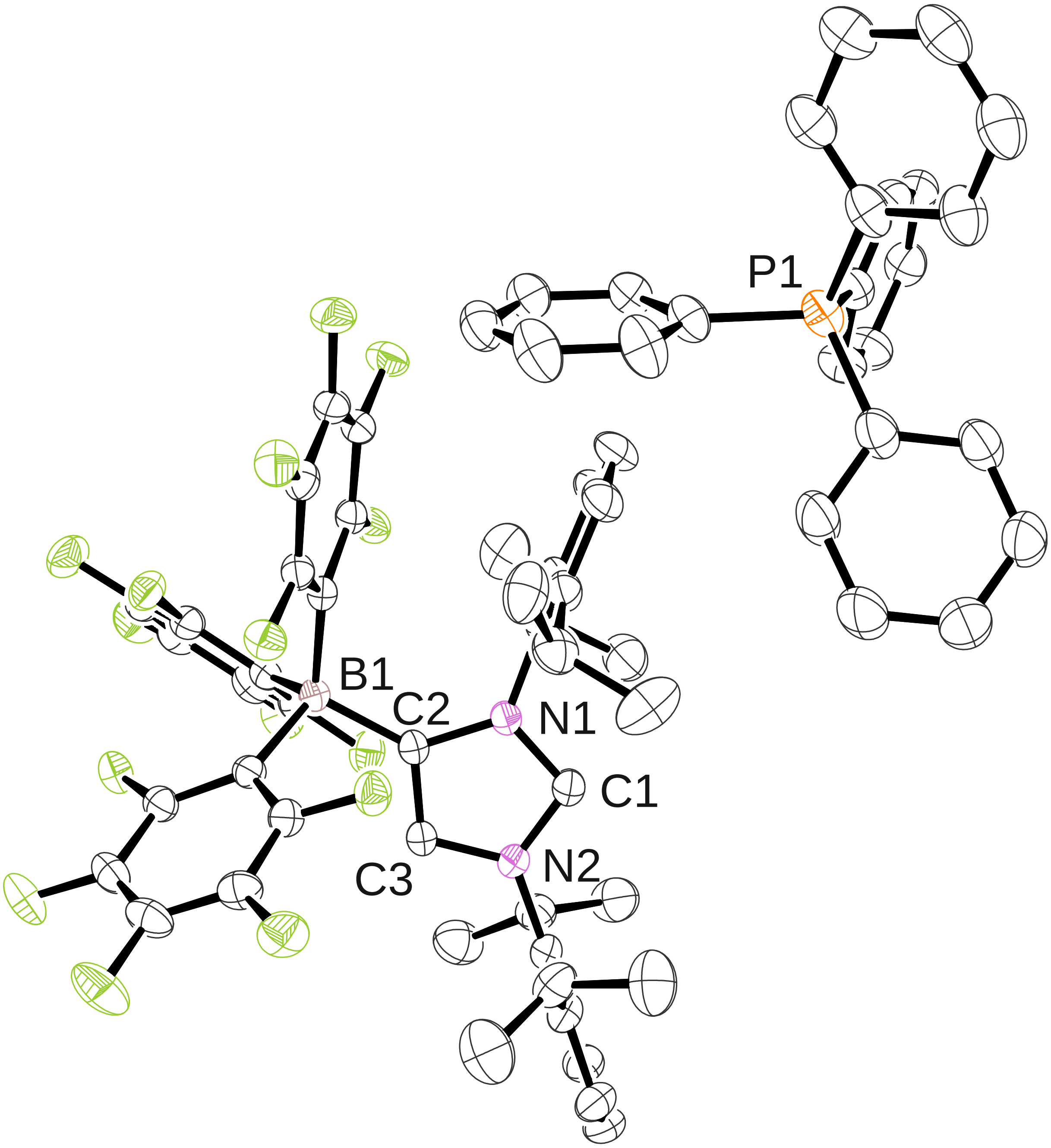

Figure S18. ORTEP diagram of **6** with thermal displacement parameters drawn at 50% probability. All hydrogen atoms are omitted for clarity. Selected bond lengths [Å] and angles [°]: C1−N1 1.3739(18), C1−N2 1.3586(19), N1−C2 1.4164(17), C2−C3 1.3512(19), C3−N2 1.3853(17), C2−B1 1.6416(19), N1−C1−N2 101.93(11), C1−N1−C2 113.89(11), N1−C2−C3 102.97(11), C2−C3−N2 109.05(12), C3−N2−C1 112.15(11), N1−C2−B1 133.29(11), C3−C2−B1 123.56(12).

3.14. $[PPh_4][(WCA\text{-}IDipp)_2I]$ (7a)

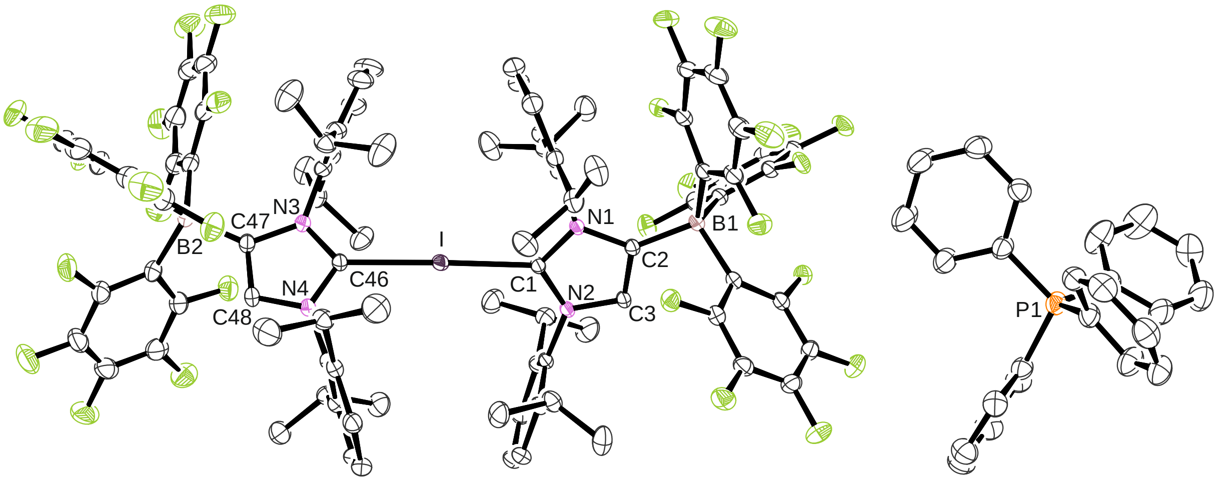

Figure S19. ORTEP diagram of **7a·(THF)₂** with thermal displacement parameters drawn at 50% probability. All hydrogen atoms and THF molecules are omitted for clarity. Selected bond lengths [Å] and angles [°]: C1−I 2.3373(11), C46−I 2.4018(12), C1−N1 1.3637(14), C1−N2 1.3415(15), N1−C2 1.4183(15), C2−C3 1.3630(16), C3−N2 1.3820(15), C2−B1 1.6549(17), C46−N3 1.3609(16), C46−N4 1.3448(15), C47−N3 1.4160(16), C47−C48 1.3611(18), C48−N4 1.3795(16), C47−B2 1.6482(19), N1−C1−N2 105.25(10), N1−C1−I 126.99(10), N2−C1−I 124.69(8), N3−C46−N4 104.85(10), N3−C46−I 130.53(8), N4−C46−I 124.07(8), C1−I−C46 178.48(4), interplanar angle of the NHC planes 42.58(5) .

Refinement special details

One THF molecule is slightly disordered. Disorder was modelled as a 60/40 occupation over two positions.

3.15. $[PPh_4][(WCA\text{-}IDipp)I(WCA\text{-}IMes)]$ (7b)

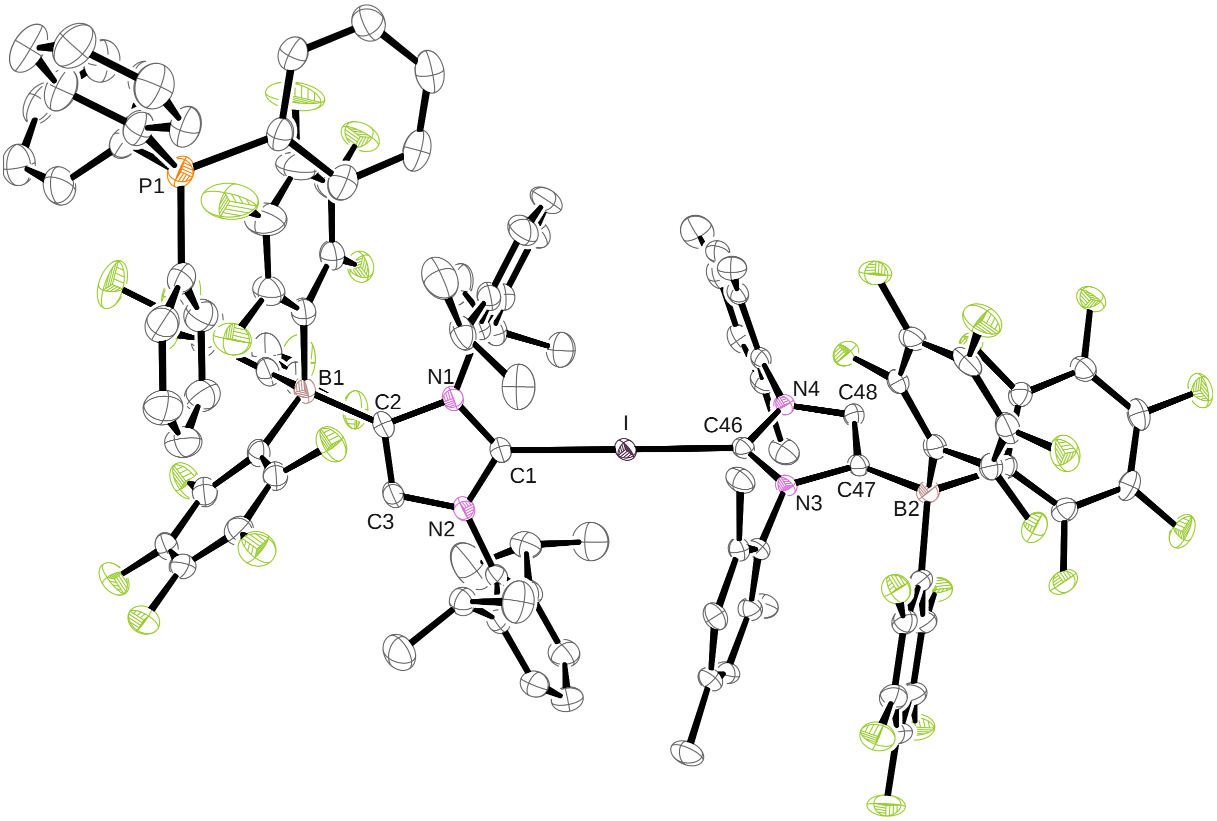

Figure S20. ORTEP diagram of **7b** with thermal displacement parameters drawn at 50% probability. All hydrogen atoms and a second molecule, which is part of the unit cell, are omitted for clarity. Selected bond lengths [Å] and angles [°]: C1−I 2.4055(17)/2.4089(17), C46−I 2.2542(17)/2.2575(17), C1−N1 1.356(2)/1.355(2), C1−N2 1.343(2)/1.339(2), N1−C2 1.425(2)/1.418(2), C2−C3 1.366(3)/1.357(3), C3−N2 1.385(2)/1.383(2), C2−B1 1.652(3)/1.654(3), C46−N3 1.347(2)/1.348(2), C46−N4 1.338(2)/1.342(2), C47−N3 1.423(2)/1.423(2), C47−C48 1.356(2)/1.361(2), C48−N4 1.388(2)/1.388(2), C47−B2 1.635(3)/1.638(3), N1−C1−N2 105.57(15)/105.22(15), N1−C1−I 131.10(12)/132.43(12), N2−C1−I 122.84(12)/ 122.30(12), N3−C46−N4 106.38(14)/106.01(14), N3−C46−I 128.76(12)/129.44(12), N4−C46−I 124.74(12)/ 124.49(12), C1−I−C46 178.71(6)/179.32(6), interplanar angle of the NHC planes 53.13(7)/48.58(7).

Refinement special details

Any attempts to model one of the chlorobenzenes or the *n*-hexane revealed at least two positions per molecule, which were additionally disordered over the inversion center, and therefore no stable refinement could be achieved. A solvent mask (aka

SQUEEZE)[12] was calculated, as implemented in OLEX2,[11] and 157 electrons were found in a volume of 661 Å^3 in 1 void per unit cell. This is consistent with the presence of 1[C_6H_5Cl], 0.5[C_6H_{14}] per Asymmetric Unit which account for 166 electrons per unit cell.

3.16. [PPh_4][((*m*-XyF_6)$_3$B(IDipp))$_2$I] (7d)

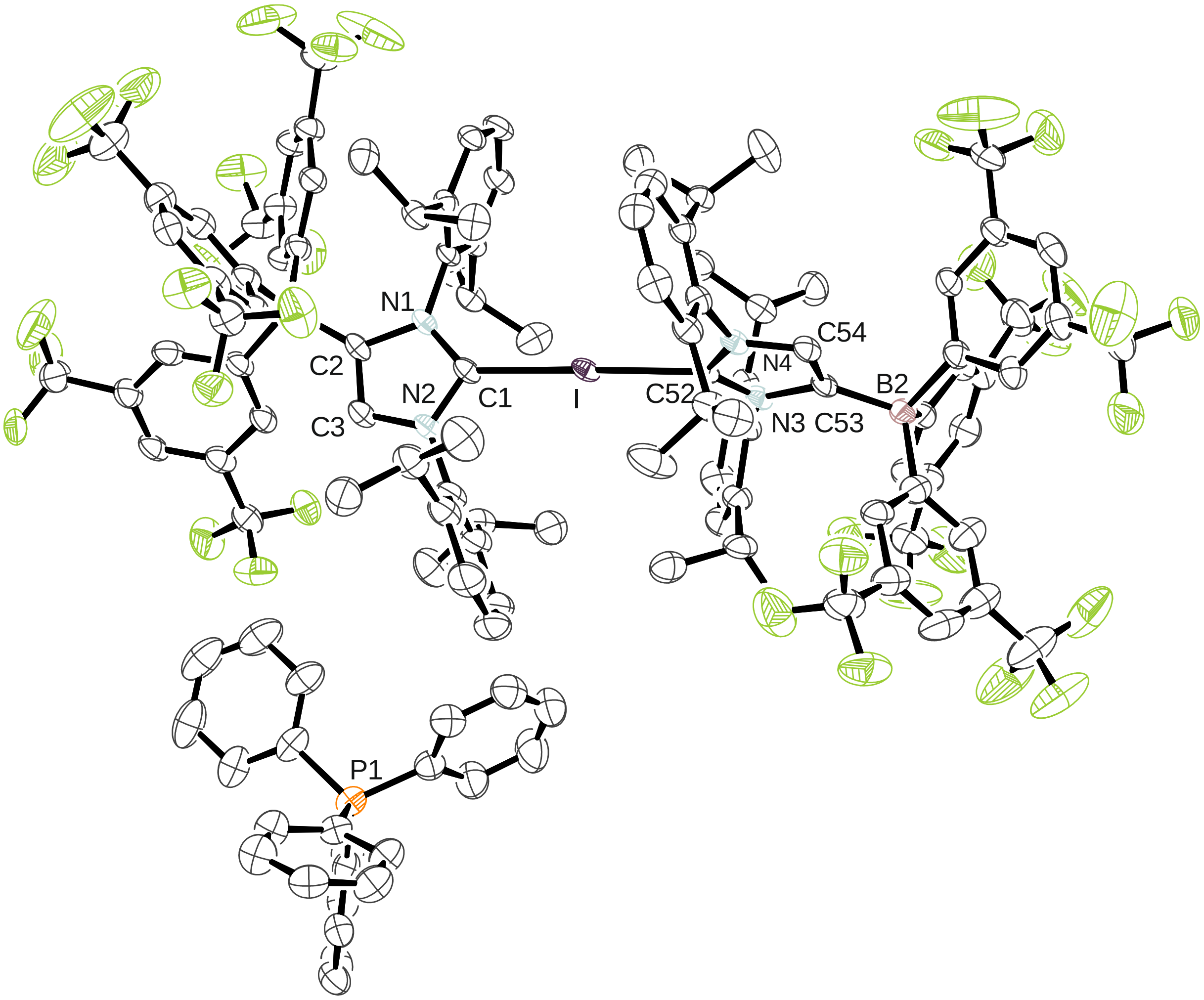

Figure S21. ORTEP diagram of **7d** with thermal displacement parameters drawn at 50% probability. All hydrogen atoms and a second molecule, which is part of the unit cell, are omitted for clarity. A detailed presentation and discussion of the molecular structue is not possible and only the connectivity is revealed due to the low quality of the single crystal.

3.17. $[PPh_4][(WCA\text{-}IDipp)_2Br]$ (8)

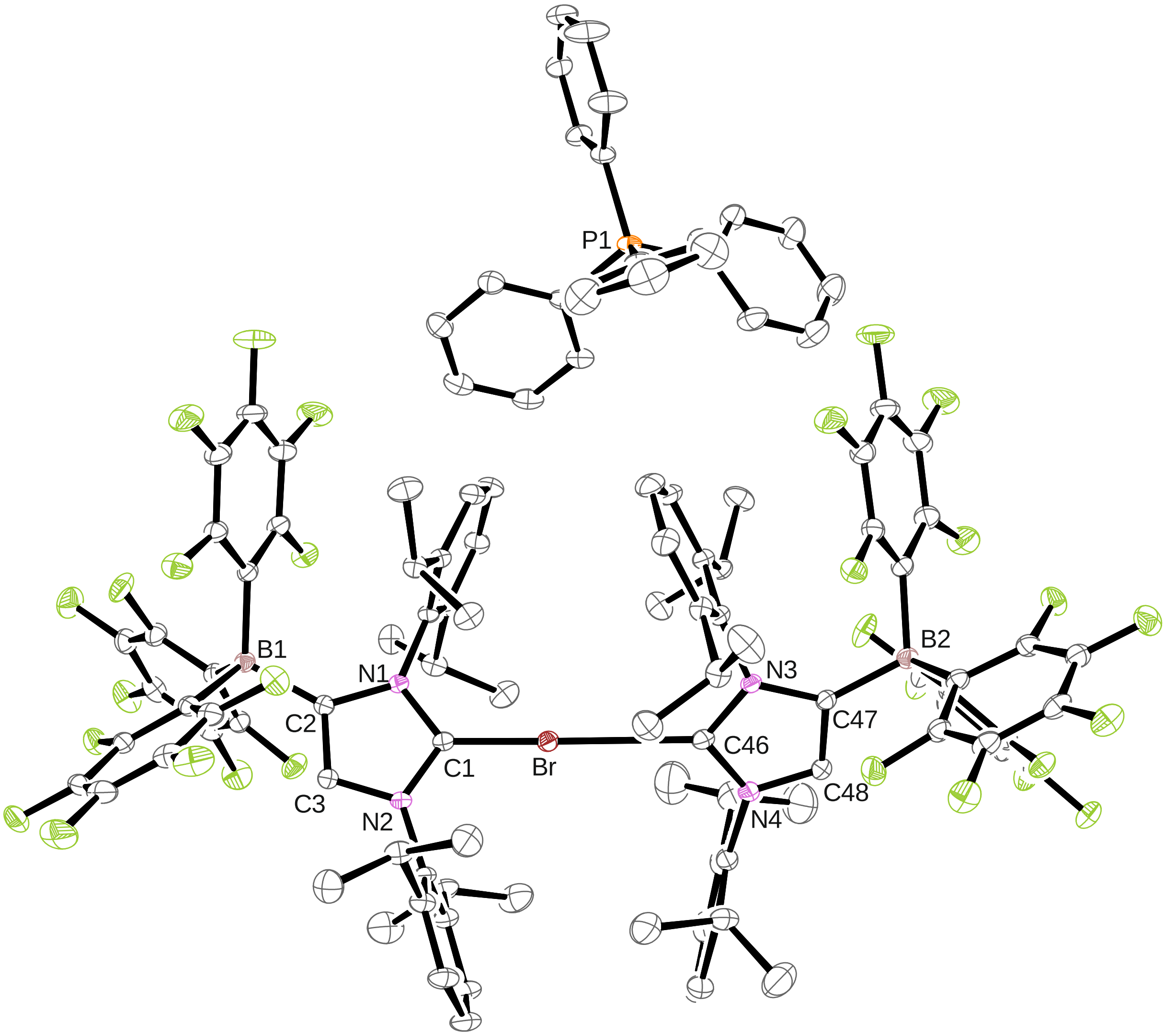

Figure S22. ORTEP diagram of **8**·C_6H_5Cl with thermal displacement parameters drawn at 50% probability. All hydrogen atoms and the solvent molecule are omitted for clarity. Selected bond lengths [Å] and angles [°]: C1−Br 1.9010(18), C46−Br 2.822(2), C1−N1 1.351(2), C1−N2 1.341(2), N1−C2 1.415(2), C2−C3 1.366(2), C3−N2 1.377(2), C2−B1 1.657(3), C46−N3 1.375(2), C46−N4 1.355(2), C47−N3 1.424(2), C47−C48 1.359(3), C48−N4 1.387(3), C47−B2 1.656(3), N1−C1−N2 107.48(15), N1−C1−Br 127.85(13), N2−C1−Br 124.52(13), N3−C46−N4 102.50(16), C1−Br−C46 177.40(6) interplanar angle of the NHC planes: 35.65(8).

Refinement special details

One molecule of chlorobenzene is disordered over two positions in a 1:1 fashion and had to be restrained employing FragmentDB[10] as implemented in OLEX2.[11]

3.18. [(WCA-IDipp)H] (9a)

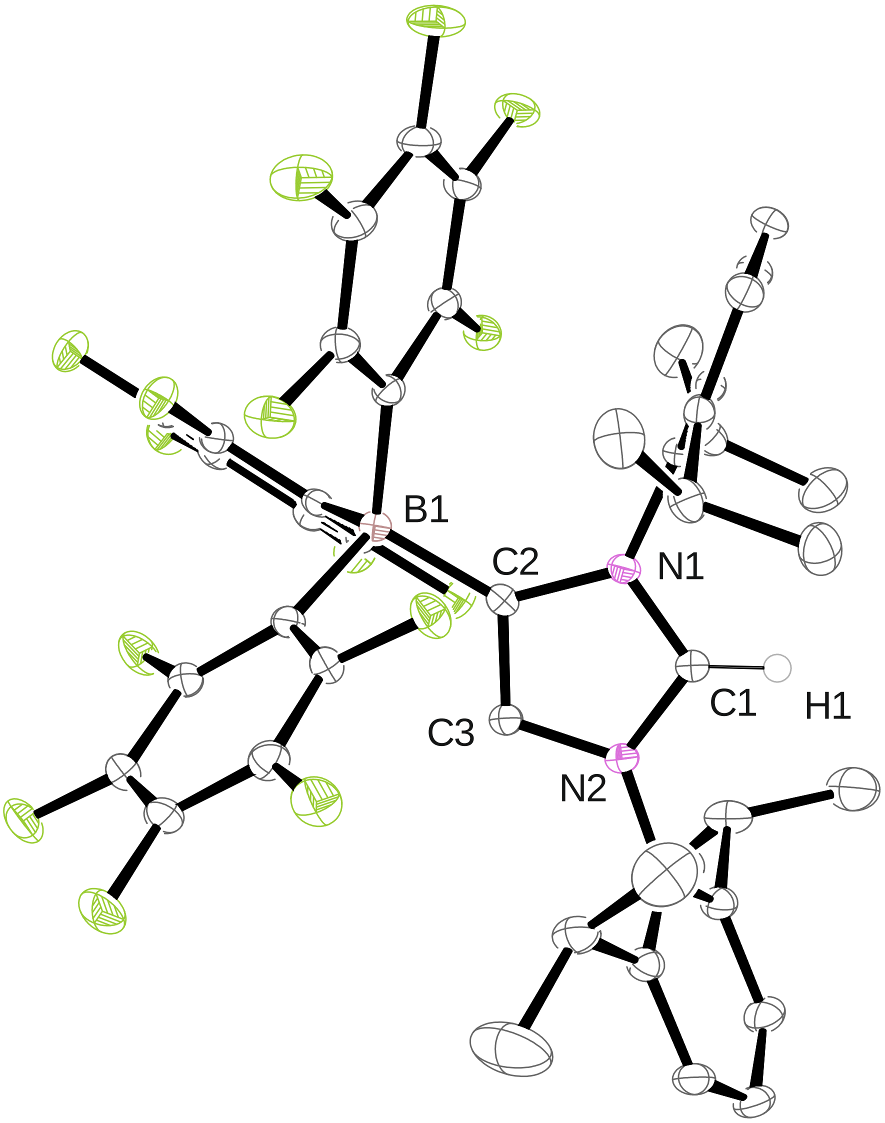

Figure S23. ORTEP diagram of **[(WCA-IDipp)H] (9a·2THF)** with thermal displacement parameters drawn at 50% probability. All solvent molecules and hydrogen atoms are omitted for clarity. Selected bond lengths [Å] and angles [°]: C1−N1 1.3383(19), C1−N2 1.330(2), N1−C2 1.411(2), C2−C3 1.367(2), C3−N2 1.377(2), C2−B1 1.657(2), N1−C1−N2 108.27(14), C1−N1−C2 110.05(12), N1−C2−C3 103.73(13), C2−C3−N2 109.49(14), C3−N2−C1 108.45(13).

3.19. [(WCA-IMes)H] (9b)

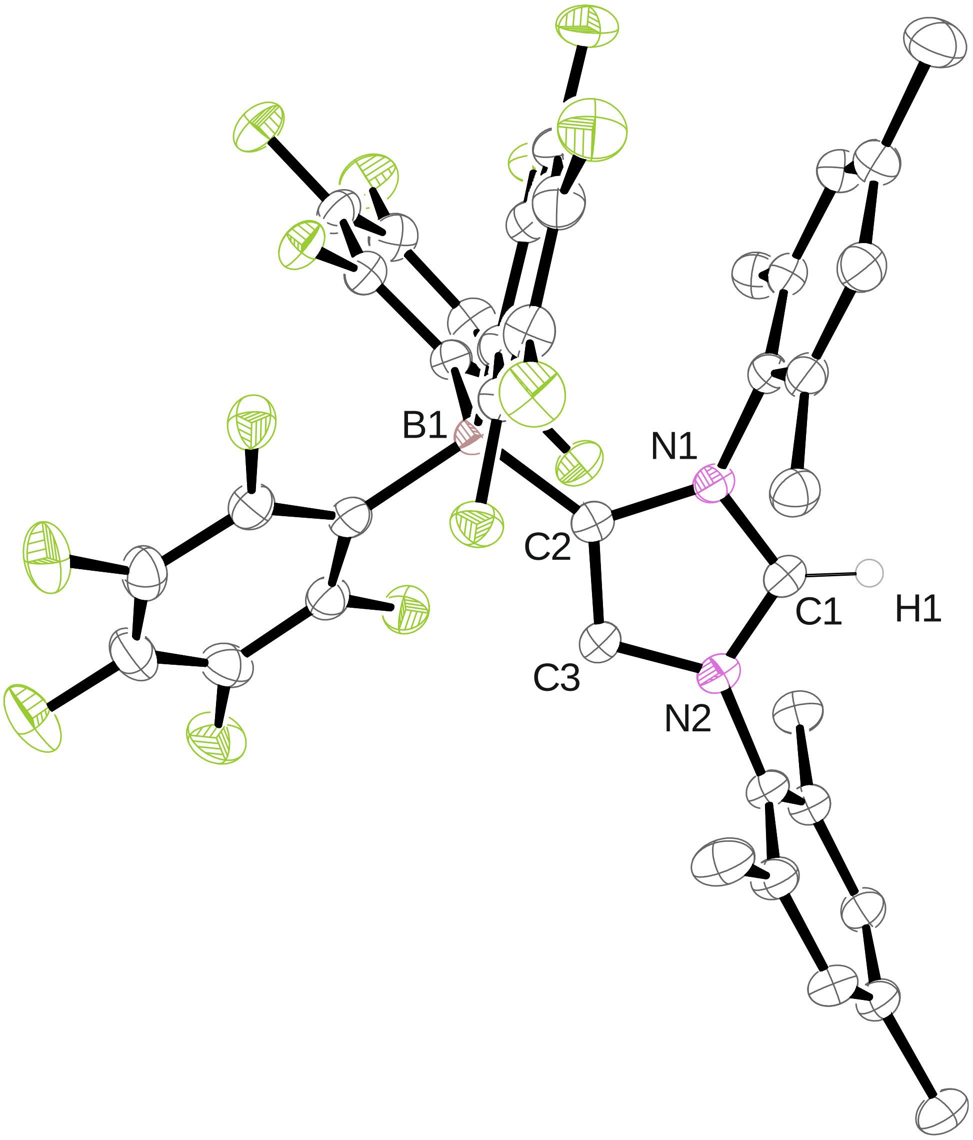

Figure S24. ORTEP diagram of **[(WCA-IMes)H] (9b·CH$_3$CN)** with thermal displacement parameters drawn at 50% probability. All solvent molecules and hydrogen atoms are omitted for clarity. Selected bond lengths [Å] and angles [°]: C1−N1 1.3340(16), C1−N2 1.3255(17), N1−C2 1.4161(16), C2−C3 1.3647(17), C3−N2 1.3845(17), C2−B1 1.6391(18), N1−C1−N2 109.00(11), C1−N1−C2 109.51(10), N1−C2−C3 104.12(11), C2−C3−N2 109.13(11), C3−N2−C1 108.22(10).

3.20. [IDipp$_2$I]I

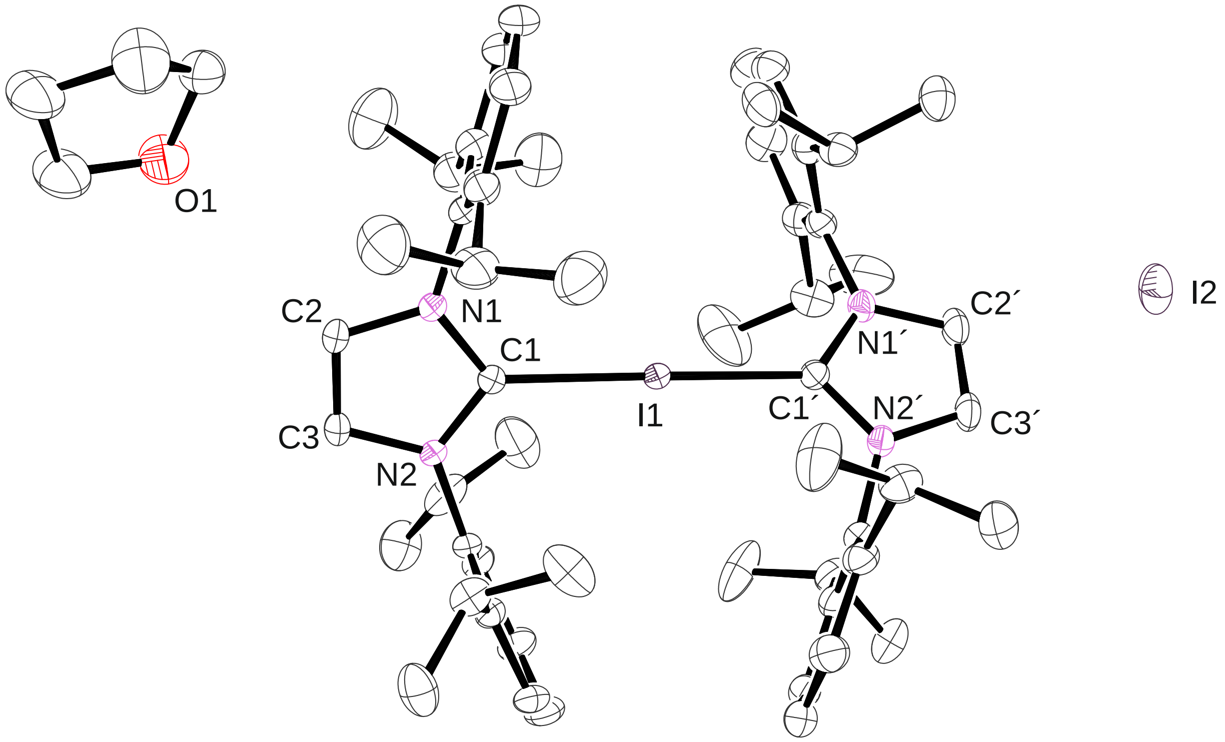

Figure S25. ORTEP diagram of **[IDipp$_2$I]I·THF** with thermal displacement parameters drawn at 50% probability. All hydrogen atoms are omitted for clarity. Selected bond lengths [Å] and angles [°]: C1−I 2.362(4), C1´−I 2.309(4), C1−N1 1.353(5), C1−N2 1.341(5), N1−C2 1.392(5), C2−C3 1.348(6), C3−N2 1.393(5), C1´−N1´ 1.352(5), C1´−N2´ 1.353(5), N1´−C2´ 1.391(5), C2´−C3´ 1.338(7), C3´−N2´ 1.384(5), N1−C1−N2 104.8(3) , N1−C1−I 128.9(3), N2−C1−I 126.2(3), N1´−C1´−N2´ 104.8(3), N1´−C1´−I 127.7(3), N2´−C1´−I 127.3(3), C1−I−C1´ 178.76(15).

Refinement special details

Remaining electron density is in close proximity to Iodine and can therefore most likely be accounted to absorption effects.

3.21. [I^{t}BuH][(WCA-IDipp)$_2$I]

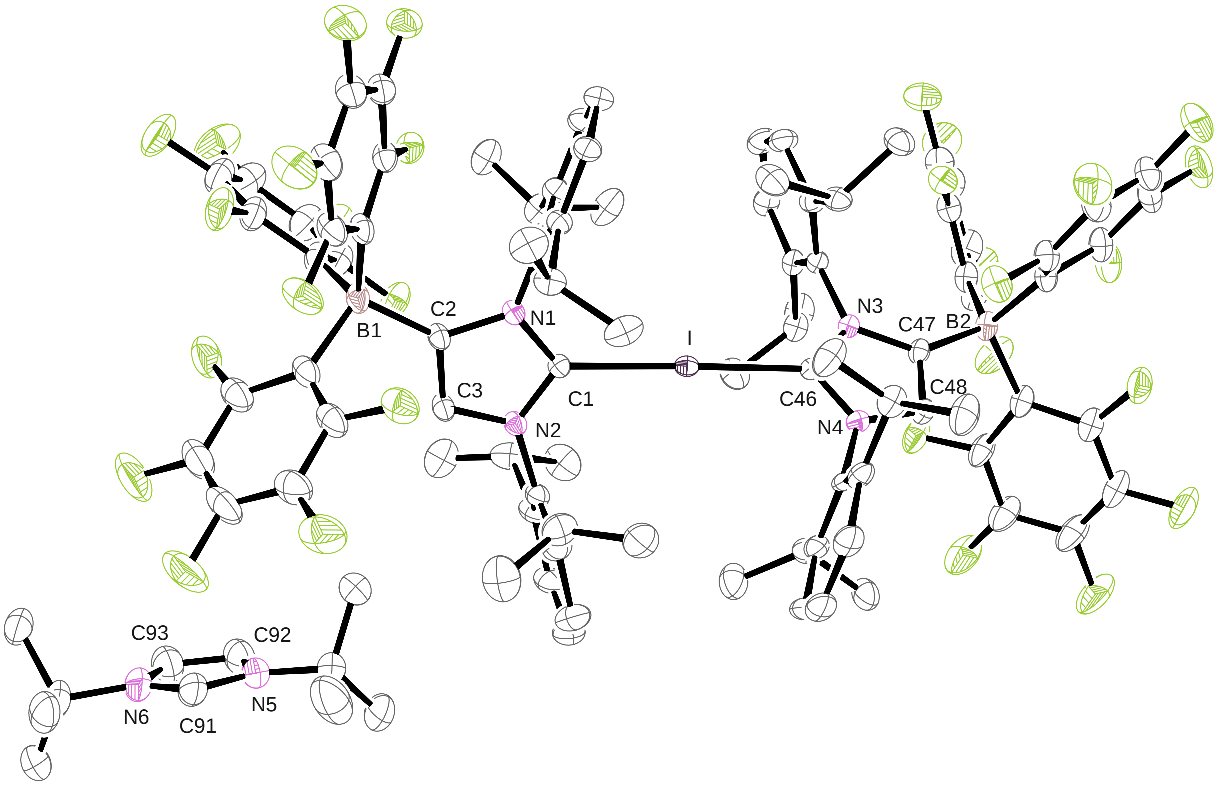

Figure S26. ORTEP diagram of **[I^{t}BuH][(WCA-IDipp)$_2$I]** with thermal displacement parameters drawn at 50% probability. All THF molecules, all hydrogen atoms and alternative positions of the Dipp group and *t*-Butyl groups are omitted for clarity. Selected bond lengths [Å] and angles [°]: C1−I 2.378(2), C46−I 2.365(2), C1−N1 1.363(3), C1−N2 1.346(3), N1−C2 1.417(3), C2−C3 1.361(3), C3−N2 1.384(3), C2−B1 1.662(3), C46−N3 1.368(3), C46−N4 1.345(3), C47−N3 1.421(3), C47−C48 1.360(3), C48−N4 1.384(3), C47−B2 1.652(3), C91−N5 1.338(4), C91−N6 1.330(5), N5−C92 1.379(5), C92−C93 1.349(5), C93−N6 1.376(5), N1−C1−N2 104.88(17), N1−C1−I 131.35(14), N2−C1−I 123.77(14), N3−C46−N4 104.64(17), N3−C46−I 130.73(14), N4−C46−I 124.37(14), C1−I−C46 178.67(7), N5−C91−N6 108.2(3).

Refinement special details

5 THF molecules had to be restrained or refined isotropic to yield a stable refinement. Disorder in Dipp/tBu was treated accordingly.

3.22. Table S1 (Part 1): Crystallographic Details

	[(WCA-IDipp)I·Toluene] (2a·C_6H_5Me)	[(WCA-IDipp)I·Chlorobenzene] (2a·C_6H_5Cl)	[(WCA-IDipp)I·THF] (2a·THF)
Empirical formula	$C_{52}H_{43}BF_{15}IN_2$	$C_{51}H_{40}BClF_{15}IN_2$	$C_{49}H_{43}BF_{15}IN_2O$
CCDC number	2034610	2034632	2034619
Formula weight [g mol^{-1}]	1118.59	1139.01	1098.56
Wavelength λ [Å]	0.71073	0.71073	0.71073
Temperature T [K]	103(2) K	100(2)	100.00(10)
Crystal size [mm^3]	0.265×0.232×0.202	0.272×0.250×0.062	0.670×0.418×0.316
Crystal system	monoclinic	monoclinic	monoclinic
Space group	$P2_1/c$	$P2_1/c$	$P2_1$
a [Å]	15.0224(2)	15.0350(2)	10.7140(2)
b [Å]	16.9909(2)	16.9420(2)	19.8170(6)
c [Å]	20.0892(2)	20.1360(2)	11.2270(2)
α [°]	90	90	90
β [°]	111.491(2)	111.780(2)	104.050(2)
γ [°]	90	90	90
Volume [Å^3]	4771.15(11)	4762.96(11)	2312.40(9)
Z	4	4	2
ρ_{calc} [Mg m^{-3}]	1.557	1.588	1.578
μ [mm^{-1}]	0.769	0.826	0.793
F (000)	2248	2280	1104
Reflections collected	286251	350848	118082
Independent reflections (R_{int})	12830 (0.0445)	23081 (0.0439)	11968 (0.0453)
GoF on F^2	1.038	1.032	1.029
R_1 ($I > 2\sigma(I)$)	0.0247	0.0278	0.0215
ωR_2 (all refl.)	0.0634	0.0785	0.0215
Theta range for data collection [°]	2.179−29.130	2.640−36.319	2.213−28.698
$\Delta\rho$ [e Å^3] min/max	0.776 /−0.746	0.796/−0.573	0.875/−0.495
crystallization details	saturated toluene/n-hexane solution at rt	toluene rt	from saturated n-hexane/THF/CH_3CN solution at RT

Table S1 (Part 2): Crystallographic Details

	[(WCA-IDipp)I·ONMe$_3$] (2a·ONMe$_3$)	[(WCA-IDipp)I·CH$_3$CN] (2a·2CH$_3$CN·C$_6$H$_5$Cl)	[(WCA-IMes)I] (2b)
Empirical formula	$C_{51}H_{51}BF_{15}IN_3O$	$C_{55}H_{46}BClF_{15}IN_4$	$C_{46}H_{31}BF_{15}IN_2$
CCDC number	2034625	2034624	2034606
Formula weight [g mol^{-1}]	1144.66	1221.12	1034.44
Wavelength λ [Å]	0.71073	0.71073	0.71073
Temperature T [K]	100(2)	100(2)	108(5)
Crystal size [mm^3]	0.380 × 0.260 ×0.150	0.350 × 0.296 × 0.178	0.303×0.192×0.121
Crystal dystem	triklin	triklin	Triclinic
Space group	$P\bar{1}$	$P\bar{1}$	$P\bar{1}$
a [Å]	10.8728(1)	12.3846(2)	12.4069(4)
b [Å]	13.2350(1)	12.7929(2)	14.3701(5)
c [Å]	18.8996(2)	17.6142(2)	14.8577(5)
α [°]	108.355(1)	81.361(1)	113.485(3)
β [°]	102.327(1)	81.482(1)	100.763(3)
γ [°]	94.931(1)	83.241(1)	108.665(3)
Volume [Å^3]	2487.15(4)	2715.58(7)	2145.91(14)
Z	2	2	2
ρ_{calc} [Mg m^{-3}]	1.528	1.493	1.601
μ [mm^{-1}]	0.741	0.731	0.848
F (000)	1158	1228	1028
Reflections collected	775015	239659	112392
Independent reflections (R_{int})	28091 (0.0527)	25698 (0.0334)	11531 (0.0362)
GoF on F^2	1.095	1.032	1.029
R_1 ($I > 2\sigma(I)$)	0.0269	0.0405	0.0230
ωR_2 (all refl.)	0.0769	0.1140	0.0559
Theta range for data collection [°]	2.578−38.568	2.675−36.957	2.371−29.126
$\Delta\rho$ [e Å^3] min/max	1.744/−0.851	2.373/−1.881	0.515/−0.410
crystallization details	chlorobenzene/*n*-hexane at RT	chlorobenzene/*n*-hexane/CH_3CN at −40°C	saturated toluene/*n*-hexane at rt

Table S1 (Part 3): Crystallographic Details

	[(*m*-XyF_6)$_3$B(IDipp)I] (2c)	[(WCA-IDipp)Br] (3a)	[(WCA-IMes)Br] (3b)
Empirical formula	$C_{51}H_{44}BF_{18}IN_2$	$C_{45}H_{35}BBrF_{15}N_2$	$C_{39}H_{23}BBrF_{15}N_2$
CCDC number	2034607	2034620	2034621
Formula weight [g mol^{-1}]	1164.59	979.47	895.31
Wavelength λ [Å]	0.71073	0.71073	0.71073
Temperature T [K]	99.90(14)	100(2)	100(2)
Crystal size [mm^3]	0.272×0.173×0.133	0.267×0.189×0.052	0.362×0.310×0.139
Crystal dystem	triclinic	triclinic	orthorhombic
Space group	$P\bar{1}$	$P\bar{1}$	*Pbca*
a [Å]	12.8280(6)	10.5300(8)	17.5470(4)
b [Å]	12.8890(4)	12.2450(8)	19.5230(4)
c [Å]	15.4030(6)	18.9180(10)	21.1890(4)
α [°]	97.420(2)	73.030(6)	90
β [°]	97.890(4)	74.550(6)	90
γ [°]	94.520(4)	65.640(6)	90
Volume [Å^3]	2489.39(17)	2095.3(3)	7258.7(3)
Z	2	2	8
ρ_{calc} [Mg m^{-3}]	1.554	1.552	1.639
μ [mm^{-1}]	0.749	1.084	1.243
F (000)	1168	988	3568
Reflections collected	134459	54108	257128
Independent reflections (R_{int})	15066 (0.0492)	10821 (0.0545)	10185 (0.0647)
GoF on F^2	1.045	1.029	1.026
R_1 ($I > 2\sigma(I)$)	0.0355	0.0419	0.0321
ωR_2 (all refl.)	0.0841	0.1050	0.0796
Theta range for data collection [°]	2.157−31.125	2.154−28.699	2.246−29.575
$\Delta\rho$ [e Å^3] min/max	1.263/−0.884	0.444/−0.719	0.423/−0.574
crystallization details	cooling of a saturated THF/*n*-hexane solution	cooling of saturated *n*-hexane/chlorobenzene solution	chlorobenzene/*n*-hexane at RT

Table S1 (Part 4): Crystallographic Details

	**[(WCA-IDipp)Cl] (4)**	**[(WCA-IDipp)I(IDipp)] (5a·C_6H_5Cl)**	**[(WCA-IDipp)I(IMes)] (5b·C_6H_5Cl·*n*-hexane)**
Empirical formula	$C_{49}H_{35}BClD_8F_{15}N_2O$	$C_{78}H_{76}BClF_{15}IN_4$ [+ solvent]	$C_{75}H_{75.5}BCl_{0.5}F_{15}IN_4$
CCDC number	2034617	2034623	2034626
Formula weight [g mol^{-1}]	1015.16	1527.58	1473.32
Wavelength λ [Å]	0.71073	0.71073	0.71073
Temperature T [K]	100(2)	100(2)	100(2)
Crystal size [mm^3]	0.663×0.134×0.109	0.280×0.190×0.111	0.156 × 0.066 × 0.059
Crystal dystem	monoclinic	triclinic	monoclinic
Space group	$P2_1/n$	$P\bar{1}$	$P2_1/n$
a [Å]	10.6410(4)	14.2857(4)	14.6899(5)
b [Å]	19.9370(8)	15.1760(4)	23.6085(7)
c [Å]	21.4150(8)	18.8369(6)	21.3872(7)
α [°]	90	100.253(3)	90
β [°]	101.750(4)	93.804(2)	100.574(3)
γ [°]	90	92.411(2)	90
Volume [Å^3]	4448.0(3)	4003.7(2)	7291.3(4)
Z	4	2	4
ρ_{calc} [Mg m^{-3}]	1.516	1.267	1.342
μ [mm^{-1}]	0.191	0.510	0.540
F (000)	2064	1564	3020
Reflections collected	138631	198216	157657
Independent reflections (R_{int})	13504 (0.0491)	24444 (0.0711)	21303 (0.0704)
GoF on F^2	1.027	1.030	1.038
R_1 ($I > 2\sigma(I)$)	0.0411	0.0499	0.0437
ωR_2 (all refl.)	0.1050	0.1341	0.1182
Theta range for data collection [°]	2.195−31.081	2.536−30.508	2.531−30.033
$\Delta\rho$ [e Å^3] min/max	0.512/−0.449	2.133/−1.119	1.038/−0.695
crystallization details	THF-d_8 /*n*-hexane at RT	chlorobenzene/n-hexane at −40 °C	chlorobenzene /*n*-hexane at RT

Table S1 (Part 5): Crystallographic Details

	**[PPh$_4$][(WCA-IDipp)] (6)**	**[PPh$_4$][(WCA-IDipp)$_2$I] (7a)**	**[PPh$_4$][(WCA-IDipp)I(WCA-IMes)] (7b)**
Empirical formula	$C_{69}H_{55}BF_{15}N_2P$	$C_{122}H_{106}B_2F_{30}IN_4O_2P$	$C_{108}H_{78}B_2F_{30}IN_4P$
CCDC number	2034630	2034618	2034631
Formula weight [g mol^{-1}]	1238.93	2409.59	2181.23
Wavelength λ [Å]	1.54184	0.71073	0.71073
Temperature T [K]	100(2)	100(2)	100(2)
Crystal size [mm^3]	0.250×0.216×0.127	0.182×0.160×0.119	0.272×0.179×0.081
Crystal dystem	triclinic	triclinic	triclinic
Space group	$P\bar{1}$	$P\bar{1}$	$P\bar{1}$
a [Å]	13.3588(3)	14.7723(2)	11.7635(2)
b [Å]	14.9063(3)	16.1697(2)	29.8462(6)
c [Å]	15.6930(3)	23.0247(4)	30.9299(6)
α [°]	75.214(2)	89.9490(10)	74.377(2)
β [°]	89.764(2)	82.7090(10)	87.058(2)
γ [°]	87.654(4)	89.9750(10)	81.635(2)
Volume [Å^3]	3018.87(11)	5455.29(14)	10346.1(4)
Z	2	2	4
ρ_{calc} [Mg m^{-3}]	1.363	1.467	1.400
μ [mm^{-1}]	1.191	0.415	0.428
F (000)	1276	2460	4408
Reflections collected	130664	380854	292937
Independent reflections (R_{int})	12777 (0.0303)	37964 (0.0430)	51326 (0.0421)
GoF on F^2	1.040	1.043	1.036
R_1 ($I > 2\sigma(I)$)	0.0438	0.0363	0.0354
ωR_2 (all refl.)	0.1228	0.0991	0.0906
Theta range for data collection [°]	2.912−77.827	2.672−32.043	3.296−76.182
$\Delta\rho$ [e Å^3] min/max	0.553/−0.513	1.225/−0.658	1.369/−0.746
crystallization details	THF/*n*-hexane at RT	toluene/*n*-hexane at RT	*n*-hexane/Toluene at RT

Table S1 (Part 6): Crystallographic Details

	$[PPh_4][(WCA\text{-}IDipp)_2Br]$ $(8\cdot C_6H_5Cl)$	$[IDipp_2I]I$	$[I^tBuH][(WCA\text{-}IDipp)_2I]\cdot(THF)_5$
Empirical formula	$C_{120}H_{95}B_2BrClF_{30}N_4P$	$C_{58}H_{80}I_2N_4O$	$C_{121}H_{131}B_2F_{30}IN_6O_5$
CCDC number	2034622	2034628	2034629
Formula weight [g mol^{-1}]	2330.94	1103.06	2467.83
Wavelength λ [Å]	0.71073	0.71073	0.71073
Temperature T [K]	100(2)	100(2)	100(2)
Crystal size [mm^3]	0.330×0.195×0.119	0.330×0.232×0.092	0.360×0.249×0.194
Crystal dystem	triclinic	tetragonal	monoclinic
Space group	$P\bar{1}$	$I4_1cd$	$P2_1/c$
a [Å]	15.8945(4)	25.7043(2)	19.31320(10)
b [Å]	17.4116(4)	25.7043(2)	24.99290(10)
c [Å]	20.0948(6)	34.0767(4)	23.76330(10)
α [°]	90.699(2)	90	90
β [°]	92.591(2)	90	93.5600(10)
γ [°]	107.383(2)	90	90
Volume [Å^3]	5299.9(2)	22514.9(4)	11448.24(9)
Z	2	16	4
ρ_{calc} [Mg m^{-3}]	1.461	1.302	1.432
μ [mm^{-1}]	0.534	1.157	0.386
F (000)	2380	9120	5088
Reflections collected	330318	426910	491472
Independent reflections (R_{int})	32357 (0.0488)	17179 (0.0587)	39850 (0.0435)
GoF on F^2	1.023	1.034	1.027
R_1 ($I > 2\sigma(I)$)	0.0543	0.0366	0.0632
ωR_2 (all refl.)	0.1543	0.0966	0.1792
Theta range for data collection [°]	2.510−30.508	2.506−30.507	2.549−32.032
$\Delta\rho$ [e Å^3] min/max	2.444/−1.621	3.810/−1.646	2.279/−1.292
crystallization details	chlorobenzene/*n*-hexane	THF/*n*-hexane at RT	slow evaporation of saturated THF solution

Table S1 (Part 7): Crystallographic Details

	[(WCA-IDipp)H] (9a·2THF)	[(WCA-IMes)H] (9b·CH_3CN)
Empirical formula	$C_{53}H_{52}BF_{15}N_2O_2$	$C_{41}H_{27}BF_{15}N_3$
CCDC number	2034609	2034608
Formula weight [g mol^{-1}]	1044.77	857.46
Wavelength λ [Å]	0.71073	1.54184
Temperature T [K]	100(2)	100(2)
Crystal size [mm^3]	0.25 × 0.25 × 0.20	0.20 × 0.20 × 0.05
Crystal dystem	triclinic	monoclinic
Space group	$P\bar{1}$	$P2_1/n$
a [Å]	12.0835(4)	11.0526(3)
b [Å]	12.1287(4)	16.9717(4)
c [Å]	18.8587(6)	20.2081(4)
α [°]	89.642(3)	90
β [°]	75.222(3)	91.260(2)
γ [°]	64.966(3)	90
Volume [Å^3]	2405.03(15)	3789.75(16)
Z	2	4
ρ_{calc} [Mg m^{-3}]	1.443	1.503
μ [mm^{-1}]	0.127	1.239
F (000)	1080	1736
Reflections collected	153520	60385
Independent reflections (R_{int})	14305 (0.0556)	7910 (0.0434)
GoF on F^2	1.034	1.019
R_1 ($I > 2\sigma(I)$)	0.0528	0.0348
ωR_2 (all refl.)	0.1342	0.0951
Theta range for data collection [°]	2.249−31.131	3.401−76.488
$\Delta\rho$ [e Å^3] min/max	0.889/−0.473	0.303/−0.282
crystallization details	THF/*n*-hexane	acetonitrile at −40°C

4. Computational Details

All computations were performed using the density functional method B97-D[13] (S. Grimme) and B3LYP[14] with and without D3 dispersion correction – D3 version of Grimme's dispersion with the original D3 damping function[15] – as implemented in the Gaussian09 program.[16] For all main group elements (C, H, B and F) Poples's all-electron triple-ζ basis set 6-311G**[17] was used, while for the halogen atoms chlorine, bromine and iodine, which are coordinated to the N-heterocyclic carbenes, a quasi-relativistic basis set *"Stuttgart-Koeln RLC ECP"* (46MWB)[18] was applied.[19] The QTAIM analysis of the wave function was carried out with the freely available program package *MultiWFN 3.7*.[20] Natural Bond Orbital (NBO) analysis (NBO charges, WBI) was accomplished using NBO version 3,[21] which is included in Gaussian09. Harmonic vibrational frequencies are calculated to characterize respective minima structures (with no imaginary frequency).

Table S2: Energies for all optimized structures according to the DFT method B97-D.

	compound	E_{0K}^{a} [Ha]	E_{298K}^{b} [Ha]	H_{298K}^{b} [Ha]	G_{298K}^{b} [Ha]
ligands	C_6H_5Cl	-691.672396	-691.666753	-691.665808	-691.702274
	C_6H_5Me	-271.301217	-271.294885	-271.293941	-271.331616
	THF	-232.237821	-232.232783	-232.231839	-232.266485
	Me_3NO	-249.409805	-249.403656	-249.402712	-249.438248
	MeCN	-132.653215	-132.649571	-132.648627	-132.676227
carbenes	[{F_5C_6}$_3$B-IDipp]$^-$	-3365.950185	-3365.889895	-3365.888951	-3366.045488
	[{F_5C_6}$_3$B-IMes]$^-$	-3130.340824	-3130.287596	-3130.286652	-3130.428661
	IDipp	-1158.937138	-1158.906667	-1158.905723	-1158.997859
	IMes	-923.329589	-923.305287	-923.304343	-923.386537
iodine adducts	({F_5C_6}$_3$B-IDipp)I	-3377.331718	-3377.269664	-3377.268720	-3377.428907
	[(IDipp)I]$^+$	-1170.221478	-1170.188700	-1170.187756	-1170.287444
	({F_5C_6}$_3$B-IDipp)I(C_6H_5Cl)	-4069.016864	-4068.947381	-4068.946437	-4069.125905
	({F_5C_6}$_3$B-IDipp)I(C_6H_5Me)	-3648.648676	-3648.578364	-3648.577419	-3648.758327
	({F_5C_6}$_3$B-IDipp)I(THF)	-3609.587240	-3609.518718	-3609.517773	-3609.693369
	({F_5C_6}$_3$B-IDipp)I($ONMe_3$)	-3626.770107	-3626.700271	-3626.699327	-3626.877297
	({F_5C_6}$_3$B-IDipp)I(NCMe)	-3509.995143	-3509.927409	-3509.926465	-3510.103940
	[({F_5C_6}$_3$B-IDipp)I(IDipp-B{C_6F_5}$_3$)]$^-$	-6743.361803	-6743.238378	-6743.237434	-6743.526274
	[({F_5C_6}$_3$B-IDipp)I(IMes-B{C_6F_5}$_3$)]$^-$	-6507.754807	-6507.637904	-6507.636960	-6507.915502
	({F_5C_6}$_3$B-IDipp)I(IDipp)	-4536.324107	-4536.230339	-4536.229395	-4536.454965
	({F_5C_6}$_3$B-IDipp)I(IMes)	-4300.714522	-4300.627011	-4300.626067	-4300.842586
	[(IDipp)I(IDipp)]$^+$	-2329.235929	-2329.170546	-2329.169602	-2329.339748
bromine add.	({F_5C_6}$_3$B-IDipp)Br	-3379.289177	-3379.227311	-3379.226367	-3379.385842
	[(IDipp)Br]$^+$	-1172.177569	-1172.144928	-1172.143984	-1172.243384
	[({F_5C_6}$_3$B-IDipp)Br(IDipp-B{C_6F_5}$_3$)]$^-$	-6745.305622	-6745.182175	-6745.181230	-6745.471438
	({F_5C_6}$_3$B-IDipp)Br(IDipp)	-4538.269550	-4538.176034	-4538.175090	-4538.400322
	[(IDipp)Br(IDipp)]$^+$	-2331.174890	-2331.109961	-2331.109017	-2331.276232
chlorine add.	({F_5C_6}$_3$B-IDipp)Cl	-3380.897240	-3380.835547	-3380.834603	-3380.993187
	[(IDipp)Cl]$^+$	-1173.784284	-1173.751638	-1173.750694	-1173.851292
	[({F_5C_6}$_3$B-IDipp)Cl(IDipp-B{C_6F_5}$_3$)]$^-$	-6746.896875	-6746.773299	-6746.772354	-6747.062937
	({F_5C_6}$_3$B-IDipp)Cl(IDipp)	-4539.868642	-4539.774975	-4539.774031	-4540.001124
	[(IDipp)Cl(IDipp)]$^+$	-2332.763867	-2332.699013	-2332.698069	-2332.865204

^{a} DFT energy incl. ZPE. ^{b} standard conditions T = 298.15 K and p = 1 atm.

Table S3: Energies for all optimized structures using DFT method B3LYP with and without D3 dispersion correction.

compound			E_{0K}[a] [Ha]	E_{298K}[b] [Ha]	H_{298K}[b] [Ha]	G_{298K}[b] [Ha]
carbenes	B3LYP-D3	[{F$_5$C$_6$}$_3$B-IDipp]$^-$	-3368.135069	-3368.075862	-3368.074918	-3368.229825
		[{F$_5$C$_6$}$_3$B-IMes]$^-$	-3132.344276	-3132.292243	-3132.291298	-3132.430087
		IDipp	-1159.834374	-1159.804379	-1159.803435	-1159.896216
		IMes	-924.047672	-924.024191	-924.023247	-924.103316
	B3LYP	[{F$_5$C$_6$}$_3$B-IDipp]$^-$	-3367.975809	-3367.915990	-3367.915046	-3368.071637
		[{F$_5$C$_6$}$_3$B-IMes]$^-$	-3132.220160	-3132.167143	-3132.166199	-3132.310176
		IDipp	-1159.757139	-1159.726640	-1159.725696	-1159.820280
		IMes	-924.001320	-923.977752	-923.976807	-924.055423
iodine adducts	B3LYP-D3	({F$_5$C$_6$}$_3$B-IDipp)I	-3379.477505	-3379.416784	-3379.415840	-3379.573300
		[(IDipp)I]$^+$	-1171.082895	-1171.051018	-1171.050074	-1171.147939
		[({F$_5$C$_6$}$_3$B-IDipp)I(IDipp-B{C$_6$F$_5$}$_3$)]$^-$	-6747.688319	-6747.567120	-6747.566175	-6747.851424
		[({F$_5$C$_6$}$_3$B-IDipp)I(IMes-B{C$_6$F$_5$}$_3$)]$^-$	-6511.902782	-6511.787645	-6511.786701	-6512.063895
		({F$_5$C$_6$}$_3$B-IDipp)I(IDipp)	-4539.365980	-4539.273997	-4539.273053	-4539.495977
		({F$_5$C$_6$}$_3$B-IDipp)I(IMes)	-4303.577856	-4303.491550	-4303.490606	-4303.707252
		[(IDipp)I(IDipp)]$^+$	-2330.990350	-2330.927102	-2330.926158	-2331.090262
	B3LYP	({F$_5$C$_6$}$_3$B-IDipp)I	-3379.302869	-3379.241168	-3379.240224	-3379.400635
		[(IDipp)I]$^+$	-1170.993687	-1170.961261	-1170.960317	-1171.060509
		[({F$_5$C$_6$}$_3$B-IDipp)I(IDipp-B{C$_6$F$_5$}$_3$)]$^-$	-6747.309694	-6747.186324	-6747.185379	-6747.479027
		[({F$_5$C$_6$}$_3$B-IDipp)I(IMes-B{C$_6$F$_5$}$_3$)]$^-$	-6511.569684	-6511.452888	-6511.451944	-6511.735393
		({F$_5$C$_6$}$_3$B-IDipp)I(IDipp)	-4539.076867	-4538.982742	-4538.981798	-4539.214364
		({F$_5$C$_6$}$_3$B-IDipp)I(IMes)	-4303.326357	-4303.238600	-4303.237655	-4303.459669
		[(IDipp)I(IDipp)]$^+$	-2330.789260	-2330.723966	-2330.723022	-2330.897987
bromine adducts	B3LYP-D3	({F$_5$C$_6$}$_3$B-IDipp)Br	-3381.436989	-3381.376257	-3381.375312	-3381.533707
		[(IDipp)Br]$^+$	-1173.040182	-1173.008199	-1173.007255	-1173.106253
		[({F$_5$C$_6$}$_3$B-IDipp)Br(IDipp-B{C$_6$F$_5$}$_3$)]$^-$	-6749.632077	-6749.511169	-6749.510224	-6749.794854
		({F$_5$C$_6$}$_3$B-IDipp)Br(IDipp)	-4541.314510	-4541.222526	-4541.221582	-4541.445406
		[(IDipp)Br(IDipp)]$^+$	-2332.931266	-2332.867850	-2332.866906	-2333.032247
	B3LYP	({F$_5$C$_6$}$_3$B-IDipp)Br	-3381.265446	-3381.203647	-3381.202703	-3381.364216
		[(IDipp)Br]$^+$	-1172.953201	-1172.920992	-1172.920047	-1173.019167
		[({F$_5$C$_6$}$_3$B-IDipp)Br(IDipp-B{C$_6$F$_5$}$_3$)]$^-$	-6749.257627	-6749.133818	-6749.132873	-6749.429500
		({F$_5$C$_6$}$_3$B-IDipp)Br(IDipp)	-4541.030161	-4540.935939	-4540.934994	-4541.169111
		[(IDipp)Br(IDipp)]$^+$	-2332.733367	-2332.668372	-2332.667428	-2332.841268
chlorine adducs	B3LYP-D3	({F$_5$C$_6$}$_3$B-IDipp)Cl	-3383.049200	-3382.988785	-3382.987841	-3383.144334
		[(IDipp)Cl]$^+$	-1174.650501	-1174.618792	-1174.617848	-1174.715415
		[({F$_5$C$_6$}$_3$B-IDipp)Cl(IDipp-B{C$_6$F$_5$}$_3$)]$^-$	-6751.232081	-6751.111260	-6751.110315	-6751.395095
		({F$_5$C$_6$}$_3$B-IDipp)Cl(IDipp)	-4542.919595	-4542.827609	-4542.826665	-4543.052572
		[(IDipp)Cl(IDipp)]$^+$	-2334.530209	-2334.467280	-2334.466336	-2334.630055
	B3LYP	({F$_5$C$_6$}$_3$B-IDipp)Cl	-3382.881090	-3382.819583	-3382.818639	-3382.979229
		[(IDipp)Cl]$^+$	-1174.565866	-1174.533906	-1174.532962	-1174.631070
		[({F$_5$C$_6$}$_3$B-IDipp)Cl(IDipp-B{C$_6$F$_5$}$_3$)]$^-$	-6750.868830	-6750.744703	-6750.743759	-6751.046476
		({F$_5$C$_6$}$_3$B-IDipp)Cl(IDipp)	-4542.641706	-4542.547225	-4542.546281	-4542.785030
		[(IDipp)Cl(IDipp)]$^+$	-2334.335340	-2334.270465	-2334.269521	-2334.444109

[a] DFT energy incl. ZPE.
[b] standard conditions T = 298.15 K and p = 1 atm.

Table S4: Natural Bond Orbital (NBO) charges (*q*) and Wiberg Bond Indices (WBI) of ($\{F_5C_6\}_3$B-IDipp)X and ($\{F_5C_6\}_3$B-IDipp)$_2$X]$^-$ (X = I, Br, Cl).

compound	X	WBI	NBO charges	
		X–C_{CHN}	*q*(X)	*q*(C_{NHC})
($\{F_5C_6\}_3$B-IDipp)**I**	iodine	1.039	0.30	0.23
[($\{F_5C_6\}_3$B-IDipp)$_2$**I**]$^-$		0.537 / 0.537 (Σ1.074)	0.22	0.19 / 0.19
($\{F_5C_6\}_3$B-IDipp)**Br**	bromine	1.101	0.19	0.33
[($\{F_5C_6\}_3$B-IDipp)$_2$**Br**]$^-$		0.542 / 0.533 (Σ1.075)	0.04	0.27 / 0.27
($\{F_5C_6\}_3$B-IDipp)**Cl**	chlorine	1.112	0.09	0.42
[($\{F_5C_6\}_3$B-IDipp)$_2$**Cl**]$^-$		0.976 / 0.136 (Σ1.112)	0.06	0.41 / 0.13

Figure S27. MO diagram for the formation of [($\{F_5C_6\}_3$B-IDipp)$_2$I]$^-$ from ($\{F_5C_6\}_3$B-IDipp)I and [$\{F_5C_6\}_3$B-IDipp]$^-$ calculated at the B97-D level of theory. The energies of the two fragments were obtained by single-point calculations.

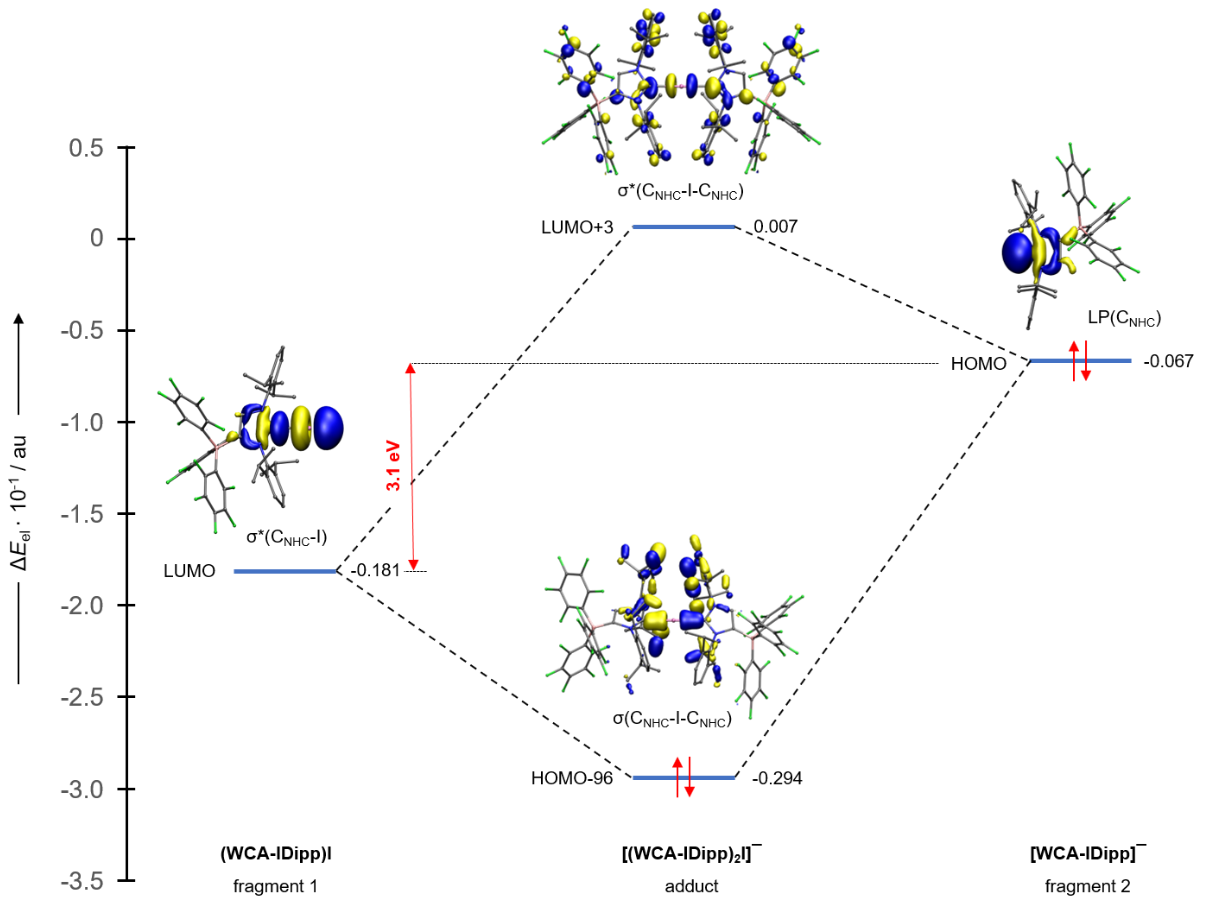

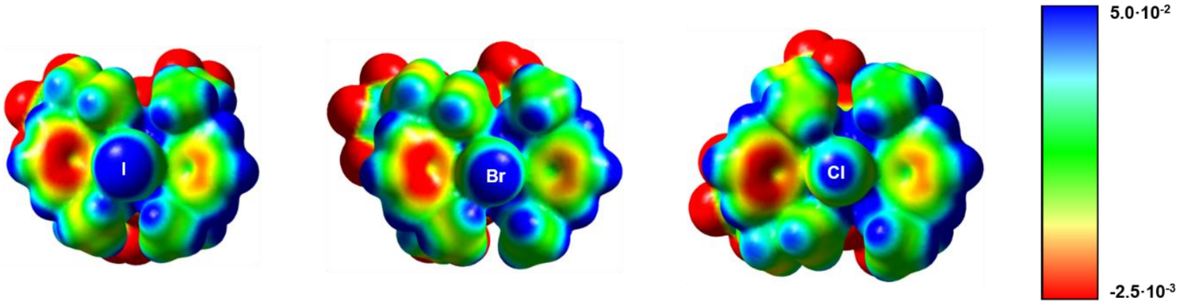

Figure S28. Calculated (B97-D) electrostatic potential (ESP) of ({F_5C_6}$_3$B-IDipp)X (X = I, Br, Cl) projected on the 0.004 e a_0^{-3} contour of the electron density using the same colour scale of –0.0025 (red) up to 0.050 au (blue).

5. Literature

[1] G. R. Fulmer, A. J. M. Miller, N. H. Sherden, H. E. Gottlieb, A. Nudelman, B. M. Stoltz, J. E. Bercaw, K. I. Goldberg, *Organometallics*, **2010**, *29*, 2176.

[2] R. K. Harris, E. D. Becker, S. M. C. de Menezes, P. Granger, R. E. Hoffman, K. W. Zilm, *Magn. Reson. Chem.* **2008**, *46*, 582.

[3] a) S. Kronig, E. Theuergarten, C. G. Daniliuc, P. G. Jones, M. Tamm, *Angew. Chem. Int. Ed.* **2012**, *51*, 3240.

[4] E. L. Kolychev, S. Kronig, K. Brandhorst, M. Freytag, P. G. Jones, M. Tamm, *J. Am. Chem. Soc.* **2013**, *135*, 12448.

[5] S. Kronig, *Dissertation*, Technische Universität Braunschweig, Braunschweig, 2012.

[6] CrysAlisPRO, Oxford Diffraction/Agilent Technologies UK Ltd, Yarnton, England.

[7] G. M. Sheldrick, Acta Cryst., **2008**, A64, 112-122.

[8] G. M. Sheldrick, *Acta Crystallogr., A,* **2015**, *71*, 3.

[9] G. M. Sheldrick, *Acta Crystallogr., C,* **2015**, *71*, 3.

[10] D. Kratzert, I. Krossing, J., *Appl. Cryst* **2018**, *51*, 928-934

[11] O. V. Dolomanov, L. J. Bourhis, R. J. Gildea, J. A. K. Howard, H. Puschmann, J. Appl. Cryst.,2009, 42, 339-341.

[12] A. L. Spek, Acta Cryst. 2015, C71, 9.

[13] S. Grimme, *J. Comp. Chem.* **2006**, *27*, 1787–1799.

[14] (a) A. D. Becke, *J. Chem. Phys.* **1993**, *98*, 5648–5652; (b) B. Miehlich, A. Savin, H. Stoll, H. Preuss, *Chem. Phys. Lett.* **1989**, *157*, 200–206; (c) C. Lee, W. Yang, G. Parr, *Phys. Rev. B* **1988**, *37*, 785–789.

[15] S. Grimme, J. Antony, S. Ehrlich, H. Krieg, *J. Chem. Phys*. **2010**, *132*, 154104–154119.

[16] Gaussian 09, Revision A.1, M. J. Frisch, G. W. Trucks, H. B. Schlegel, G. E. Scuseria, M. A. Robb, J. R. Cheeseman, G. Scalmani, V. Barone, B. Mennucci, G. A. Petersson, H. Nakatsuji, M. Caricato, X. Li, H. P. Hratchian, A. F. Izmaylov, J. Bloino, G. Zheng, J. L. Sonnenberg, M. Hada, M. Ehara, K. Toyota, R. Fukuda, J. Hasegawa, M. Ishida, T. Nakajima, Y. Honda, O. Kitao, H. Nakai, T. Vreven, J. A. Montgomery, Jr., J. E. Peralta, F. Ogliaro, M. Bearpark, J. J. Heyd, E. Brothers, K. N. Kudin, V. N. Staroverov, R. Kobayashi, J. Normand, K. Raghavachari, A. Rendell, J. C. Burant, S. S. Iyengar, J. Tomasi, M. Cossi, N. Rega, J. M. Millam, M. Klene, J. E. Knox, J. B.

Cross, V. Bakken, C. Adamo, J. Jaramillo, R. Gomperts, R. E. Stratmann, O. Yazyev, A. J. Austin, R. Cammi, C. Pomelli, J. W. Ochterski, R. L. Martin, K. Morokuma, V. G. Zakrzewski, G. A. Voth, P. Salvador, J. J. Dannenberg, S. Dapprich, A. D. Daniels, Ö. Farkas, J. B. Foresman, J. V. Ortiz, J. Cioslowski and D. J. Fox, Gaussian, Inc., Wallingford CT, **2009**.

[17] a) R. Krishnan, J. S. Binkley R. Seeger, J. A. Pople, *J. Chem. Phys.* **1980**, *72*, 650–654; b) A. D. McLean, G. S. Chandler, *J. Chem. Phys.* **1980**, *72*, 5639–5648; c) A. J. H. Wachters, *J. Chem. Phys.* **1970**, *52*, 1033–1036.

[18] A. Bergner, M. Dolg, W. Kuechle, H. Stoll, H. Preuss, *Mol. Phys.* **1993**, *80*, 1431–1441.

[19] The quasi-relativistic basic set for the three halogen atoms chlorine, bromine and iodine was obtained from*"Basis Set Exchange"* at [https://www.basissetexchange.org/]. B. P. Pritchard, D. Altarawy, B. Didier, T. D. Gibson, T. L. Windus. *J. Chem. Inf. Model.* **2019**, *59*, 4814–4820.

[20] T. Lu, F. Chen, *J. Comput. Chem*. **2012**, *33*, 580–592.

[21] a) J. P. Foster, F. Weinhold, *J. Am. Chem. Soc.* **1980**, *102*, 7211–7218; b) A. E. Reed, F. Weinhold, *J. Chem. Phys.* **1983**, *78*, 4066–4073; c) A. E. Reed, R. B. Weinstock, F. Weinhold, *J. Chem. Phys.* **1985**, *83*, 735–746; d) A. E. Reed, F. Weinhold, *J. Chem. Phys.* **1985**, *83*, 1736–1740.
